# Design, Synthesis, and Cytotoxicity and Topoisomerase I/IIα Inhibition Activity of Pyrazolo[4,3-*f*]quinoline Derivatives

**DOI:** 10.3390/ph15040399

**Published:** 2022-03-24

**Authors:** Chhabi Lal Chaudhary, Seungyun Ko, Chaerim Lee, Yerin Kim, Chanhyun Jung, Soonsil Hyun, Youngjoo Kwon, Jong-Soon Kang, Jae-Kyung Jung, Heesoon Lee

**Affiliations:** 1 College of Pharmacy and Medicinal Research Center (MRC), Chungbuk National University, Cheongju 28160, Korea; chhabichaudhary319@gmail.com (C.L.C.); gozzang05@gmail.com (S.K.); ihl0905@gmail.com (C.L.); ggp02019@naver.com (Y.K.); cksgus9823@naver.com (C.J.); shyun@chungbuk.ac.kr (S.H.); orgjkjung@chungbuk.ac.kr (J.-K.J.); 2 College of Pharmacy, Ewha Womans University, Seoul 03760, Korea; ykwon@ewha.ac.kr; 3 Korea Research Institute of Bioscience and Biotechnology, Daejeon 28116, Korea; kanjon@kribb.re.kr

**Keywords:** pyrazolo[4,3-*f*]quinoline derivatives, imino Diels–Alder reaction, anticancer agents, cytotoxic effect, human topoisomerase I and IIα inhibitors

## Abstract

With the several targets of cancer treatment, inhibition of DNA topoisomerase activity is one of the well-known focuses in cancer chemotherapy. Here, we describe the design and synthesis of a novel series of pyrazolo[4,3-*f*]quinolines with potential anticancer/topoisomerase inhibition activity. Forty newly designed pyrazolo[4,3-*f*]quinoline derivatives were synthesized via inverse imino Diels–Alder reaction. The antiproliferative activity of the synthesized derivatives was initially measured in the human NUGC-3 cancer cell line. Then, the selected compounds **1B**, **1C**, **1M**, **2A**, **2D**, **2E**, **2F**, and **2R** with higher activity among tested compounds were screened against six cancer cell lines, including ACHN, HCT-15, MM231, NCI-H23, NUGC-3, and PC-3. The results demonstrated that the compounds **1M**, **2E**, and **2P** were most effective in all cancer cell lines exhibiting GI_50_ below 8 µM. Among them, **2E** showed an equivalent inhibition pattern of topoisomerase IIα activity to that of etoposide, positive control at a 100 µM dose.

## 1. Introduction

Cancer is expected to be the leading cause of death and the only most critical barrier to increasing life expectancy in the 21st century due to its precipitously rising incidence and mortality rate globally [1,2,3]. Regardless of enormous efforts and achievements in cancer management and prophylaxis, the development of resistance to traditional chemotherapeutic drugs and/or novel targeted drugs, cancer cell selectivity, and relatively high toxicity remain a significant challenge [4,5]. As a result, the continued advancement of novel, more selective chemotherapeutics, as well as novel biological targets, notably for the most aggressive tumors, is highly desirable to address current and future treatment goals [6,7,8].

DNA topoisomerases (topos) are nuclear enzymes that help to restore DNA topology by relieving torsional strains produced during replication, transcription, segregation, and recombination [9,10]. Among two types, in the absence of ATP and Mg, human type I topoisomerase (topo I) splits and rejoins a single DNA strand while in the presence of ATP and Mg, type II (topo II), which behaves as a homodimer, breaks, and rejoins the double DNA strand. Based on their nuclear activities, topo II is further subdivided into two isoforms: topo IIα, which is frequently linked to proliferating cells and topo Iiβ, which is independent of cell proliferation [11,12,13,14]. Despite the fact that topoisomerase inhibitors such as etoposide, camptothecin, and irinotecan have been used as anticancer drugs for decades, they have well-defined shortcomings, such as dose-limiting toxicities like myelosuppression and diarrhea. Similarly, topo II inhibitors are also known to cause major side effects, such as cardiomyopathy and secondary leukemia [15,16,17,18]. In rapidly proliferating tumor cells, activities of topo enzymes are generally elevated. In this vain, topos have remained a promising target in medicinal chemistry due to high antitumor selectivity when compared with other DNA damaging agents [7,19].

Camptothecin and its structural derivatives have been studied extensively in the treatment of colon, ovarian, and lung malignancies in both research and clinical trials. However, as the lactone ring opens at physiological pH, they have substantial off-target consequences, such as poor solubility, limited potency, and structural instability [20]. With these considerations in mind and our long-standing interest in synthesizing heterocyclic motif carrying biological applications, our group reported a series of novel angularly fused pentacyclic scaffolds, such as 1,3-diphenylbenzo[*f*][1,7]naphthyridines, and 13*H*-benzo[*f*]chromeno[4,3-*b*][1,7]naphthyridines for their cytotoxic activities against cancer cells and topoisomerase enzyme activities in the past few years [21,22,23].

**Figure 1:**
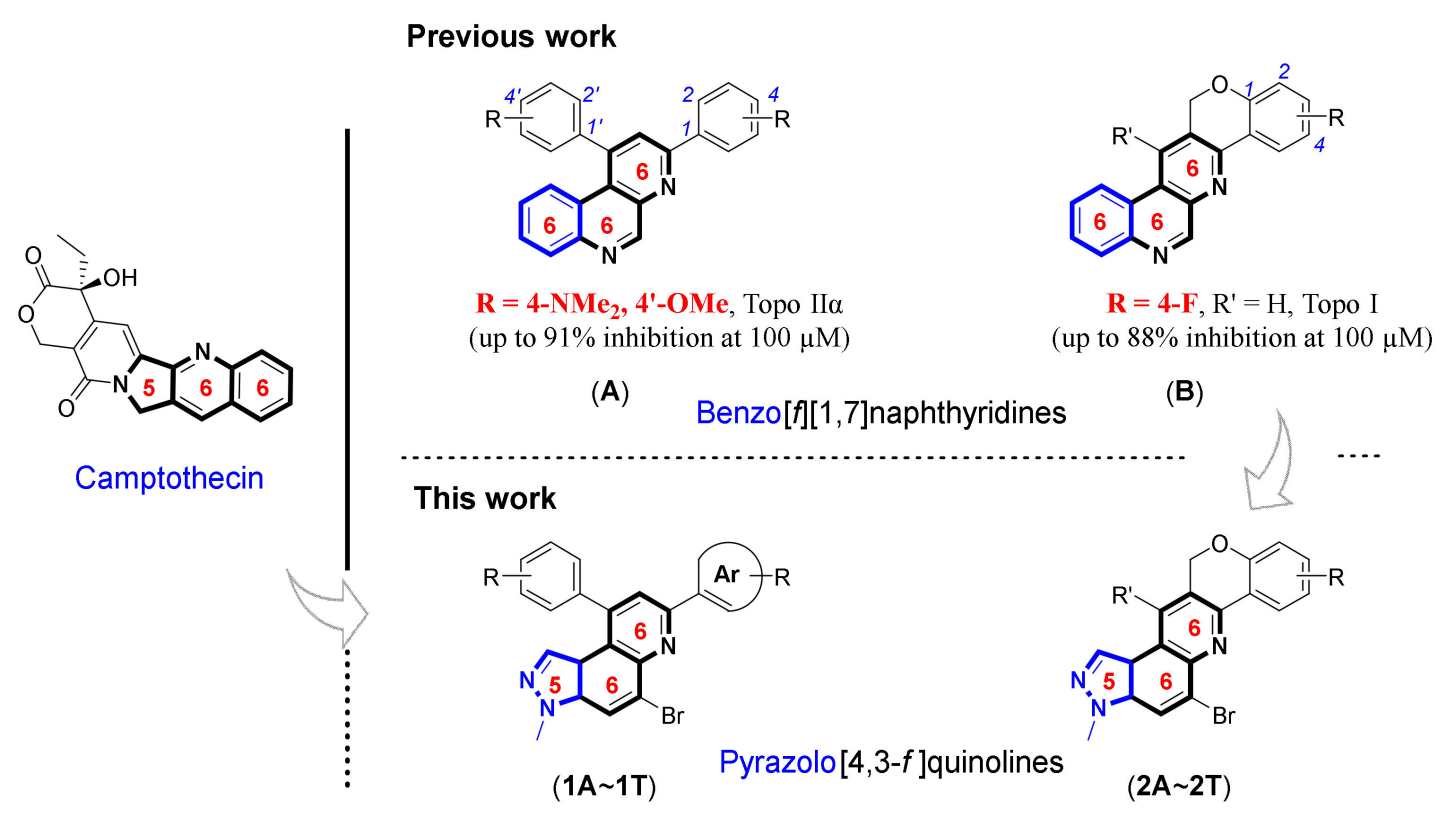
Representation of bioisostere-based research design.

In the current research, we show the design, synthesis, and cytotoxicity evaluation of pyrazolo[4,3-*f*]quinoline derivatives as potential anticancer agents (Figure 1). In addition, several studies reported that pyrazoloquinolines synergized in a single nucleus have been linked to a variety of biological actions, including anticancer, antiviral, antibacterial, antimalarial, anti-inflammatory, immunostimulant, and high corrosion inhibition activity [24,25,26,27]. Based on a bioisosterism strategy, this study aimed to generate a string of more active molecules by fusion of our 6,6,6-heterocyclic skeleton-similar previous work and 5,6,6-heterocyclic skeleton-similar anticancer medicines such as camptothecin (Figure 1). The strategy is an efficient and practical tool for developing novel compounds in which a functional group substitution in the lead molecule improves affinity, efficacy, druggability, and/or toxicity by modifying the binding affinity to the biological target [28,29].

## 2. Results

### 2.1. Chemistry

**Scheme 1:**
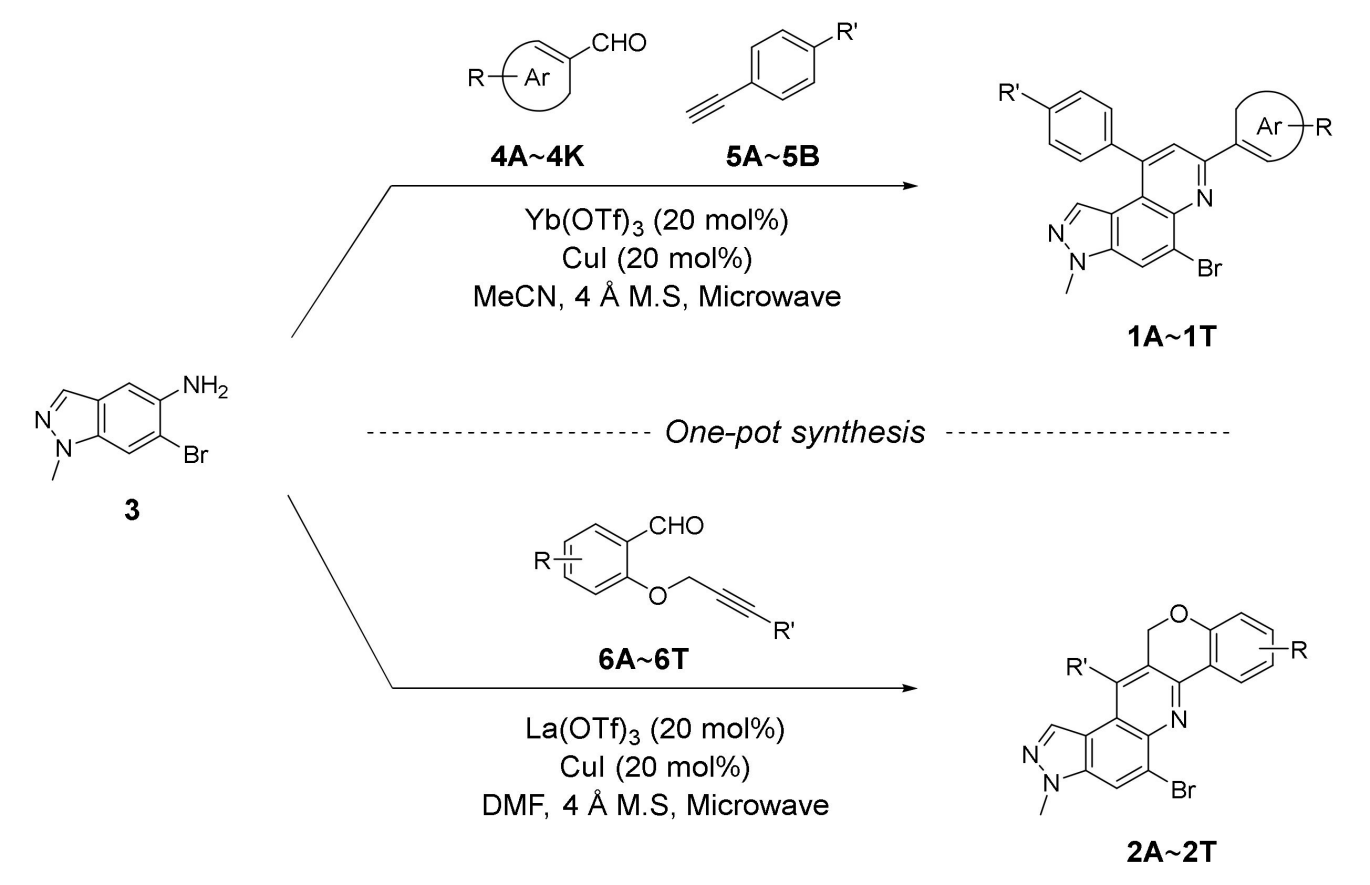
Synthesis of pyrazolo[4,3-*f*]quinolines.

As shown in Scheme 1, we utilized one-pot inter- or intramolecular synthetic methods to prepare pyrazolo[4,3-*f*]quinolines developed by us (i.e., synthesis of the compounds **A** and **B**). To replace the benzo[*f*][1,7]naphthyridine backbone (Figure 1, structures **A** and **B**) with pyrazolo[4,3-*f*]quinoline skeleton, we utilized 6-bromo-1-methyl-1*H*-indazol-5-amine (**3**) as a starting precursor. The precursor **3** in inverse imino Diels–Alder (DA) reaction mode was reacted either with the functionalized benzaldehydes **4A**~**4K** and salicylaldehydes **5A**~**5B** to achieve 5-bromo-3-methyl-7,9-diphenyl-3*H*-pyrazolo[4,3-*f*]quinolines **1Ai**~**1T** or with the 2-((3-phenylprop-2-yn-1-yl)oxy)benzaldehydes **6A**~**6T** to achieve 5-bromo-3-methyl-3,12-dihydrochromeno[4,3-*b*]pyrazolo[4,3-*f*]quinolines **2A**~**2T**. Besides, the starting precursors **3** and **6** were synthesized using literature procedures as shown in Schemes S1 and S4 (see Supplementary Materials) [30,31,32,33,34,35].

As depicted in Table 1, 20 new 7,9-diphenyl-3*H*-pyrazolo[4,3-*f*]quinoline derivatives **1** were prepared using the optimized condition developed previously [22]. The starting precursor **3** was condensed with **4A**~**4K** and **5A**~**5B** in intermolecular DA fashion in microwave condition. The microwave reactor progressed reactions more efficiently in a shorter time compared with conventional heating. The reaction yields for this transformation was low to moderate (Table 1, **1A**~**1T**, 16~45%) because the intermediate (i.e., imine) derived from **3** and **4** acts as a proton acceptor during the aromatization process of product **1** formation (Scheme S2, see Supplementary Materials) [36]. Roughly, the amount of intermediate reduced (i.e., imine to amine) was observed to be directly proportional to the reaction time; however, microwave condition turned advantageous to increase product yield slightly. Thus, the role of air oxidation was also considered as anticipated in our previous work [21]. To further occlude the imine reduction phenomenon, AgSbF_6_ was added as an external oxidizing source [37]. Although the formation of an amine was remarkably prevented, the solidification of the reaction mixture caused complications in the purification process, which lowered the yield. Therefore, the use of AgSbF_6_ was not adapted to the further reactions.

Likewise, the intramolecular inverse imino DA reactions were conducted between the precursors **3** and *O*-propargylated salicylaldehydes **6**. The reaction was initially conducted in the conditions developed by us [23] using the substrate **6B**; however, only a trace of the product **2B** was obtained (Table 2, entry 1). Therefore, various transition metal triflates as a Lewis acid catalyst, such as Yb(OTf)_3_, La(OTf)_3_, and Sc(OTf)_3_, along with solvents such as MeCN, xylene, toluene, and DMF, were screened in order to optimize the reaction condition, and the results were summarized in Table 2. Among several trials in different parameters, entry 9 was found to be the suitable condition, which yielded 43% of the desired product **2B** in 19 h reflux. Subsequently, the reaction was performed in a microwave reactor, which significantly reduced the reaction time and delivered 46% of the product **2B** within a 3.5 h interval. Then the condition was utilized in further derivatization as an optimized condition (Table 2, entry 14). As in intermolecular conditions, when AgSbF_6_ was used as an external oxidizing agent, solidification of the reaction mixture noticeably decreased reaction yields (entries 16–18) even though the reduction of imine intermediate was considerably avoided (Scheme S3, see Supplementary Materials).

Optimized condition (Table 2, entry 14) in hand, we utilized **3** as a starting precursor and coupled with the substituted *O*-propargylated salicylaldehydes **6A**~**6T** to prepare 20 new chromeno[4,3-*b*]pyrazolo[4,3-*f*]quinolines (yields: 23%~68%, Table 3, **2A**~**2T**). The yields in the intramolecular series were moderately improved over the intermolecular series. However, the improvement with respect to the amount of amine formed was not as expected. Despite the small amounts of amines (i.e., imine to amine) compared with that of the intermolecular reactions, benzofurans directly from *O*-propargylated salicylaldehydes as an additional side reaction might lead to low yields of the desired products **2**. The benzofuran was expected to generate as a result of hydration, followed by the intramolecular aldol condensation process (Scheme S5, See Supplementary Materials) [32].

### 2.2. Biological Results

The cytotoxicity of new compounds including 7,9-diphenyl-3*H*-pyrazolo[4,3-*f*]quinolines (**1A**~**1T**) and chromeno[4,3-*b*]pyrazolo[4,3-*f*]quinolines (**2A**~**2T**) at a fixed concentration (30 µM) was first screened against a gastric cancer cell line (NUGC-3) using a cell-based in vitro system (see Supplementary Materials for procedure) [38]. Most of the compounds showed a smaller extent of effects against NUGC-3 cancer cells, where more than 60% cell proliferation was observed compared with control sets. A few compounds, **1B**, **1C**, **2A**, **2D**, **2F**, and **2R**, were relatively better antiproliferative (less than 30% cell proliferation), and three compounds, **1M**, **2E**, and **2P**, were highly toxic against a tested cancer cell line (cells up to −30% less compared with an initial number of cells) at the experimental concentration (Table 4).

To clarify the cytotoxic effects, the compounds with a higher antiproliferative ability (less than 30% cell) were chosen for the follow-up antiproliferative evaluation in six different human cancer cell lines, namely, kidney cancer cell line (ACHN), colon cancer cell line (HCT-15), breast cancer cell line (MM231), lung cancer cell line (NCI-H23), gastric cancer cell line (NUGC-3), and prostate cancer cell line (PC-3). The antiproliferative ability of selected compounds was compared with doxorubicin (a positive control), and the data were expressed in 50% growth inhibition concentration (GI_50_) values (Table 5). Even though the tested compounds were less toxic to all human cancer cell lines compared with a positive control (GI_50_ less than 1 µM), growth inhibition (GI_50_) patterns were aligned with initial NUGC-3 cell screening results (Table 4). For example, the compounds **1M** (−29.01 ± 0.84%), **2E** (−17.83 ± 1.63%), and **2P** (−22.67 ± 4.55%) with negative NUGC-3 cell proliferation values demonstrated minimum GI_50_ concentration within entire cancer cell lines except for **1C** in MM231 (IG_50_ = 4.514 ± 0.170 µM) compared with **1M**, **2E**, and **2P**, and in PC-3 (IG_50_ = 6.068 ± 1.101 µM) compared with **1M**. It was proved that all compounds displayed significant inhibitions in six cancer cell lines at less than 14 µM concentrations. More specifically, the highly cytotoxic compounds **1M**, **2E**, and **2P** in NUGC-3 cells (Table 4) even inhibited 50% growth of all cancer cell lines below an 8 µM range concentration (Table 5). Therefore, the current pyrazolo[4,3-*f*]quinoline skeleton may serve as a potential source of anticancer agents upon further investigation.

**Figure 2:**
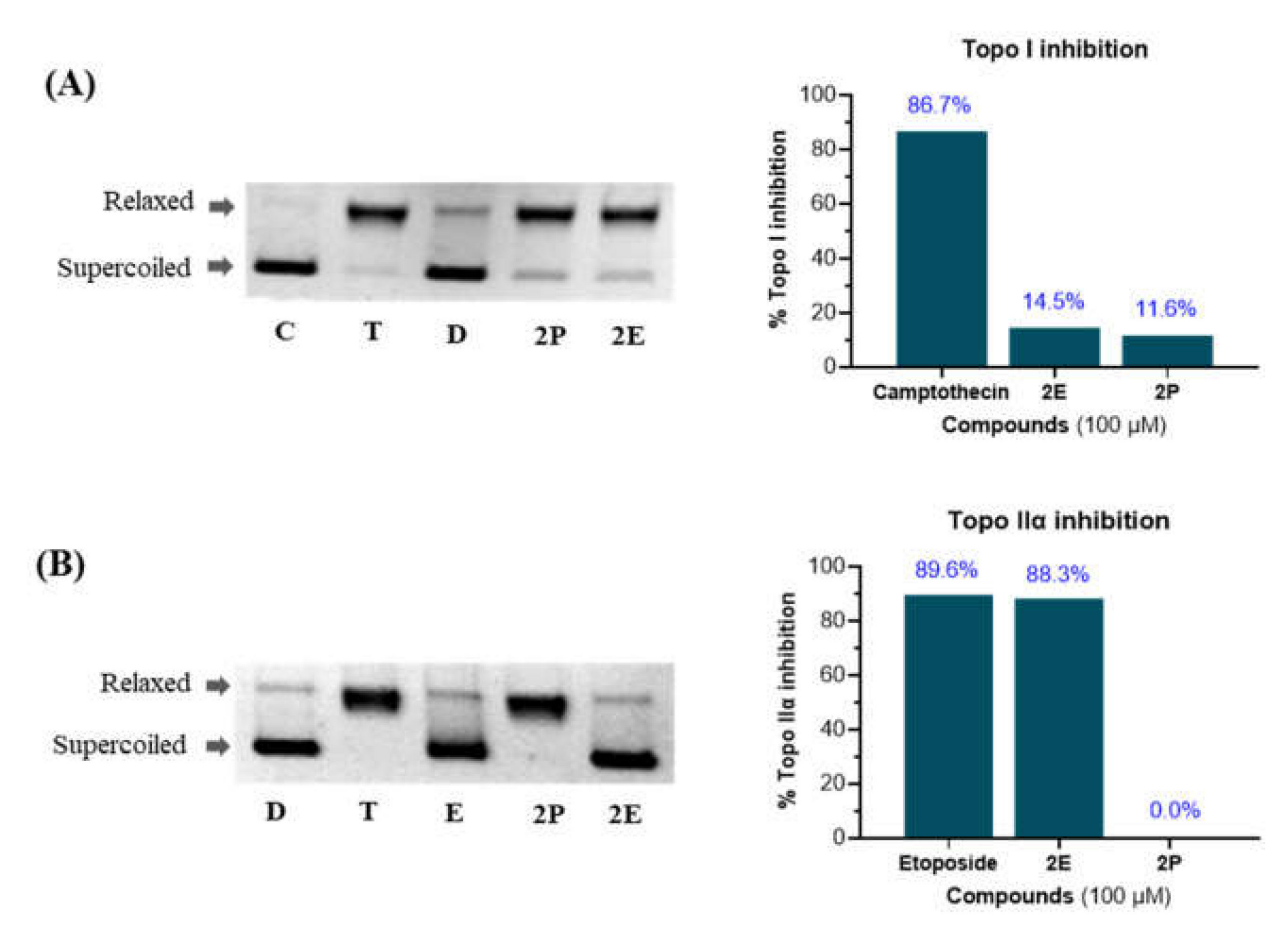
Human DNA topo I (**A**) and human DNA topo IIα (**B**) inhibitory activity. Lane D (pBR322 DNA), Lane T (pBR322 DNA + topo I or topo IIα), Lane C (pBR322 DNA + topo I + camptothecin), Lane E (pBR322 DNA + topo IIα + etoposide), Lane 2E (pBR322 DNA + topo I or topo IIα + compound **2E**), and Lane 2P (pBR322 DNA + topo I or topo IIα + compound **2P**).

To precisely access molecular targets for the lead compounds **2E** and **2P** (consistent GI_50_ values in six cancer cell lines, less than 7 µM), we examined their capacity to block the activity of topoisomerase enzymes in relaxing supercoiled plasmid DNA to its relaxed form (see Supplementary Materials for procedure) [39]. The camptothecin for topo I and the etoposide for topo IIα were taken as positive drug controls, and their relative topo inhibition patterns along with **2E** and **2P** were measured by Western blot analysis as demonstrated in Figure 2. Both the positive controls and compounds were used at a single-dose concentration (100 µM). As the results revealed, the compounds **2E** and **2P** were weakly effective in inhibiting topo I activity in respect to the camptothecin effect. The camptothecin blocked the catalytic activity of topo I in supercoiled DNA by 86.7%, where the activities were approximately 6 folds less for **2E** and 7 folds less for **2P** (Figure 2A).

Additionally, the compounds **2E** and **2P** were evaluated against topo IIα activity in comparison with etoposide. Interestingly, the compound **2E** was highly active in preventing topo IIα catalytic activity. It inhibited 88.3% of enzyme activity, which was similar to the activity of positive control (etoposide, 89.6% inhibition). However, the compound **2P** with marginal inhibition activity in topo I (11.6%) was inactive in the intercalation of the topo IIα enzyme (Figure 2B).

## 3. Discussion

The bioisosteric replacement is taken as a powerful tool in medicinal chemistry for improving druglike properties, toxicity, and pharmacokinetics of experimental therapeutics [40,41]. Utilizing the key concept of bioisosterism, the 6,6,6-heterocyclic skeleton of benzo[*f*][1,7]naphthyridines was replaced to the 5,6,6-heterocyclic skeleton of pyrazolo[4,3-*f*]quinolines (**1A**~**1T** and **2A**~**2T**) and successfully prepared 40 different derivatives via inverse imino DA condition. A receptor interaction of new compounds for anticancer activity was expected to be consistent with previously studied compounds during the design process [21,22]. The molecular docking simulations of best analogues of benzo[*f*][4,3-*b*][1,7]naphthyridines (i.e., 1,3-diphenylbenzo[*f*][1,7]naphthyridines (**A**) and 3*H*-benzo[*f*]chromeno[4,3-*b*][1,7]naphthyridines (**B**), Figure 1) inhibiting topo IIα activity showed good fit at the DNA cleavage site where etoposide was located. Moreover, the compound **B** intercalates into a single-strand cleavage site as well for topo I activity by van der Waals interactions with Arg364, Pro431, and Asn722 residues and π–π stacking interactions with the stacked DNA bases.

Unfortunately, most derivatives of the new series were less effective in antiproliferative activity against NUGC-3 cancer cells. We tried to figure out the effect of chemical properties of compounds in antiproliferative activity even though the data were acquired at a fixed dose of 30 µM concentration (Table 4). Initially, we thought of comparing substituents’ effect on ring B when R^1^ was methyl or methoxy on ring A in diphenyl-pyrazoloquinolines **1**. The electron-withdrawing nitrile substituent on the R^5^ position showed higher activity in both compounds **1C** (9.80% cell proliferation compared with drug untreated positive control) and **1M** (−29.01% cells). However, neither the R^5^ position, the compound **1G**, nor the electron-withdrawing property was consistent with the other compounds, **1D**, **1E**, **1N**, and **1O**. The effects of halogens, irrespective of their positions, were poorly effective against cancer cell proliferation (compounds **1F**~**1I**, **1Q**~**1S**). Similarly, very low effects were observed in the case of rings without substituents, the compounds **1A** and **1K**, or with electron-donating bulky substituents, the compounds **1J** and **1T**. The cytotoxic effect of pyridine heterocycle (ring B) was remarkably high when R^1^ was methyl (compound **1B**, 22.89% cell proliferation), but the effect was not restored when the methyl was replaced with the methoxy substituent (compound **1L**, 82.07% cell proliferation). Likewise, the thiophen-2-yl heterocycle **1P** was also not effective in the inhibition of cancer cell proliferation. Next, the structure–activity relationship (SAR) study was concentrated in chromeno-pyrazoloquinoline skeleton **2**. Six compounds most effectively inhibited NUGC-3 cell proliferation in this series. In the compounds **2A**, **2D**, **2E**, **2F**, **2P**, and **2R**, the cell proliferation was 19.41%, 27.68%, −17.83%, 14.67%, −22.67%, and 11.16%, respectively, compared with the positive control set. Unfortunately, as in the diphenyl-pyrazoloquinolines **1** series, the structure–activity relationship was not systematically established in chromeno-pyrazoloquinolines **2** too. Briefly, compounds having electron-releasing substituents (compounds **2B**, **2C**, and **2S**) on ring B’ were weakly active. Conversely, the methyl at the R^2′^ position (**2D**, 27.68% growth) was slightly active in antiproliferative activity. We analyzed the effects of halogen substituents, and –Br at R^2′^ (**2P**) and –F at R^6′^ (**2E**) were highly toxic to NUGC-3 cancer cells. At the same time, –F or –Cl at R^2′^ and –Cl at the R^6′^ (**2H**, **2L**, and **2I**, respectively) positions did not show a notable effect on cell proliferation inhibition. Halogens at R^3′^ or R^4′^ (**2G**, **2J**, **2K**, **2N**, and **2O**) were also poorly active except for –F at the R^4′^ position (**2F**, showing 14.67% cell proliferation), which showed no evidence of linear correlation of activity to halogen atomic size, electronegativity, and their substituted positions. Further, the naphthalenes (compounds **2Q**, **2R,** and **2T**) instead of ring B’ with a different orientation were evaluated, where **2R** displayed higher activity out of three different naphthalene compounds. The activity of compound **2R** was similar to that of compound **2F**, whose relation is indescribable with respect to their structures and chemical properties.

Eight effective analogues, **1B**, **1C**, **1M**, **2A**, **2D**, **2E**, **2F**, **2P**, and **2R**, were tested in six different human cancer cell lines for antitumorigenic ability, where only two chromeno analogues, **2E** and **2P**, were selected for follow-up evaluation (Table 5 and Figure 2). Both were less effective in blocking the catalytic activity of topo I enzyme activity. Interestingly, **2E** showed strong inhibition of topo IIα activity in DNA religation.

To this end, it was found that **2M** of the diphenyl-pyrazolo[4,3-*f*]quinolines **1** and **2E** and **2P** of chromeno-pyrazolo[4,3-*f*]quinolines **2** showed high cytotoxicity regardless of structural relation to other analogues. Therefore, further experiments are essential to better understand the SAR relationship and mode of antiproliferative action to develop pyrazolo[4,3-*f*]quinoline scaffold as a potential source of anticancer drug candidates.

## 4. Materials and Methods

### 4.1. General Information

Unless noted otherwise, all reagents were purchased from commercial sources (Aldrich, TCI, Alfa Aesar, and Acros) and used as received. Air or moisture labile reactions were conducted in oven-dried glassware under a nitrogen atmosphere. Reaction progress was monitored by thin-layer chromatography (TLC) using silica gel F_254_ plates. Products were purified by flash column chromatography using silica gel 60 (70–230 mesh) of Kieselgel^60^ (Merck, KGaA, 64271 Darmstadt, Germany) or by using the Biotage ‘Isolera One’ system with indicated solvents. High-resolution mass spectrometry was performed with LCQ Fleet, Thermo Scientific (Waltham, MA, USA), recorded in positive ion mode with an electrospray ionization (ESI) source. NMR spectra were recorded on a Jeol Resonance ECZ 400S (400 MHz for ^1^H NMR and 100 MHz for ^13^C NMR). Chemical shifts were reported in ppm from tetramethylsilane (TMS) with the solvent resonance resulting from incomplete deuteration as the internal reference (CDCl_3_: 7.26 ppm, CD_3_OD: 3.31 ppm, DMSO: 2.5 ppm, 3.33 ppm of water peak) or relative to TMS (*δ* 0.0). Data are reported as follows: chemical shift *δ*, multiplicity (s = singlet, d = doublet, t = triplet, m = multiplet, dd = doublet of doublet, td = triplet of doublet, ddd = doublet of doublets of doublets, ddt = doublet of doublet of triplets), coupling constants (Hz), number of protons.

### 4.2. General Procedure for the Synthesis of Diphenyl-pyrazolo[4,3-f]quinolines 1

The starting precursor **3** (98 mg, 0.44 mmol), intermediates **4** (0.44 mmol), **5** (0.44 mmol), Yb(OTf)_3_ (30 mol%), and CuI (20 mol%) in DMF (1 mL) with a molecular sieve was reacted under microwave conditions (175 °C) for 1.5 h. The reaction mixture was diluted with DCM and water, then extracted with DCM. Combined organic layers were dried over MgSO_4_, filtered, and concentrated. The concentrated crude was purified using silica gel column chromatography (Hex/EtOAc = 5:1) to afford the desired products **1** as solids.

5-Bromo-3-methyl-7-phenyl-9-(*p*-tolyl)-3*H*-pyrazolo[4,3-*f*]quinoline (**1A**)

White solid; 30% yield; *R_f_* 0.6 (Hex/EtOAc = 2:1); ^1^H NMR (400 MHz, CDCl_3_): *δ* 8.34 (d, *J* = 7.2 Hz, 2H), 8.18 (d, *J* = 0.8 Hz, 1H), 7.94 (s, 1H), 7.54–7.52 (m, 2H), 7.46 (tt, *J* = 7.2, 2.4, 1.2 Hz, 1H), 7.39 (d, *J* = 7.6 Hz, 2H), 7.36 (dd, *J* = 8.0, 2.0 Hz, 2H), 6.86 (d, *J* = 0.8 Hz, 1H), 4.09 (s, 3H), 2.52 (s, 3H); ^13^C NMR (100 MHz, CDCl_3_): *δ* 154.42, 148.48, 142.70, 138.97, 138.95, 137.78, 137.15, 134.63, 129.96, 129.61, 129.05, 128.59, 127.50, 126.38, 121.57, 120.95, 117.95, 117.46, 36.05, 21.58; HRMS *m/z* [M+H]^+^ calculated for C_24_H_19_BrN_3_^+^: 428.0757; found: 428.0761.

5-Bromo-3-methyl-7-(pyridin-3-yl)-9-(*p*-tolyl)-3*H*-pyrazolo[4,3-*f*]quinoline (**1B**)

White solid; 17% yield; *R_f_* 0.2 (Hex/EtOAc = 1:1); ^1^H NMR (400 MHz, CDCl_3_): *δ* 9.49 (s, 1H), 8.73–8.64 (m, 2H), 8.20 (d, *J* = 0.9 Hz, 1H), 7.94 (s, 1H), 7.48 (dd, *J* = 7.8, 4.8 Hz, 1H), 7.40 (d, *J* = 7.9 Hz, 2H), 7.38–7.34 (m, 2H), 6.88 (d, *J* = 0.9 Hz, 1H), 4.10 (s, 3H), 2.53 (s, 3H); ^13^C NMR (100 MHz, CDCl_3_): *δ* 151.75, 150.41, 148.86, 148.82, 142.86, 139.16, 137.87, 136.84, 134.93, 134.90, 134.55, 130.03, 128.53, 125.94, 123.92, 122.02, 120.61, 117.87, 36.11, 21.58; HRMS *m/z* [M+H]^+^ calculated for C_23_H_18_BrN_4_^+^: 429.0709; found: 429.0711.

3-(5-Bromo-3-methyl-9-(*p*-tolyl)-3*H*-pyrazolo[4,3-*f*]quinolin-7-yl)benzonitrile (**1C**)

Yellow solid; 27% yield; *R_f_* 0.5 (Hex/EtOAc = 2:1); ^1^H NMR (400 MHz, CDCl_3_): *δ* 8.65 (s, 1H), 8.54 (d, *J* = 7.9 Hz, 1H), 8.20 (s, 1H), 7.90 (s, 1H), 7.72 (d, *J* = 7.6 Hz, 1H), 7.63 (t, *J* = 7.8 Hz, 1H), 7.39 (d, *J* = 8.0 Hz, 2H), 7.34 (d, *J* = 7.9 Hz, 2H), 6.87 (s, 1H), 4.09 (s, 3H), 2.51 (s, 3H); ^13^C NMR (100 MHz, CDCl_3_): *δ* 151.72, 149.08, 142.78, 140.17, 139.26, 137.95, 136.72, 134.85, 132.72, 131.20, 130.07, 129.85, 128.49, 126.06, 122.20, 120.57, 119.03, 118.01, 117.79, 113.32, 36.11, 21.59; HRMS *m/z* [M+H]^+^ calculated for C_25_H_18_BrN_4_^+^: 453.0709; found: 453.0713.

5-Bromo-3-methyl-9-(*p*-tolyl)-7-(4-(trifluoromethyl)phenyl)-3*H*-pyrazolo[4,3-*f*]quinoline (**1D**)

Yellow solid; 29% yield; *R_f_* 0.5 (Hex/EtOAc = 4:1); ^1^H NMR (400 MHz, CDCl_3_): *δ* 8.45 (d, *J* = 8.8 Hz, 2H), 8.20 (s, 1H), 7.95 (s, 1H), 7.79 (d, *J* = 8.8 Hz, 2H), 7.40 (d, *J* = 8.2 Hz, 2H), 7.36 (d, *J* = 8.2 Hz, 2H), 6.88 (s, 1H), 4.10 (s, 3H), 2.53 (s, 3H); ^13^C NMR (100 MHz, CDCl_3_): *δ* 152.64, 148.79, 142.73, 142.29, 139.13, 137.88, 136.88, 134.89, 128.53, 127.72, 126.04, 125.94, 122.07, 120.93, 117.84, 36.11, 21.59; HRMS *m/z* [M+H]^+^ calculated for C_25_H_18_BrF_3_N_3_^+^: 496.0631; found: 496.0635.

5-Bromo-3-methyl-7-(4-nitrophenyl)-9-(*p*-tolyl)-3*H*-pyrazolo[4,3-*f*]quinoline (**1E**)

Yellow solid; 45% yield; *R_f_* 0.4 (Hex/EtOAc = 2:1); ^1^H NMR (400 MHz, CDCl_3_): *δ* 8.52 (dt, *J* = 9.5, 2.4 Hz, 2H), 8.39 (dt, *J* = 8.8, 2.4 Hz, 2H), 8.22 (d, *J* = 0.8 Hz, 1H), 7.99 (s, 1H), 7.41 (d, *J* = 7.6 Hz, 2H), 7.36 (dd, *J* = 6.4, 2.0 Hz, 2H), 6.90 (s, 1H), 4.11 (s, 3H), 2.53 (s, 3H); ^13^C NMR (100 MHz, CDCl_3_): *δ* 151.49, 149.02, 148.48, 144.87, 142.84, 139.29, 138.02, 136.70, 135.04, 130.09, 128.50, 128.17, 126.00, 124.29, 122.42, 121.11, 118.15, 117.79, 36.16, 21.60; HRMS *m/z* [M+H]^+^ calculated for C_24_H_18_BrN_4_O_2_^+^: 473.0608; found: 473.0611.

5-Bromo-7-(2-chlorophenyl)-3-methyl-9-(*p*-tolyl)-3*H*-pyrazolo[4,3-*f*]quinoline (**1F**)

White solid; 36% yield; *R_f_* 0.5 (Hex/EtOAc = 2:1); ^1^H NMR (400 MHz, CDCl_3_): *δ* 8.18 (s, 1H), 7.97 (dd, *J* = 7.6, 1.6 Hz, 1H), 7.93 (s, 1H), 7.51 (dd, *J* = 8.0, 1.6 Hz, 1H), 7.97 (dt, *J* = 7.6, 1.6 Hz, 1H), 7.40–7.35 (m, 5H), 6.92 (s, 1H), 4.10 (s, 3H), 2.51 (s, 3H); ^13^C NMR (100 MHz, CDCl_3_): *δ* 154.27, 147.24, 142.77, 138.93, 137.90, 136.98, 134.99, 132.80, 132.65, 130.50, 130.03, 129.93, 128.67, 127.40, 125.82, 125.41, 121.65, 117.93, 117.45, 36.09, 21.57; HRMS *m/z* [M+H]^+^ calculated for C_24_H_18_BrClN_3_^+^: 462.0367; found: 462.0376.

5-Bromo-7-(3-chlorophenyl)-3-methyl-9-(*p*-tolyl)-3*H*-pyrazolo[4,3-*f*]quinoline (**1G**)

Pale brown solid; 27% yield; *R_f_* 0.6 (Hex/EtOAc = 2:1); ^1^H NMR (400 MHz, CDCl_3_): *δ* 8.33 (t, *J* = 1.6, 1H), 8.22–8.19 (m, 2H), 7.9 (s, 1H), 7.46–7.34 (m, 6H), 6.87 (d, *J* = 0.8 Hz, 1H), 4.10 (s, 3H), 2.52 (s, 3H); ^13^C NMR (100 MHz, CDCl_3_): *δ* 152.75, 148.69, 142.66, 140.80, 139.06, 137.84, 136.97, 135.13, 134.84, 130.25, 129.97, 129.49, 128.55, 127.59, 126.04, 125.51, 121.90, 120.77, 117.90, 117.71, 36.08, 21.57; HRMS *m/z* [M+H]+ calculated for C_24_H_18_BrClN_3_^+^: 462.0367; found: 462.0373.

5-Bromo-7-(4-chlorophenyl)-3-methyl-9-(*p*-tolyl)-3*H*-pyrazolo[4,3-*f*]quinoline (**1H**)

Red solid; 22% yield; *R_f_* 0.6 (Hex/EtOAc = 4:1); ^1^H NMR (400 MHz, CDCl_3_): *δ* 8.28 (d, *J* = 8.7 Hz, 2H), 8.18 (s, 1H), 7.89 (s, 1H), 7.50 (d, *J* = 8.7 Hz, 2H), 7.39 (d, *J* = 8.2 Hz, 2H), 7.34 (d, *J* = 8.2 Hz, 2H), 6.86 (s, 1H), 4.10 (s, 3H), 2.52 (s, 3H); ^13^C NMR (100 MHz, CDCl_3_): *δ* 153.02, 148.60, 142.62, 139.01, 137.77, 137.41, 137.00, 135.69, 134.78, 129.96, 129.58, 129.20, 128.69, 128.54, 127.28, 126.00, 121.67, 120.55, 117.91, 117.64, 36.07, 21.57; HRMS *m/z* [M+H]^+^ calculated for C_24_H_18_BrClN_3_^+^: 462.0367; found: 462.0374.

5-Bromo-7-(4-bromophenyl)-3-methyl-9-(*p*-tolyl)-3*H*-pyrazolo[4,3-*f*]quinoline (**1I**)

Pale brown solid; 21% yield; *R_f_* 0.6 (Hex/EtOAc = 2:1); ^1^H NMR (400 MHz, CDCl_3_): *δ* 8.21 (d, *J* = 8.8 Hz, 2H), 8.18 (d, *J* = 0.8 Hz, 1H), 7.90 (s, 1H), 7.65 (d, *J* = 8.8 Hz, 2H), 7.39 (d, *J* = 8.0 Hz, 2H), 7.34 (d, *J* = 8.0 Hz, 2H), 6.88 (d, *J* = 1.2 Hz, 1H), 4.11 (s, 3H), 2.52 (s, 3H); ^13^C NMR (100 MHz, CDCl_3_): *δ* 153.09, 148.65, 142.65, 139.04, 137.90, 137.80, 137.00, 134.80, 132.17, 129.97, 128.99, 128.55, 126.01, 124.12, 121.74, 120.52, 117.94, 117.98, 36.09, 21.58; HRMS *m/z* [M+H]^+^ calculated for C_24_H_18_Br_2_N_3_^+^: 505.9862; found: 505.9872.

4-(5-Bromo-3-methyl-9-(*p*-tolyl)-3*H*-pyrazolo[4,3-*f*]quinolin-7-yl)-N,N-dimethylaniline (**1J**)

Orange solid; 16% yield; *R_f_* 0.5 (Hex/EtOAc = 2:1); ^1^H NMR (400 MHz, DMSO): *δ* 8.58 (s, 1H), 8.26 (d, *J* = 8.8 Hz, 2H), 7.96 (s, 1H), 7.44 (d, *J* = 8.0 Hz, 2H), 7.38 (d, *J* = 8.0 Hz, 2H), 6.86 (d, *J* = 8.8 Hz, 1H), 6.57 (s, 1H), 4.07 (s, 3H), 3.00 (s, 6H), 2.48 (s, 3H); ^13^C NMR (100 MHz, DMSO): *δ* 153.72, 151.22, 147.68, 141.67, 138.36, 137.18, 136.93, 133.01, 129.68, 128.52, 128.13, 125.75, 124.60, 119.72, 119.22, 118.36, 117.16, 112.28, 40.07, 35.95, 21.05; HRMS *m/z* [M+H]^+^ calculated for C_26_H_24_BrN_4_^+^: 471.1179; found: 471.1187.

5-Bromo-9-(4-methoxyphenyl)-3-methyl-7-phenyl-3*H*-pyrazolo[4,3-*f*]quinoline (**1K**)

Brown solid; 12% yield; *R_f_* 0.4 (Hex/EtOAc = 2:1); ^1^H NMR (400 MHz, CDCl_3_): *δ* 8.68 (dd, *J* = 8.4, 8.4 Hz, 2H), 8.18 (s, 1H), 7.94 (s, 1H), 7.56–7.52 (m, 2H), 7.48–7.44 (m, 1H), 7.39 (d, *J* = 8.8 Hz, 2H), 7.11 (d, *J* = 8.8 Hz, 2H), 6.95 (s, 1H), 4.11 (s, 3H), 3.94 (s, 3H); ^13^C NMR (100 MHz, CDCl_3_): *δ* 160.23, 154.33, 148.12, 142.70, 138.96, 137.77, 134.72, 132.32, 129.99, 129.56, 129.03, 127.46, 126.18, 121.73, 121.11, 117.98, 117.45, 114.65, 55.59, 36.07, 31.72, 31.10, 29.85, 22.79, 14.27; HRMS *m/z* [M+H]^+^ calculated for C_24_H_19_BrN_3_O^+^: 444.0706; found: 444.0706.

5-Bromo-9-(4-methoxyphenyl)-3-methyl-7-(pyridin-3-yl)-3*H*-pyrazolo[4,3-*f*]quinoline (**1L**)

Orange solid; 33% yield; *R_f_* 0.3 (Hex/EtOAc = 2:1); ^1^H NMR (400 MHz, CDCl_3_): *δ* 9.49 (s, 1H), 8.68 (tt, *J* = 3.9, 1.8 Hz, 2H), 8.19 (s, 1H), 7.93 (s, 1H), 7.47 (dd, *J* = 7.9, 4.8 Hz, 1H), 7.39 (d, *J* = 8.7 Hz, 2H), 7.11 (d, *J* = 8.7 Hz, 2H), 6.95 (s, 1H), 4.10 (s, 3H), 3.93 (s, 3H); ^13^C NMR (100 MHz, CDCl_3_): *δ* 160.38, 151.74, 150.41, 148.82, 148.56, 142.90, 137.87, 134.91, 134.86, 134.53, 131.95, 129.95, 125.95, 123.90, 122.18, 120.81, 117.85, 114.76, 55.61, 36.11; HRMS *m/z* [M+H]^+^ calculated for C_23_H_18_BrN_4_O^+^: 445.0659; found: 445.0667.

3-(5-Bromo-9-(4-methoxyphenyl)-3-methyl-3*H*-pyrazolo[4,3-*f*]quinolin-7-yl)benzonitrile (**1M**)

Yellow solid; 26% yield; *R_f_* 0.4 (Hex/EtOAc = 2:1); ^1^H NMR (400 MHz, CDCl_3_): *δ* 8.65 (s, 1H), 8.56 (dt, *J* = 8.4, 1.6 Hz, 1H), 8.21 (s, 1H), 7.81 (s, 1H), 7.73 (dt, *J* = 8.0, 1.6 Hz, 1H), 7.64 (t, *J* = 7.6, 1H), 7.39 (dt, *J* = 8.8, 2.4 Hz, 2H), 7.12 (dt, *J* = 8.4, 2.4 Hz, 2H), 6.96 (s, 1H), 4.12 (s, 3H), 3.95 (s, 3H); ^13^C NMR (100 MHz, CDCl_3_): *δ* 160.46, 151.78, 148.80, 142.86, 140.15, 137.97, 134.70, 132.73, 131.79, 131.55, 131.19, 129.90, 129.85, 126.85, 122.35, 120.81, 119.04, 117.97, 117.77, 114.83, 113.31, 55.64, 36.11; HRMS *m/z* [M+H]^+^ calculated for C_25_H_18_BrN_4_O^+^: 469.0659; found: 469.0663.

5-Bromo-9-(4-methoxyphenyl)-3-methyl-7-(4-(trifluoromethyl)phenyl)-3*H*-pyrazolo[4,3-*f*]quinoline (**1N**)

Red solid; 8% yield; *R_f_* 0.4 (Hex/EtOAc = 3:1); ^1^H NMR (400 MHz, CDCl_3_): *δ* 8.44 (d, *J* = 8.3 Hz, 2H), 8.19 (s, 1H), 7.94 (s, 1H), 7.78 (d, *J* = 8.3 Hz, 2H), 7.39 (d, *J* = 8.7 Hz, 2H), 7.11 (d, *J* = 8.7 Hz, 2H), 6.95 (s, 1H), 4.10 (s, 3H), 3.93 (s, 3H); ^13^C NMR (100 MHz, CDCl_3_): *δ* 160.37, 152.64, 148.49, 142.79, 142.30, 137.91, 134.86, 132.02, 131.37, 131.05, 129.95, 127.72, 126.06, 125.96, 125.93, 125.71, 122.24, 117.83, 114.75, 55.61, 36.11; HRMS *m/z* [M+H]^+^ calculated for C_25_H_18_BrF_3_N_3_O^+^: 512.0580; found: 512.0581.

5-Bromo-9-(4-methoxyphenyl)-3-methyl-7-(4-nitrophenyl)-3*H*-pyrazolo[4,3-*f*]quinoline (**1O**)

Yellow solid; 20% yield; *R_f_* 0.5 (Hex/EtOAc = 2:1); ^1^H NMR (400 MHz, CDCl_3_): *δ* 8.51 (d, *J* = 9.2 Hz, 2H), 8.22 (d, *J* = 0.8 Hz, 1H), 7.98 (s, 1H), 7.40 (d, *J* = 8.8 Hz, 2H), 7.12 (d, *J* = 8.8 Hz, 2H), 7.11 (d, *J* = 8.7 Hz, 2H), 6.97 (d, *J* = 0.8 Hz, 1H), 4.12 (s, 3H), 3.95 (s, 3H); ^13^C NMR (100 MHz, CDCl_3_): *δ* 160.48, 151.52, 148.74, 148.48, 144.86, 142.92, 138.05, 134.98, 131.80, 129.92, 128.17, 126.08, 124.28, 122.59, 121.34, 118.13, 117.80, 114.83, 55.65, 36.15; HRMS *m/z* [M+H]^+^ calculated for C_24_H_19_BrN_4_O_3_^+^: 489.0557; found: 489.0560.

5-Bromo-9-(4-methoxyphenyl)-3-methyl-7-(thiophen-2-yl)-3*H*-pyrazolo[4,3-*f*]quinoline (**1P**)

Brown solid; 35% yield; *R_f_* 0.3 (Hex/EtOAc = 4:1); ^1^H NMR (400 MHz, CDCl_3_): *δ* 8.15 (s, 1H), 8.12 (d, *J* = 3.0 Hz, 1H), 7.96 (d, *J* = 5.0 Hz, 1H), 7.77 (s, 1H), 7.43 (dd, *J* = 5.0, 3.0 Hz, 1H), 7.37 (d, *J* = 8.7 Hz, 2H), 7.09 (d, *J* = 8.7 Hz, 2H), 6.90 (s, 1H), 4.08 (s, 3H), 3.93 (s, 3H); ^13^C NMR (100 MHz, CDCl_3_): *δ* 160.25, 150.80, 148.06, 142.64, 142.39, 137.70, 134.60, 132.21, 129.97, 126.91, 126.54, 125.86, 124.58, 121.51, 121.29, 118.06, 117.45, 114.66, 55.60, 36.06; HRMS *m/z* [M+H]^+^ calculated for C_22_H_17_BrN_3_OS^+^: 450.0270; found: 450.0271.

5-Bromo-7-(2-chlorophenyl)-9-(4-methoxyphenyl)-3-methyl-3*H*-pyrazolo[4,3-*f*]quinoline (**1Q**)

White solid; 38% yield; *R_f_* 0.4 (Hex/EtOAc = 2:1); ^1^H NMR (400 MHz, CDCl_3_): *δ* 8.19 (d, *J* = 1.2 Hz, 1H), 7.97 (dd, *J* = 7.6, 1.6 Hz, 1H), 7.93 (s, 1H), 7.51 (dd, *J* = 8.0, 1.6 Hz, 1H), 7.47–7.36 (m, 4H), 7.10 (dt, *J* = 8.8, 2.8 Hz, 2H), 7.00 (d, *J* = 1.20 Hz, 1H), 4.11 (s, 3H), 3.93 (s, 3H); ^13^C NMR (100 MHz, CDCl_3_): *δ* 160.24, 154.30, 146.94, 142.80, 138.80, 137.86, 134.69, 132.72, 132.58, 131.97, 130.44, 129.99, 127.36, 126.07, 125.64, 121.73, 117.80, 117.35, 114.65, 55.53, 36.01; HRMS *m/z* [M+H]^+^ calculated for C_24_H_18_BrClN_3_O^+^: 478.0316; found: 478.0321.

5-Bromo-7-(4-chlorophenyl)-9-(4-methoxyphenyl)-3-methyl-3*H*-pyrazolo[4,3-*f*]quinoline (**1R**)

Yellow solid; 23% yield; *R_f_* 0.4 (Hex/EtOAc = 4:1); ^1^H NMR (400 MHz, CDCl_3_): *δ* 8.27 (d, *J* = 8.5 Hz, 2H), 8.17 (s, 1H), 7.88 (s, 1H), 7.49 (d, *J* = 8.5 Hz, 2H), 7.38 (d, *J* = 8.5 Hz, 2H), 7.10 (d, *J* = 8.5 Hz, 2H), 6.93 (s, 1H), 4.09 (s, 3H), 3.93 (s, 3H); ^13^C NMR (100 MHz, CDCl_3_): *δ* 160.32, 153.06, 148.34, 142.71, 137.82, 137.45, 135.71, 134.78, 132.17, 129.97, 129.21, 128.72, 126.05, 121.87, 120.79, 117.95, 117.65, 114.71, 55.61, 36.08; HRMS *m/z* [M+H]^+^ calculated for C_24_H_18_BrClN_3_O^+^: 478.0316; found: 478.0320.

5-Bromo-7-(4-bromophenyl)-9-(4-methoxyphenyl)-3-methyl-3*H*-pyrazolo[4,3-*f*]quinoline (**1S**)

Brown solid; 20% yield; *R_f_* 0.3 (Hex/EtOAc = 4:1); ^1^H NMR (400 MHz, CDCl_3_): *δ* 8.21 (d, *J* = 8.6 Hz, 2H), 8.17 (s, 1H), 7.88 (s, 1H), 7.65 (d, *J* = 8.6 Hz, 2H), 7.38 (d, *J* = 8.7 Hz, 2H), 7.10 (d, *J* = 8.7 Hz, 2H), 6.93 (s, 1H), 4.09 (s, 3H), 3.93 (s, 3H); ^13^C NMR (100 MHz, CDCl_3_): *δ* 160.30, 153.09, 148.35, 142.70, 137.89, 137.81, 134.77, 132.16, 129.96, 129.00, 126.03, 124.11, 121.91, 120.74, 117.94, 117.66, 114.70, 55.61, 36.09; HRMS *m/z* [M+H]^+^ calculated for C_24_H_18_Br_2_N_3_O^+^: 521.9811; found: 521.9818.

4-(5-Bromo-9-(4-methoxyphenyl)-3-methyl-3*H*-pyrazolo[4,3-*f*]quinolin-7-yl)-*N,N*-dimethylaniline (**1T**)

Orange solid; 40% yield; *R_f_* 0.4 (Hex/EtOAc = 2:1); ^1^H NMR (400 MHz, CDCl_3_): *δ* 8.25 (d, *J* = 8.8 Hz, 2H), 8.13 (s, 1H), 7.84 (s, 1H), 7.39 (d, *J* = 8.8 Hz, 2H), 7.10 (d, *J* = 8.8 Hz, 2H), 6.88 (s, 1H), 6.84 (d, *J* = 8.8 Hz, 2H), 4.08 (s, 3H), 3.94 (s, 3H), 3.05 (s, 6H); ^13^C NMR (100 MHz, CDCl_3_): *δ* 160.11, 154.70, 151.53, 147.62, 142.68, 137.51, 134.47, 132.71, 130.05, 128.41, 126.87, 126.20, 120.76, 120.18, 118.22, 117.02, 114.57, 112.40, 55.60, 40.52, 36.02; HRMS *m/z* [M+H]^+^ calculated for C_26_H_24_BrN_4_O^+^: 487.1128; found: 487.1132.

### 4.3. General Procedure for the Synthesis of Chromeno-pyrazolo[4,3-f]quinolines 2

The starting precursor **3** (98 mg, 0.44 mmol), substituted intermediates **6** (0.44 mmol), Yb(OTf)_3_ (30 mol%), and CuI (20 mol%) in DMF (1 mL) with a molecular sieve, was reacted under microwave conditions (175 °C) for 3.5 h. The reaction mixture was diluted with DCM and water, then extracted with DCM. Combined organic layers were dried over MgSO_4_, filtered, and concentrated. The concentrated crude was purified using silica gel column chromatography (Hex/EtOAc = 5:1) to afford the desired products **2** as solids.

5-Bromo-3-methyl-3,12-dihydrochromeno[4,3-*b*]pyrazolo[4,3-*f* ]quinoline (**2A**)

Deep yellow solid; 54% yield; *R_f_* 0.5 (Hex/EtOAc = 1:1); ^1^H NMR (CDCl_3_, 400 MHz): *δ* 8.54 (dd, *J* = 7.6, 2.0 Hz, 1H), 8.30 (s, 1H), 8.17 (s, 1H), 8.09 (s, 1H), 7.37 (dd, *J* = 7.6, 2.0 Hz, 1H), 7.18 (dd, *J* = 7.6, 0.8 Hz, 1H), 7.01 (dd, *J* = 7.6, 0.8 Hz, 1H), 5.44 (s, 2H), 4.14 (s, 3H); ^13^C NMR (100 MHz, CDCl_3_): *δ* 157.05, 147.46, 142.00, 137.42, 131.94, 126.74, 126.69, 125.67, 125.33, 123.21, 122.82, 122.03, 118.71, 117.29, 117.09, 68.23, 36.23; HRMS *m/z* [M+H]^+^ calculated for C_18_H_13_BrN_3_O^+^: 366.0237; found: 366.0235.

5-Bromo-9-methoxy-3-methyl-3,12-dihydrochromeno[4,3-*b*]pyrazolo[4,3-*f*]quinoline (**2B**)

Orange brown solid; 46% yield; *R_f_* 0.5 (Hex/EtOAc = 1:1); ^1^H NMR (CDCl_3_, 400 MHz): *δ* 8.45 (d, *J* = 8.4 Hz, 1H), 8.29 (s, 1H), 8.14 (s, 1H), 8.08 (s, 1H), 6.75 (dd, *J* = 8.4, 2.4 Hz, 1H), 6.53 (d, *J* = 2.4 Hz, 1H), 5.42 (s, 2H), 4.15 (s, 3H), 3.86 (s, 3H); ^13^C NMR (100 MHz, CDCl_3_): *δ* 163.08, 158.50, 147.75, 142.01, 137.29, 131.79, 126.90, 126.60, 125.80, 125.17, 121.48, 118.86, 116.94, 116.31, 109.95, 101.82, 68.54, 55.65, 36.22; HRMS *m/z* [M+H]^+^ calculated for C_19_H_15_BrN_3_O_2_^+^: 396.0342; found: 396.0341.

5-Bromo-8-methoxy-3-methyl-3,12-dihydrochromeno[4,3-*b*]pyrazolo[4,3-*f*]quinoline (**2C**)

Brown solid; 39% yield; *R_f_* 0.5 (Hex/EtOAc = 1:1); ^1^H NMR (CDCl_3_, 400 MHz): *δ* 8.30 (d, *J* = 0.8 Hz, 1H), 8.16 (s, 1H), 8.08 (d, *J* = 1.2 Hz, 1H), 8.04 (t, *J* = 2.0 Hz, 1H), 6.93 (d, *J* = 2.0 Hz, 2H), 5.37 (d, *J* = 0.8 Hz, 2H), 4.14 (s, 3H), 3.92 (s, 3H); ^13^C NMR (100 MHz, CDCl_3_): *δ* 155.30, 151.27, 147.49, 141.90, 137.45, 131.99, 127.04, 126.72, 125.26, 123.67, 122.12, 119.04, 118.75, 116.26, 117.06, 106.60, 68.31, 55.96, 36.24; HRMS *m/z* [M+H]^+^ calculated for C_19_H_15_BrN_3_O_2_^+^: 396.0342; found: 396.0341.

5-Bromo-3,10-dimethyl-3,12-dihydrochromeno[4,3-*b*]pyrazolo[4,3-*f* ]quinoline (**2D**)

Pale yellow solid; 50% yield; yield; *R_f_* 0.5 (Hex/EtOAc = 1:1); ^1^H NMR (CDCl_3_, 400 MHz): *δ* 8.39 (dd, *J* = 7.6, 1.2 Hz, 1H), 8.31 (s, 1H), 8.17 (s, 1H), 8.08 (s, 1H), 7.22 (dd, *J* = 7.6, 1.2 Hz, 1H), 7.08 (t, *J* = 7.6 Hz, 1H), 5.44 (s, 2H), 4.14 (s, 3H), 2.30 (s, 3H); ^13^C NMR (100 MHz, CDCl_3_): *δ* 155.36, 151.27, 147.49, 141.90, 137.45, 131.99, 127.04, 126.72, 125.26, 123.67, 122.12, 119.04, 118.75, 116.26, 117.08, 106.60, 68.31, 55.96, 36.24; HRMS *m/z* [M+H]^+^ calculated for C_19_H_15_BrN_3_O^+^: 380.0393; found: 380.0392.

5-Bromo-7-fluoro-3-methyl-3,12-dihydrochromeno[4,3-*b*]pyrazolo[4,3-*f*]quinoline (**2E**)

Deep yellow solid; 62% yield; yield; *R_f_* 0.4 (Hex/EtOAc = 1:1); ^1^H NMR (CDCl_3_, 400 MHz): *δ* 8.54 (dd, *J* = 7.6, 2.0 Hz, 1H), 8.30 (s, 1H), 8.17 (s, 1H), 8.09 (s, 1H), 7.37 (dd, *J* = 7.6, 2.0 Hz, 1H), 7.18 (dd, *J* = 7.6, 0.8 Hz, 1H), 7.01 (dd, *J* = 7.6, 0.8 Hz, 1H), 5.44 (s, 2H), 4.14 (s, 3H); ^13^C NMR (100 MHz, CDCl_3_): *δ* 162.55, 159.96, 158.75 (d, *J* = 19.2 Hz), 146.18 (d, *J* = 22.8 Hz), 142.31, 137.64, 132.07, 131.67 (d, *J* = 42.0 Hz), 126.96, 126.91, 125.45, 121.73, 118.53, 117.26, 113.21 (d, *J* = 15.2 Hz), 111.09 (d, *J* = 88.4 Hz), 68.58, 36.27; HRMS *m/z* [M+H]^+^ calculated for C_18_H_12_BrFN_3_O^+^: 384.0142; found: 384.0142.

5-Bromo-8-fluoro-3-methyl-3,12-dihydrochromeno[4,3-*b*]pyrazolo[4,3-*f*]quinoline (**2F**)

Deep yellow solid; 37% yield; *R_f_* 0.5 (Hex/EtOAc = 1:1); ^1^H NMR (CDCl_3_, 400 MHz): *δ* 8.32 (s, 1H), 8.23–8.20 (m, 2H), 8.12 (s, 1H), 7.05 (td, *J* = 8.4, 3.2 Hz, 1H), 6.96 (dd, *J* = 8.4, 4.0 Hz, 1H), 5.42 (s, 2H), 4.15 (s, 3H); ^13^C NMR (100 MHz, CDCl_3_): *δ* 159.83, 153.08 (d, *J* = 8.0 Hz), 146.69 (d, *J* = 7.6 Hz), 142.08, 137.59, 132.07, 126.91, 126.66, 125.28, 122.43, 118.72 (d, *J* = 34.8 Hz), 118.61 (d, *J* = 30.8 Hz), 118.52, 117.34, 111.70, 111.46, 68.33, 36.29; HRMS *m/z* [M+H]^+^ calculated for C_18_H_12_BrFN_3_O^+^: 384.0142; found: 384.0141.

5-Bromo-9-fluoro-3-methyl-3,12-dihydrochromeno[4,3-*b*]pyrazolo[4,3-*f*]quinoline (**2G**)

Yellow solid; 64% yield; *R_f_* 0.6 (Hex/EtOAc = 1:1); ^1^H NMR (CDCl_3_, 400 MHz): *δ* 8.51 (d, *J* = 8.8, 6.8 Hz, 1H), 8.29 (d, *J* = 1.2 Hz, 1H), 8.18 (s, 1H), 8.09 (d, *J* = 0.8 Hz, 1H), 6.88 (td, *J* = 8.8, 2.4 Hz, 1H), 6.71 (dd, *J* = 9.6, 2.4 Hz, 1H), 5.45 (d, *J* = 0.8 Hz, 2H), 4.14 (s, 3H); ^13^C NMR (100 MHz, CDCl_3_): *δ* 166.39, 163.89, 158.28 (d, *J* = 50.0 Hz), 146.78, 142.03, 137.40, 131.95, 127.37 (d, *J* = 42.0 Hz), 126.86, 125.44 (d, *J* = 256.8 Hz), 121.96, 119.60 (d, *J* = 8.0 Hz), 118.71, 117.25, 110.34 (d, *J* = 88.0 Hz), 104.70 (d, *J* = 100.4 Hz), 68.58, 36.26; HRMS *m/z* [M+H]^+^ calculated for C_18_H_12_BrFN_3_O^+^: 384.0142; found: 384.0142.

5-Bromo-10-fluoro-3-methyl-3,12-dihydrochromeno[4,3-*b*]pyrazolo[4,3-*f*]quinoline (**2H**)

Yellow solid; 45% yield; *R_f_* 0.6 (Hex/EtOAc = 1:1); ^1^H NMR (CDCl_3_, 400 MHz): *δ* 8.34 (s, 1H), 8.33 (td, *J* = 7.6, 1.6 Hz, 1H), 8.24 (s, 1H), 8.13 (d, *J* = 0.8 Hz, 1H), 7.20–7.15 (m, 1H), 7.13–7.08 (m, 1H), 5.54 (d, *J* = 0.8 Hz, 2H), 4.17 (s, 3H); ^13^C NMR (100 MHz, CDCl_3_): *δ* 152.99, 150.55, 146.42 (d, *J* = 16.8 Hz), 144.83 (d, *J* = 46.0 Hz), 142.00, 137.47, 132.02, 127.00, 126.25, 125.41, 125.13, 122.21 (d, *J* = 30.8 Hz), 120.69 (d, *J* = 15.2 Hz), 118.59, 118.16 (d, *J* = 72.8 Hz), 117.36, 68.54, 36.22; HRMS *m/z* [M+H]^+^ calculated for C_18_H_12_BrFN_3_O^+^: 384.0142; found: 384.0142.

5-Bromo-7-chloro-3-methyl-3,12-dihydrochromeno[4,3-*b*]pyrazolo[4,3-*f*]quinoline (**2I**)

Orange yellow solid; 44% yield; *R_f_* 0.6 (Hex/EtOAc = 1:1); ^1^H NMR (CDCl_3_, 400 MHz): *δ* 8.35 (d, *J* = 0.8 Hz, 1H), 8.29 (s, 1H), 8.15 (d, *J* = 1.2 Hz, 1H), 7.28–7.23 (m, 2H), 6.99 (dd, *J* = 6.4, 2.4 Hz, 1H), 5.33 (s, 2H), 4.17 (s, 3H); ^13^C NMR (100 MHz, CDCl_3_): *δ* 155.97, 146.06, 142.00, 137.50, 134.47, 132.01, 128.17, 126.94, 128.56, 126.32, 124.86, 122.33, 119.24, 118.56, 117.39, 115.41, 68.24, 36.27; HRMS *m/z* [M+H]^+^ calculated for C_18_H_12_BrClN_3_O^+^: 399.9847; found: 399.9846.

5-Bromo-8-chloro-3-methyl-3,12-dihydrochromeno[4,3-*b*]pyrazolo[4,3-*f*]quinoline (**2J**)

Deep yellow solid; 43% yield; *R_f_* 0.5 (Hex/EtOAc = 1:1); ^1^H NMR (CDCl_3_, 400 MHz): *δ* 8.48 (d, *J* = 2.8 Hz, 1H), 8.32 (d, *J* = 1.2 Hz, 1H), 8.21 (s, 1H), 8.12 (s, 1H), 7.30 (dd, *J* = 8.8, 2.8 Hz, 1H), 6.95 (d, *J* = 8.8 Hz, 1H), 5.45 (d, *J* = 0.8 Hz, 2H), 4.16 (s, 3H); ^13^C NMR (100 MHz, CDCl_3_): *δ* 155.52, 149.17, 146.30, 142.10, 137.58, 132.09, 131.62, 128.07, 126.99, 126.42, 125.26, 124.51, 122.43, 118.85, 118.64, 117.46, 68.31, 36.30; HRMS *m/z* [M+H]^+^ calculated for C_18_H_12_BrClN_3_O^+^: 399.9847; found: 399.9846.

5-Bromo-9-chloro-3-methyl-3,12-dihydrochromeno[4,3-*b*]pyrazolo[4,3-*f*]quinoline (**2K**)

Pale yellow solid; 43% yield; *R_f_* 0.4 (Hex/EtOAc = 2:1); ^1^H NMR (CDCl_3_, 400 MHz): *δ* 8.47 (d, *J* = 8.8 Hz, 1H), 8.32 (s, 1H), 8.19 (s, 1H), 8.12 (s, 1H), 7.15 (dd, *J* = 8.8, 2.4 Hz, 1H), 7.03 (d, *J* = 2.4 Hz, 1H), 5.46 (s, 2H), 4.16 (s, 3H); ^13^C NMR (100 MHz, CDCl_3_): *δ* 157.48, 146.61, 142.09, 137.50, 137.22, 132.05, 126.91, 126.74, 126.13, 125.15, 123.25, 122.22, 121.84, 118.71, 117.68, 117.38, 68.49, 36.30; HRMS *m/z* [M+H]^+^ calculated for C_18_H_12_BrClN_3_O^+^: 399.9847; found: 399.9846.

5-Bromo-10-chloro-3-methyl-3,12-dihydrochromeno[4,3-*b*]pyrazolo[4,3-*f*]quinoline (**2L**)

Pale yellow solid; 50% yield; *R_f_* 0.5 (Hex/EtOAc = 1:1); ^1^H NMR (CDCl_3_, 400 MHz): *δ* 8.48 (dd, *J* = 7.6, 1.6 Hz, 1H), 8.33 (d, *J* = 0.8 Hz, 1H), 8.23 (s, 1H), 8.12 (d, *J* = 1.2 Hz, 1H), 7.43 (dd, *J* = 7.6, 1.6 Hz, 1H), 7.12 (t, *J* = 7.6 Hz, 1H), 5.56 (d, *J* = 0.8 Hz, 2H), 4.15 (s, 3H); ^13^C NMR (100 MHz, CDCl_3_): *δ* 152.55, 146.51, 142.08, 137.49, 132.13, 132.05, 126.94, 126.04, 125.16, 124.66, 124.13, 122.87, 122.30, 122.22, 118.59, 117.31, 68.64, 36.23; HRMS *m/z* [M+H]^+^ calculated for C_18_H_12_BrClN_3_O^+^: 399.9847; found: 399.9846.

5-Bromo-8,10-dichloro-3-methyl-3,12-dihydrochromeno[4,3-*b*]pyrazolo[4,3-*f*]quinoline (**2M**)

Yellow solid; 38% yield; *R_f_* 0.4 (Hex/EtOAc = 2:1); ^1^H NMR (CDCl_3_, 400 MHz): *δ* 8.42 (d, *J* = 2.4 Hz, 1H), 8.33 (s, 1H), 8.25 (s, 1H), 8.14 (s, 1H), 7.41 (d, *J* = 2.4 Hz, 1H), 5.56 (s, 2H), 4.17 (s, 3H); ^13^C NMR (100 MHz, CDCl_3_): *δ* 151.24, 145.27, 142.09, 137.60, 132.19, 131.57, 127.75, 127.14, 125.81, 125.37, 125.06, 123.86, 123.17, 122.63, 118.51, 117.71, 68.72, 36.32; HRMS *m/z* [M+H]^+^ calculated for C_18_H_11_BrCl_2_N_3_O^+^: 433.9457; found: 433.9456.

5-Bromo-8-bromo-3-methyl-3,12-dihydrochromeno[4,3-*b*]pyrazolo[4,3-*f*]quinoline (**2N**)

Pale yellow solid; 24% yield; *R_f_* 0.4 (Hex/EtOAc = 1:1); ^1^H NMR (CDCl_3_, 400 MHz): *δ* 8.57 (d, *J* = 2.8 Hz, 1H), 8.29 (d, *J* = 0.8 Hz, 1H), 8.16 (s, 1H), 8.09 (d, *J* = 0.8 Hz, 1H), 7.42 (dd, *J* = 8.4, 2.8 Hz, 1H), 6.88 (d, *J* = 8.4 Hz, 1H), 5.44 (d, *J* = 1.2 Hz, 2H), 4.15 (s, 3H); ^13^C NMR (100 MHz, CDCl_3_): *δ* 156.00, 146.12, 142.06, 137.55, 134.48, 132.07, 128.22, 126.99, 126.36, 125.22, 124.92, 122.40, 119.25, 118.62, 117.44, 115.43, 68.26, 36.29; HRMS *m/z* [M+H]^+^ calculated for C_18_H_12_Br_2_N_3_O^+^: 433.9342; found: 433.9341.

5-Bromo-9-bromo-3-methyl-3,12-dihydrochromeno[4,3-*b*]pyrazolo[4,3-*f*]quinoline (**2O**)

Pale yellow solid; 47% yield; *R_f_* 0.5 (Hex/EtOAc = 1:1); ^1^H NMR (CDCl_3_, 400 MHz): *δ* 8.50 (dd, *J* = 8.0, 1.6 Hz, 1H), 8.30 (d, *J* = 1.2 Hz, 1H), 8.19 (s, 1H), 8.09 (d, *J* = 1.2 Hz, 1H), 7.58 (dd, *J* = 8.0, 1.6 Hz, 1H), 7.05 (t, *J* = 8.0 Hz, 1H), 5.55 (d, *J* = 0.8 Hz, 2H), 4.14 (s, 3H); ^13^C NMR (100 MHz, CDCl_3_): *δ* 157.47, 146.60, 142.07, 137.49, 137.22, 132.03, 126.91, 126.73, 126.13, 125.16, 123.25, 122.21, 121.82, 118.69, 117.67, 117.39, 48.49, 36.29; HRMS *m/z* [M+H]^+^ calculated for C_18_H_12_Br_2_N_3_O^+^: 433.9342; found: 433.9341.

5-Bromo-10-bromo-3-methyl-3,12-dihydrochromeno[4,3-*b*]pyrazolo[4,3-*f*]quinoline (**2P**)

White yellow solid; 50% yield; *R_f_* 0.3 (Hex/EtOAc = 2:1); ^1^H NMR (CDCl_3_, 400 MHz): *δ* 8.50 (dd, *J* = 8.0, 1.6 Hz, 1H), 8.30 (d, *J* = 1.2 Hz, 1H), 8.19 (s, 1H), 8.09 (d, *J* = 1.2 Hz, 1H), 7.58 (dd, *J* = 8.0, 1.6 Hz, 1H), 7.05 (t, *J* = 8.0 Hz, 1H), 5.55 (d, *J* = 0.8 Hz, 2H), 4.14 (s, 3H); ^13^C NMR (100 MHz, CDCl_3_): *δ* 153.59, 146.60, 142.18,137.57, 135.23, 132.12,126.96, 126.10, 125.25, 124.98, 124.73, 123.56, 122.39, 118.67, 117.45, 111.25, 68.75, 36.28; HRMS *m/z* [M+H]^+^ calculated for C_18_H_12_Br_2_N_3_O^+^: 433.9342; found: 433.9341.

12-Bromo-10-methyl-6,10-dihydrobenzo[7,8]chromeno[4,3-*b*]pyrazolo[4,3-*f*]quinoline (**2Q**)

Orange brown solid; 40% yield; *R_f_* 0.6 (Hex/EtOAc = 1:1); ^1^H NMR (CDCl_3_, 400 MHz): *δ* 8.63 (d, *J* = 8.8 Hz, 1H), 8.35 (s, 1H), 8.29 (d, *J* = 7.6 Hz, 1H), 8.24 (s, 1H), 8.12 (s, 1H), 7.84 (dd, *J* = 6.8, 2.4 Hz, 1H), 7.62 (d, *J* = 8.8 Hz, 1H), 7.57–7.49 (m, 1H), 5.67 (s, 2H), 4.17 (s, 3H); ^13^C NMR (100 MHz, CDCl_3_): *δ* 153.53, 148.03, 142.15, 137.46, 135.92, 131.98, 128.07, 127.73, 126.61, 126.23, 126.02, 125.37, 125.14, 122.54, 122.24, 121.99, 118.89, 117.66, 117.05, 68.78, 36.27; HRMS *m/z* [M+H]^+^ calculated for C_22_H_15_BrN_3_O^+^: 416.0393; found: 416.0392.

14-Bromo-12-methyl-8,12-dihydrobenzo[5,6]chromeno[4,3-*b*]pyrazolo[4,3-*f*]quinoline (**2R**)

Brown solid; 68% yield; *R_f_* 0.4 (Hex/EtOAc = 1:1); ^1^H NMR (CDCl_3_, 400 MHz): *δ* 10.34 (d, *J* = 8.8 Hz, 1H), 8.38 (s, 1H), 8.32 (s, 1H), 8.16 (s, 1H), 7.87 (d, *J* = 9.2 Hz, 1H), 7.85 (dd, *J* = 8.4, 1.6 Hz, 1H), 7.74 (ddd, *J* = 8.4, 6.8, 1.6 Hz, 1H), 7.52–7.48 (m, 1H), 7.23 (d, *J* = 8.4 Hz, 1H), 5.42 (s, 2H), 4.19 (s, 3H); ^13^C NMR (100 MHz, CDCl_3_): *δ* 157.59, 149.35, 142.03, 137.59, 133.36, 132.00, 131.28, 130.93, 128.58, 128.24, 127.97, 127.82, 126.87, 125.70, 124.75, 121.32, 118.66, 118.32, 116.95, 116.03, 68.60, 36.28; HRMS *m/z* [M+H]^+^ calculated for C_22_H_15_BrN_3_O^+^: 416.0393; found: 416.0392.

8-Allyl-5-bromo-10-methoxy-3-methyl-3,12-dihydrochromeno[4,3-*b*]pyrazolo[4,3-*f*]quinoline (**2S**)

Brown solid; 33% yield; *R_f_* 0.4 (Hex/EtOAc = 1:1); ^1^H NMR (CDCl_3_, 400 MHz): *δ* 8.31 (d, *J* = 0.8 Hz, 1H), 8.21 (s, 1H), 8.11 (d, *J* = 1.2 Hz, 1H), 7.98 (d, *J* = 2.0 Hz, 1H), 6.82 (d, *J* = 2.0 Hz, 1H), 6.61–6.01 (m, 1H), 5.49 (d, *J* = 0.8 Hz, 2H), 5.21–5.16 (m, 1H), 5.15–5.13 (m, 1H), 5.44 (s, 2H), 4.15 (s, 3H), 3.93 (s, 3H), 3.49 (d, *J* = 7.2 Hz, 2H); ^13^C NMR (100 MHz, CDCl_3_): *δ* 148.73, 147.55, 144.87, 141.98, 137.60, 137.46, 134.25, 131.09, 126.83, 126.75, 125.29, 123.67, 122.10, 118.73, 117.12, 116.93, 116.15, 114.25, 68.62, 56.27, 40.38, 36.24; HRMS *m/z* [M+H]^+^ calculated for C_22_H_19_BrN_3_O_2_^+^: 436.0655; found: 436.0653.

14-Bromo-9,12-dimethyl-8,12-dihydrobenzo[5,6]chromeno[4,3-*b*]pyrazolo[4,3-*f*]quinoline (**2T**)

Orange brown solid; 23% yield; *R_f_* 0.5 (Hex/EtOAc = 1:1); ^1^H NMR (CDCl_3_, 400 MHz): *δ* 10.28 (d, *J* = 8.8 Hz, 1H), 8.46 (d, *J* = 0.8 Hz, 1H), 8.19 (d, *J* = 0.8 Hz, 1H), 7.86 (d, *J* = 8.8 Hz, 1H), 7.84 (d, *J* = 8.0 Hz, 1H), 7.71 (ddd, *J* = 8.8, 6.8, 2.0 Hz, 1H), 7.48 (ddd, *J* = 8.0, 6.8, 2.0 Hz, 1H), 7.22 (d, *J* = 8.8 Hz, 1H), 5.53 (s, 2H), 4.21 (s, 3H), 2.90 (s, 3H); ^13^C NMR (100 MHz, CDCl_3_): *δ* 157.29, 148.44, 141.73, 138.10, 137.88, 134.70, 133.15, 131.36, 131.00, 128.56, 128.04, 127.94, 126.75, 126.49, 124.62, 122.53, 118.11, 118.04, 116.90, 116.29, 66.04, 36.25, 17.76; HRMS *m/z* [M+H]^+^ calculated for C_23_H_17_BrN_3_O^+^: 430.0550; found: 430.0547.

## 5. Conclusions

In summary, we have presented a design of 20 diphenyl-pyrazoloquinolines **1** and 20 chromeno-pyrazoloquinolines **2** and their successful synthesis via inverse imino Diels–Alder reaction in microwave conditions. The straightforward microwave protocol is advantageous in many ways, as it is economical, single-step reaction, multicomponent reaction and has broad functional group tolerance and so forth. In vitro cytotoxicity evaluation indicated strong activity of 8 (**1B**, **1C**, **1M**, **2A**, **2D**, **2E**, **2F**, and **2R**) compounds out of 40 against NUGC-3 human cancer cell proliferation (less than 30% growth compared with vehicle-treated sets at 30 µM). Among the 8 compounds, **1M**, **2E**, and **2P** demonstrated high inhibition of cancer cell proliferation (GI_50_ less than 8 µM) in six different human cancer cell lines (ACHN, HCT-15, MM231, NCI-H23, NUGC-3, and PC-3). Finally, the compounds **2E** and **2P** having consistent GI_50_ values in tested cancer cells were selected and accessed for their mode of action via topo I and topo IIα enzyme assay. The results demonstrated that cytotoxicity of the compound **2E** was achieved via inhibition of the activity of the topo IIα enzyme and blocked relaxation of supercoiled plasmid DNA by 88.3%, which is almost equal to that of positive control, etoposide (89.6%), at 100 µM. Despite high cytotoxicity of the compounds **2E** and **2P**, marginal effects were observed against topo I activity compared with camptothecin. Unfortunately, **2P** was completely inactive to topo IIα activity.

## Figures and Tables

**Table 1:** Synthesis of 7,9-diphenyl-3*H*-pyrazolo[4,3-*f*]quinoline ^a, b^.

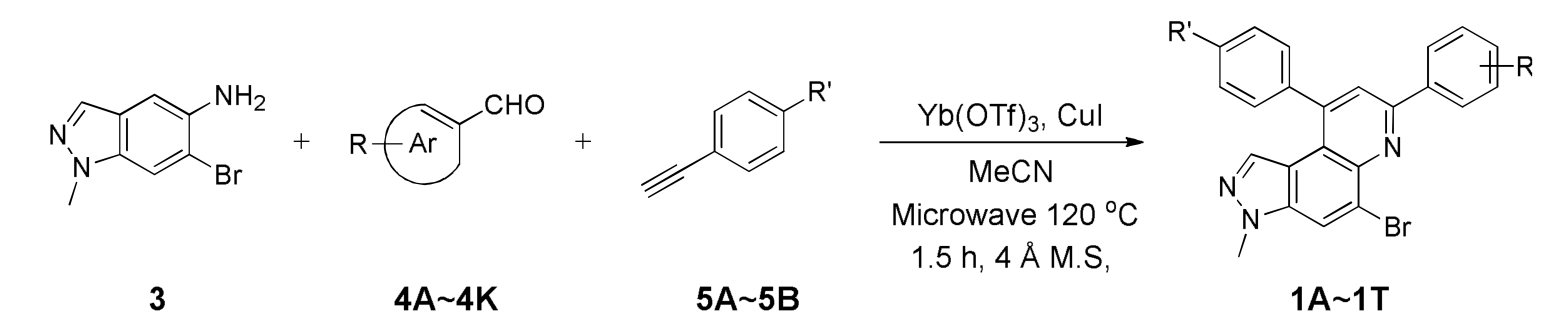
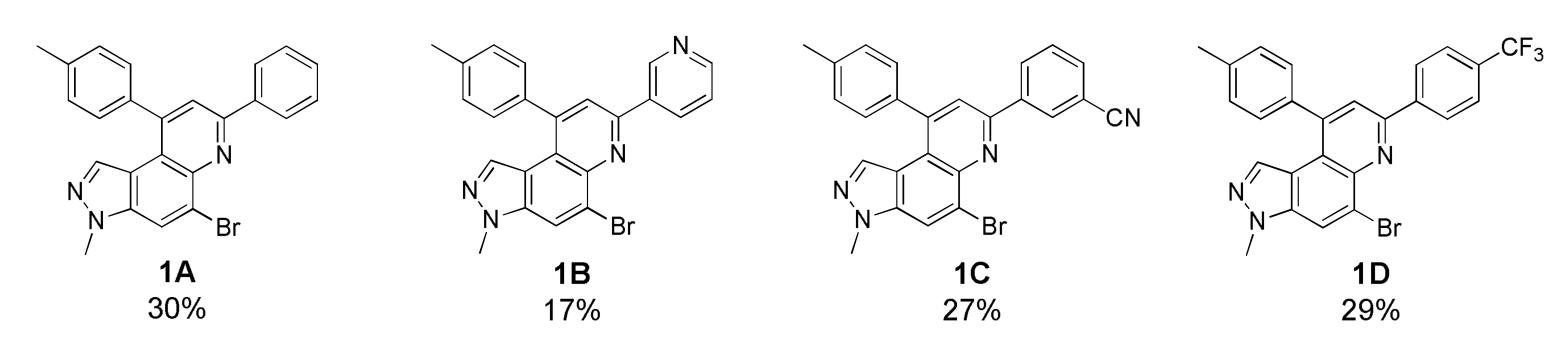
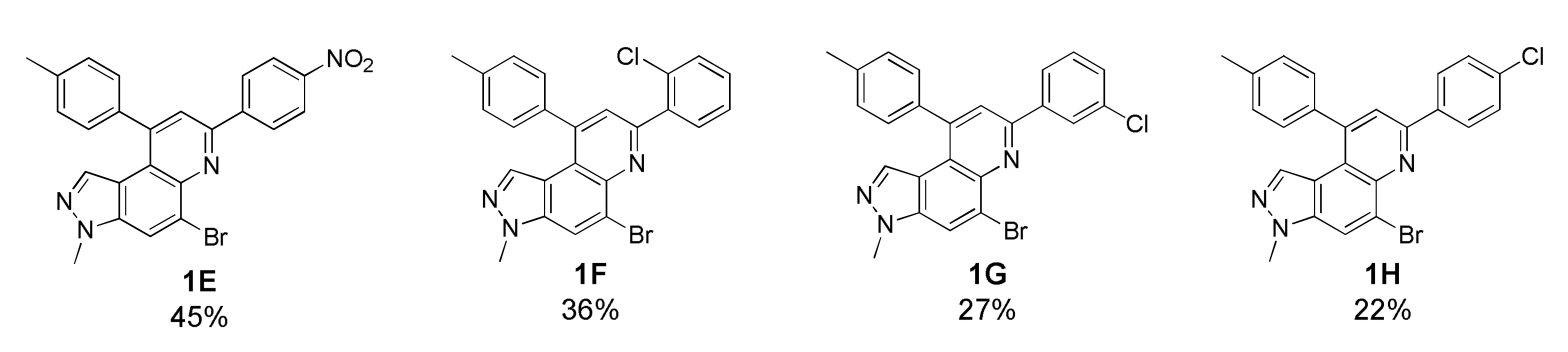
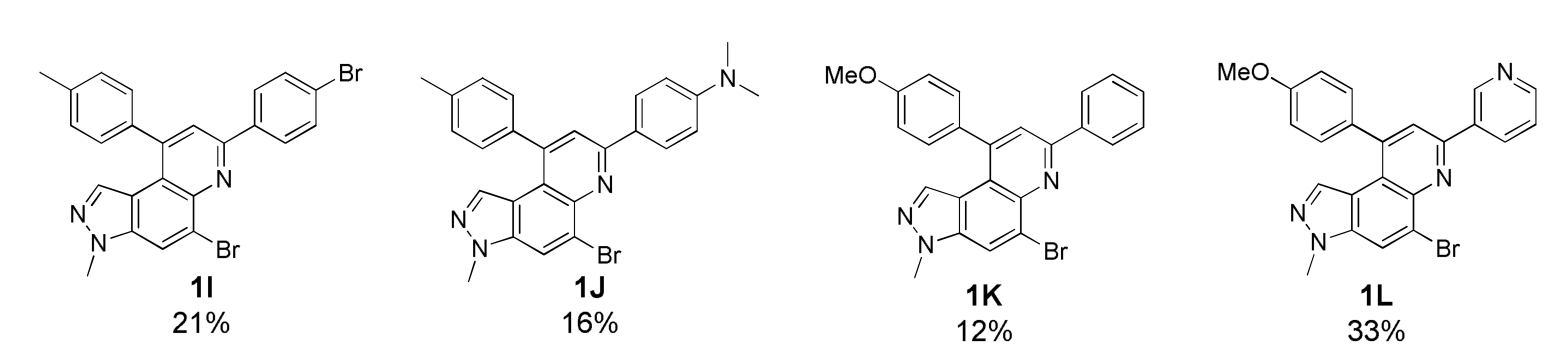
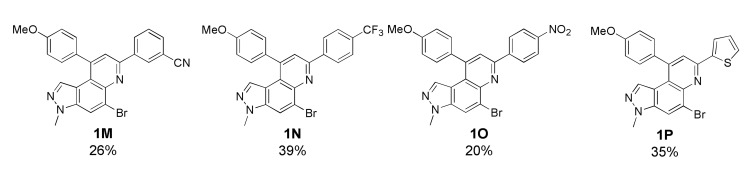
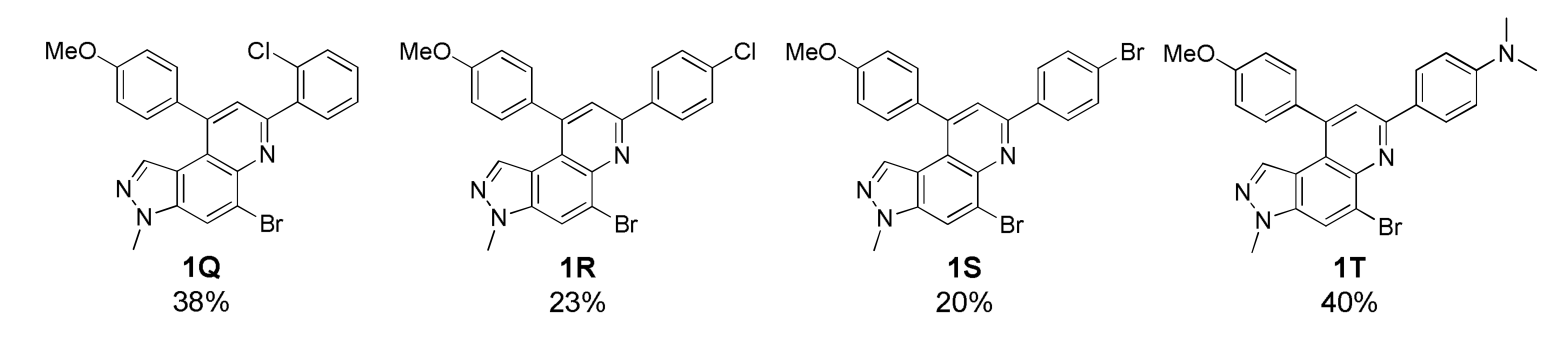

^a^ Reaction conditions: substrates **3** (0.44 mmol), **4** (0.44 mmol), **5** (0.44 mmol), Yb(OTf)_3_ (20 mol%), and CuI (20 mol%) in 1 mL of MeCN. ^b^ Isolated yields.

**Table 2:**
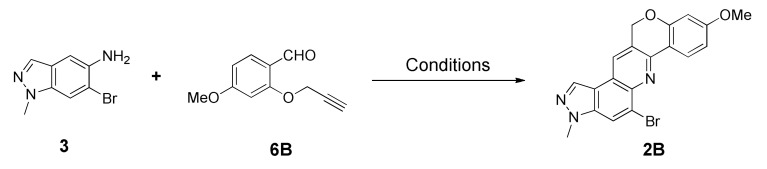
Optimization of reaction conditions for intramolecular DA reaction ^a^.

Entry	Conditions				
	Catalyst	Additive	Solvent	Time (h)	Yield ^e^
1	Yb(OTf)_3_/CuI ^b^	-	MeCN	19	<5%
2	Yb(OTf)_3_/CuI	-	Xylene	19	18%
3	La(OTf)_3_/CuI	-	Xylene	19	42%
4	Sc(OTf)_3_/CuI	-	Xylene	19	18%
5	Yb(OTf)_3_/CuI	-	Toluene	19	16%
6	La(OTf)_3_/CuI	-	Toluene	19	21%
7	Sc(OTf)_3_/CuI	-	Toluene	19	17%
8	Yb(OTf)_3_/CuI	-	DMF	19	29%
9	La(OTf)_3_/CuI	-	DMF	19	43%
10	Sc(OTf)_3_/CuI	-	DMF	19	21%
11	Yb(OTf)_3_/CuI	-	MeCN	19	<5%
12	La(OTf)_3_/CuI	-	MeCN	19	<5%
13	Sc(OTf)_3_/CuI	-	MeCN	19	<5%
14	La(OTf)_3_/CuI ^c^	-	DMF	3.5	46%
15	CuI ^c^	-	Xylene	3.5	15%
16	La(OTf)_3_^c^	AgSbF_6_	Xylene	3.5	37%
17	La(OTf)_3_/CuI ^c^	AgSbF_6_	Xylene	3.5	19%
18	La(OTf)_3_/CuI ^c^	AgSbF_6_^d^	Xylene	3.5	3%

^a^ Reaction conditions: substrates **3** (0.44 mmol), **6B** (0.44 mmol or ^b^ 0.22 mmol), Yb(OTf)_3_ (20 mol%), CuI (20 mol%), and AgSbF_6_ (20 mol% or ^d^ 100 mol%) in 2 mL of refluxing solvents. ^c^ Microwave conditions (175 °C, 200 watt). ^e^ Isolated yield.

**Table 3:** Synthesis of chromeno[4,3-*b*]pyrazolo[4,3-*f*]quinoline ^a, b^.

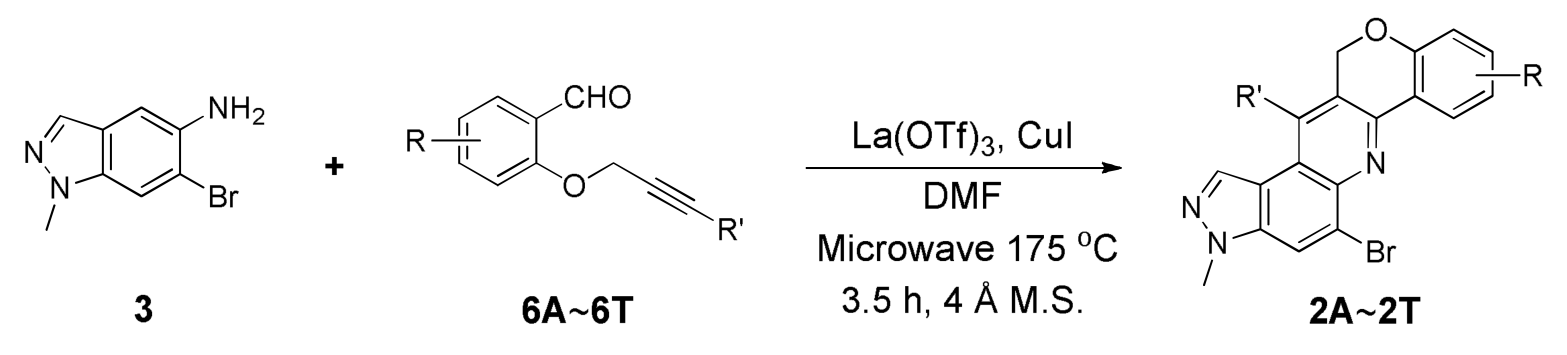
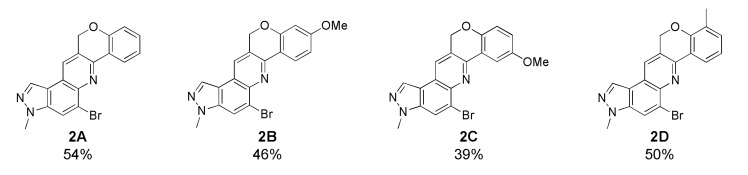
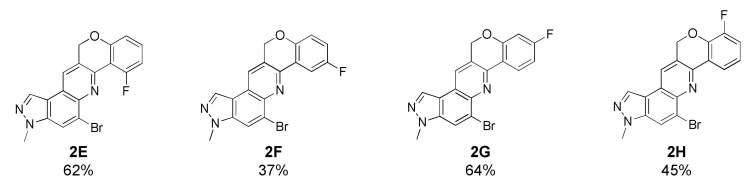
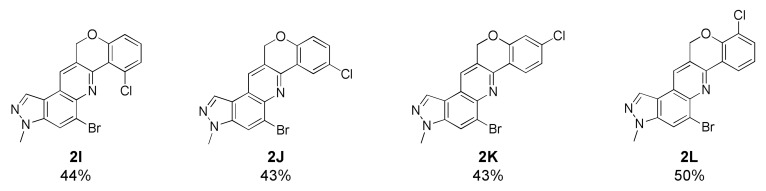
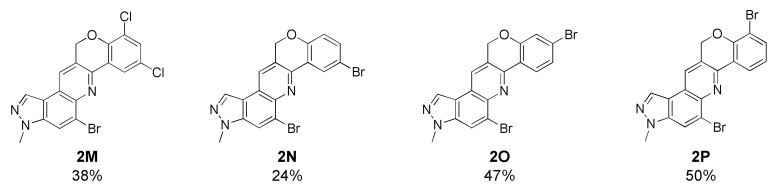
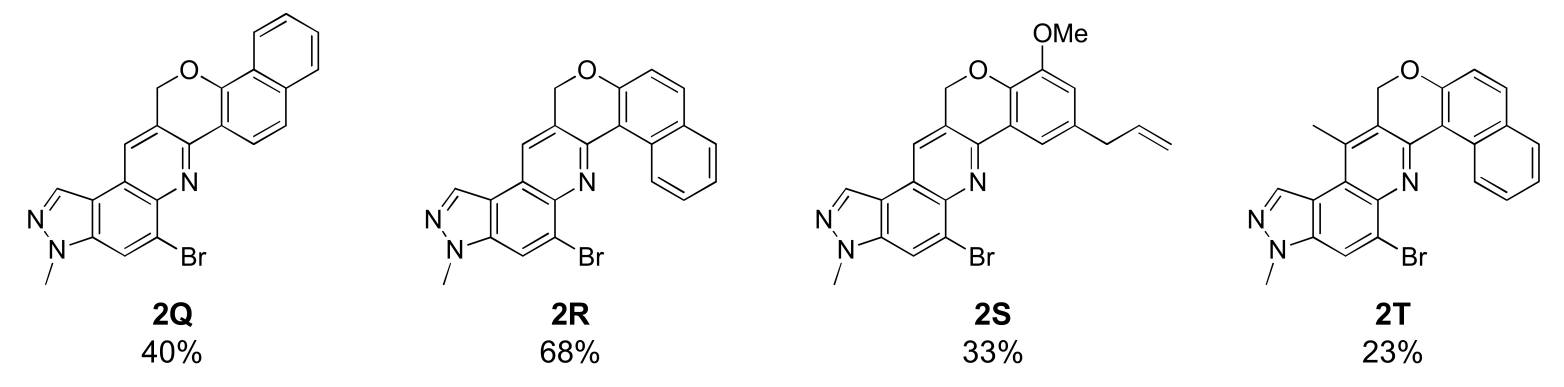

^a^ Reaction conditions: substrates **3** (0.44 mmol), **6** (0.44 mmol), Yb(OTf)_3_ (20 mol%), and CuI (20 mol%) in 1 mL of DMF. ^b^ Isolated yields.

**Table 4:**
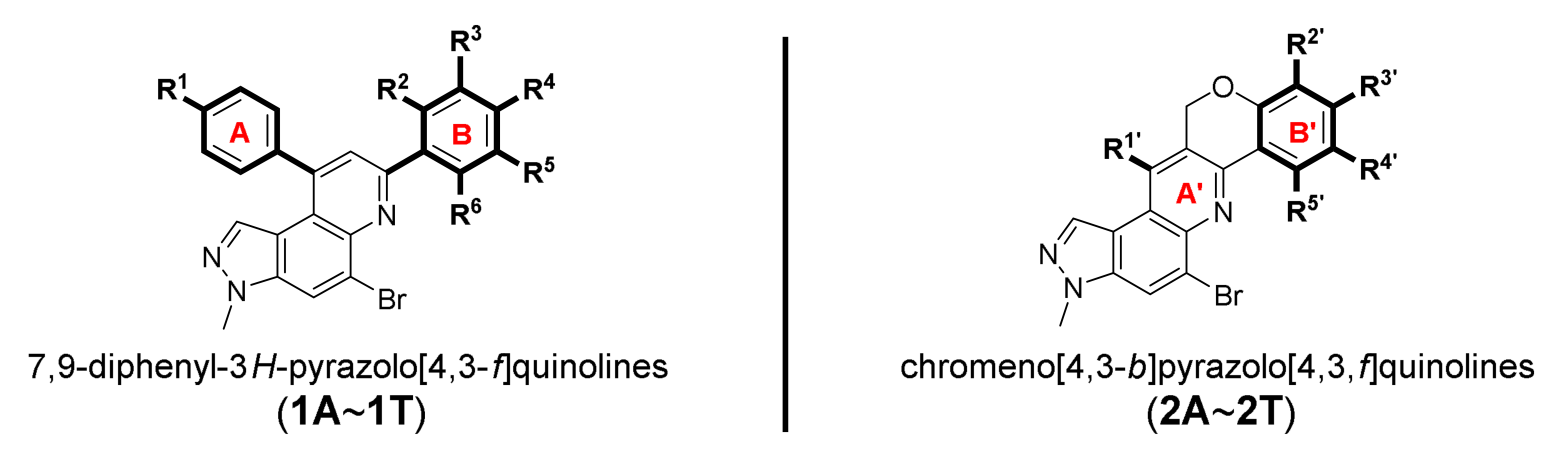
In vitro antiproliferative activity of synthesized pyrazolo[4,3-*f*]quinoline analogues in human NUGC-3 cell line at 30 µM concentration.

Cpd.	Substituents	Cell Proliferation (% Control) ^a^	Cpd.	Substituents	Cell Proliferation (% Control) ^a^
R^1^ = Me			R^1^′ = H		
**1A**	R^2^, R^3^, R^4^, R^5^, R^6^ = H	78.40 ± 4.95	**2A**	R^2^′, R^3^′, R^4^′, R^5^′ = H	19.41 ± 6.67
**1B**	Pyridin-3-yl	22.89 ± 4.49	**2B**	R^2^′, R^4^′, R^5^′ = H; R^3^′ = OMe	86.51 ± 2.72
**1C**	R^2^, R^3^, R^4^, R^6^ = H; R^5^ = CN	9.80 ± 5.89	**2C**	R^2^′, R^3^′, R^5^′ = H; R^4^′ = OMe	77.36 ± 6.80
**1D**	R^2^, R^3^, R^5^, R^6^ = H; R^4^ = CF_3_	81.29 ± 7.33	**2D**	R^3^′, R^4^′, R^5^′ = H; R^2^′ = Me	27.68 ± 3.06
**1E**	R^2^, R^3^, R^5^, R^6^ = H; R^4^ = NO_2_	88.89 ± 4.20	**2E**	R^2^′, R^3^′, R^4^′ = H; R^5^′ = F	−17.83 ± 1.63
**1F**	R^3^, R^4^, R^5^, R^6^ = H; R^2^ = Cl	82.79 ± 3.63	**2F**	R^2^′, R^3^′, R^5^′ = H; R^4^′ = F	14.67 ± 6.46
**1G**	R^2^, R^3^, R^4^, R^6^ = H; R^5^ = Cl	76.66 ± 8.59	**2G**	R^2^′, R^4^′, R^5^′ = H; R^3^′ = F	76.07 ± 2.78
**1H**	R^2^, R^3^, R^5^, R^6^ = H; R^4^ = Cl	86.35 ± 8.70	**2H**	R^3^′, R^4^′, R^5^′ = H; R^2^′ = F	85.39 ± 4.24
**1I**	R^2^, R^3^, R^5^, R^6^ = H; R^4^ = Br	80.57 ± 2.74	**2I**	R^2^′, R^3^′, R^4^′ = H; R^5^′ = Cl	69.54 ± 4.23
**1J**	R^2^, R^3^, R^5^, R^6^ = H; R^4^ = N(CH_3_)_2_	74.52 ± 7.91	**2J**	R^2^′, R^3^′, R^5^′ = H; R^4^′ = Cl	86.56 ± 5.28
R^1^ = OMe			**2K**	R^2^′, R^4^′, R^5^′ = H; R^3^′ = Cl	77.49 ± 7.38
**1K**	R^2^, R^3^, R^4^, R^5^, R^6^ = H	74.57 ± 7.34	**2L**	R^3^′, R^4^′, R^5^′ = H; R^2^′ = Cl	82.07 ± 10.94
**1L**	Pyridin-3-yl	79.68 ± 4.00	**2M**	R^3^′, R^5^′ = H; R^2^′, R^4^′ = Cl	64.13 ± 2.78
**1M**	R^2^, R^3^, R^4^, R^6^ = H; R^5^ = CN	−29.01 ± 0.84	**2N**	R^2^′, R^3^′, R^5^′ = H; R^4^′ = Br	83.43 ± 6.92
**1N**	R^2^, R^3^, R^5^, R^6^ = H; R^4^ = CF_3_	84.37 ± 7.71	**2O**	R^2^′, R^4^′, R^5^′ = H; R^3^′ = Br	86.72 ± 4.67
**1O**	R^2^, R^3^, R^5^, R^6^ = H; R^4^ = NO_2_	90.20 ± 5.89	**2P**	R^3^′, R^4^′, R^5^′ = H; R^2^′ = Br	−22.67 ± 4.55
**1P**	Thiophen-2-yl	86.43 ± 7.19	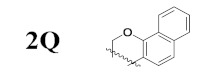 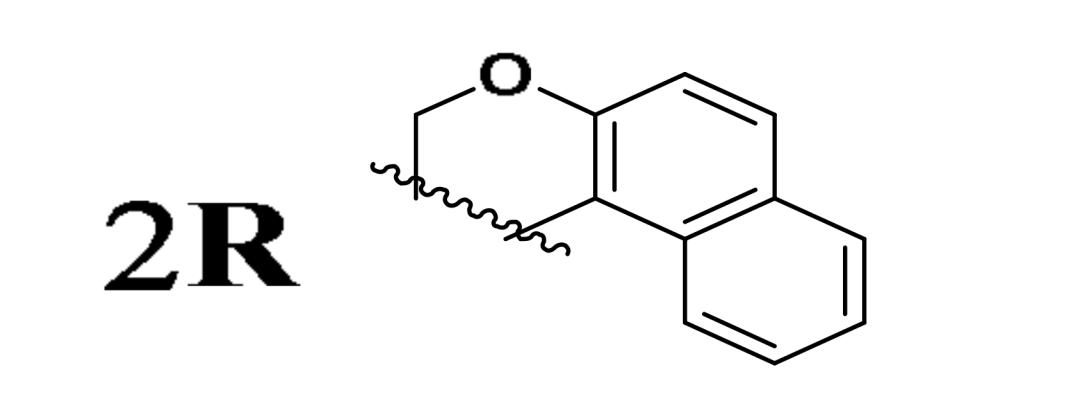		**2Q** = 81.99 ± 7.50
**1Q**	R^3^, R^4^, R^5^, R^6^ = H; R^2^ = Cl	72.54 ± 4.79			**2R** = 11.16 ± 6.95
**1R**	R^2^, R^3^, R^5^, R^6^ = H; R^4^ = Cl	81.53 ± 7.85	**2S**	R^2^′, R^6^′ = H; R^1^′ = OMe; R^4^′ = prop-1-ene	74.09 ± 8.60
**1S**	R^2^, R^3^, R^5^, R^6^ = H; R^4^ = Br	84.34 ± 3.56	R^1^′ = Me		
**1T**	R^2^, R^3^, R^5^, R^6^ = H; R^4^ = N(CH_3_)_2_	88.12 ± 5.80	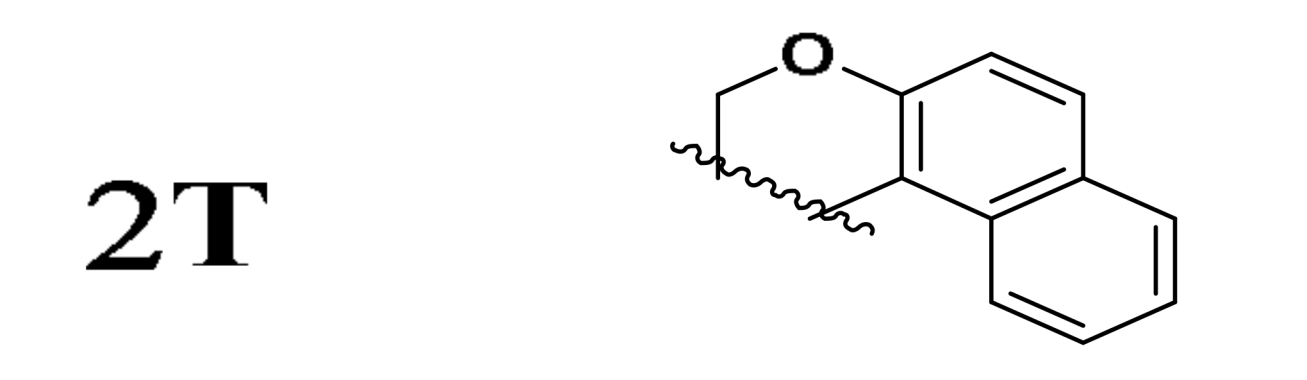		83.99 ± 5.38
Doxorubicin (ADR), −17.38 ± 2.68% at 3 µM.					

^a^ Means and standard deviations (SD) were taken as a mean from three independent experiments and correspond to the % of net cell proliferation on day 3 compared with day 0. Compounds (Cpd.).

**Table 5:** In vitro antiproliferative activity of selected compounds in six different human cancer cell lines.

Cpd.	Human Cancer Cell Lines, GI_50_ (µM) ^a^					
	ACHN	HCT-15	MM231	NCI-H23	NUGC-3	PC-3
ADR	0.400 ± 0.456	0.088 ± 0.005	0.085 ± 0.002	0.069 ± 0.001	0.081 ± 0.005	0.417 ± 0.481
**1B**	8.412 ± 0.396	9.402 ± 0.888	7.935 ± 0.485	7.862 ± 0.235	7.946 ± 0.086	7.262 ± 1.339
**1C**	6.762 ± 0.706	7.872 ± 0.607	4.514 ± 0.170	4.794 ± 0.518	6.521 ± 0.116	6.068 ± 1.101
**1M**	4.305 ± 0.378	6.569 ± 0.193	5.894 ± 0.164	4.391 ± 0.540	5.321 ± 0.579	7.236 ± 0.466
**2A**	9.163 ± 1.382	11.970 ± 1.867	13.555 ± 1.648	11.670 ± 0.580	8.010 ± 0.885	12.125 ± 1.039
**2D**	11.476 ± 2.424	11.310 ± 0.594	15.655 ± 1.266	10.352 ± 1.539	7.628 ± 0.640	13.760 ± 4.073
**2E**	5.020 ± 0.390	5.201 ± 0.449	5.989 ± 0.730	4.359 ± 0.139	3.279 ± 0.609	4.666 ± 0.189
**2F**	14.025 ± 1.082	11.615 ± 1.096	11.670 ± 1.754	13.355 ± 0.771	8.355 ± 0.109	9.757 ± 2.140
**2P**	3.843 ± 0.031	6.443 ± 1.705	5.234 ± 0.260	6.494 ± 0.981	3.930 ± 0.133	5.901 ± 0.281
**2R**	13.760 ± 2.121	12.000 ± 0.735	13.965 ± 0.502	7.580 ± 0.617	8.866 ± 0.211	7.726 ± 1.540

^a^ The GI_50_ values were obtained by three independent experiments.

## Data Availability

Data is contained within the article and Supplementary Materials.

## Supplementary Materials

The following supporting information can be downloaded at: https://www.mdpi.com/article/10.3390/ph15040399/s1, Scheme S1. Synthesis of starting precursor 3; Scheme S2. A plausible mechanism of intermolecular Diels-Alder reaction; Scheme S3. A plausible mechanism of intramolecular Diels-Alder reaction; Scheme S4. Representative synthetic method of compounds 6; Scheme S5. A plausible mechanism of benzofuran formation.
