# Supplementary material for: Design, Synthesis, and Cytotoxicity and Topoisomerase I/IIα Inhibition Activity of Pyrazolo[4,3-f]quinoline Derivatives
Source: Pharmaceuticals (Basel). 2022 Mar 24;15(4):399. doi: 10.3390/ph15040399 (PMC9026320; doi:10.3390/ph15040399)
Supplement: Supplementary file 1 [file pharmaceuticals-15-00399-s001.zip › pharmaceuticals-1632197-supplementary.pdf]

Supplementary Materials

Design, Synthesis, and Cytotoxicity and Topoisomerase I/IIα Inhibition Activity of Pyrazolo[4,3-*f*]quinoline Derivatives

Chhabi Lal Chaudhary [1,†], Seungyun Ko [1,†], Chaerim Lee [1], Yerin Kim [1], Chanhyun Jung [1], Soonsil Hyun [1], Youngjoo Kwon [2], Jong-Soon Kang [3], Jae-Kyung Jung [1] and Heesoon Lee [1,*]

[1] College of Pharmacy and Medicinal Research Center (MRC), Chungbuk National University,
Cheongju 28160, Korea; chhabichaudhary319@gmail.com (C.L.C.);
gozzang05@gmail.com (S.K.); ihl0905@gmail.com (C.L.); ggp02019@naver.com (Y.K.);
cksgus9823@naver.com (C.J.);
shyun@chungbuk.ac.kr (S.H.); orgjkjung@chungbuk.ac.kr (J.-K.J.)
[2] College of Pharmacy, Ewha Womans University, Seoul 03760, Korea;
ykwon@ewha.ac.kr
[3] Korea Research Institute of Bioscience and Biotechnology, Chungbuk 28116, Korea;
kanjon@kribb.re.kr
* Correspondence: medchem@chungbuk.ac.kr; Tel.: +82-43-261-2811; Fax: +82-43-268-2732
† These authors contributed equally to this work.

Table of Contents

S.N.	Contents	Page No.
1	Synthesis	S2
2	Biology	S9
3	References	S10
4	NMR and HRMS spectra	S11

1. Synthesis

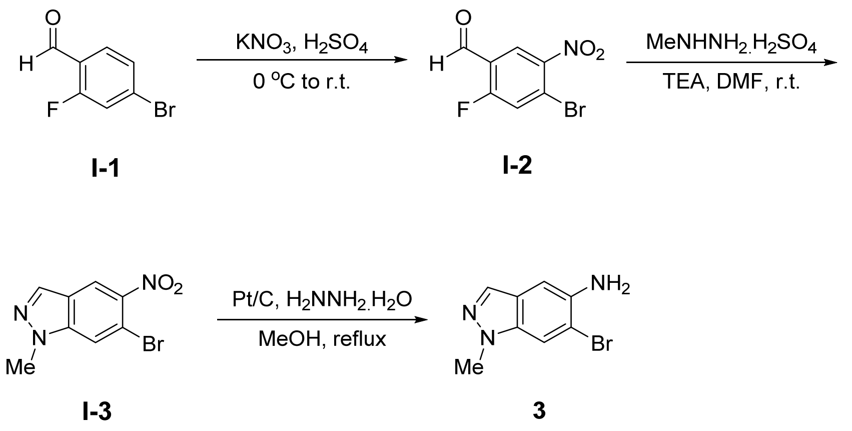

Scheme S1. Synthesis of starting precursor **3**.

4-Bromo-2-fluoro-5-nitrobenzaldehyde (I-2)[31]

As shown in Scheme S1, KNO_3 (1.1 g, 10.84 mmol) in the round bottom flask was added H_2SO_4 (8.2 ml, 153.66 mmol) dropwise at 0 °C under nitrogen atmosphere. To the solution, 4-bromo-2-fluorobenzaldehyde (**I-1**) (2.0 g, 9.85 mmol) was added and stirred at room temperature for 3 h. The reaction mixture was quenched by dropwise addition of ice water. Then, the resulting solid precipitate was filtered, dried, and obtained as a product **I-2**. White solid; 93% yield; ^1H NMR (400 MHz, $CDCl_3$) δ 10.13 (s, 1H), 8.49 (d, J = 6.4 Hz, 1H), 8.22 (d, J = 10.4 Hz, 1H).

6-Bromo-5-nitro-1*H*-indazole (I-3)[33]

To a methylhydrazine sulfate (0.9 g, 6.05 mmol) in DMF (10 ml) was added TEA (1.69 mL, 12.090 mmol) dropwise under nitrogen exchange. The mixture was stirred at room temperature for 1 h. then added **I-2** (1 g, 4.03 mmol) and stirring continued for an additional 1 h. After completion, the reaction was terminated by the addition of water. The mixture was further stirred, and the resulting precipitate was collected by filtration and water washing. The obtained solid was washed again with EtOAc to afford **I-3**. Yellow solid; 93% yield; ^1H NMR (400 MHz, $CDCl_3$): δ 8.38 (s, 1H), 8.13 (s, 1H), 7.77 (s, 1H), 4.11 (s ,3H).

6-Bromo-1-methyl-1*H*-indazol-5-amine (3)[34,35]

To intermediate **I-3** (0.5 g, 1.95 mmol) and platinum on carbon (0.03 g, 0.13 mmol) in MeOH (25 ml) was added hydrazine monohydrate (1 ml, 19.50 mmol) dropwise and refluxed under nitrogen atmosphere for 12 h. The reaction mixture was filtered through a pad of celite. The

filtrate was concentrated and purified by silica gel column chromatography Hex:EtOAc = 3:1) to afford **3** as a starting precursor. Yellow solid; 93% yield; ^{1}H NMR (400 MHz, DMSO-*d*$_6$): δ 7.84 (s, 1H), 7.76 (s, 1H), 7.03 (s, 1H), 4.94 (s, 2H), 3.93 (s, 3H).

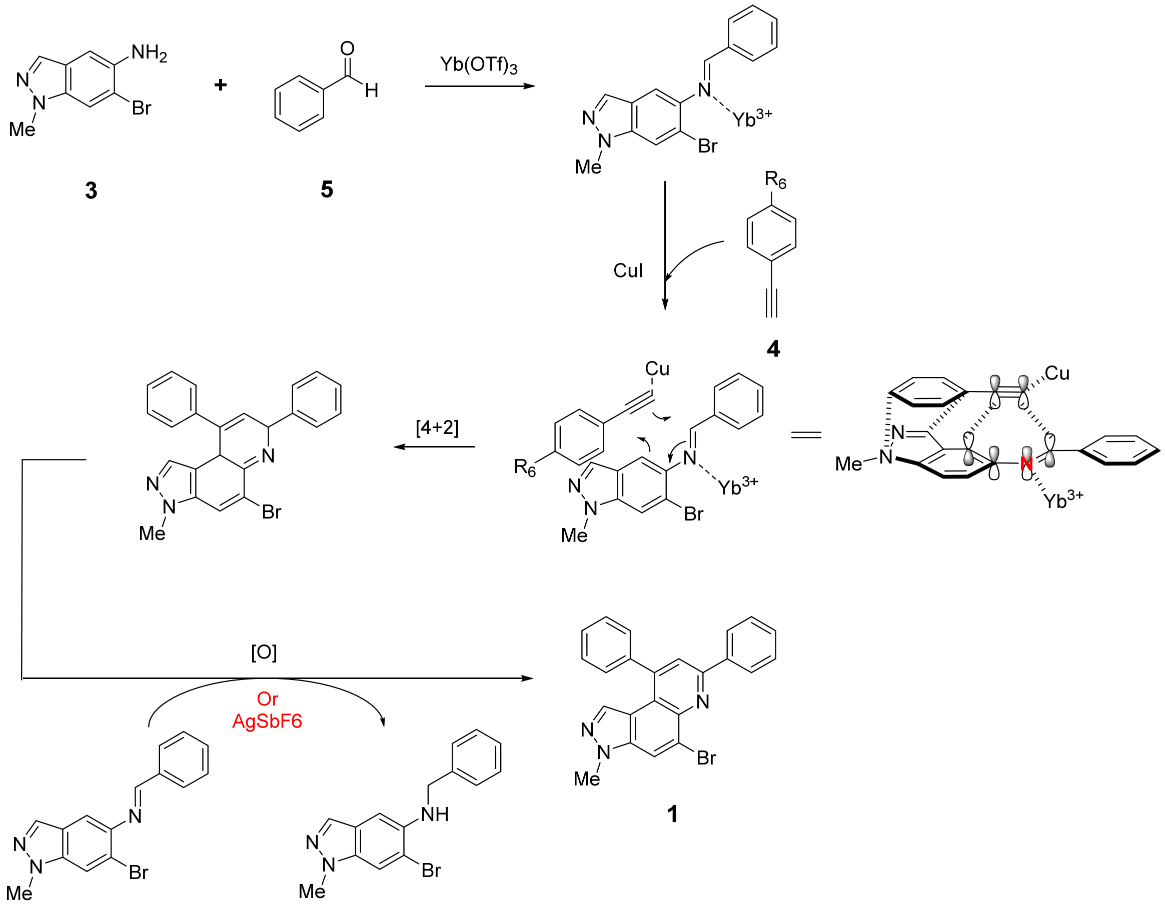

Scheme S2. A plausible mechanism of intermolecular Diels-Alder reaction.

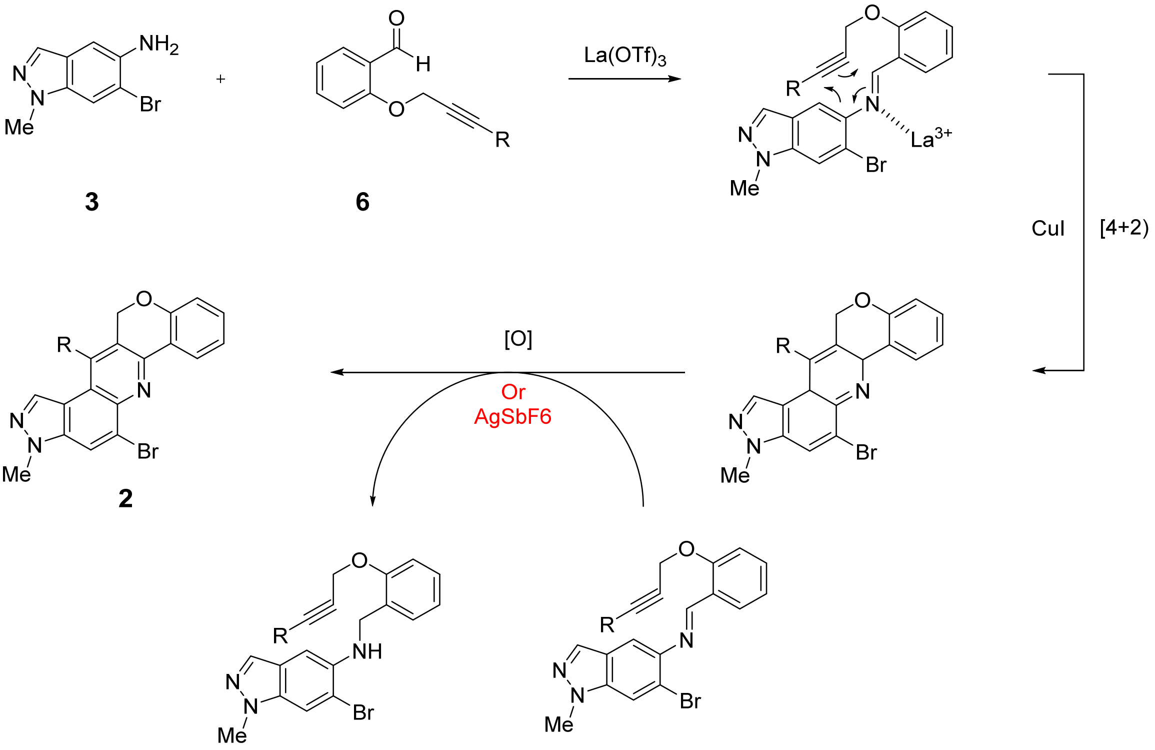

Scheme S3. A plausible mechanism of intramolecular Diels-Alder reaction

General procedure for the synthesis of intermediates[30,32]

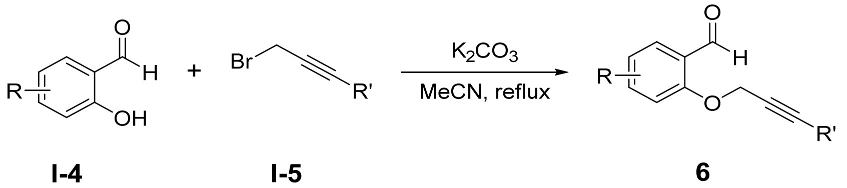

Scheme S4. Representative synthetic method of compounds **6**.

The substituted salicylaldehydes **I-4** (500 mg, 1 eq) and K_2CO_3 (5 eq) in MeCN (10 ml) was added stirred at room temperature for 15 min under nitrogen atmosphere. Then, the substituted propargyl bromides **I-5** (1 eq) was added dropwise. The resulting mixture was reflux for 4 h. After completion, the reaction mixture was filtered off, and the filtrate was concentrated for further purification in silica gel column chromatography (Hex:EtOAc = 5:1) to afford intermediates **6**.

2-(Prop-2-yn-1-yloxy)benzaldehyde (6A) [CAS RN: 29978-83-4]

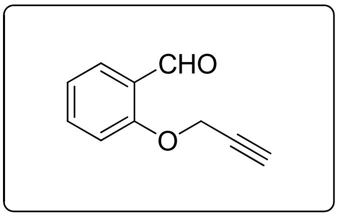

Orange solid; 97% yield; ^{1}H NMR (400 MHz, $CDCl_3$): δ 10.48 (s, 1H), 7.86 (dd, J = 7.6, 1.6 Hz, 1H), 7.57 (ddd, J = 7.6, 7.6, 1.6 Hz, 1H), 7.12 (d, J = 8.8 Hz, 1H), 7.08 (td, J = 8.8, 7.6 Hz, 1H), 4.83 (d, J = 2.4 Hz, 2H), 2.57 (t, J = 2.4 Hz, 1H).

4-Methoxy-2-(prop-2-yn-1-yloxy)benzaldehyde (6B) [CAS RN: 236390-57-1]

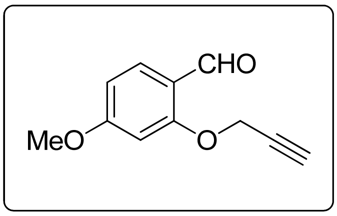

Orange solid; 94% yield; ^{1}H NMR (400 MHz, $CDCl_3$): δ 10.31 (s, 1H), 7.85 (dd, J = 8.0, 2.0 Hz, 1H), 6.62 – 6.60 (m, 2H), 4.81 (d, J = 2.4 Hz, 2H), 2.58 (t, J = 2.4 Hz, 1H).

5-Methoxy-2-(prop-2-yn-1-yloxy)benzaldehyde (6C) [CAS RN: 224317-65-1]

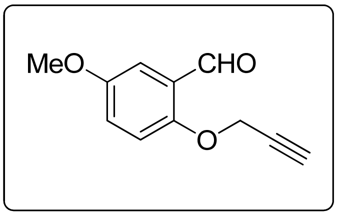

Brown solid; 98% yield; ^{1}H NMR (400 MHz, $CDCl_3$): δ 10. 45 (s, 1H), 7.35 (d, J = 3.2 Hz, 1H), 7.14 (dd, J = 9.2, 3.2 Hz, 1H), 7.08 (d, J = 9.2 Hz, 1H), 4.79 (d, J = 2.4 Hz, 2H), 3.81 (s, 3H), 2.55 (t, J = 2.4 Hz, 1H).

3-Methyl-2-(prop-2-yn-1-yloxy)benzaldehyde (6D) [CAS RN: 1006030-20-1]

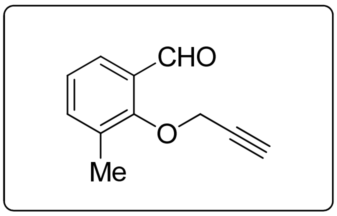

Yellow liquid; 90% yield; ^{1}H NMR (400 MHz, $CDCl_3$): δ 10.42 (s, 1H), 7.71 (d, J = 7.6 Hz, 1H), 7.46 (d, J = 7.6 Hz, 1H), 7.18 (t, J = 7.6 Hz, 1H), 4.68 (d, J = 2.0 Hz, 2H), 2.53 (t, J = 2.0 Hz, 1H), 2.36 (s, 3H).

6-Fluoro-2-(prop-2-yn-1-yloxy)benzaldehyde (6E) [CAS RN: 1692480-05-9]

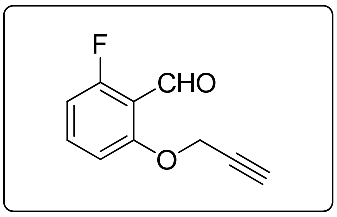

Orange solid; 73% yield; ^{1}H NMR (400 MHz, $CDCl_3$): δ 10.44 (s, 1H), 7.51 (dt, J = 8.4, 6.4 Hz, 1H), 6.92 (d, J = 8.4 Hz, 1H), 6.80 (dd, J = 10.0, 8.4 Hz, 1H), 4.84 (d, J = 2.4 Hz, 2H), 2.58 (t, J = 2.4 Hz, 1H).

5-Fluoro-2-(prop-2-yn-1-yloxy)benzaldehyde (6F) [CAS RN: 1096333-23-1]

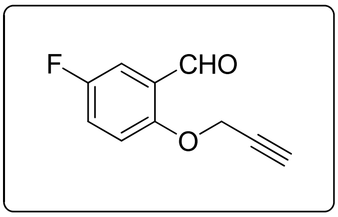

Yellow solid; 96% yield; ^{1}H NMR (400 MHz, $CDCl_3$): δ 10.43 (d, J = 3.2 Hz, 1H), 7.54 (dd, J = 8.4, 3.2 Hz,, 1H), 7.30 – 7.25 (m, 1H), 7.11 (dd, J = 9.2, 4.0 Hz, 1H), 4.82 (d, J = 2.4 Hz, 2H), 2.58 (t, J = 2.8 Hz, 1H).

4-Fluoro-2-(prop-2-yn-1-yloxy)benzaldehyde (6G) [CAS RN: 1279702-70-3]

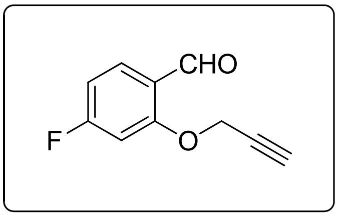

Pale yellow solid; 87% yield; ^{1}H NMR (400 MHz, $CDCl_3$): δ 10.37 (s, 1H), 7.88 (dd, J = 8.4, 6.8 Hz, 1H), 6.84 (dd, J = 10.4, 2.4 Hz, 1H), 6.78 (dd, J = 8.4, 2.4 Hz, 1H), 4.82 (d, J = 2.4 Hz, 2H), 2.61 (t, J = 2.8 Hz, 1H).

3-Fluoro-2-(prop-2-yn-1-yloxy)benzaldehyde (6H) [CAS RN:1271773-10-4]

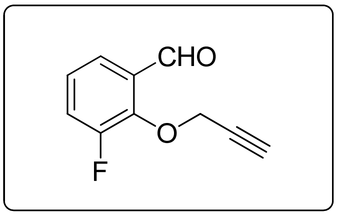

Orange liquid; 92% yield; ^{1}H NMR (400 MHz, $CDCl_3$): δ 10.44 (s, 1H), 7.65 (dt, J = 8.0 Hz, 1H), 7.36 (ddd, J = 11.2, 8.0, 1.6 Hz, 1H), 7.18 (dt, J = 8.0, 4.4 Hz, 1H), 4.91 (d, J = 2.4 Hz, 2H), 2.52 (t, J = 2.4 Hz, 1H).

6-Chloro-2-(prop-2-yn-1-yloxy)benzaldehyde (6I) [CAS RN: 1092306-71-2]

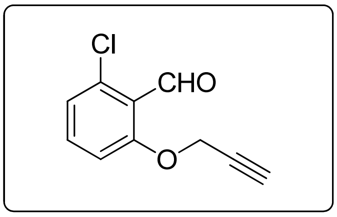

Pale yellow solid; 93% yield; ^{1}H NMR (400 MHz, $CDCl_3$): δ 10. 46 (s, 1H), 7.81 (dd, J = 8.0, 1.6 Hz, 1H), 7.65 (d, J = 8.0, 1.6 Hz, 1H), 7.24 (t, J = 8.0 Hz, 1H), 4.90 (d, J = 2.4 Hz, 2H), 2.54 (t, J = 2.4 Hz, 1H).

5-Chloro-2-(prop-2-yn-1-yloxy)benzaldehyde (6J) [CAS RN: 224317-64-0]

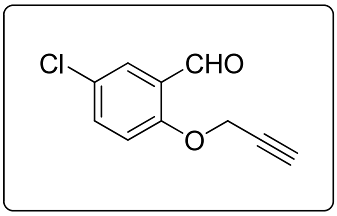

Pale yellow solid; 90% yield; ^{1}H NMR (400 MHz, $CDCl_3$): δ 10.40 (s, 1H), 7.80 (d, J = 2.8 Hz, 1H), 7.51 (dd, J = 8.4, 2.8 Hz, 1H), 7.09 (d, J = 8.4 Hz, 1H), 4.83 (d, J = 2.8 Hz, 2H), 2.59 (t, J = 2.8 Hz, 1H).

4-Chloro-2-(prop-2-yn-1-yloxy)benzaldehyde (6K) [CAS RN: 1595918-82-3]

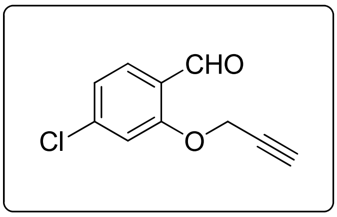

Pale yellow solid; 96% yield; ^{1}H NMR (400 MHz, CDCl3): δ 10. 39 (s, 1H), 7.79 (d, J = 8.0 Hz, 1H), 7.12 (d, J = 1.2 Hz, 1H), 7.08 – 7.05 (m 1H), 4.82 (d, J = 2.4 Hz, 2H), 2.62 (t, J = 2.4 Hz, 1H).

3-Chloro-2-(prop-2-yn-1-yloxy)benzaldehyde (6L) [CAS RN: 1271773-06-8]

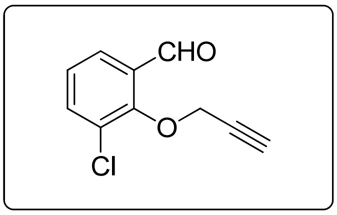

Pale yellow solid; 93% yield; ^{1}H NMR (400 MHz, CDCl3): δ 10.51 (s, 1H), 7.43 (t, J = 8.4 Hz, 1H), 7.10 (d, J = 8.4 Hz, 1H), 7.06 (d, J = 8.4 Hz, 1H), 4.82 (d, J = 2.4 Hz, 2H), 2.56 (t, J = 2.4 Hz, 1H).

3,5-Dichloro-2-(prop-2-yn-1-yloxy)benzaldehyde (6M) [CAS RN: 1126527-53-4]

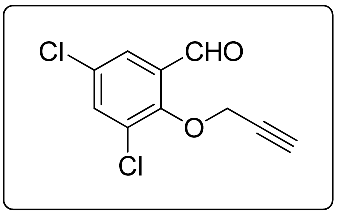

Pale yellow solid; 94% yield; ^{1}H NMR (400 MHz, CDCl3): δ 10.39 (s, 1H), 7.75 (d, J = 2.8 Hz, 1H), 7.64 (d, J = 2.8 Hz, 1H), 4.89 (d, J = 2.4 Hz, 2H), 2.55 (t, J = 2.4 Hz, 1H).

5-Bromo-2-(prop-2-yn-1-yloxy)benzaldehyde (6N) [CAS RN: 122835-14-7]

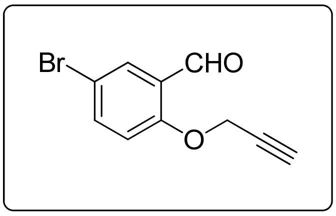

Pale yellow solid; 94% yield; ^{1}H NMR (400 MHz, CDCl3): δ 10.39 (s, 1H), 7.95 (d, J = 2.4 Hz, 1H), 7.65 (dd, J = 9.2, 2.4 Hz, 1H), 7.03 (d, J = 9.2 Hz, 1H), 4.82 (d, J = 2.4 Hz, 2H), 2.59 (t, J = 2.4 Hz, 1H).

4-Bromo-2-(prop-2-yn-1-yloxy)benzaldehyde (6O) [CAS RN: 1099609-05-8]

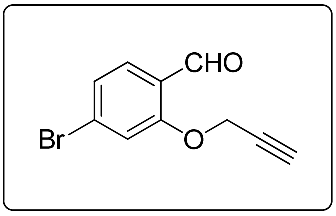

Pale yellow solid; 93% yield; ^{1}H NMR (400 MHz, CDCl3): δ 10.41 (s, 1H), 7.72 (d, J = 8.8 Hz, 1H), 7.29 (d, J = 1.2 Hz, 1H), 7.26 – 7.23 (m, 1H), 4.83 (d, J = 2.0 Hz, 2H), 2.62 (t, J = 2.0 Hz, 1H).

3-Bromo-2-(prop-2-yn-1-yloxy)benzaldehyde (6P) [CAS RN: 1271773-00-2]

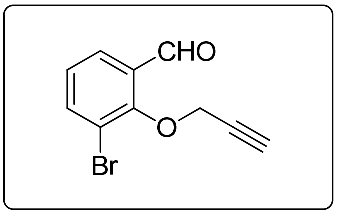

Pale yellow solid; 75% yield; ^{1}H NMR (400 MHz, $CDCl_3$): δ 10.45 (s, 1H), 7.85 (dd, J = 8.0, 1.6 Hz, 1H), 7.82 (dd, J = 8.0, 1.6 Hz, 1H), 7.18 (t, J = 8.0 Hz, 1H), 4.89 (d, J = 2.4 Hz, 2H), 2.55 (t, J = 2.4 Hz, 1H).

1-(Prop-2-yn-1-yloxy)-2-naphthaldehyde (6Q) [CAS RN: 1271773-34-2]

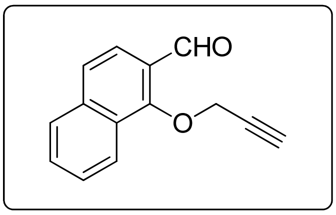

Orange solid; 82% yield; ^{1}H NMR (400 MHz, $CDCl_3$): δ 10.66 (s, 1H), 8.23 (d, J = 8.4 Hz, 1H), 7.90 (d, J = 8.0 Hz, 1H), 7.89 (d, J = 8.0 Hz, 1H), 7.71 (d, J = 8.4 Hz, 1H), 7.65 (td, J = 8.0, 1.6 Hz, 1H), 7.60 (td, J = 8.0, 1.6 Hz, 1H), 4.94 (d, J = 2.4 Hz, 2H), 2.61 (t, J = 2.4 Hz, 1H).

2-(Prop-2-yn-1-yloxy)-1-naphthaldehyde (6R) [CAS RN: 58758-48-8]

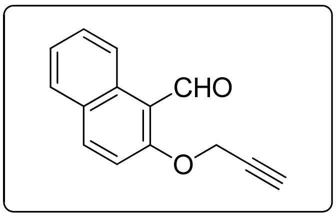

Brown solid; 87% yield; ^{1}H NMR (400 MHz, $CDCl_3$): δ 10.90 (s, 1H), 9.28 (d, J = 9.2 Hz, 1H), 8.06 (d, J = 8.8 Hz, 1H), 7.78 (d, J = 8.0 Hz, 1H), 7.63 (ddd, J = 8.8, 6.8, 1.2 Hz, 1H), 7.45 (ddd, J = 8.0, 6.8, 1.2 Hz, 1H), 7.37 (d, J = 9.2 Hz, 1H), 4.93 (d, J = 2.4 Hz, 2H), 2.58 (t, J = 2.4 Hz, 1H).

5-Allyl-3-methoxy-2-(prop-2-yn-1-yloxy)benzaldehyde (6S)

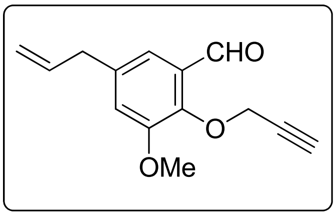

Brown liquid; 99% yield; ^{1}H NMR (400 MHz, $CDCl_3$): δ 10.47 (s, 1H), 7.29 (d, J = 2.4 Hz, 1H), 6.98 (d, J = 2.4 Hz, 1H), 5.99 – 5.89 (m, 1H), 5.14 – 5.08 (m, 2H), 4.85 (d, J = 2.4 Hz, 2H), 3.90 (s, 3H), 3.39 (d, J = 6.4 Hz, 1H), 2.47 (t, J = 2.4 Hz, 1H).

2-(But-2-yn-1-yloxy)-1-naphthaldehyde (6T)

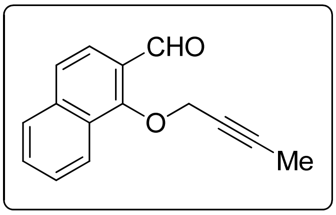

Brown solid; 87% yield; ^{1}H NMR (400 MHz, $CDCl_3$): δ 10. 90 (s, 1H), 9.29 (d, J = 8.4 Hz, 1H), 8.06 (d, J = 9.2 Hz, 1H), 7.79 (d, J = 7.2 Hz, 1H), 7.63 (ddd, J = 8.4, 8.0, 1.2 Hz, 1H), 7.44 (ddd, J = 8.0, 7.2, 1.2 Hz, 1H), 7.39 (d, J = 9.2 Hz, 1H), 4.90 (q, J = 2.4 Hz, 2H), 1.85 (t, J = 2.4 Hz, 3H).

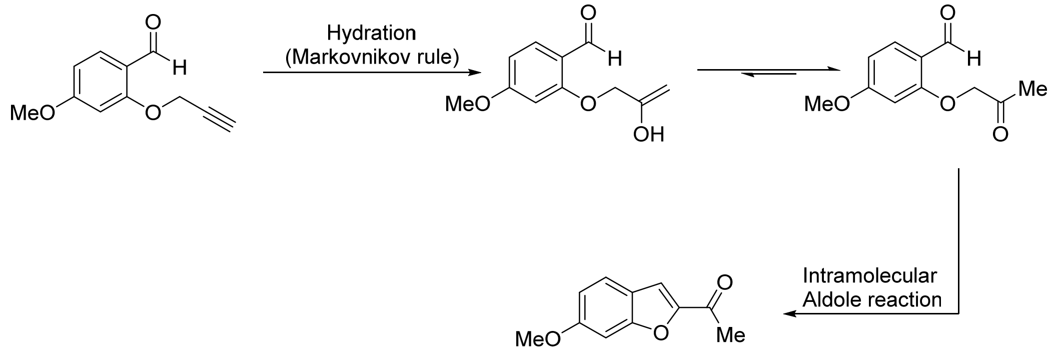

Scheme S5. A plausible mechanism of benzofuran formation.

1-(6-Methoxybenzofuran-2-yl)ethenone [CAS RN: 52814-92-3]

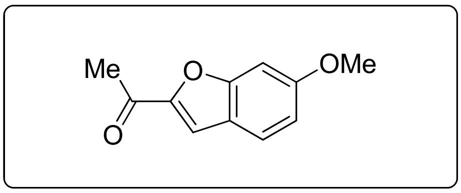

Brown solid; 27% yield; ^{1}H NMR ($CDCl_3$, 400 MHz): δ 7.56 (d, J = 8.8 Hz, 1H), 7.45 (s, 1H), 7.05 (d, J = 2.4 Hz, 1H), 6.95 (dd, J = 8.8, 2.4 Hz, 1H), 3.88 (s, 3H), 2.56 (s, 3H); ^{13}C NMR (100 MHz, $CDCl_3$): δ 188.04, 161.27, 157.44, 152.46, 123.73, 120.48, 114.55, 113.90, 95.73, 55.87, 26.34.

2. Biology

In vitro cytotoxicity assay[38]

The cytotoxicity was performed using sulforhodamine B (SRB) assay. Six human cancer cell lines, including ACHN (ATCC, CRL-1611), HCT-15 (ATCC, CCL-225), MDA-MB-231 (ATCC, HTB-26), NCI-H23 (ATCC, CRL-5800), NUGC-3 (JCRB Cell Bank, JCRB0822), and PC-3 (ATCC, CRL-1435, 5 x 10^3 cells/well), were seeded in 96-well plates (5 x 10^3 cells/well), incubated overnight and treated with different concentrations of compounds for 48 h. Cells were fixed using 50% trichloroacetic acid for 1 hr at 4°C and stained with 0.4% SRB solution for 30 min. The unbound dye was removed by rinsing with 1% acetic acid, the stain was extracted using 10 mM Tris and the absorbance at 540 nm was measured using Versamax microplate reader (Molecular Devices, San Jose, CA, USA). GI_{50} values were calculated using GraphPad Prism (GraphPad Software Inc., San Diego, CA, USA).

In vitro DNA topoisomerase I or IIα relaxation assay[39]

A mixture comprising of 100 ng supercoiled pBR322 plasmid DNA (Thermo Scientific, USA) and 0.2~1 unit of recombinant human DNA topo I (TopoGEN INC., USA) or topo IIα (USB Corp., USA) was incubated with or without the prepared compounds in the assay buffer (For

topo I, 10 mM Tris-HCl (pH 7.9), 150 mM NaCl and 0.1% BSA, 0.1 mM spermidine and 5% glycerol; for topo II, 10 mM Tris-HCl (pH 7.9), 50 mM NaCl, 50 mM KCl, 5 mM MgCl2, 1 mM EDTA, 1 mM ATP and 15 mg/mL BSA) for 30 min at 37 °C. The reaction with a final volume of 10 μL was stopped by adding the topo stop buffer (For topo I, 10% SDS solution containing 0.2% bromophenol blue, 0.2% xylene cyanol and 30% glycerol; for topo II, 7 mM EDTA). The reaction products were electrophoresed on 0.8% agarose gel at 50 V for 1 h with TAE electrophoresis buffer. The gels were stained in an EtBr solution (0.5 mg/mL) and visualized by transillumination with UV light and were quantitated using Alpha Tech ImagerTM (Alpha Innotech Corp., USA).

4. NMR and HRMS spectra

4-Bromo-2-fluoro-5-nitrobenzaldehyde (**I-2**)

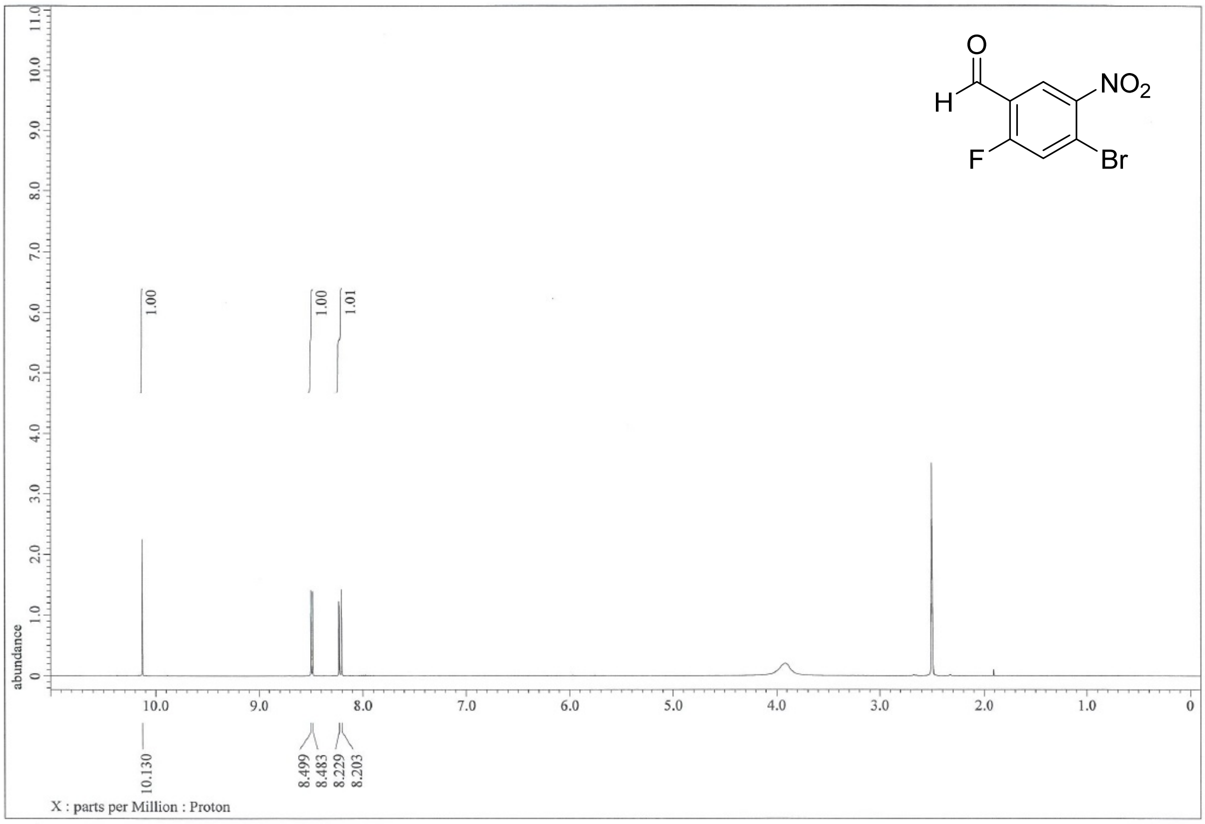

6-Bromo-5-nitro-1*H*-indazole (**I-3**)

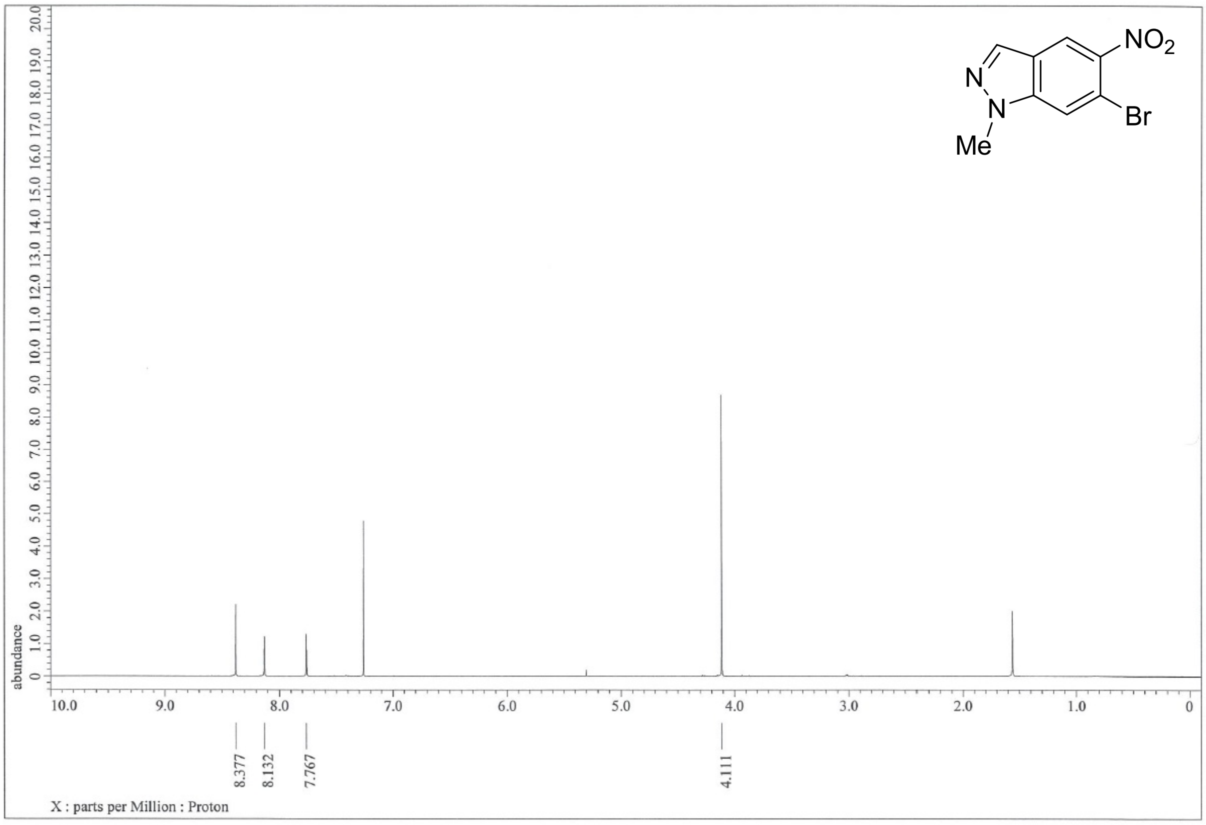

6-Bromo-1-methyl-1*H*-indazol-5-amine (**3**)

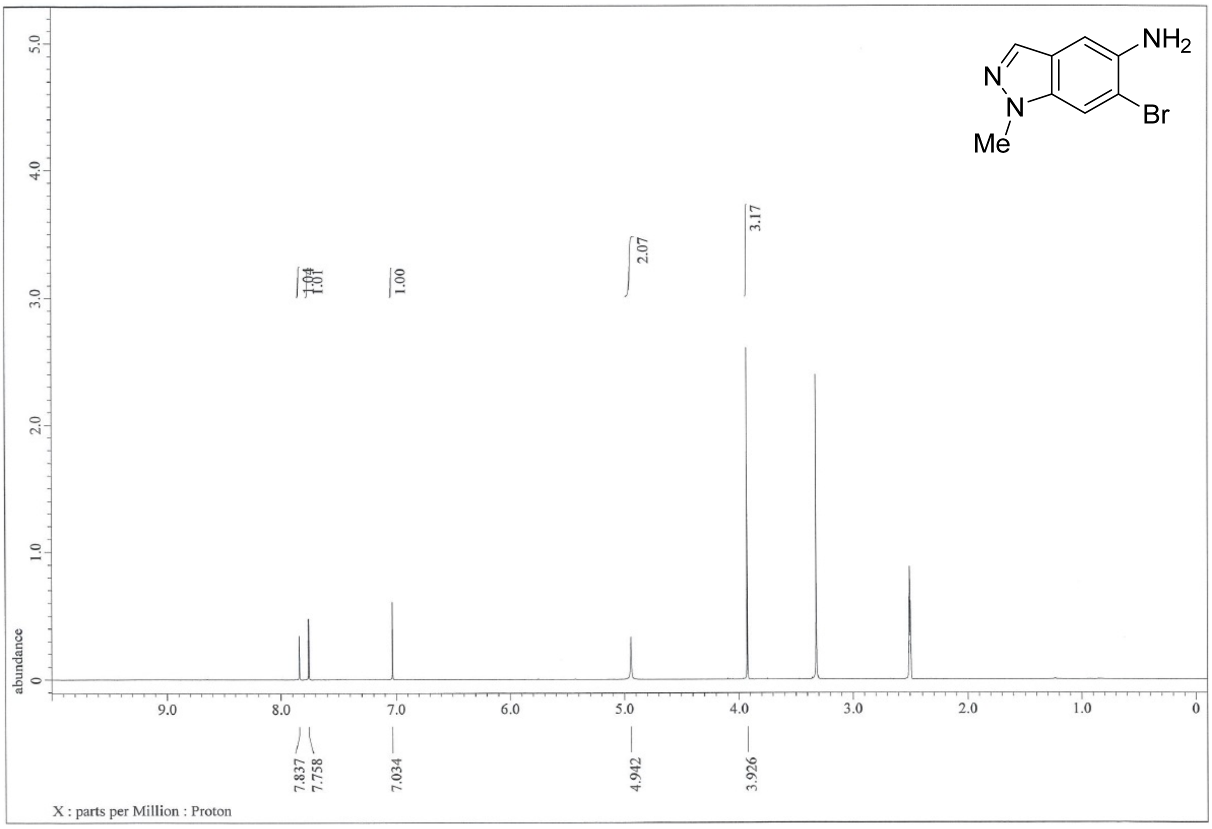

5-Bromo-3-methyl-7-phenyl-9-(*p*-tolyl)-3*H*-pyrazolo[4,3-*f*]quinoline (**1A**)

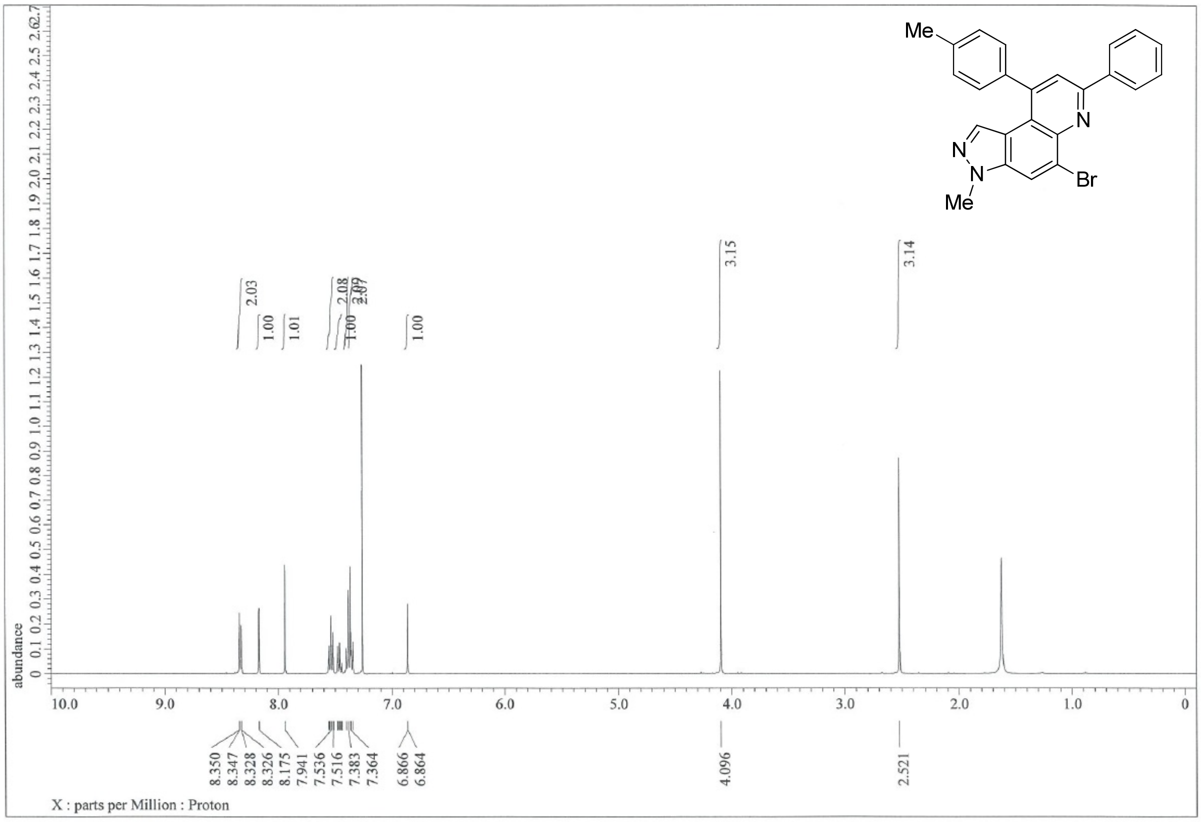

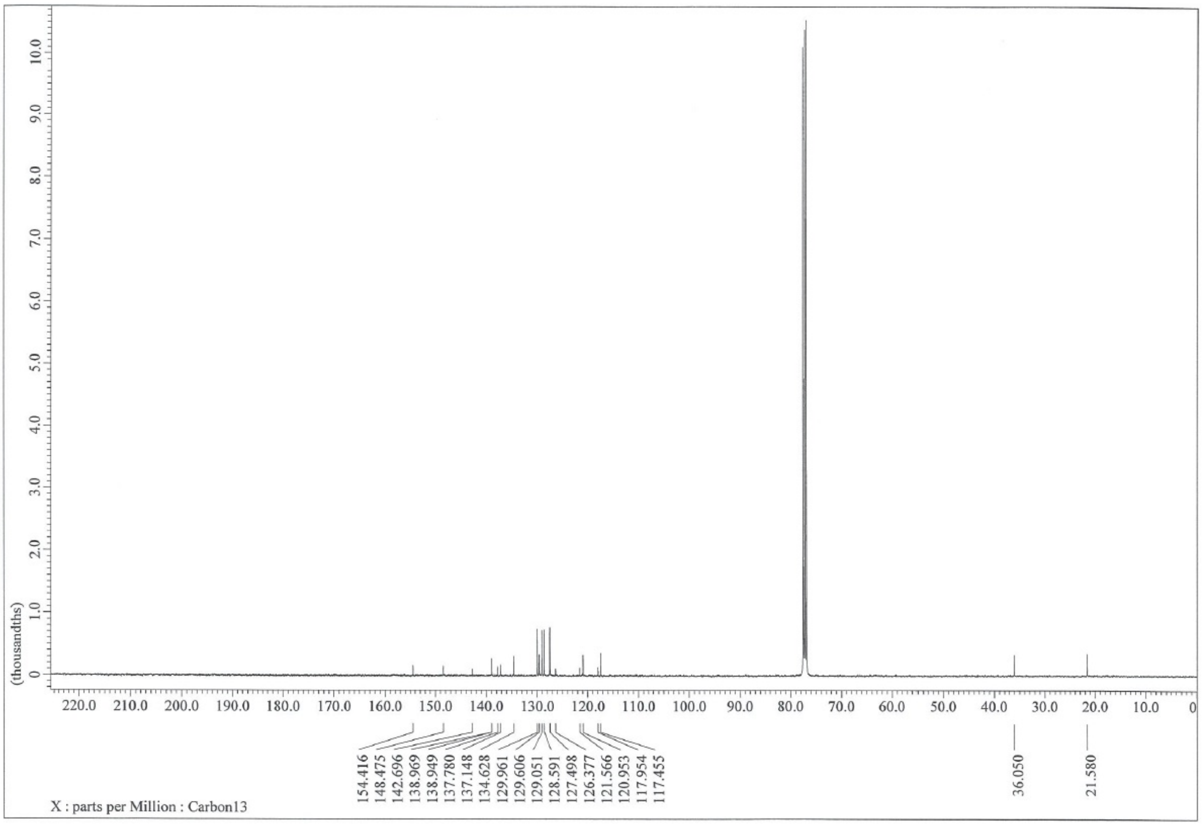

(thousandths)
0
1.0
2.0
3.0
4.0
5.0
6.0
7.0
8.0
9.0
10.0
220.0 210.0 200.0 190.0 180.0 170.0 160.0 150.0 140.0 130.0 120.0 110.0 100.0 90.0 80.0 70.0 60.0 50.0 40.0 30.0 20.0 10.0 0
154.416
148.475
142.696
138.969
138.949
137.780
137.148
134.628
129.961
129.606
129.051
128.591
127.498
126.377
121.566
120.953
117.954
117.455
36.050
21.580
X : parts per Million : Carbon13

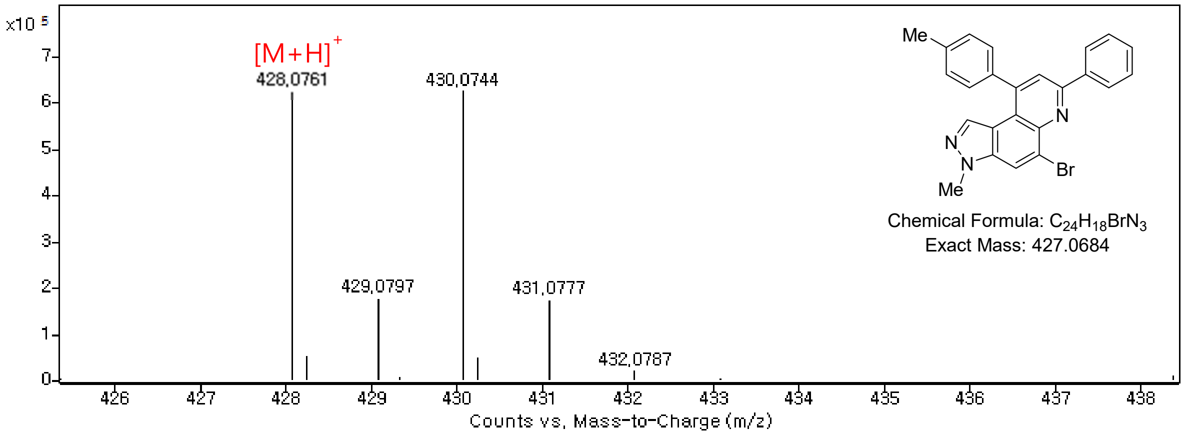
x10 5
[M+H]+
428.0761
430.0744
429.0797
431.0777
432.0787
Counts vs. Mass-to-Charge (m/z)
Me
N
N
N
Me
Br
Chemical Formula: C24H18BrN3
Exact Mass: 427.0684

5-Bromo-3-methyl-7-(pyridin-3-yl)-9-(*p*-tolyl)-3*H*-pyrazolo[4,3-*f*]quinoline (**1B**)

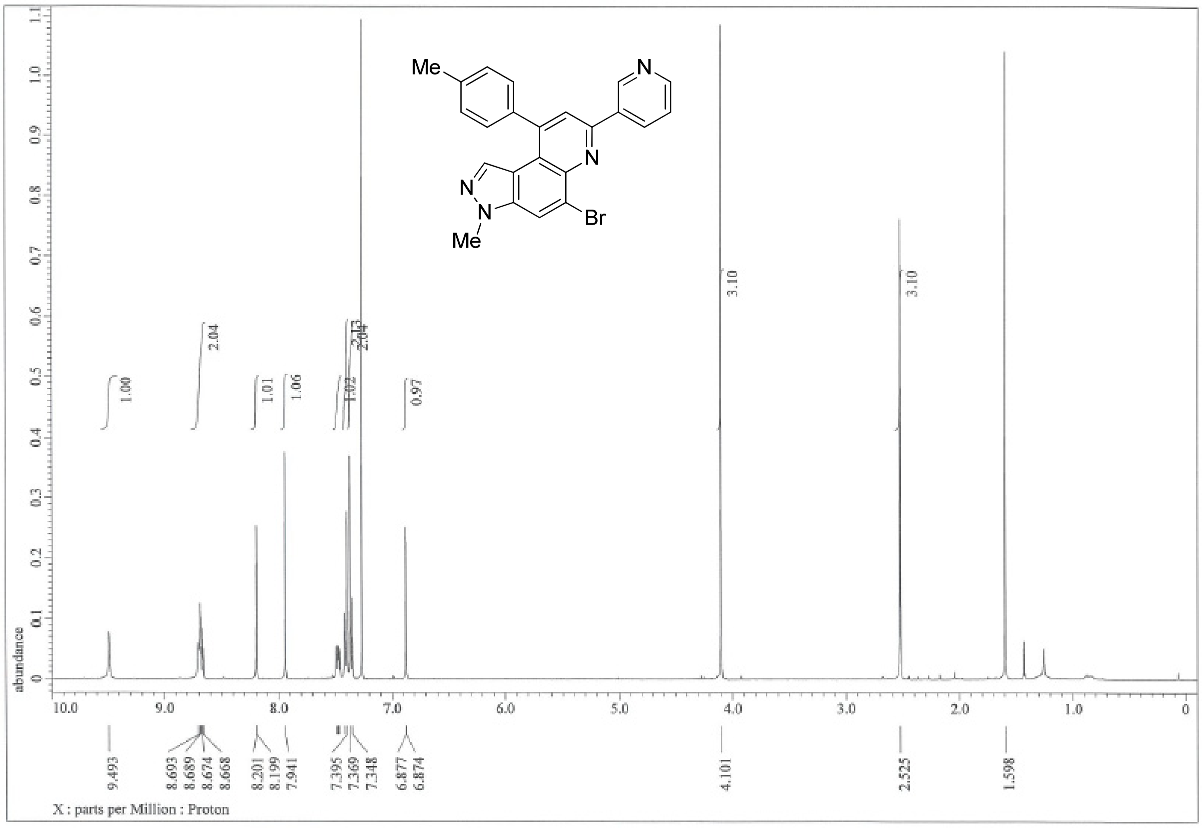

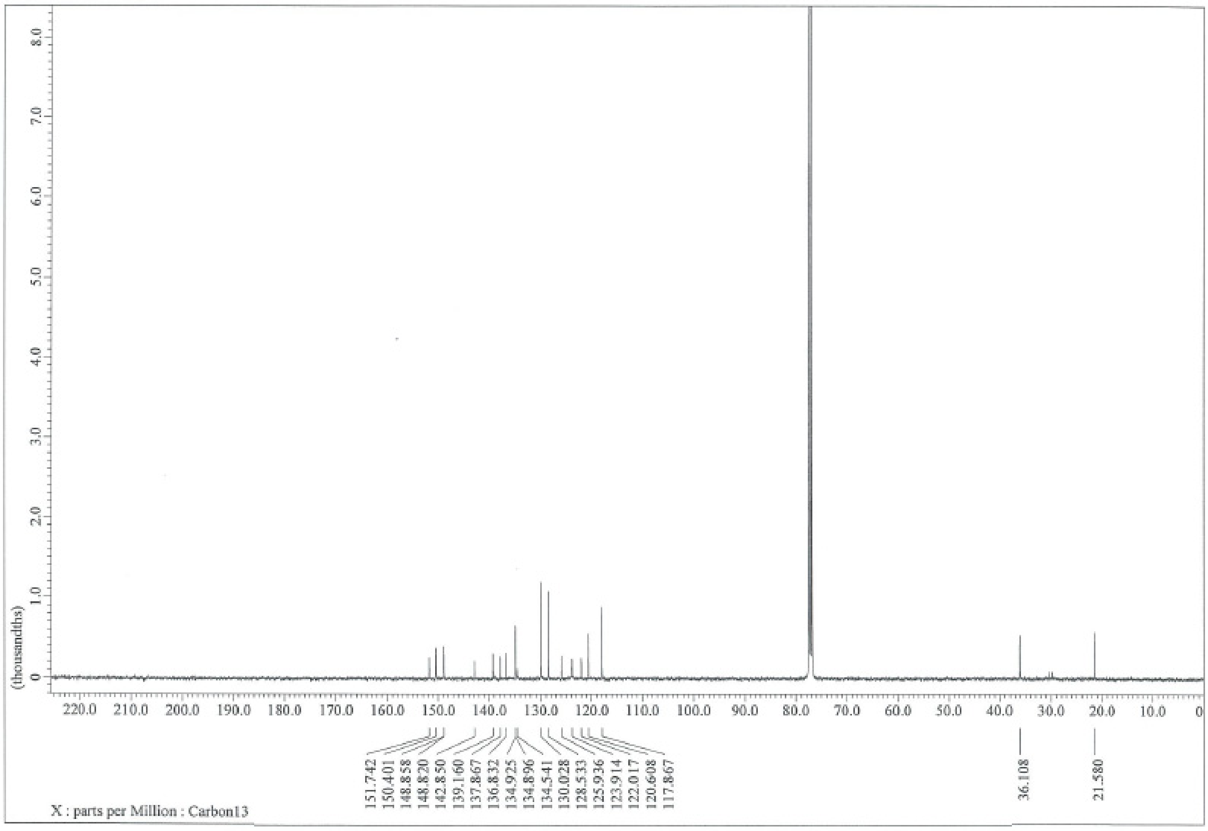

(thousandths)
8.0
7.0
6.0
5.0
4.0
3.0
2.0
1.0
0
220.0 210.0 200.0 190.0 180.0 170.0 160.0 150.0 140.0 130.0 120.0 110.0 100.0 90.0 80.0 70.0 60.0 50.0 40.0 30.0 20.0 10.0 0
151.742
150.401
148.858
148.820
142.850
139.160
137.867
136.832
134.925
134.896
134.541
130.028
128.533
125.936
123.914
122.017
120.608
117.867
36.108
21.580
X : parts per Million : Carbon13

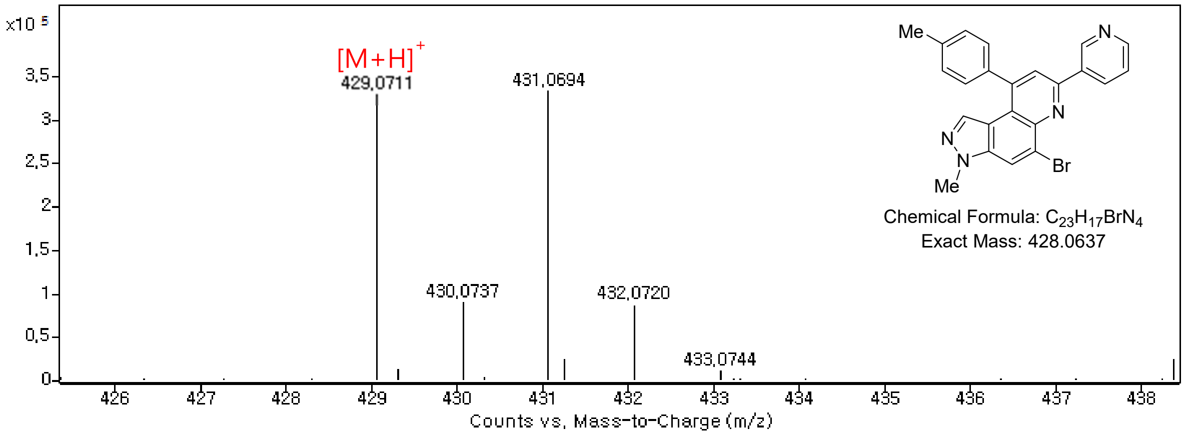

x10 5
[M+H]+
429,0711
431,0694
430,0737
432,0720
433,0744
3,5
3
2,5
2
1,5
1
0,5
0
426
427
428
429
430
431
432
433
434
435
436
437
438
Counts vs. Mass-to-Charge (m/z)
Me
N
N
N
N
Br
Me
Chemical Formula: C23H17BrN4
Exact Mass: 428.0637

3-(5-Bromo-3-methyl-9-(*p*-tolyl)-3*H*-pyrazolo[4,3-*f*]quinolin-7-yl)benzonitrile (**1C**)

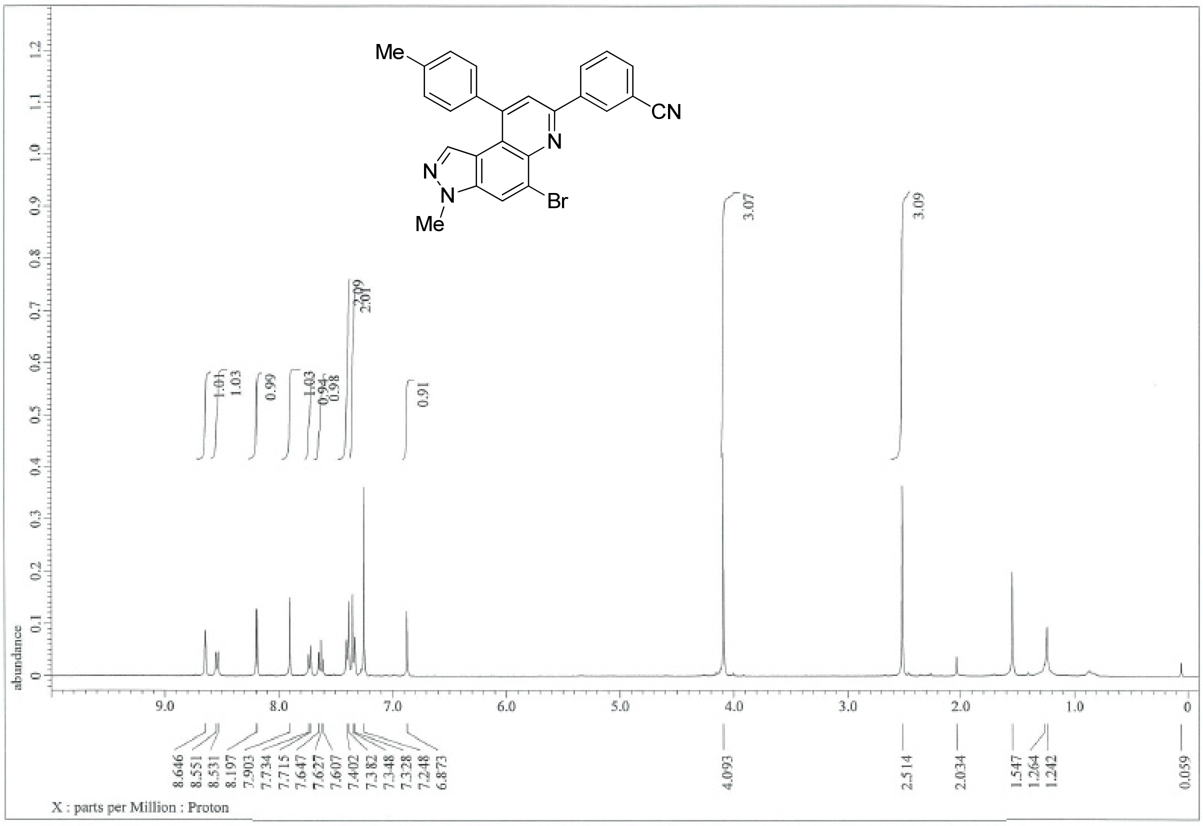

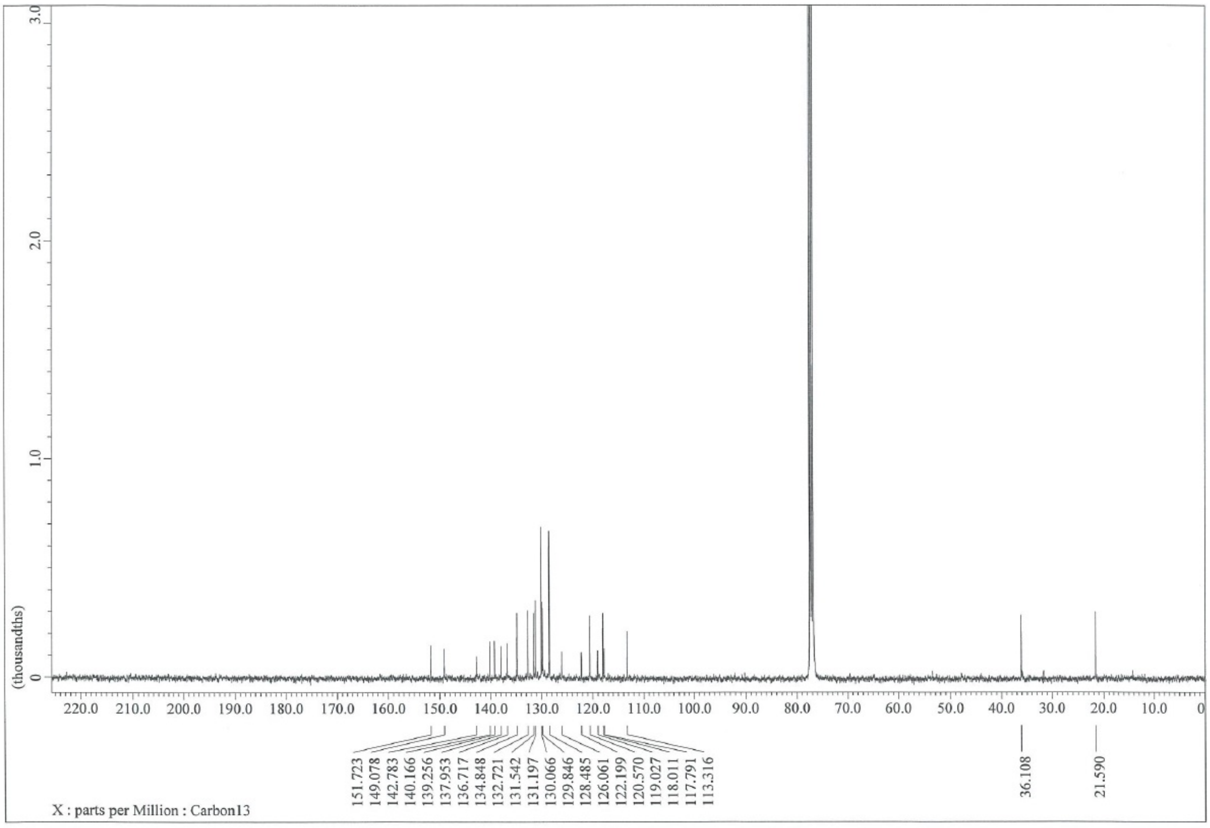
(thousandths)
3.0
2.0
1.0
0
220.0 210.0 200.0 190.0 180.0 170.0 160.0 150.0 140.0 130.0 120.0 110.0 100.0 90.0 80.0 70.0 60.0 50.0 40.0 30.0 20.0 10.0 0
151.723
149.078
142.783
140.166
139.256
137.953
136.717
134.848
132.721
131.542
131.197
130.066
129.846
128.485
126.061
122.199
120.570
119.027
118.011
117.791
113.316
36.108
21.590
X : parts per Million : Carbon13

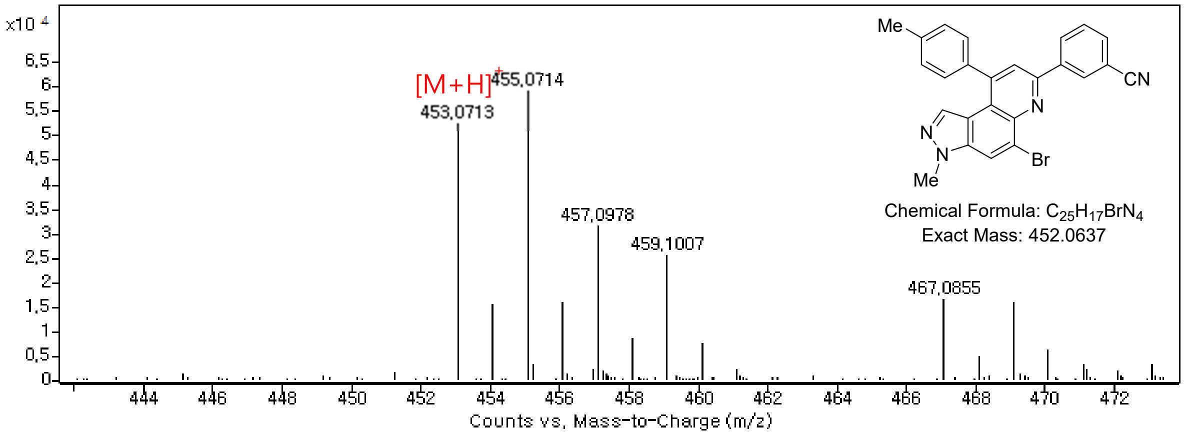

x10 4
[M+H]+
453,0713
455,0714
457,0978
459,1007
467,0855
Counts vs. Mass-to-Charge (m/z)
Me
CN
N
N
N
Br
Me
Chemical Formula: C25H17BrN4
Exact Mass: 452.0637

5-Bromo-3-methyl-9-(*p*-tolyl)-7-(4-(trifluoromethyl)phenyl)-3*H*-pyrazolo[4,3-*f*]quinoline (**1D**)

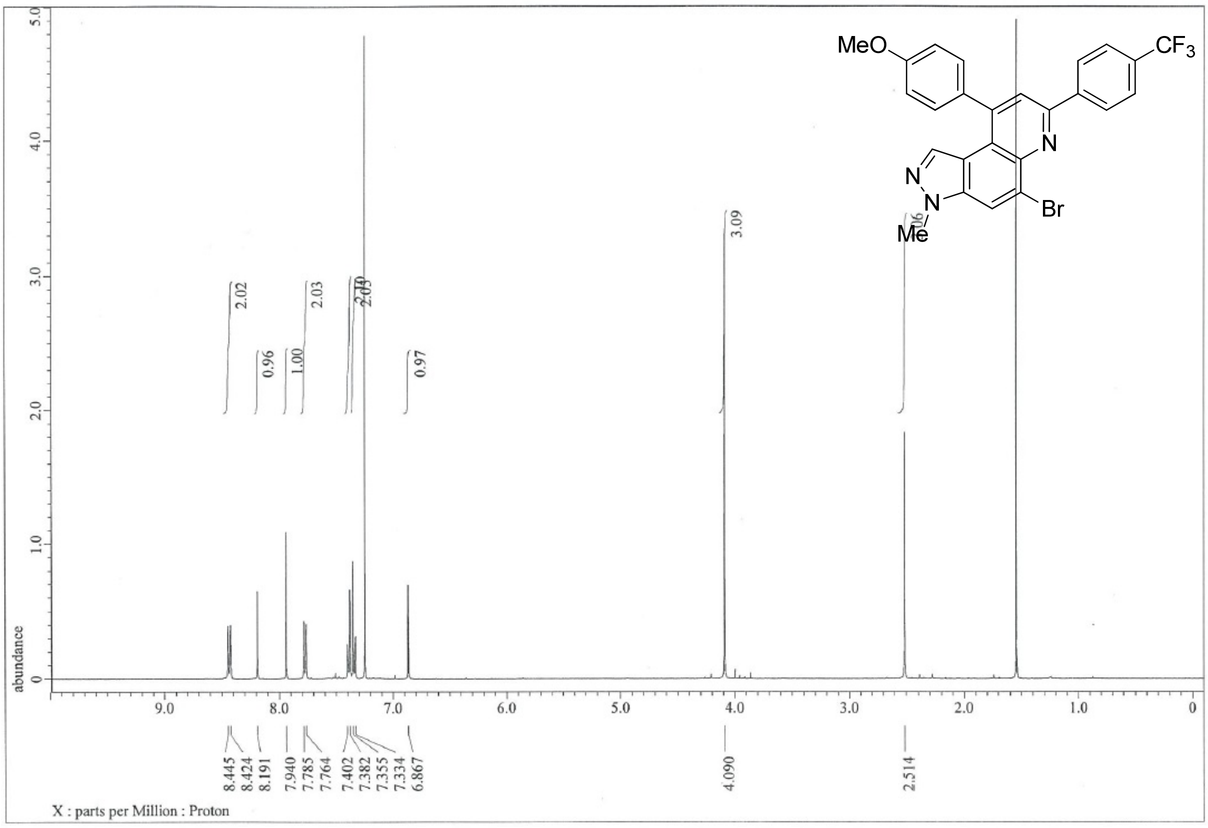

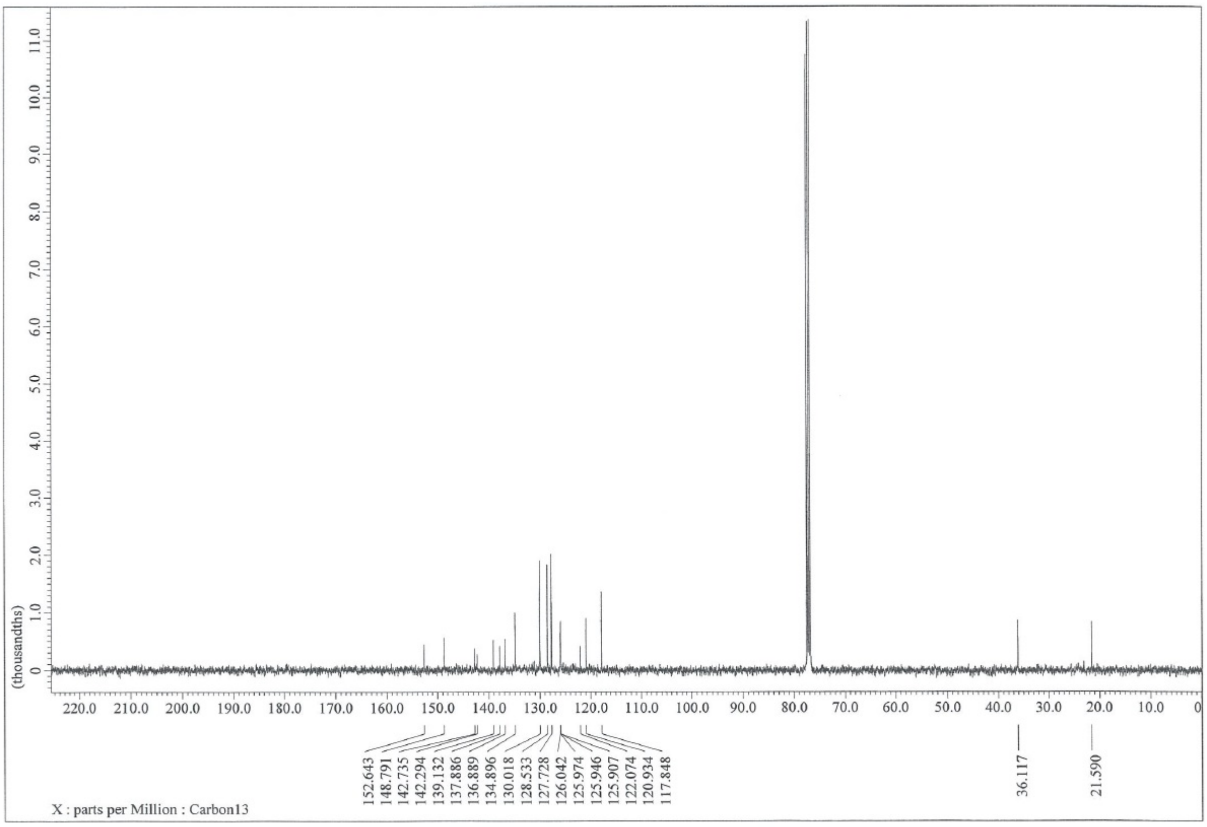
(thousandths)
X : parts per Million : Carbon13
152.643
148.791
142.735
142.294
139.132
137.886
136.889
134.896
130.018
128.533
127.728
126.042
125.974
125.946
125.907
122.074
120.934
117.848
36.117
21.590

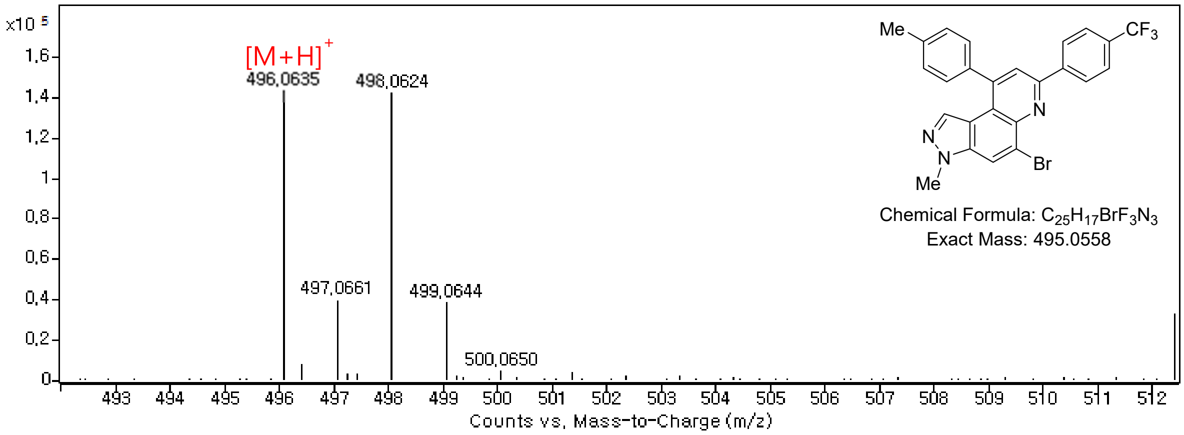

x10 5
[M+H]+
496.0635
498.0624
497.0661
499.0644
500.0650
Counts vs. Mass-to-Charge (m/z)
Me
CF3
N
N
N
Me
Br
Chemical Formula: C25H17BrF3N3
Exact Mass: 495.0558

5-Bromo-3-methyl-7-(4-nitrophenyl)-9-(*p*-tolyl)-3*H*-pyrazolo[4,3-*f*]quinoline (**1E**)

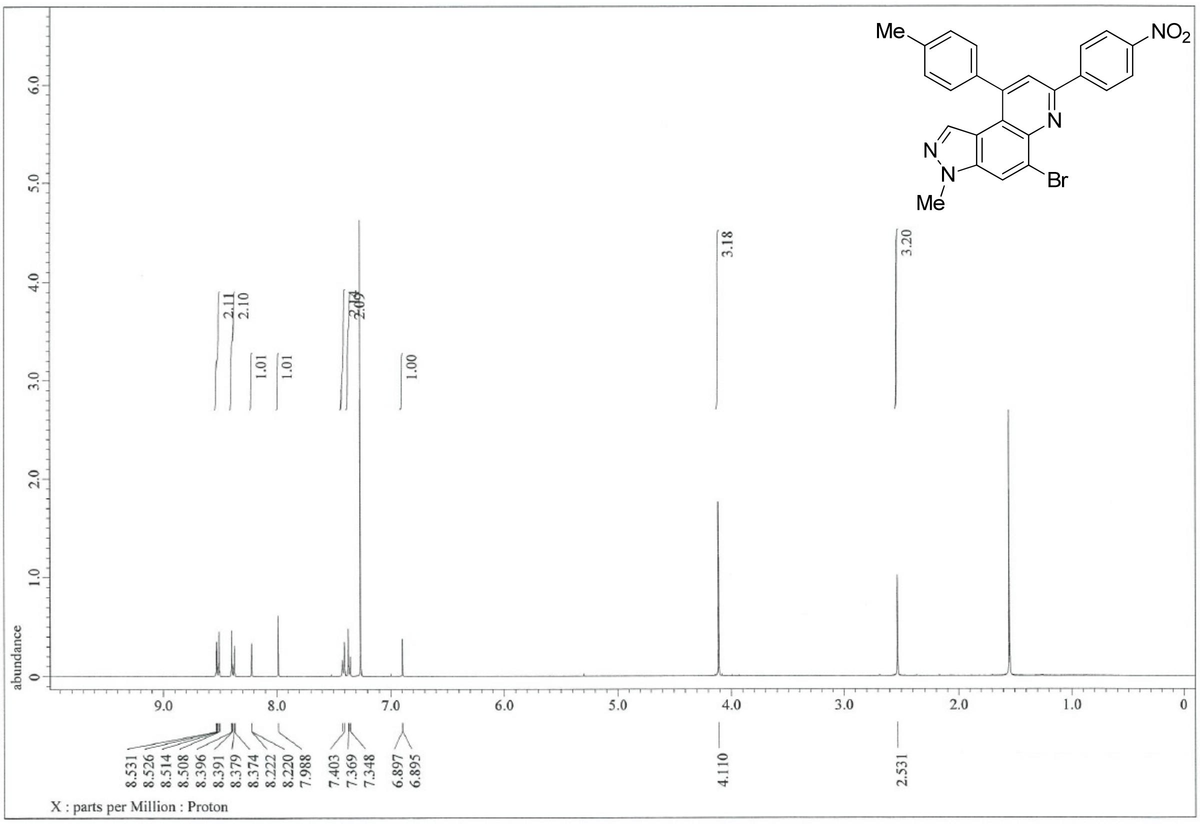

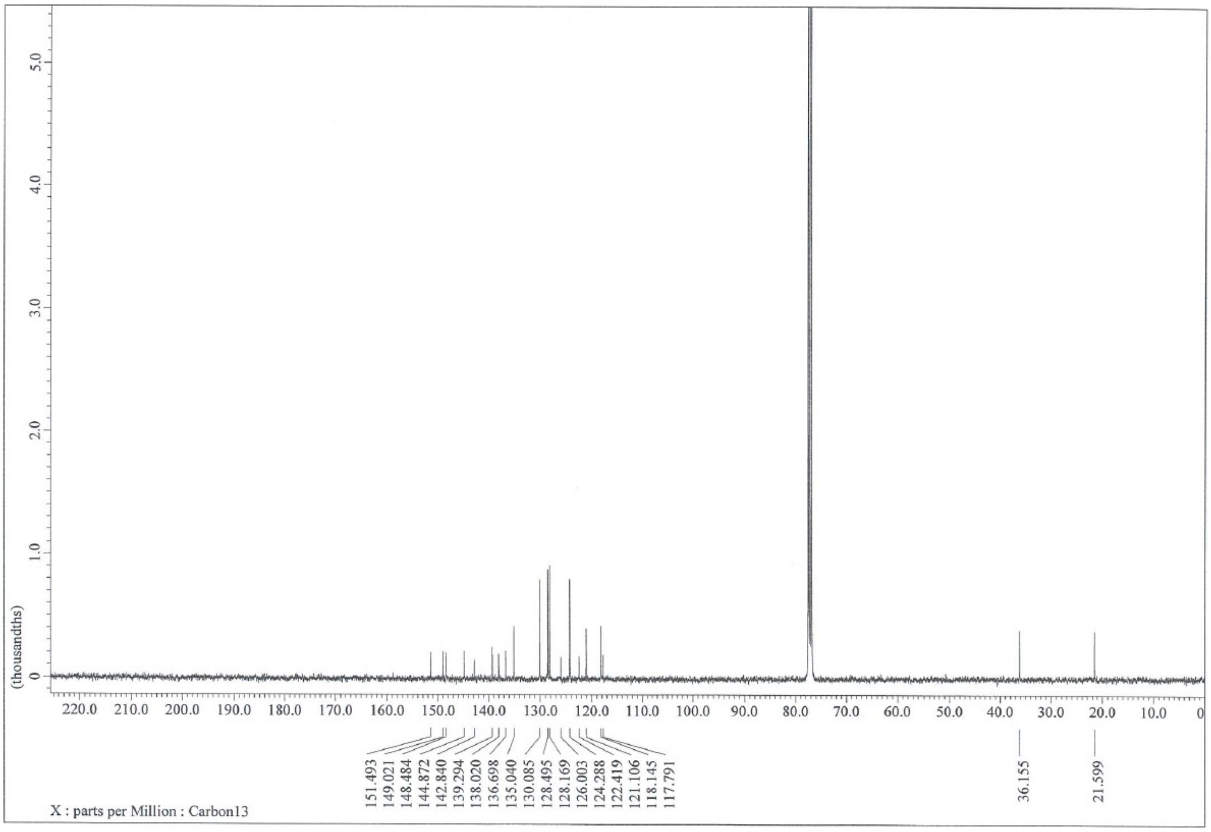
(thousandths)
X : parts per Million : Carbon13
151.493
149.021
148.484
144.872
142.840
139.294
138.020
136.698
135.040
130.085
128.495
128.169
126.003
124.288
122.419
121.106
118.145
117.791
36.155
21.599

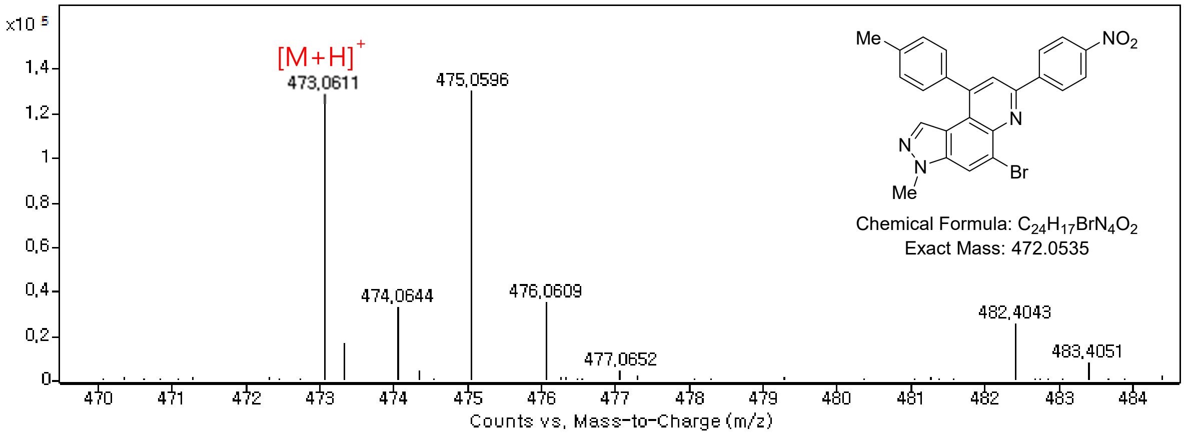
x10 5
1,4
1,2
1
0,8
0,6
0,4
0,2
0
[M+H]+
473,0611
475,0596
474,0644
476,0609
477,0652
482,4043
483,4051
470
471
472
473
474
475
476
477
478
479
480
481
482
483
484
Counts vs. Mass-to-Charge (m/z)
Me
NO2
N
N
N
Br
Me
Chemical Formula: C24H17BrN4O2
Exact Mass: 472.0535

5-Bromo-7-(2-chlorophenyl)-3-methyl-9-(*p*-tolyl)-3H-pyrazolo[4,3-*f*]quinoline (**1F**)

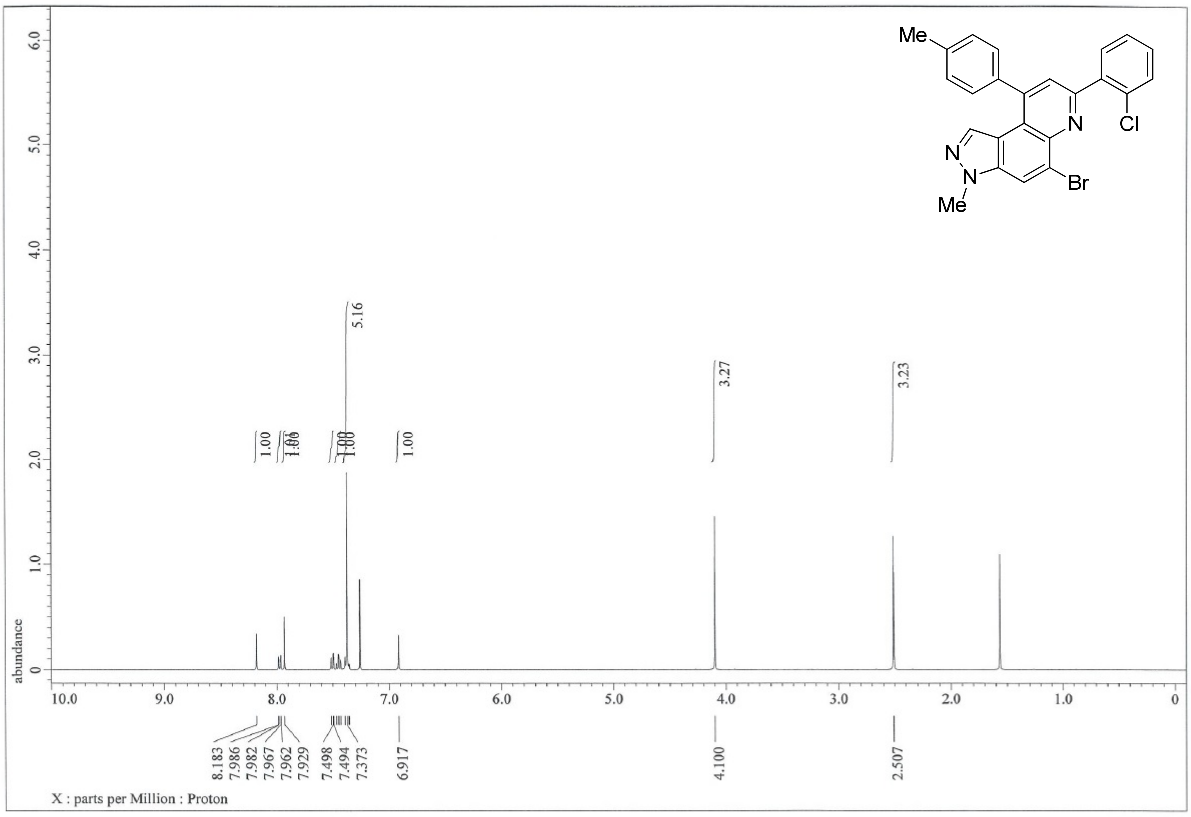

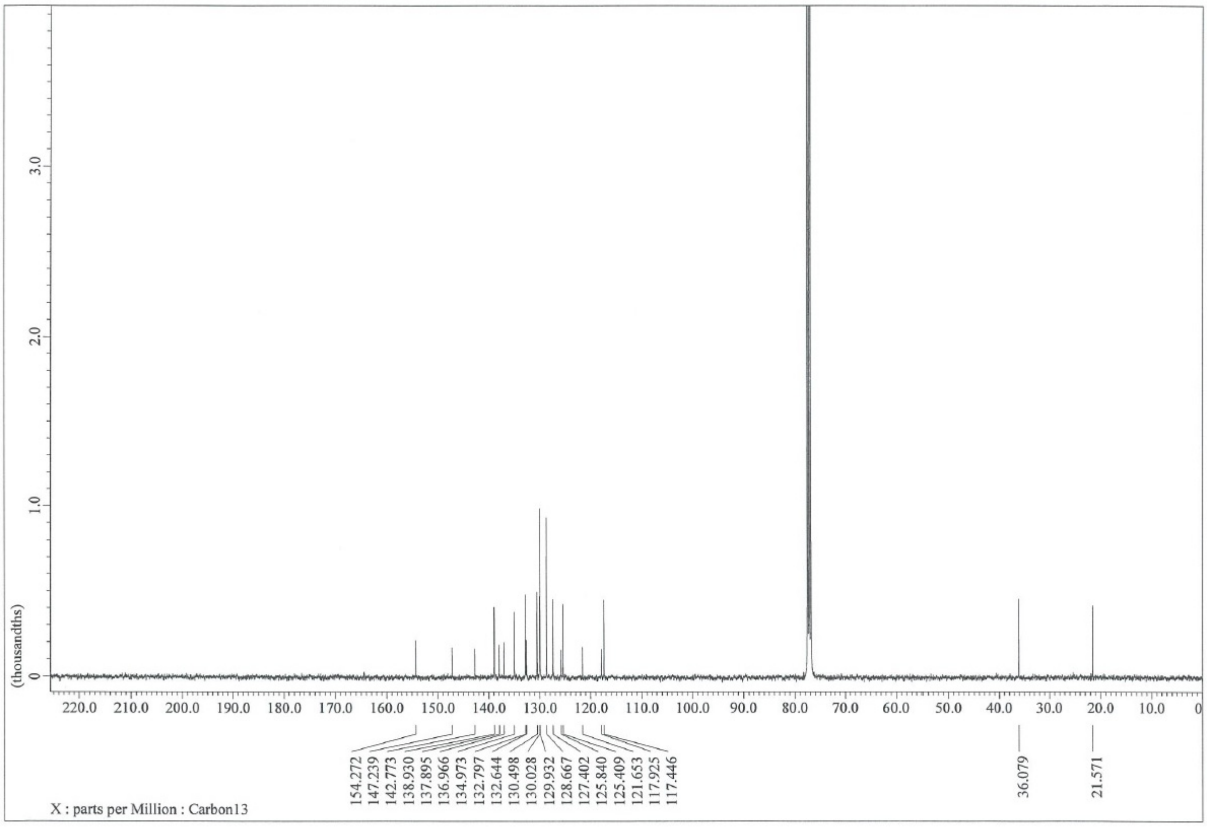

(thousandths)
0
1.0
2.0
3.0
220.0 210.0 200.0 190.0 180.0 170.0 160.0 150.0 140.0 130.0 120.0 110.0 100.0 90.0 80.0 70.0 60.0 50.0 40.0 30.0 20.0 10.0 0
154.272
147.239
142.773
138.930
137.895
136.966
134.973
132.797
132.644
130.498
130.028
129.932
128.667
127.402
125.840
125.409
121.653
117.925
117.446
36.079
21.571
X : parts per Million : Carbon13

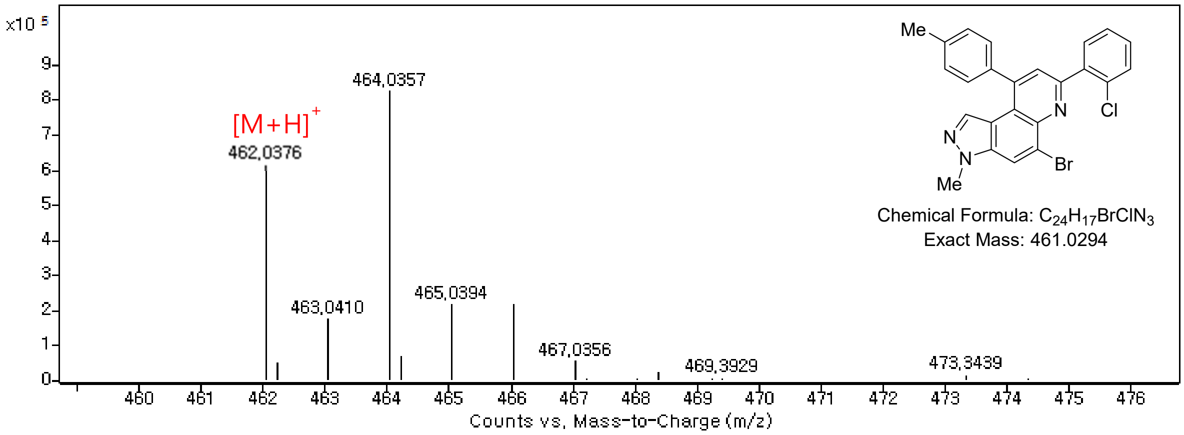
x10 5
[M+H]+
462,0376
464,0357
463,0410
465,0394
467,0356
469,3929
473,3439
Counts vs. Mass-to-Charge (m/z)
Me
N
Cl
N
N
Br
Me
Chemical Formula: C24H17BrClN3
Exact Mass: 461.0294

5-Bromo-7-(3-chlorophenyl)-3-methyl-9-(p-tolyl)-3H-pyrazolo[4,3-f]quinoline (1G)

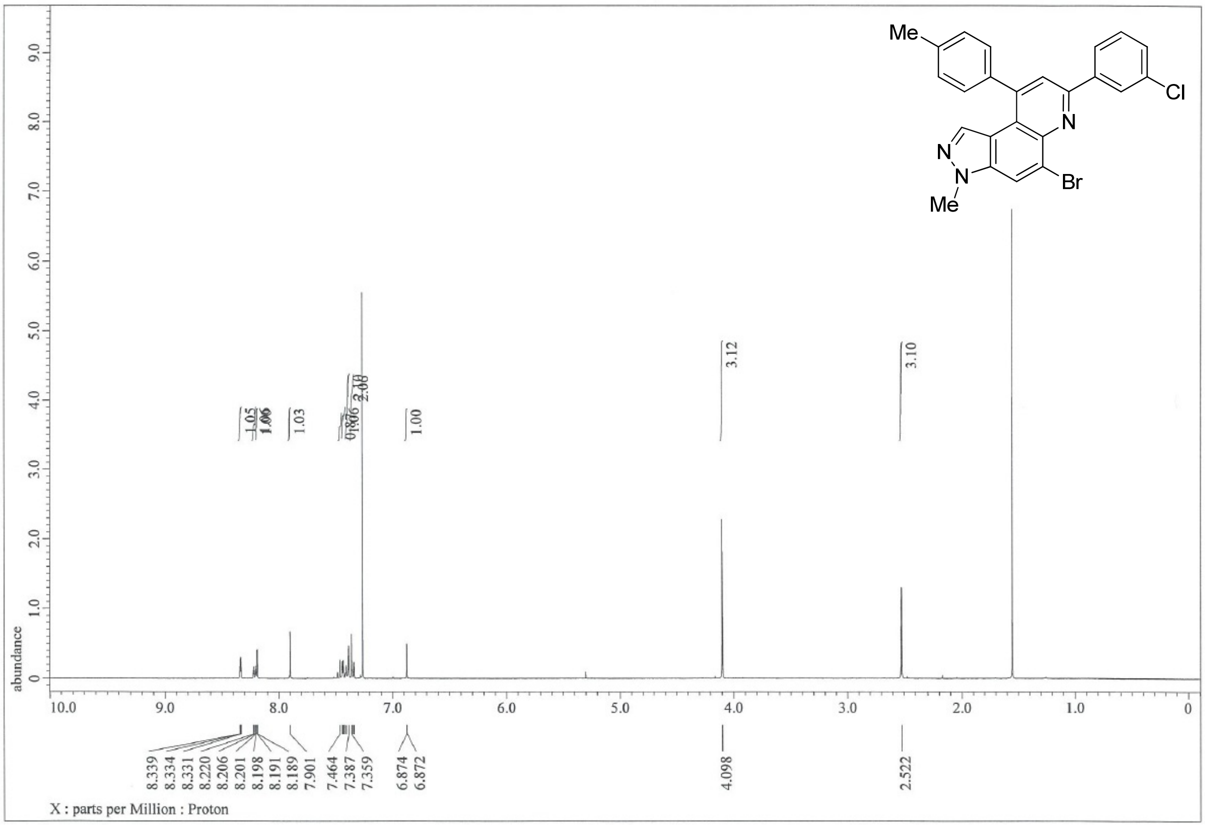

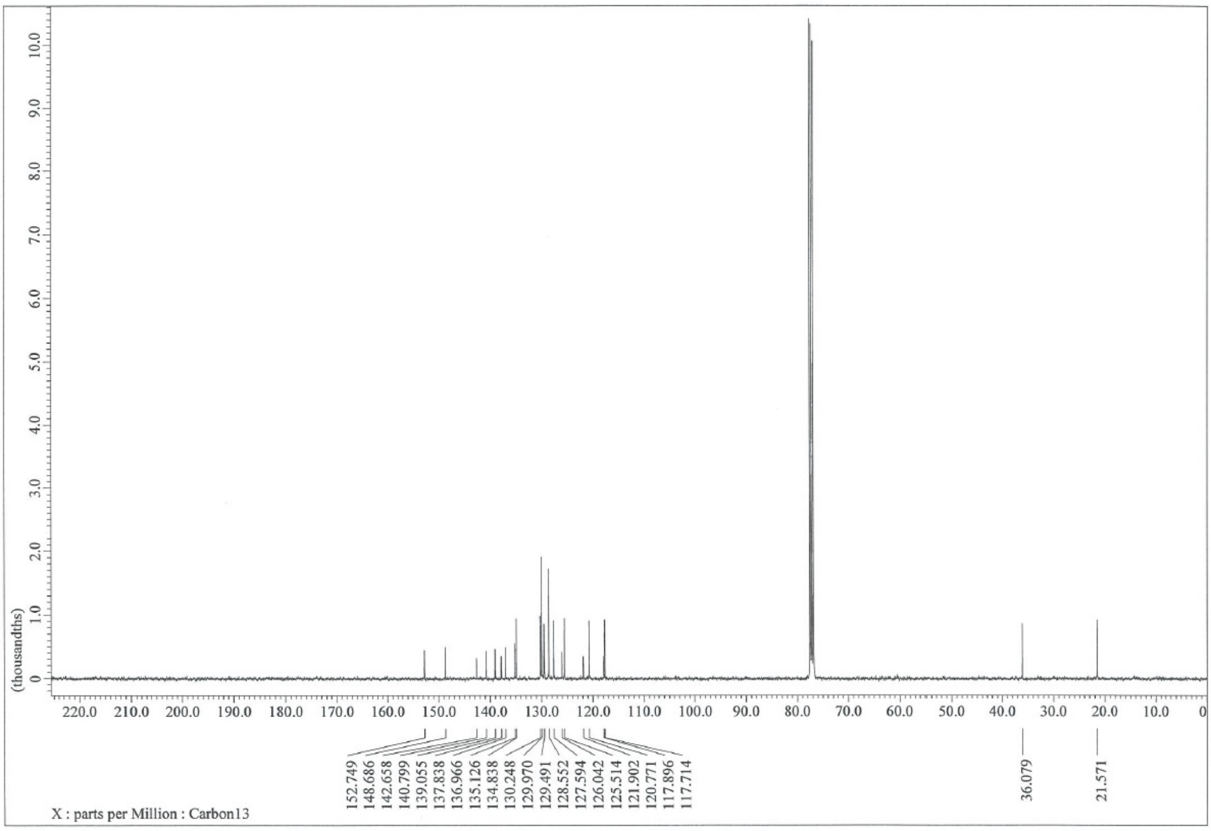
(thousandths)
X : parts per Million : Carbon13
152.749
148.686
142.658
140.799
139.055
137.838
136.966
135.126
134.838
130.248
129.970
129.491
128.552
127.594
126.042
125.514
121.902
120.771
117.896
117.714
36.079
21.571

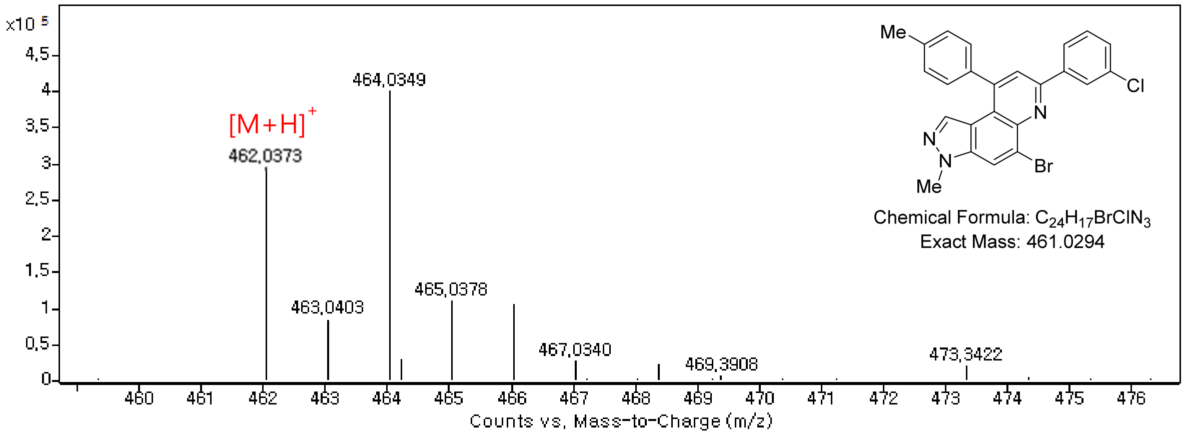
x10 5
[M+H]+
462,0373
463,0403
464,0349
465,0378
467,0340
469,3908
473,3422
Me
Cl
N
N
N
Br
Me
Chemical Formula: C24H17BrClN3
Exact Mass: 461.0294
Counts vs. Mass-to-Charge (m/z)

5-Bromo-7-(4-chlorophenyl)-3-methyl-9-(p-tolyl)-3H-pyrazolo[4,3-f]quinoline (1H)

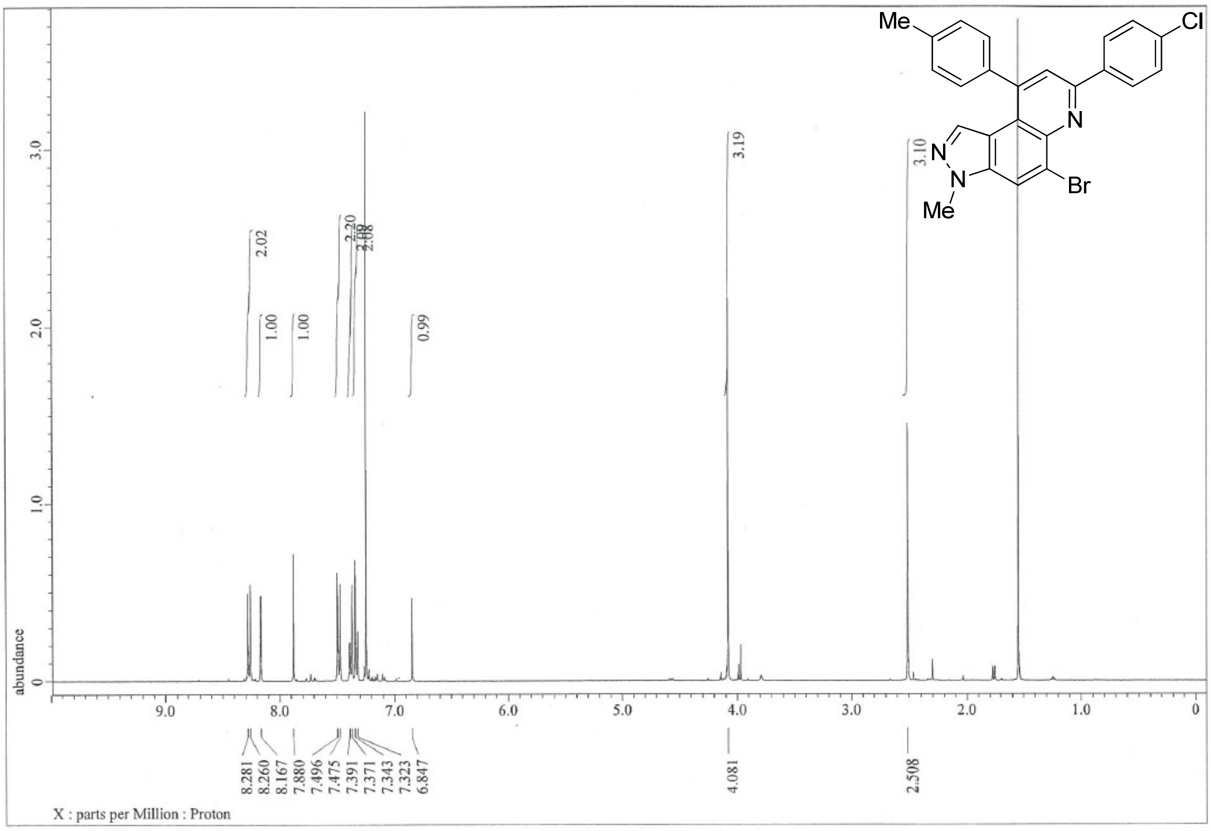

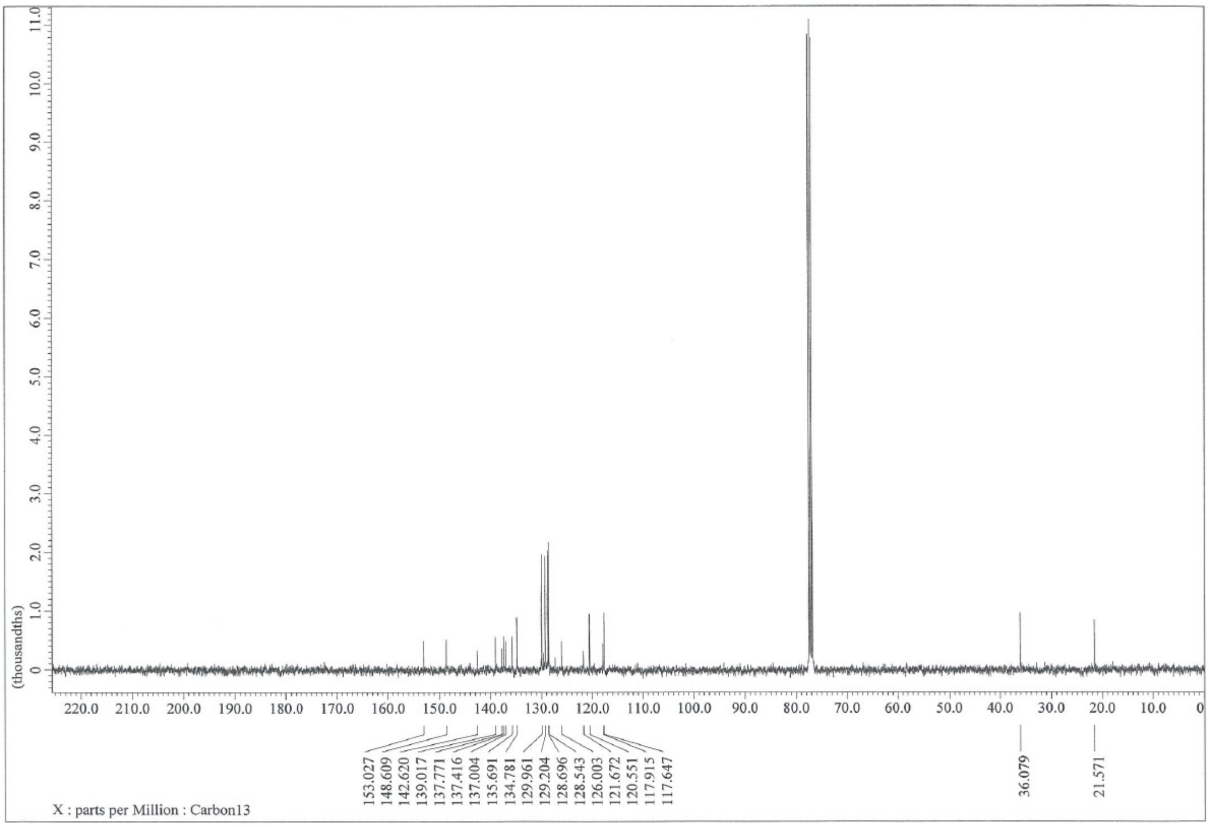

(thousandths)
11.0
10.0
9.0
8.0
7.0
6.0
5.0
4.0
3.0
2.0
1.0
0
220.0 210.0 200.0 190.0 180.0 170.0 160.0 150.0 140.0 130.0 120.0 110.0 100.0 90.0 80.0 70.0 60.0 50.0 40.0 30.0 20.0 10.0 0
153.027
148.609
142.620
139.017
137.771
137.416
137.004
135.691
134.781
129.961
129.204
128.696
128.543
126.003
121.672
120.551
117.915
117.647
36.079
21.571
X : parts per Million : Carbon13

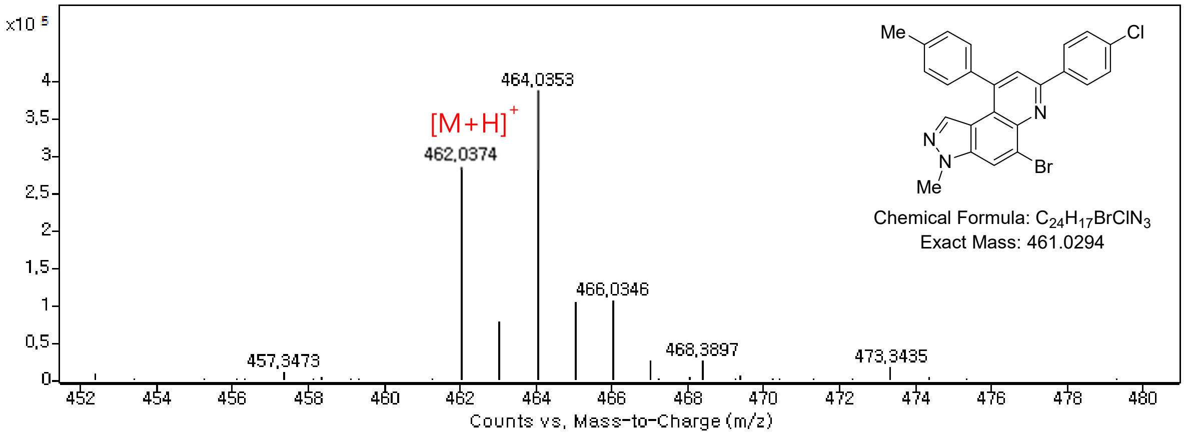

x10 5
4
3,5
3
2,5
2
1,5
1
0,5
0
[M+H]+
464,0353
462,0374
466,0346
457,3473
468,3897
473,3435
452
454
456
458
460
462
464
466
468
470
472
474
476
478
480
Counts vs. Mass-to-Charge (m/z)
Me
Cl
N
N
N
Br
Me
Chemical Formula: C24H17BrClN3
Exact Mass: 461.0294

5-Bromo-7-(4-bromophenyl)-3-methyl-9-(p-tolyl)-3H-pyrazolo[4,3-f]quinoline (1l)

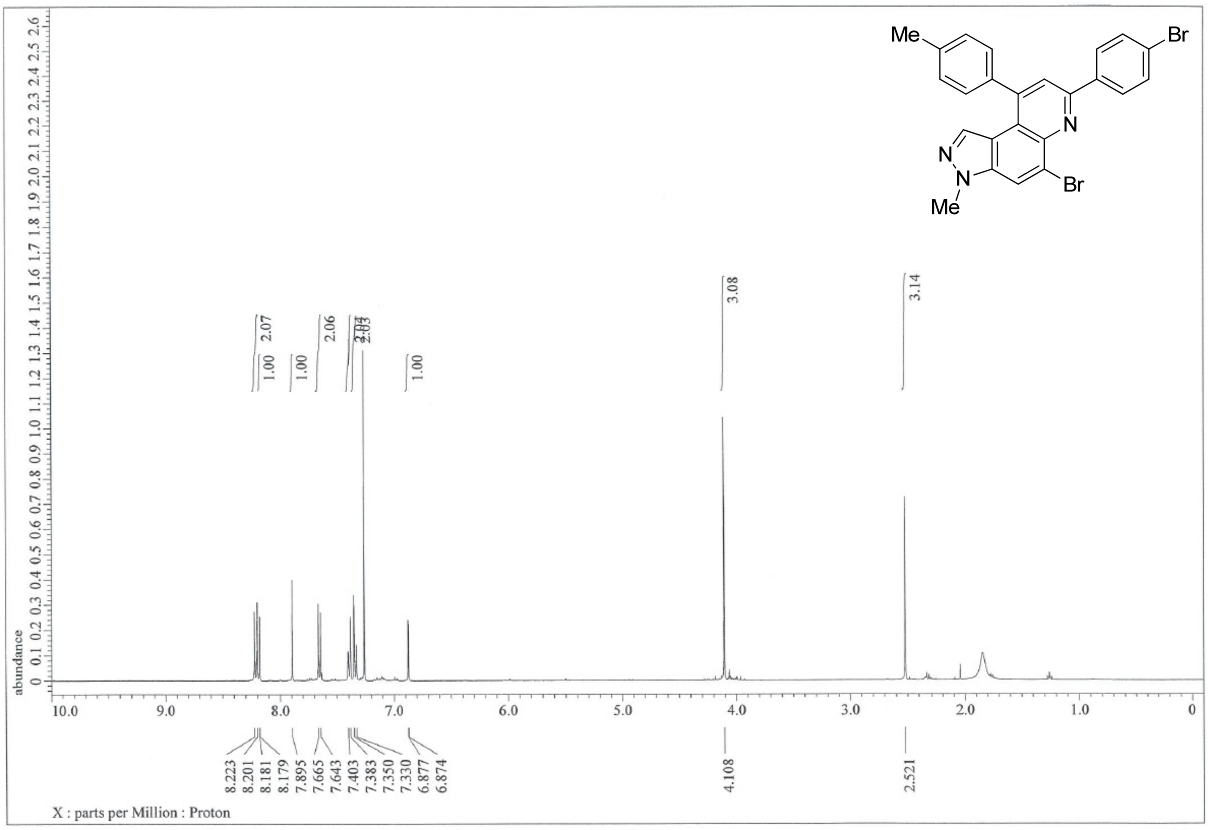

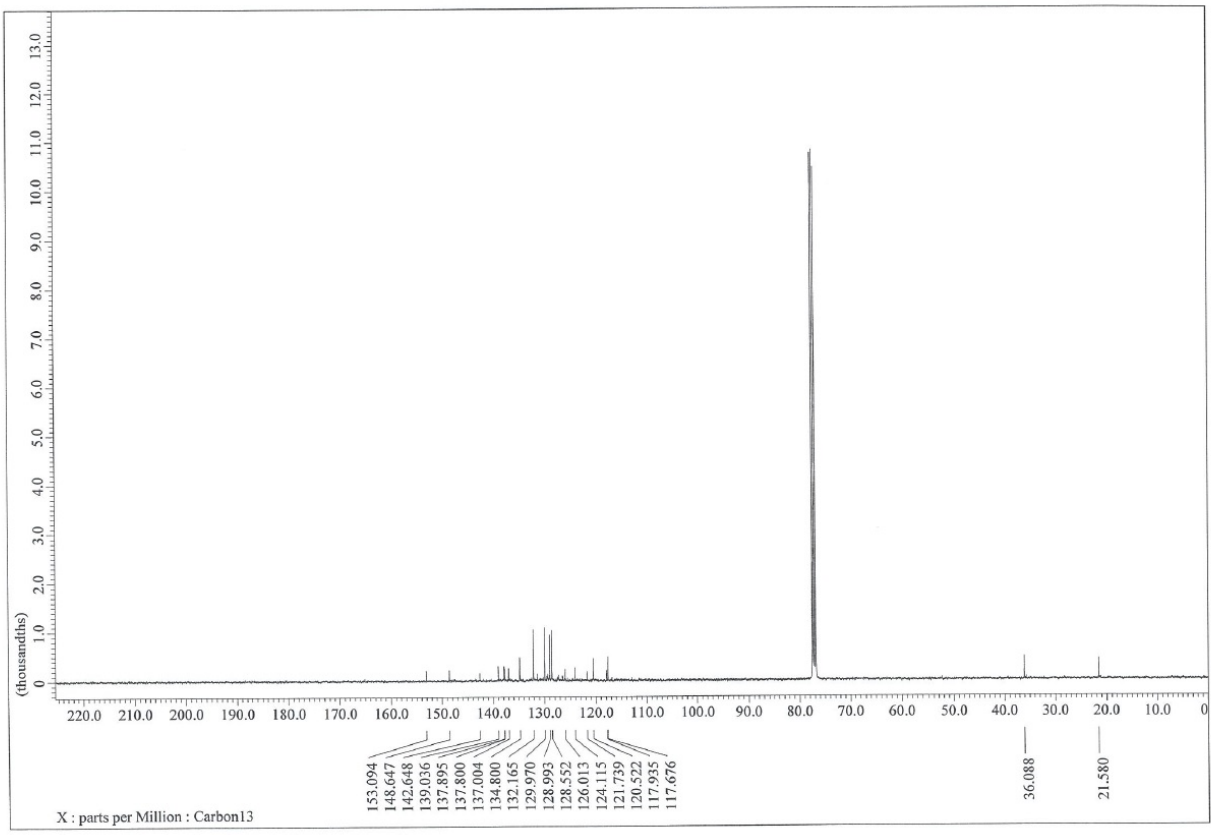
(thousandths)
X : parts per Million : Carbon13
153.094
148.647
142.648
139.036
137.895
137.800
137.004
134.800
132.165
129.970
128.993
128.552
126.013
124.115
121.739
120.522
117.935
117.676
36.088
21.580

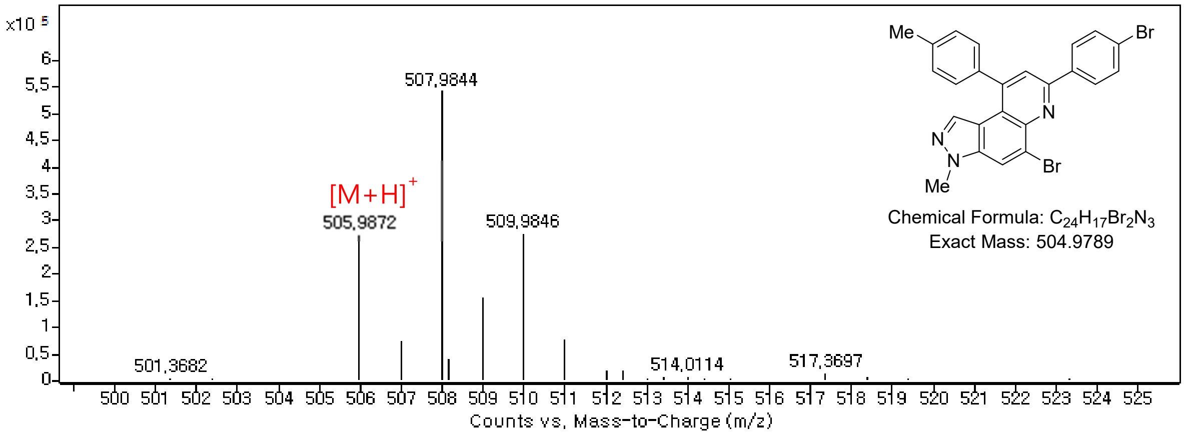

x10 5
6
5,5
5
4,5
4
3,5
3
2,5
2
1,5
1
0,5
0
[M+H]+
505,9872
507,9844
509,9846
501,3682
514,0114
517,3697
500 501 502 503 504 505 506 507 508 509 510 511 512 513 514 515 516 517 518 519 520 521 522 523 524 525
Counts vs. Mass-to-Charge (m/z)
Me
Br
N
N
N
Me
Br
Chemical Formula: C24H17Br2N3
Exact Mass: 504.9789

4-(5-Bromo-3-methyl-9-(p-tolyl)-3H-pyrazolo[4,3-f]quinolin-7-yl)-N,N-dimethylaniline (1J)

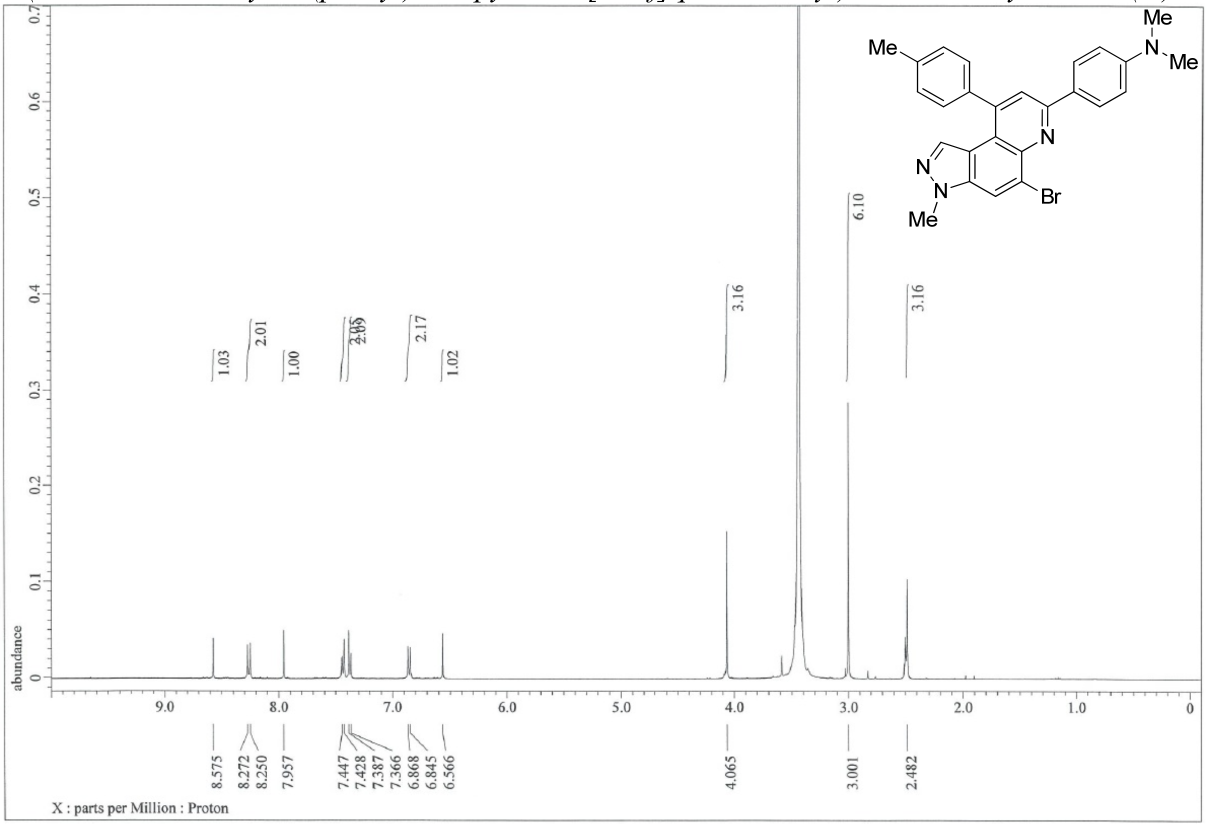

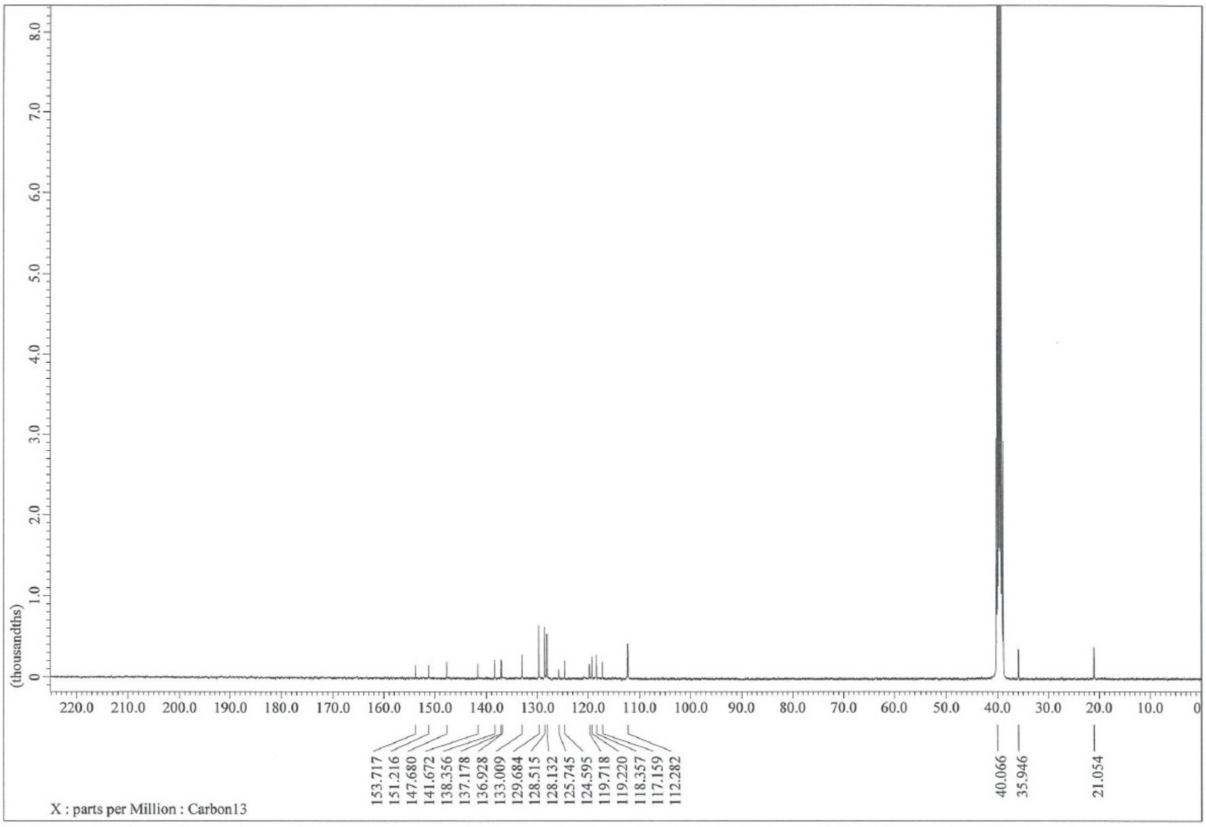
(thousandths)
8.0
7.0
6.0
5.0
4.0
3.0
2.0
1.0
0
220.0 210.0 200.0 190.0 180.0 170.0 160.0 150.0 140.0 130.0 120.0 110.0 100.0 90.0 80.0 70.0 60.0 50.0 40.0 30.0 20.0 10.0 0
153.717
151.216
147.680
141.672
138.356
137.178
136.928
133.009
129.684
128.515
128.132
125.745
124.595
119.718
119.220
118.357
117.159
112.282
40.066
35.946
21.054
X : parts per Million : Carbon13

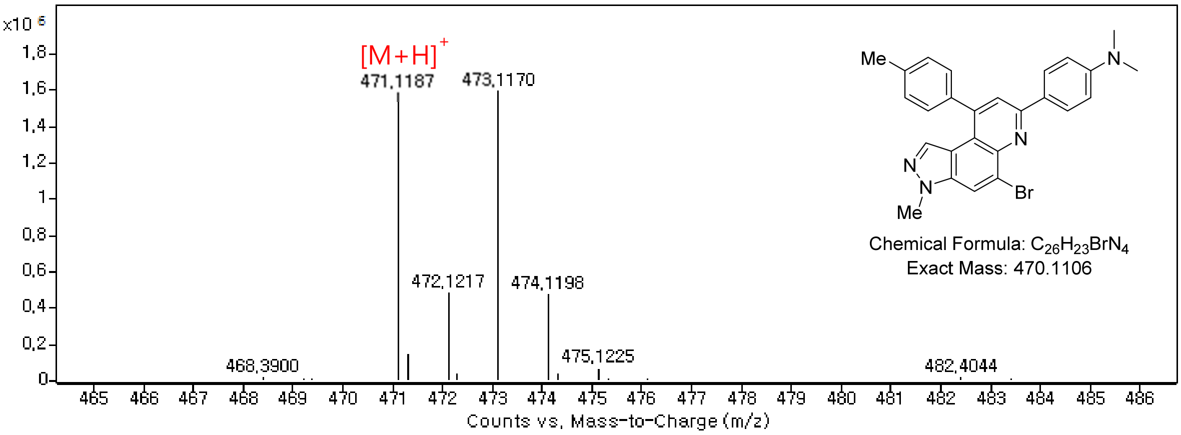
x10 6
1,8
1,6
1,4
1,2
1
0,8
0,6
0,4
0,2
0
[M+H]+
471,1187
473,1170
472,1217
474,1198
475,1225
468,3900
482,4044
465 466 467 468 469 470 471 472 473 474 475 476 477 478 479 480 481 482 483 484 485 486
Counts vs. Mass-to-Charge (m/z)
Me
N
N
N
Br
Me
Chemical Formula: C26H23BrN4
Exact Mass: 470.1106

5-Bromo-9-(4-methoxyphenyl)-3-methyl-7-phenyl-3H-pyrazolo[4,3-f]quinoline (1K)

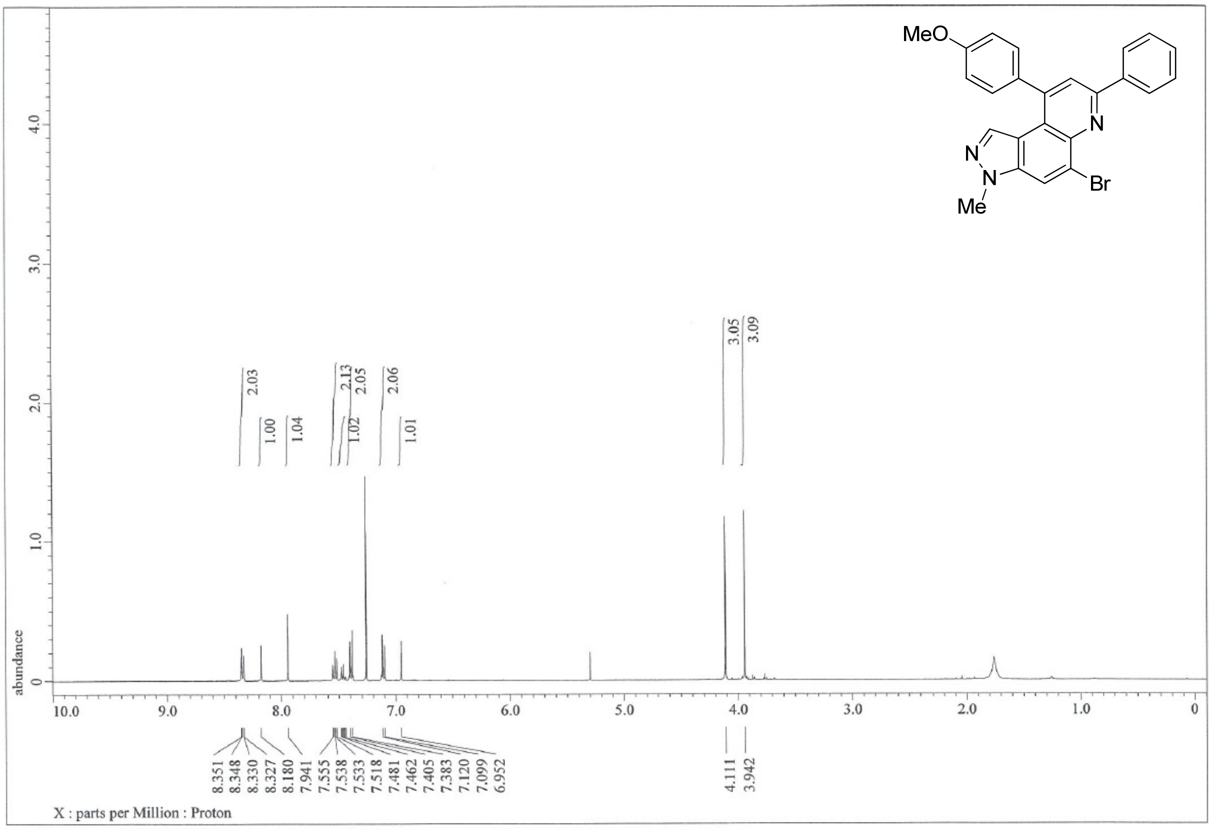

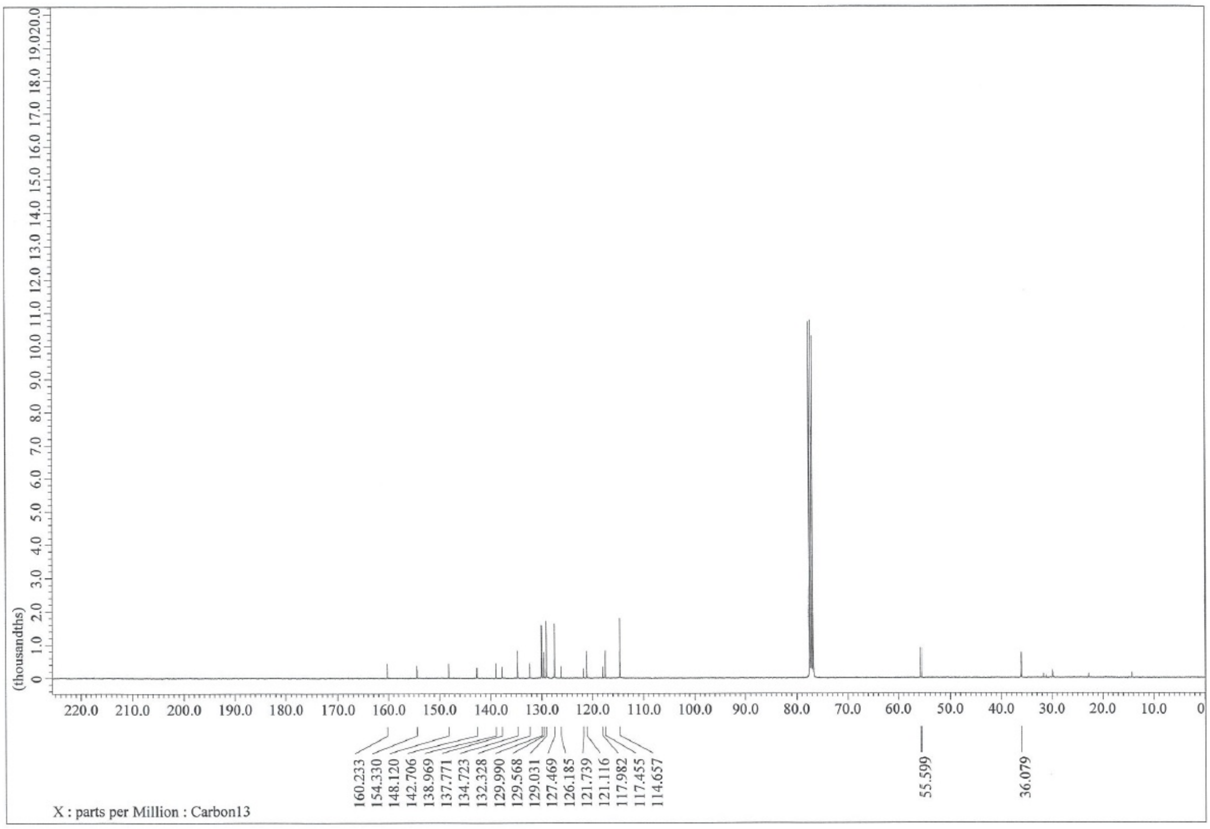

(thousandths)
X : parts per Million : Carbon13
160.233
154.330
148.120
142.706
138.969
137.771
134.723
132.328
129.990
129.568
129.031
127.469
126.185
121.739
121.116
117.982
117.455
114.657
55.599
36.079

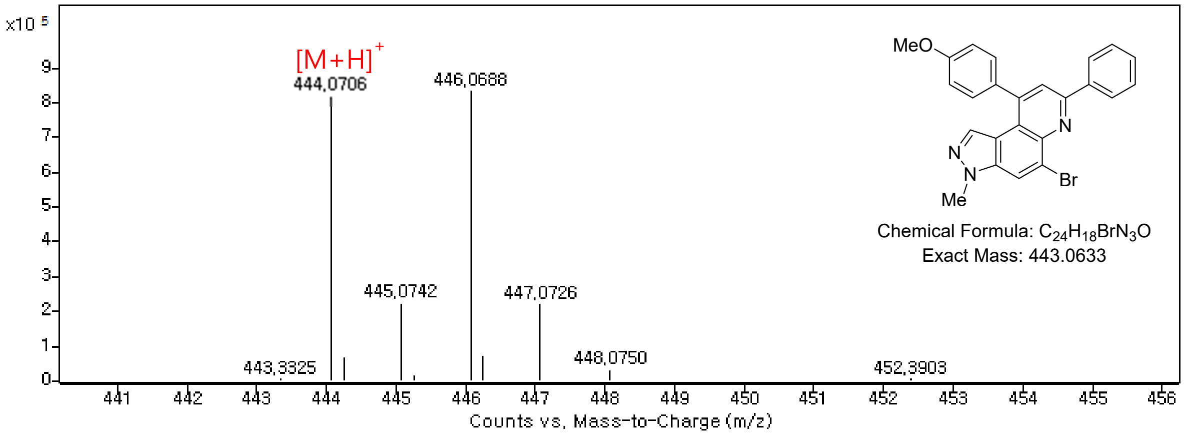
x10 5
[M+H]+
444.0706
446.0688
445.0742
447.0726
448.0750
443.3325
452.3903
MeO
N
N
N
Me
Br
Chemical Formula: C24H18BrN3O
Exact Mass: 443.0633
Counts vs. Mass-to-Charge (m/z)

5-Bromo-9-(4-methoxyphenyl)-3-methyl-7-(pyridin-3-yl)-3H-pyrazolo[4,3-f]quinoline (1L)

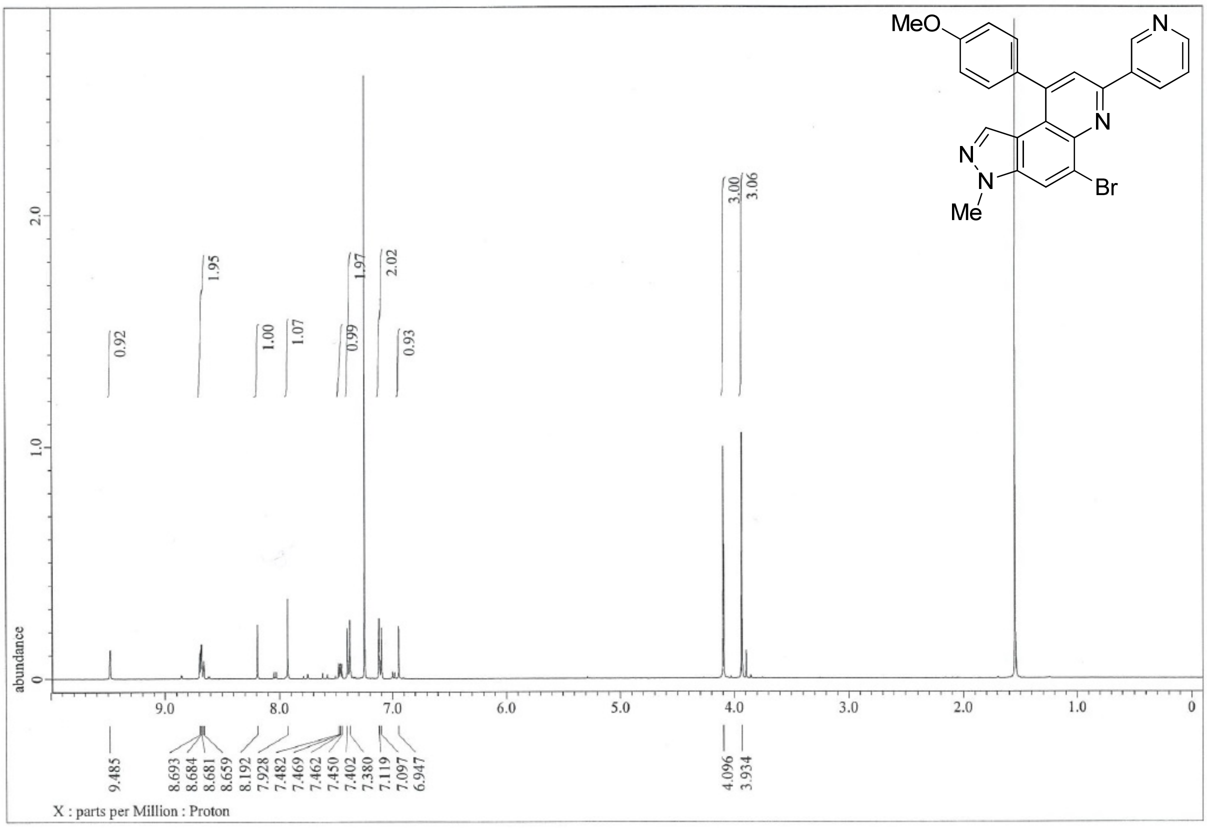

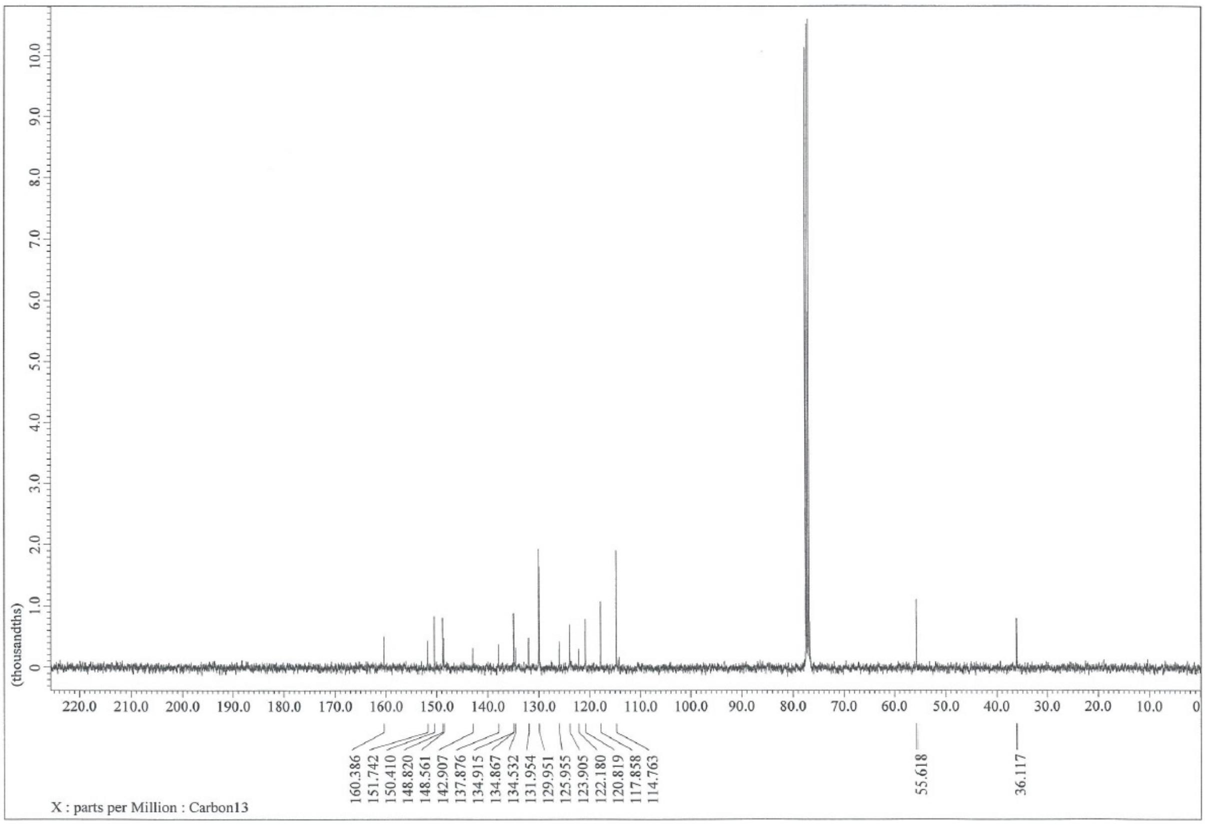

(thousandths)
10.0
9.0
8.0
7.0
6.0
5.0
4.0
3.0
2.0
1.0
0
220.0 210.0 200.0 190.0 180.0 170.0 160.0 150.0 140.0 130.0 120.0 110.0 100.0 90.0 80.0 70.0 60.0 50.0 40.0 30.0 20.0 10.0 0
160.386
151.742
150.410
148.820
148.561
142.907
137.876
134.915
134.867
134.532
131.954
129.951
125.955
123.905
122.180
120.819
117.858
114.763
55.618
36.117
X : parts per Million : Carbon13

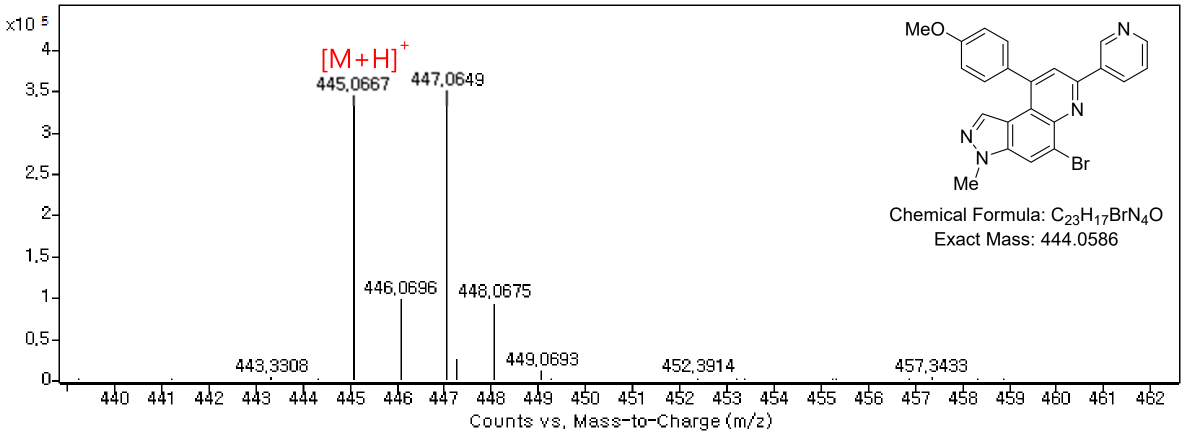
x10 5
[M+H]+
445,0667
447,0649
446,0696
448,0675
443,3308
449,0693
452,3914
457,3433
Counts vs. Mass-to-Charge (m/z)
MeO
N
N
N
N
Me
Br
Chemical Formula: C23H17BrN4O
Exact Mass: 444.0586

3-(5-Bromo-9-(4-methoxyphenyl)-3-methyl-3H-pyrazolo[4,3-f]quinolin-7-yl)benzonitrile (1M)

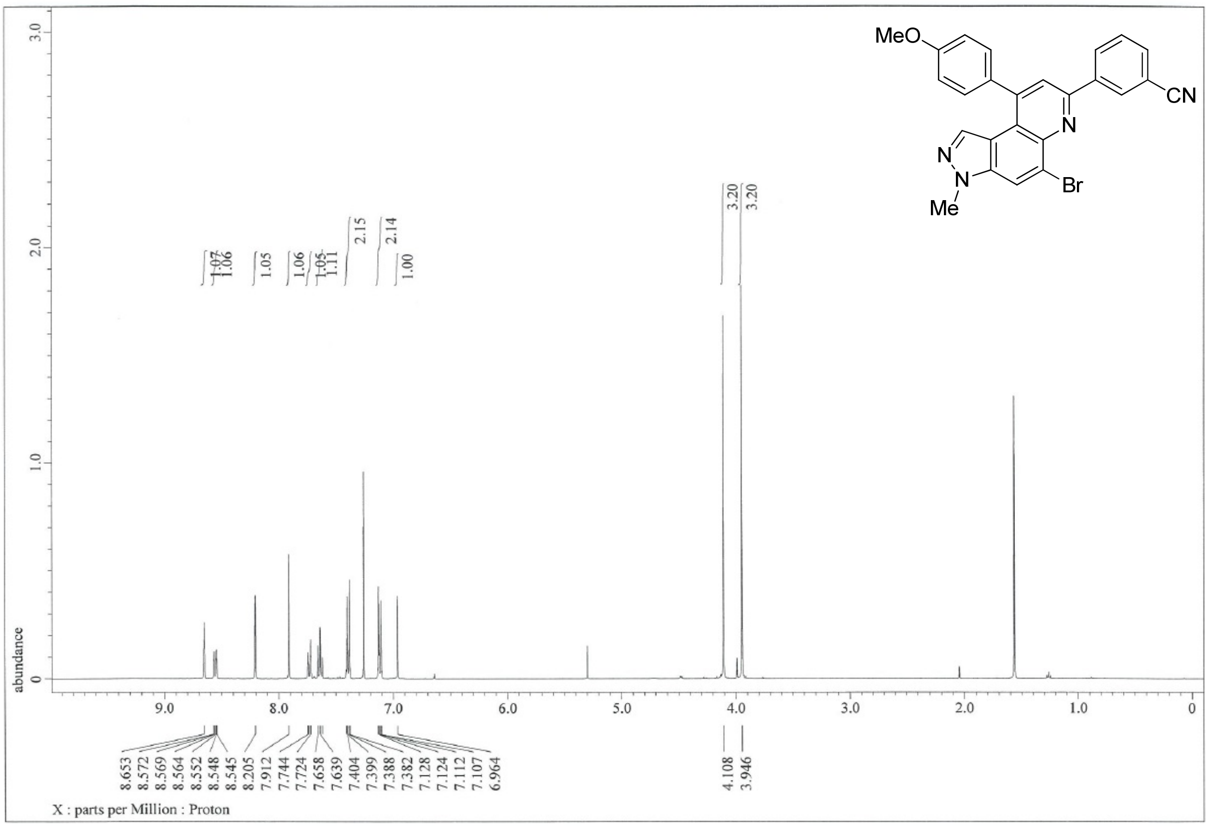

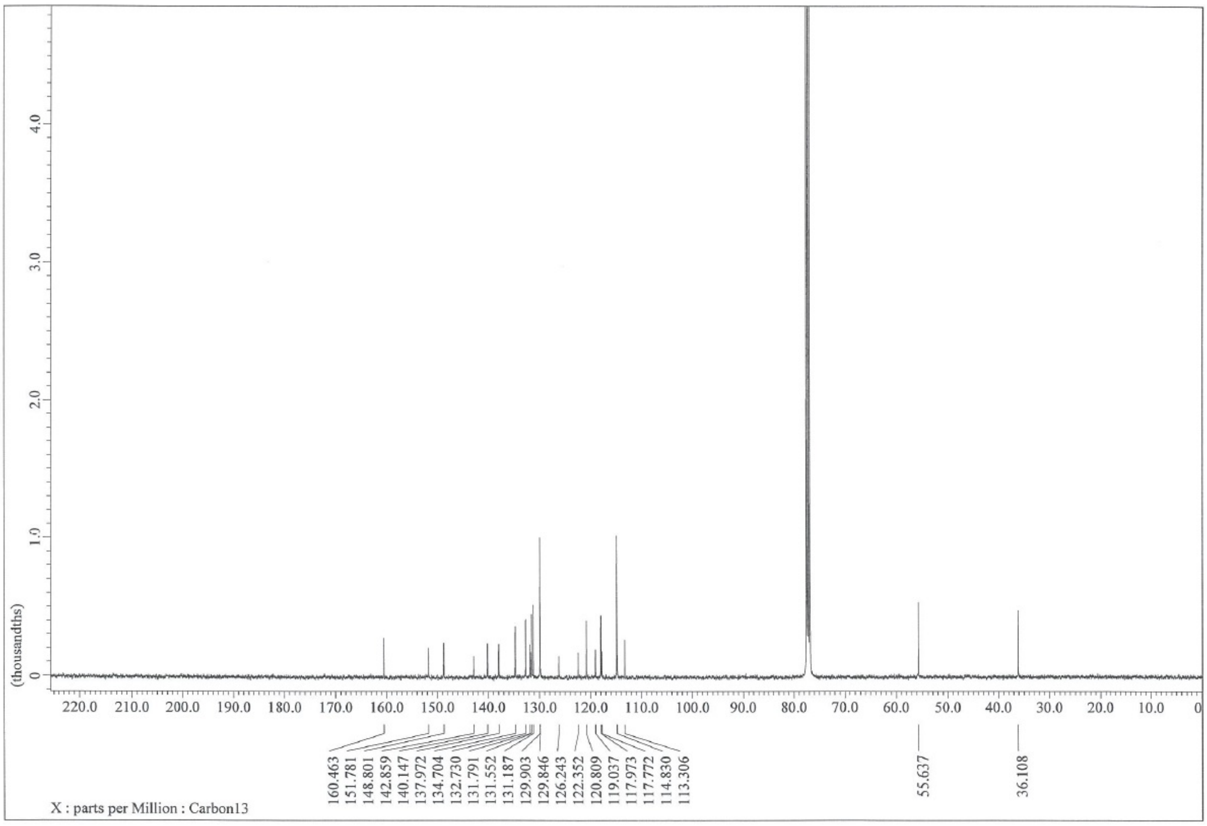

(thousandths)
4.0
3.0
2.0
1.0
0
220.0 210.0 200.0 190.0 180.0 170.0 160.0 150.0 140.0 130.0 120.0 110.0 100.0 90.0 80.0 70.0 60.0 50.0 40.0 30.0 20.0 10.0 0
160.463
151.781
148.801
142.859
140.147
137.972
134.704
132.730
131.791
131.552
131.187
129.903
129.846
126.243
122.352
120.809
119.037
117.973
117.772
114.830
113.306
55.637
36.108
X : parts per Million : Carbon13

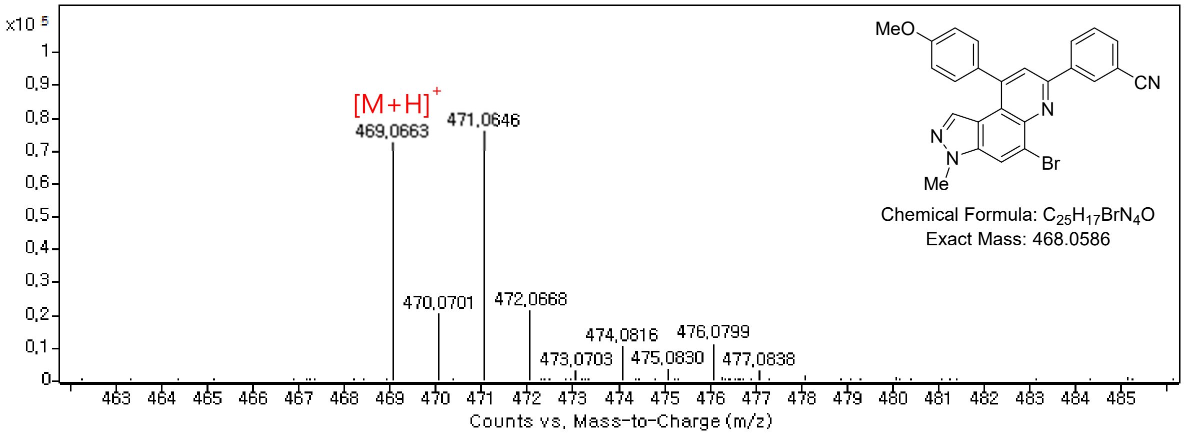
x10 5
1
0,9
0,8
0,7
0,6
0,5
0,4
0,3
0,2
0,1
0
[M+H]+
469,0663
471,0646
470,0701
472,0668
473,0703
474,0816
475,0830
476,0799
477,0838
463 464 465 466 467 468 469 470 471 472 473 474 475 476 477 478 479 480 481 482 483 484 485
Counts vs. Mass-to-Charge (m/z)
MeO
CN
N
N
N
Me
Br
Chemical Formula: C25H17BrN4O
Exact Mass: 468.0586

5-Bromo-9-(4-methoxyphenyl)-3-methyl-7-(4-(trifluoromethyl)phenyl)-3H-pyrazolo[4,3-f]quinoline (1N)

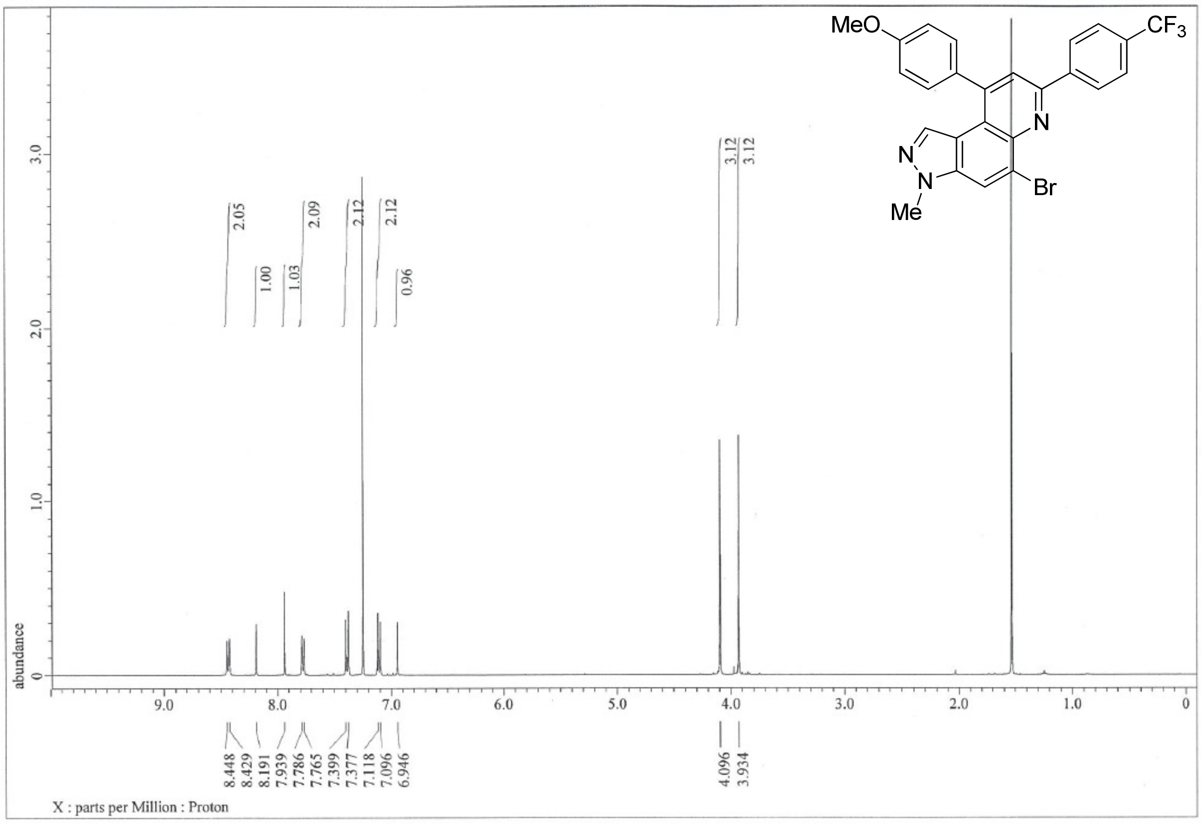

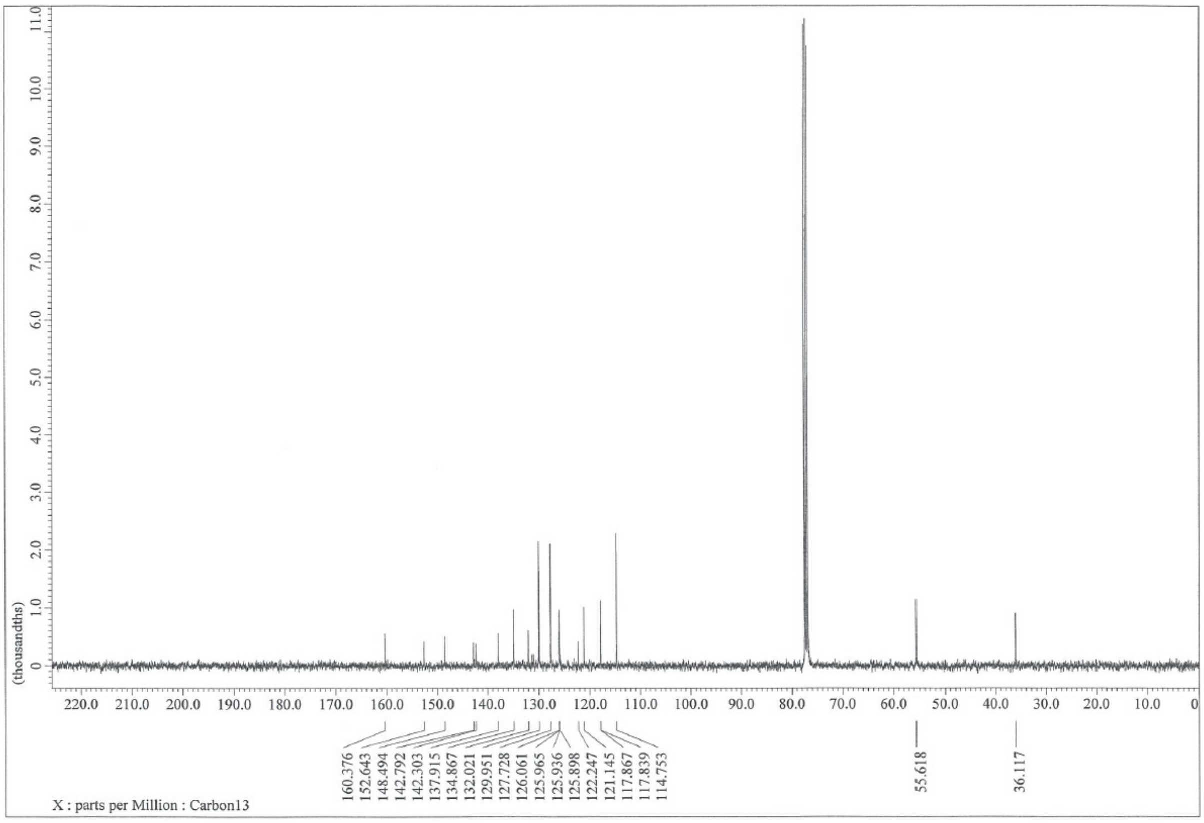

(thousandths)
X : parts per Million : Carbon13
160.376
152.643
148.494
142.792
142.303
137.915
134.867
132.021
129.951
127.728
126.061
125.965
125.936
125.898
122.247
121.145
117.867
117.839
114.753
55.618
36.117

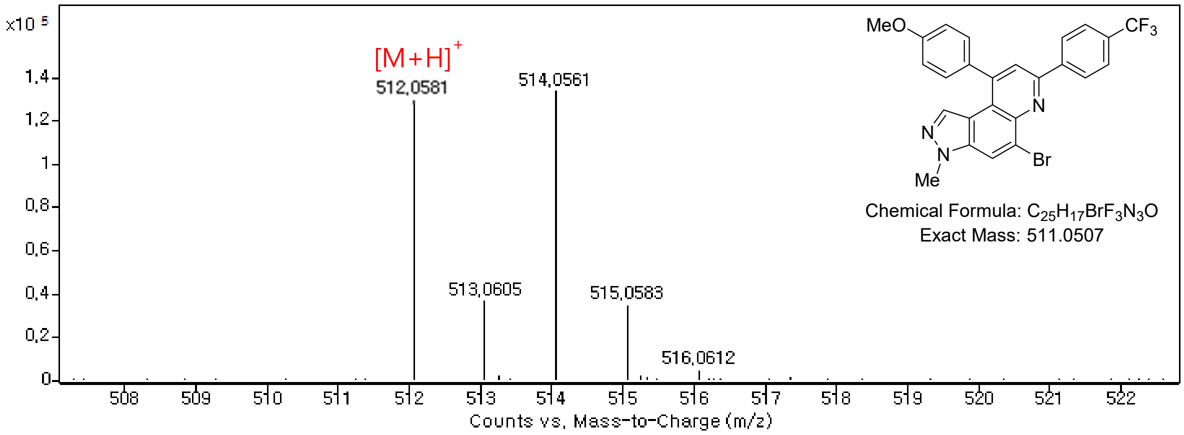
x10 5
[M+H]+
512,0581
514,0561
513,0605
515,0583
516,0612
1,4
1,2
1
0,8
0,6
0,4
0,2
0
508
509
510
511
512
513
514
515
516
517
518
519
520
521
522
Counts vs. Mass-to-Charge (m/z)
MeO
CF3
N
N
N
Me
Br
Chemical Formula: C25H17BrF3N3O
Exact Mass: 511.0507

5-Bromo-9-(4-methoxyphenyl)-3-methyl-7-(4-nitrophenyl)-3H-pyrazolo[4,3-f]quinoline (1O)

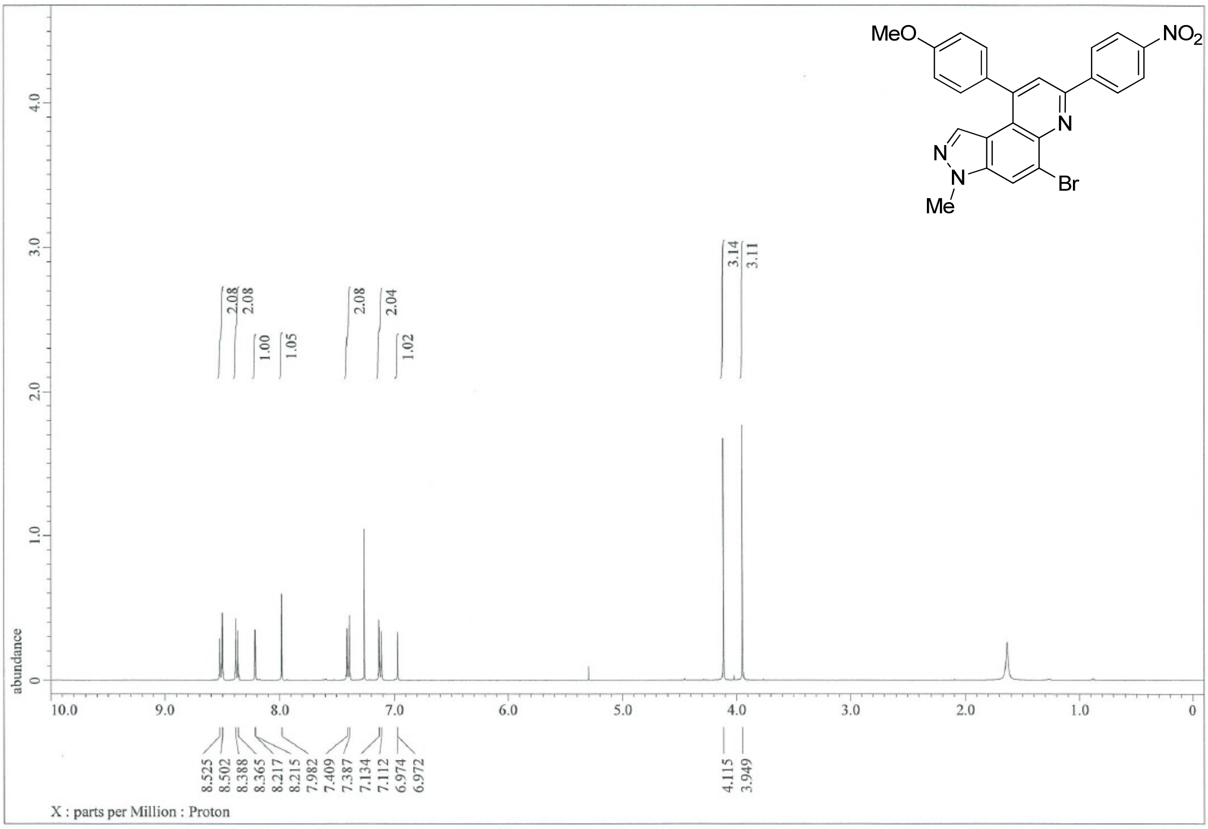

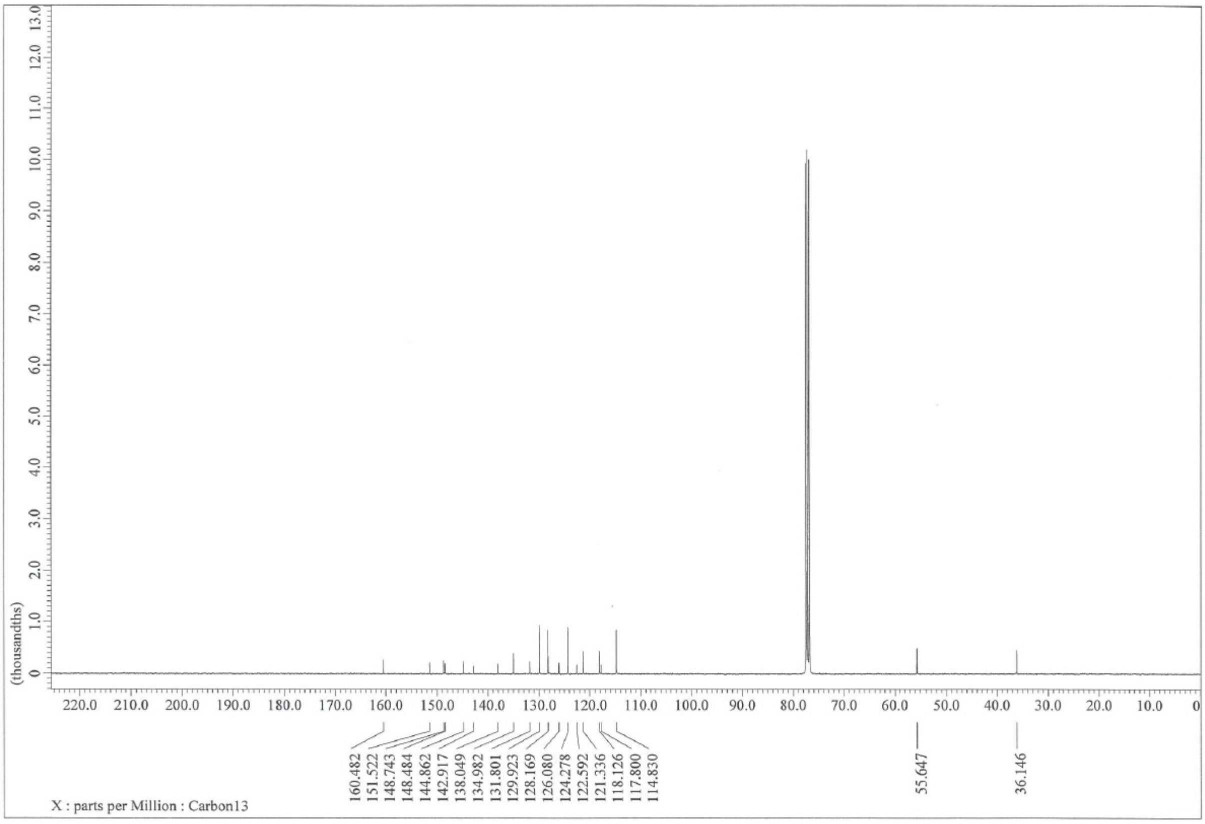

(thousandths)
13.0
12.0
11.0
10.0
9.0
8.0
7.0
6.0
5.0
4.0
3.0
2.0
1.0
0
220.0 210.0 200.0 190.0 180.0 170.0 160.0 150.0 140.0 130.0 120.0 110.0 100.0 90.0 80.0 70.0 60.0 50.0 40.0 30.0 20.0 10.0 0
160.482
151.522
148.743
148.484
144.862
142.917
138.049
134.982
131.801
129.923
128.169
126.080
124.278
122.592
121.336
118.126
117.800
114.830
55.647
36.146
X : parts per Million : Carbon13

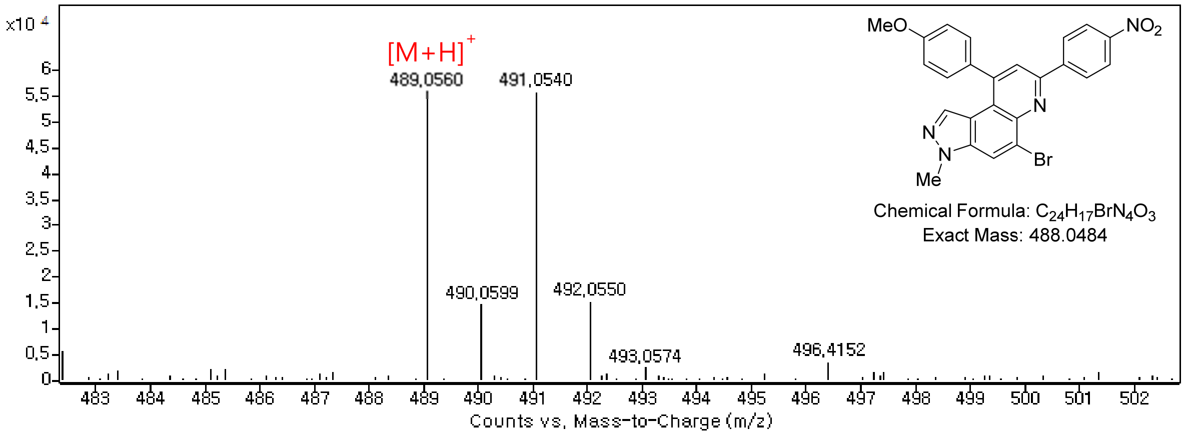
x10 4
[M+H]+
489,0560
491,0540
490,0599
492,0550
493,0574
496,4152
Counts vs. Mass-to-Charge (m/z)
MeO
NO2
N
N
N
Me
Br
Chemical Formula: C24H17BrN4O3
Exact Mass: 488.0484

5-Bromo-9-(4-methoxyphenyl)-3-methyl-7-(thiophen-2-yl)-3H-pyrazolo[4,3-f]quinoline (1P)

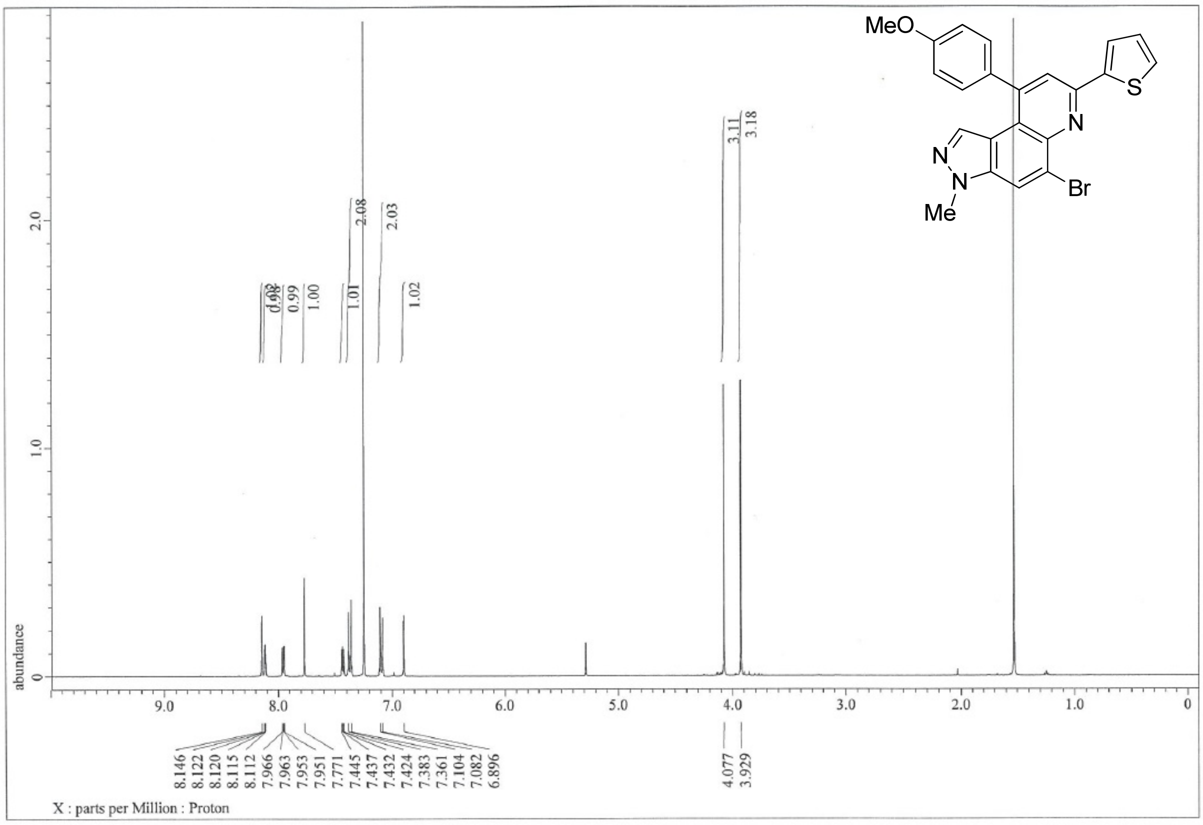

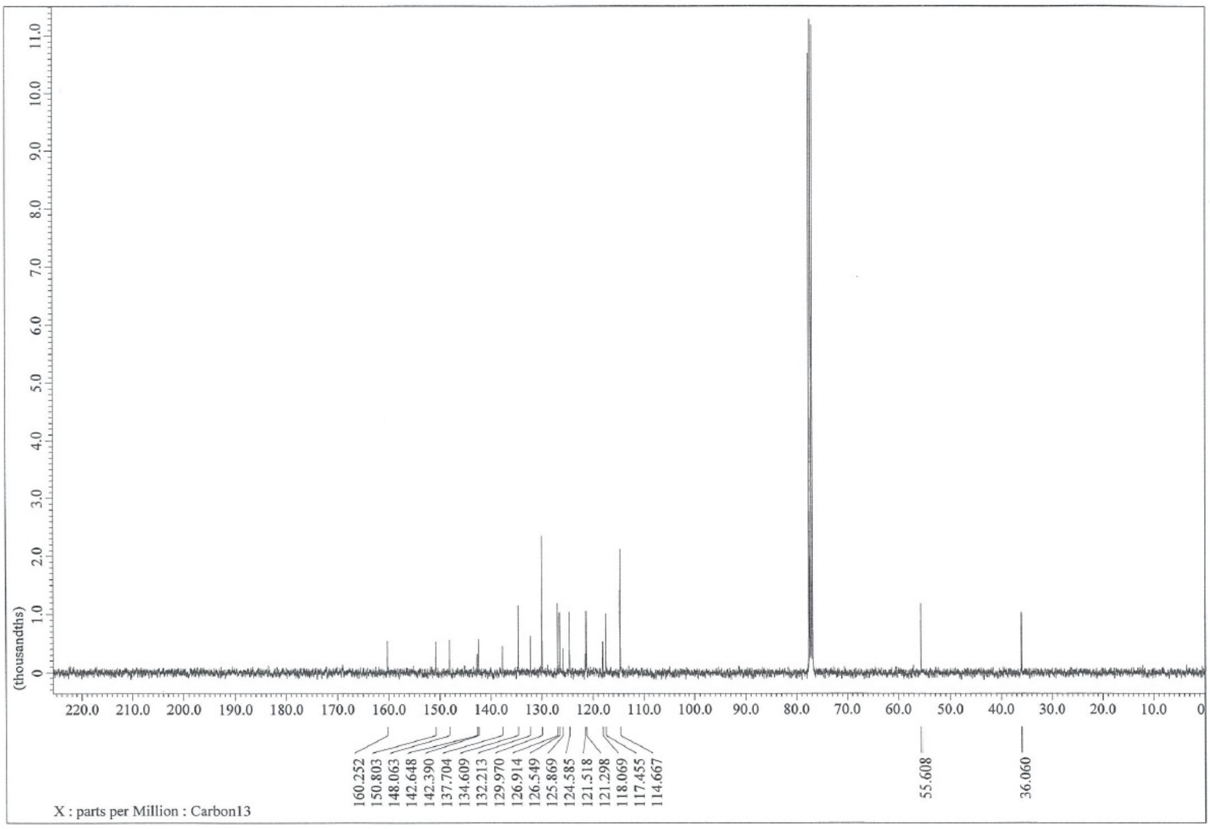
(thousandths)
0
1.0
2.0
3.0
4.0
5.0
6.0
7.0
8.0
9.0
10.0
11.0
220.0 210.0 200.0 190.0 180.0 170.0 160.0 150.0 140.0 130.0 120.0 110.0 100.0 90.0 80.0 70.0 60.0 50.0 40.0 30.0 20.0 10.0 0
160.252
150.803
148.063
142.648
142.390
137.704
134.609
132.213
129.970
126.914
126.549
125.869
124.585
121.518
121.298
118.069
117.455
114.667
55.608
36.060
X : parts per Million : Carbon13

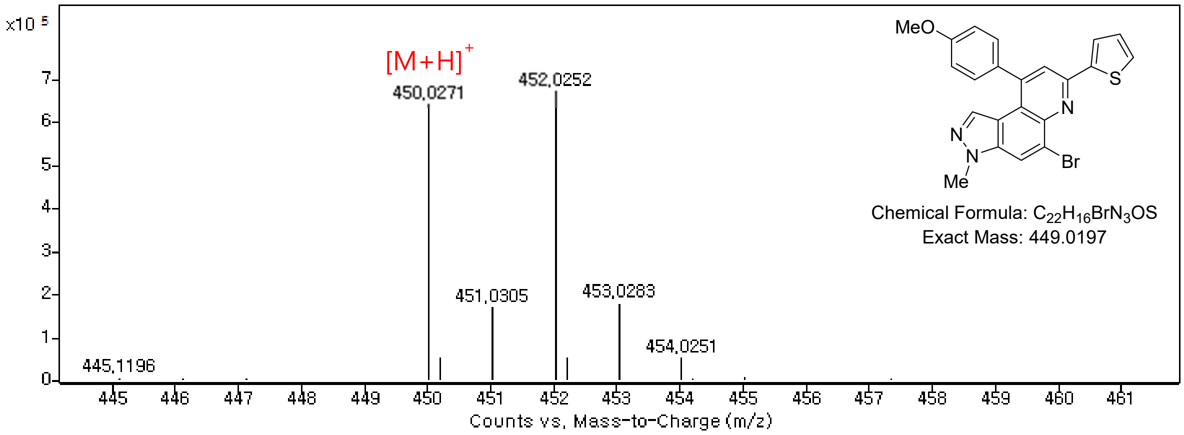
x10 5
[M+H]+
450.0271
452.0252
451.0305
453.0283
454.0251
445.1196
Counts vs. Mass-to-Charge (m/z)
MeO
S
N
N
N
Me
Br
Chemical Formula: C22H16BrN3OS
Exact Mass: 449.0197

5-Bomo-7-(2-chlorophenyl)-9-(4-methoxyphenyl)-3-methyl-3H-pyrazolo[4,3-f]quinoline (1Q)

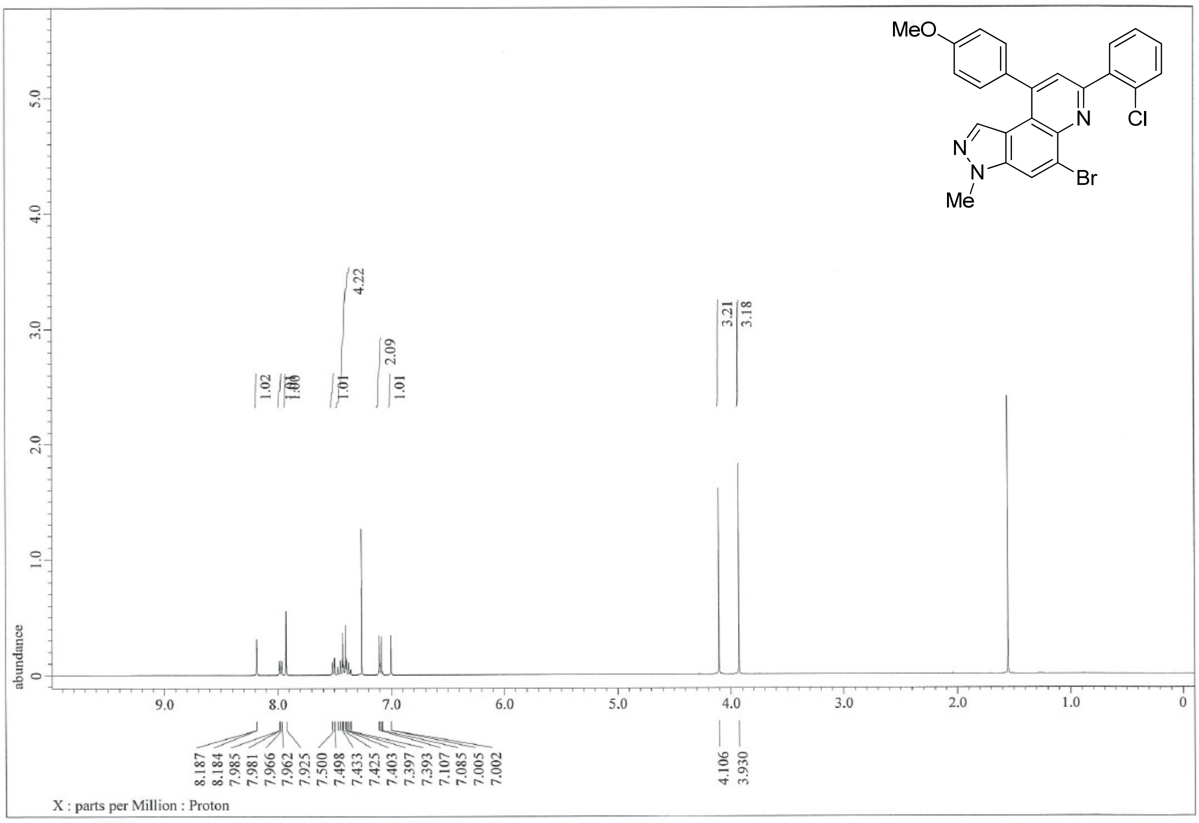

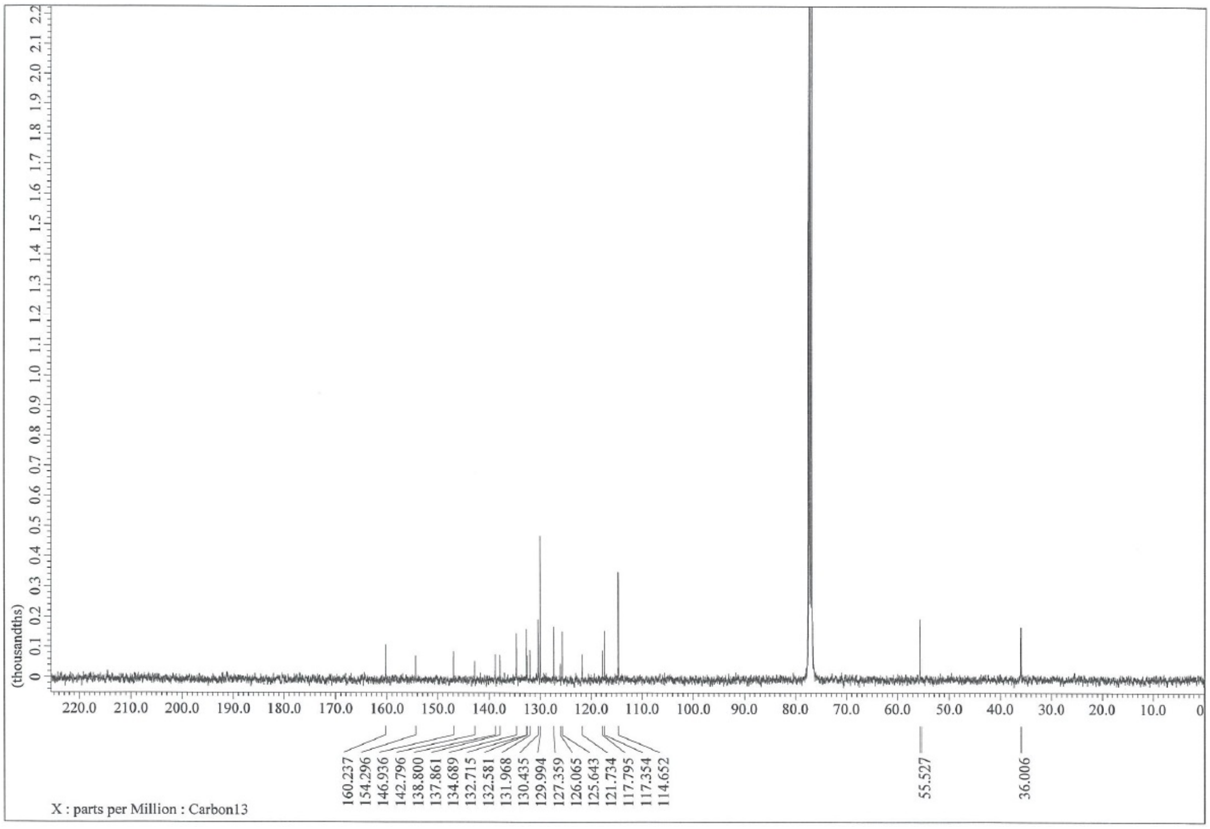

(thousandths)
X : parts per Million : Carbon13
160.237
154.296
146.936
142.796
138.800
137.861
134.689
132.715
132.581
131.968
130.435
129.994
127.359
126.065
125.643
121.734
117.795
117.354
114.652
55.527
36.006

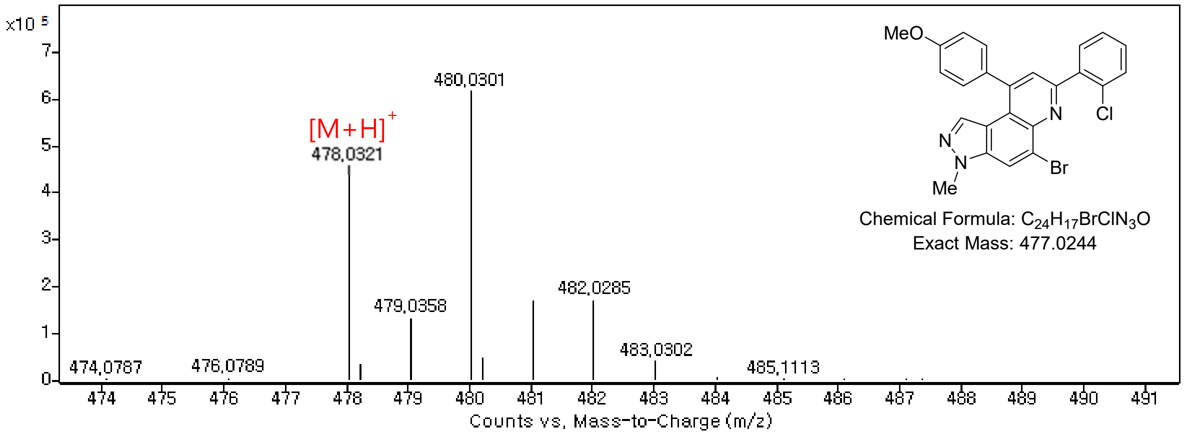

x10 5
[M+H]+
478.0321
480.0301
479.0358
482.0285
483.0302
474.0787
476.0789
485.1113
Counts vs. Mass-to-Charge (m/z)
MeO
N
Cl
N
N
Br
Me
Chemical Formula: C24H17BrClN3O
Exact Mass: 477.0244

5-Bomo-7-(4-chlorophenyl)-9-(4-methoxyphenyl)-3-methyl-3H-pyrazolo[4,3-f]quinoline (1R)

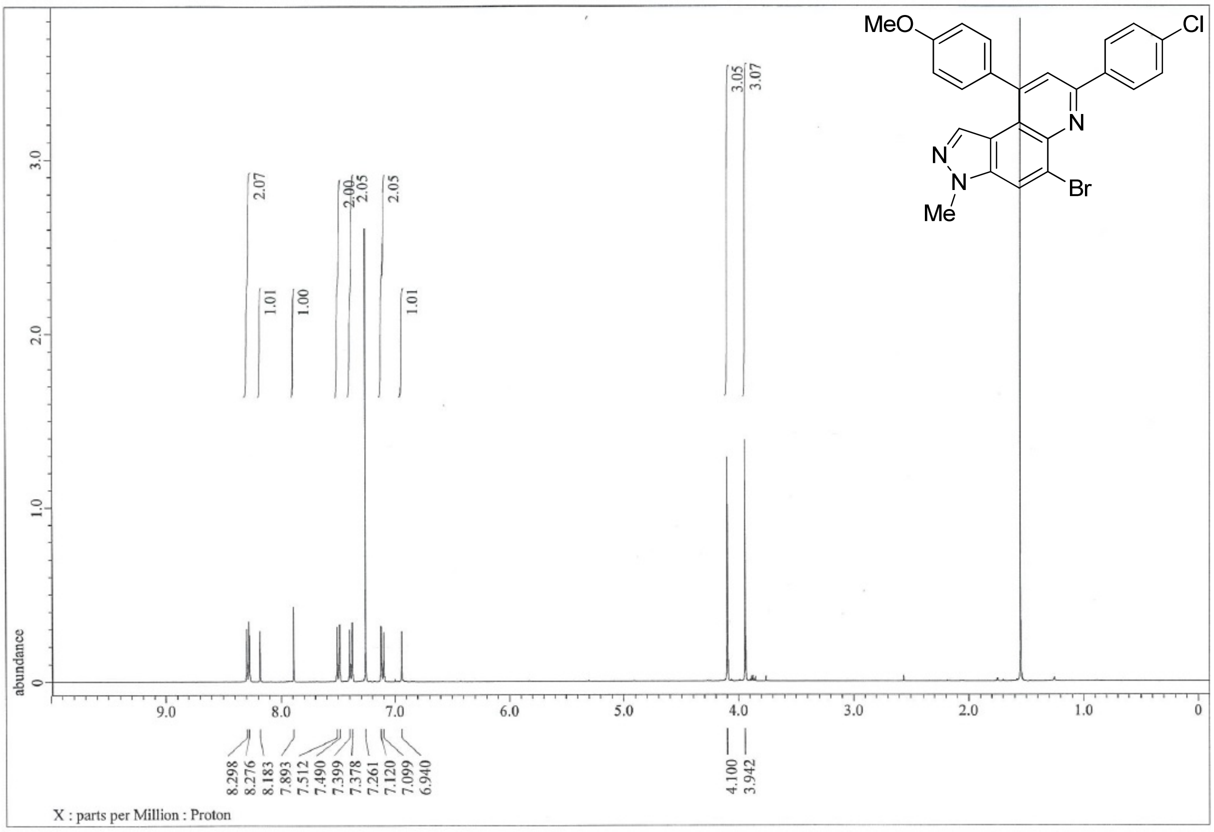

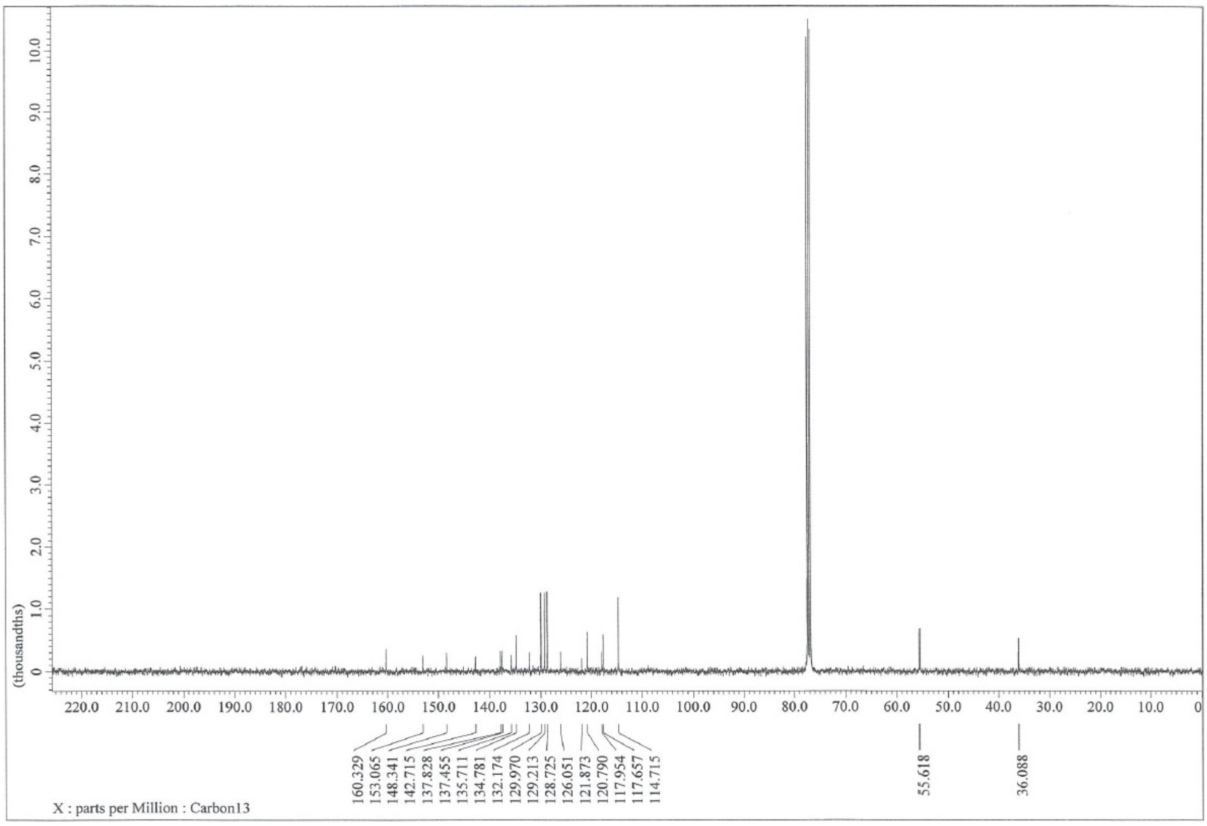
(thousandths)
10.0
9.0
8.0
7.0
6.0
5.0
4.0
3.0
2.0
1.0
0
220.0 210.0 200.0 190.0 180.0 170.0 160.0 150.0 140.0 130.0 120.0 110.0 100.0 90.0 80.0 70.0 60.0 50.0 40.0 30.0 20.0 10.0 0
160.329
153.065
148.341
142.715
137.828
137.455
135.711
134.781
132.174
129.970
129.213
128.725
126.051
121.873
120.790
117.954
117.657
114.715
55.618
36.088
X : parts per Million : Carbon13

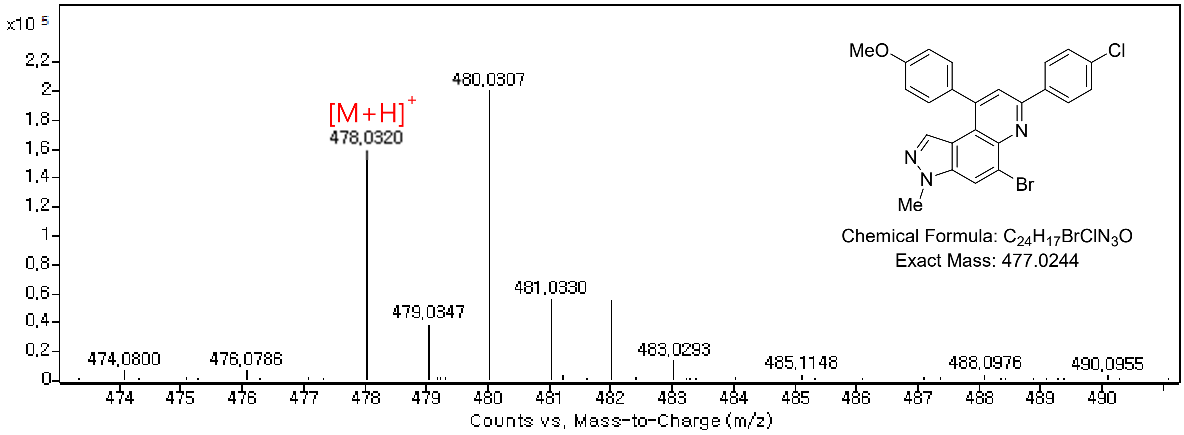
x10 5
2,2
2
1,8
1,6
1,4
1,2
1
0,8
0,6
0,4
0,2
0
[M+H]+
478,0320
480,0307
479,0347
481,0330
474,0800
476,0786
483,0293
485,1148
488,0976
490,0955
474
475
476
477
478
479
480
481
482
483
484
485
486
487
488
489
490
Counts vs. Mass-to-Charge (m/z)
MeO
Cl
N
N
N
Me
Br
Chemical Formula: C24H17BrClN3O
Exact Mass: 477.0244

5-Bomo-7-(4-bromophenyl)-9-(4-methoxyphenyl)-3-methyl-3H-pyrazolo[4,3-f]quinoline (1S)

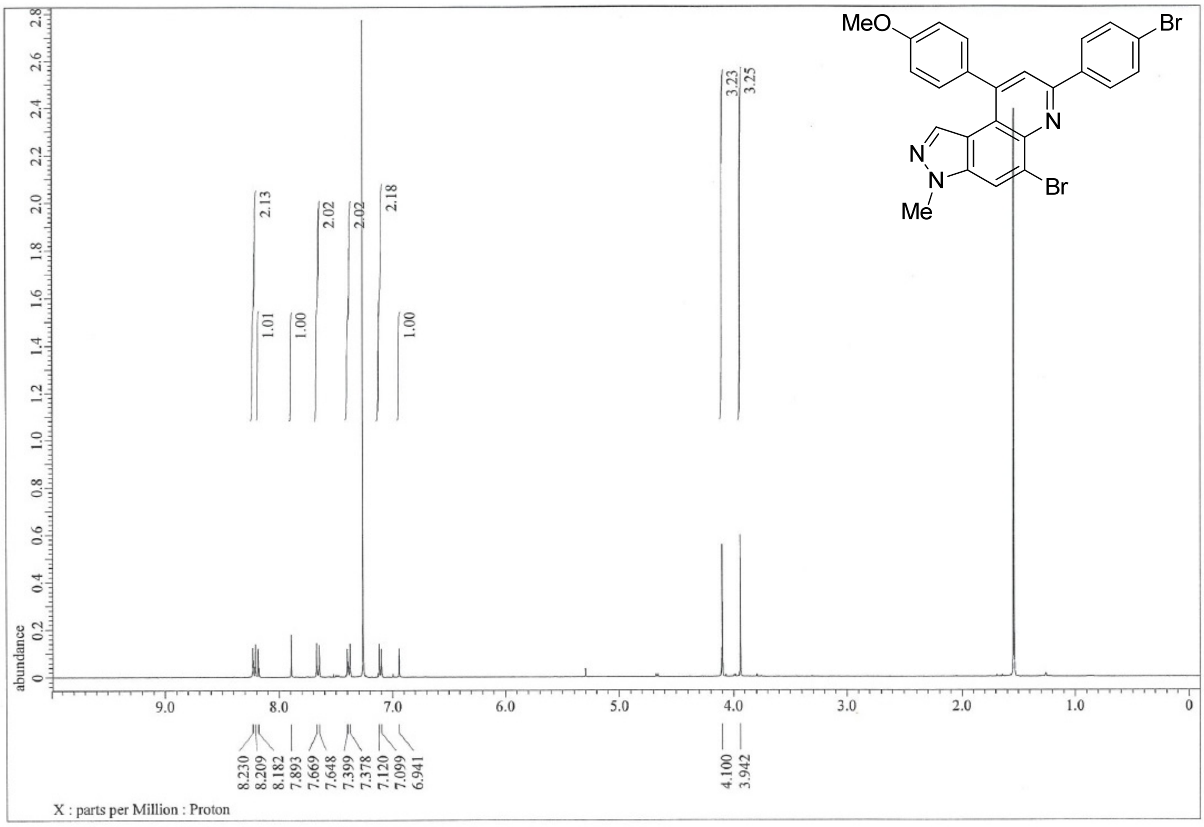

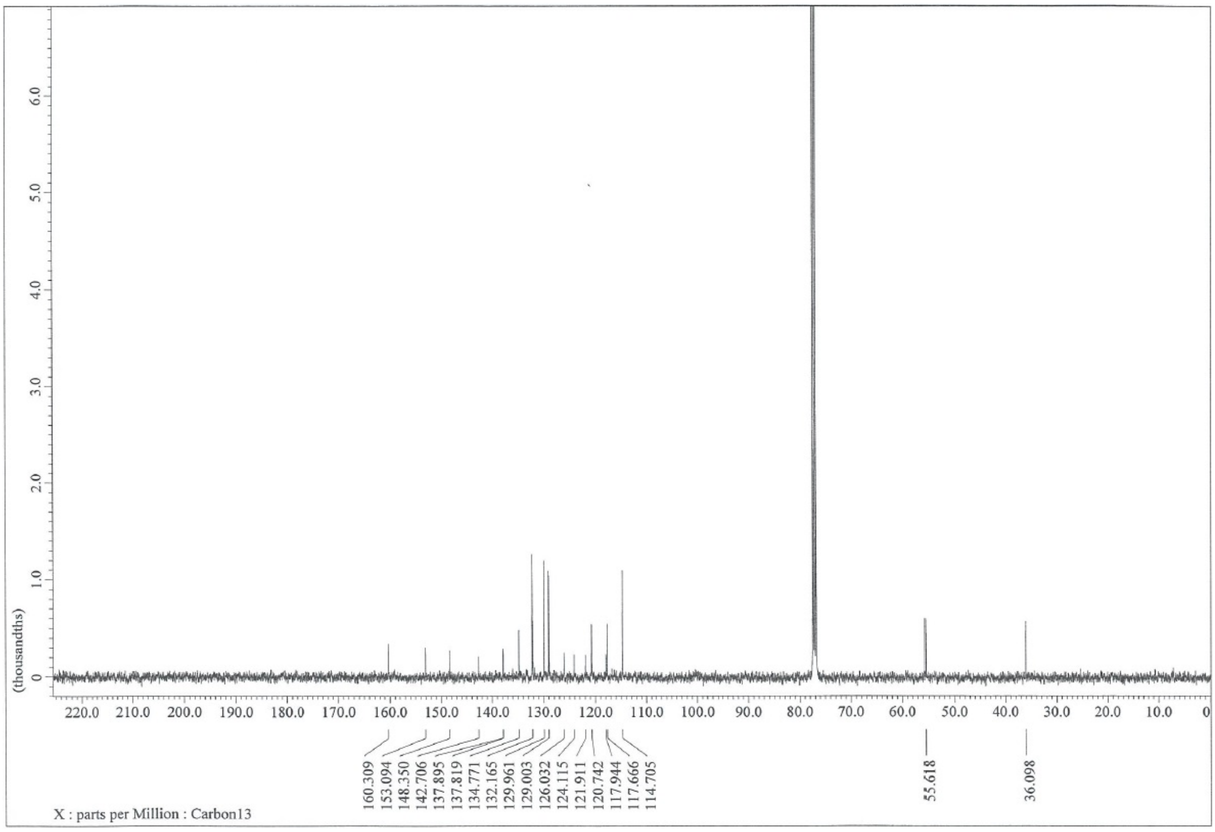
(thousandths)
6.0
5.0
4.0
3.0
2.0
1.0
0
220.0 210.0 200.0 190.0 180.0 170.0 160.0 150.0 140.0 130.0 120.0 110.0 100.0 90.0 80.0 70.0 60.0 50.0 40.0 30.0 20.0 10.0 0
160.309
153.094
148.350
142.706
137.895
137.819
134.771
132.165
129.961
129.003
126.032
124.115
121.911
120.742
117.944
117.666
114.705
55.618
36.098
X : parts per Million : Carbon13

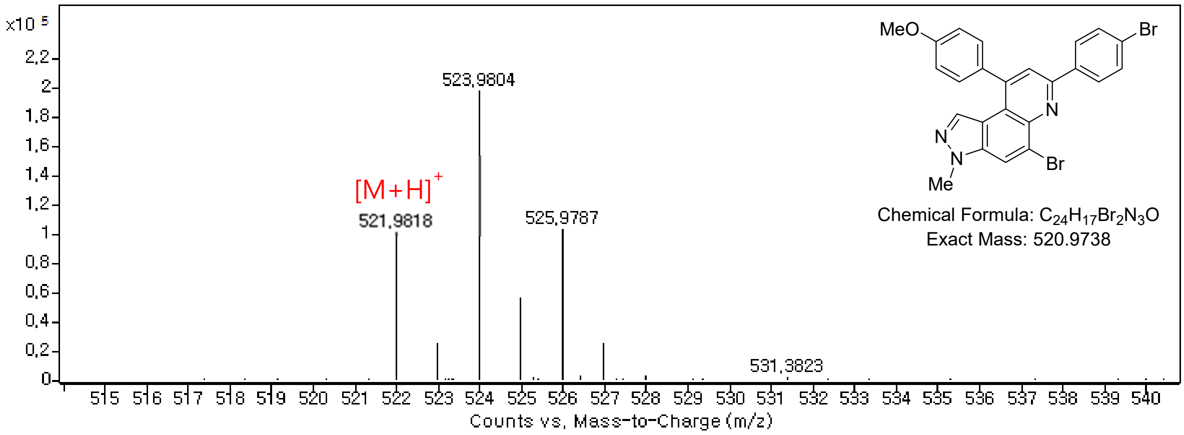
x10 5
2,2
2
1,8
1,6
1,4
1,2
1
0,8
0,6
0,4
0,2
0
523,9804
[M+H]+
521,9818
525,9787
531,3823
515 516 517 518 519 520 521 522 523 524 525 526 527 528 529 530 531 532 533 534 535 536 537 538 539 540
Counts vs. Mass-to-Charge (m/z)
MeO
Br
N
N
N
Br
Me
Chemical Formula: C24H17Br2N3O
Exact Mass: 520.9738

4-(5-Bomo-9-(4-methoxyphenyl)-3-methyl-3H-pyrazolo[4,3-f]quinolin-7-yl)-N,N-dimethylaniline (1T)

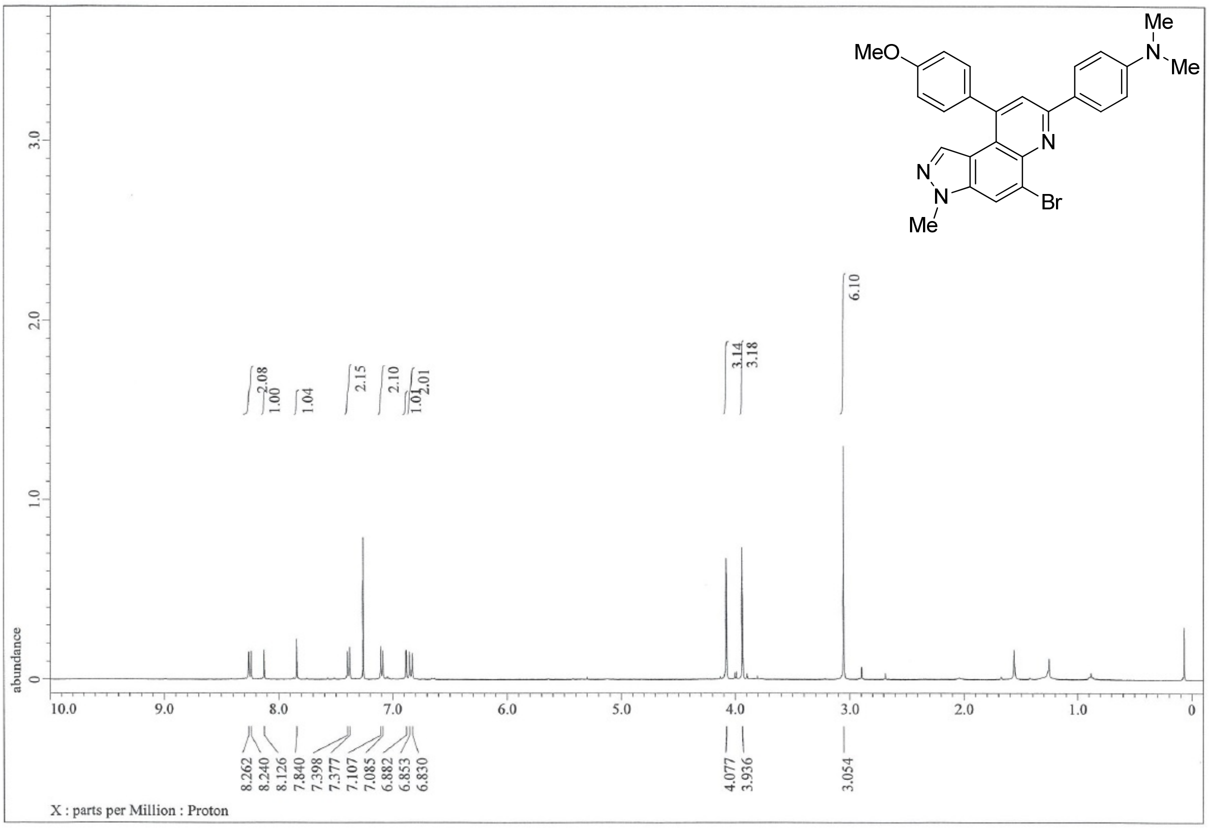

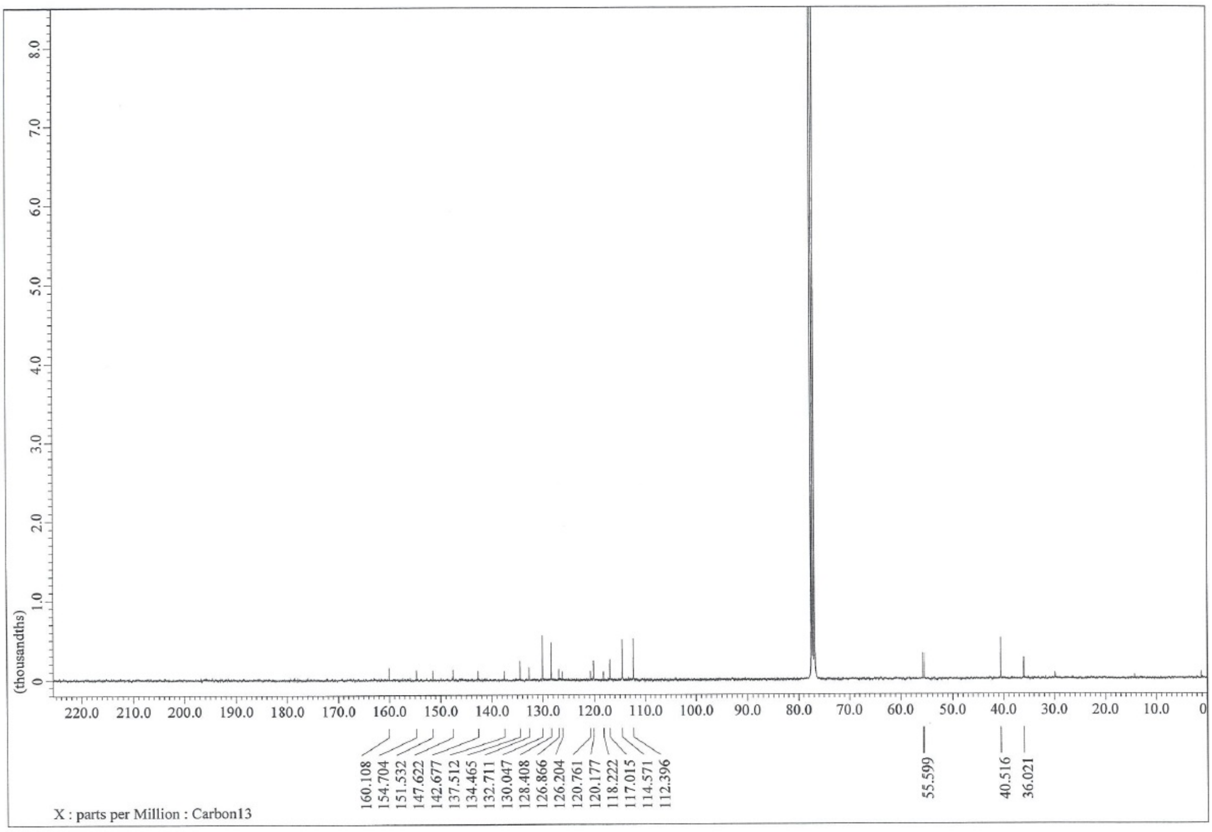
(thousandths)
X : parts per Million : Carbon13
160.108
154.704
151.532
147.622
142.677
137.512
134.465
132.711
130.047
128.408
126.866
126.204
120.761
120.177
118.222
117.015
114.571
112.396
55.599
40.516
36.021

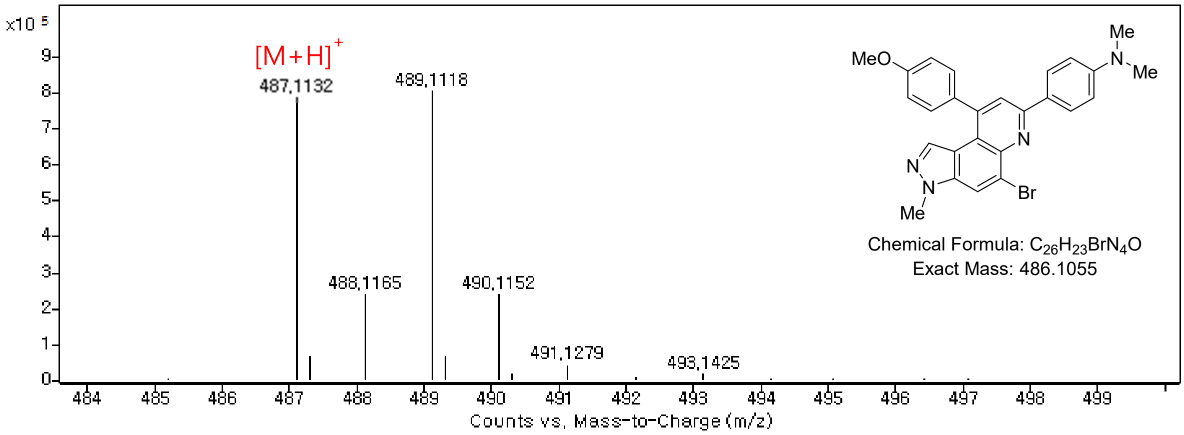
x10 5
[M+H]+
487.1132
489.1118
488.1165
490.1152
491.1279
493.1425
Counts vs. Mass-to-Charge (m/z)
MeO
Me
N
Me
N
N
N
Me
Br
Chemical Formula: C26H23BrN4O
Exact Mass: 486.1055

5-Bromo-3-methyl-3,12-dihydrochromeno[4,3-b]pyrazolo[4,3-f]quinoline (2A)

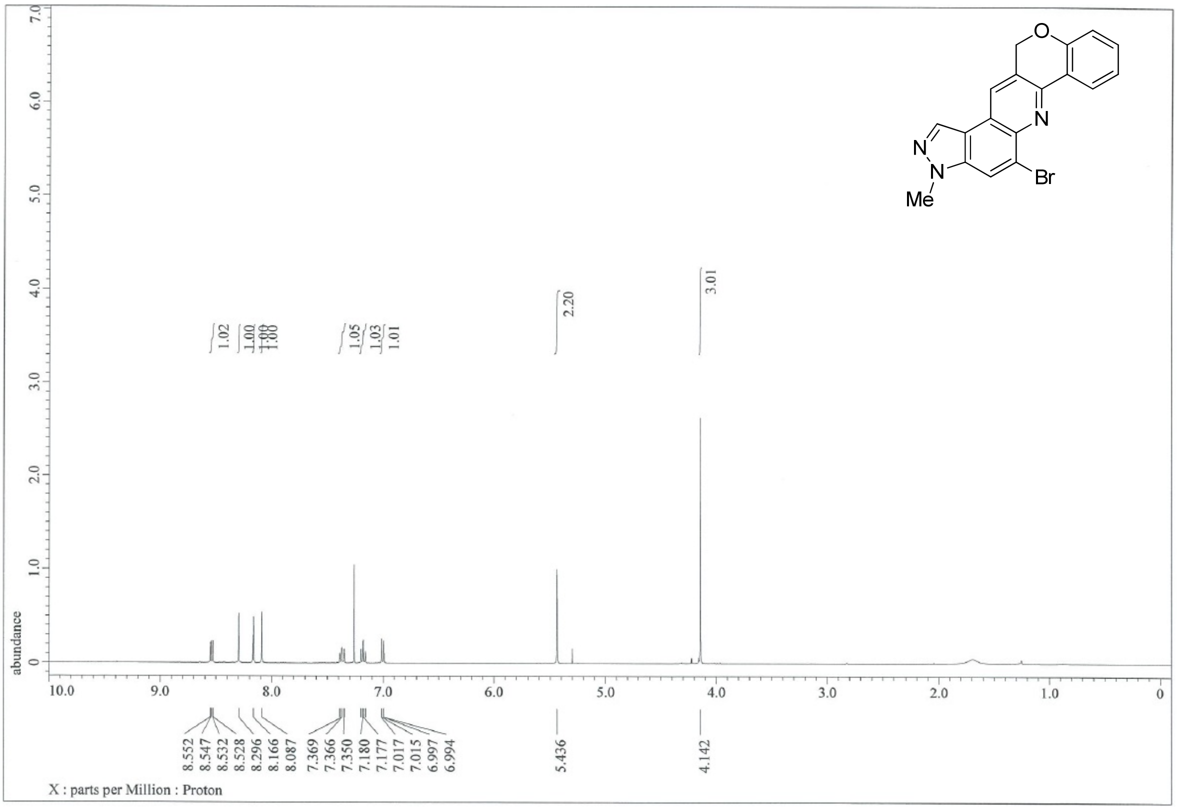

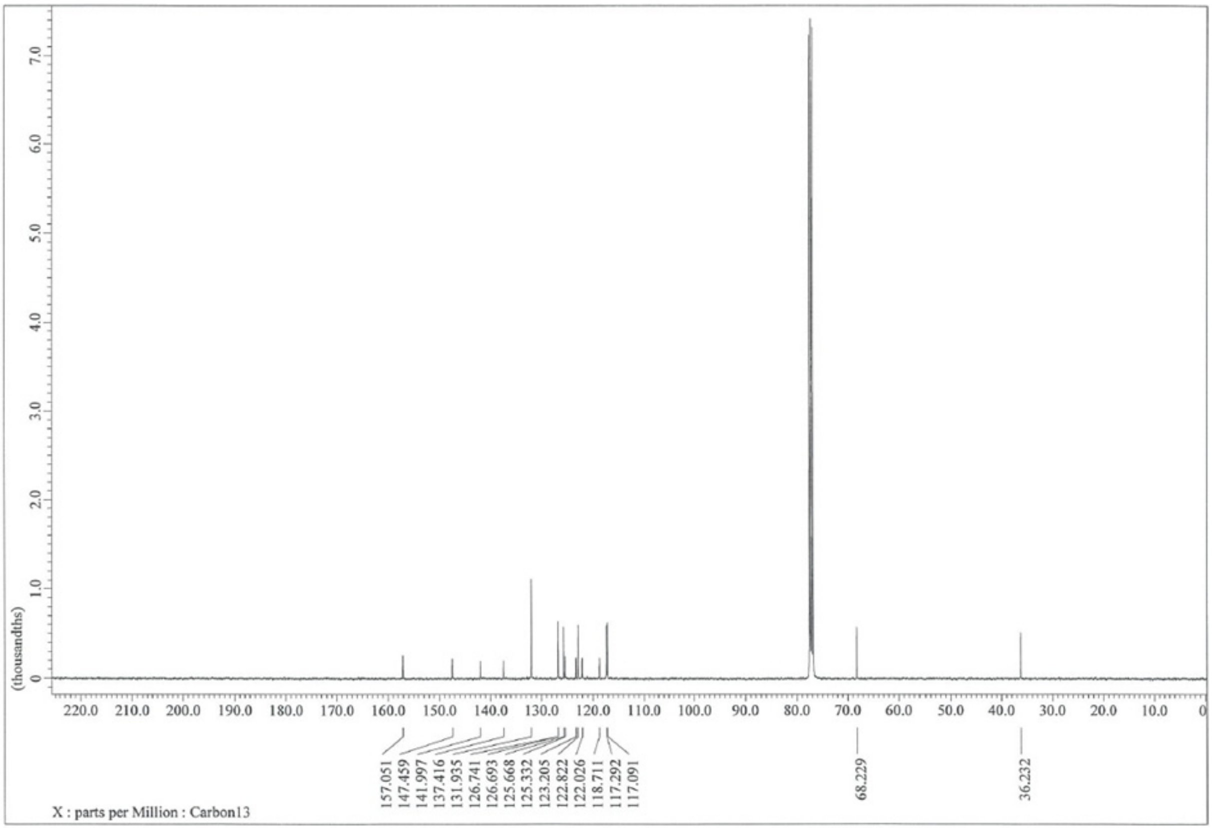

(thousandths)
7.0
6.0
5.0
4.0
3.0
2.0
1.0
0
220.0 210.0 200.0 190.0 180.0 170.0 160.0 150.0 140.0 130.0 120.0 110.0 100.0 90.0 80.0 70.0 60.0 50.0 40.0 30.0 20.0 10.0 0
157.051
147.459
141.997
137.416
131.935
126.741
126.693
125.668
125.332
123.205
122.822
122.026
118.711
117.292
117.091
68.229
36.232
X : parts per Million : Carbon13

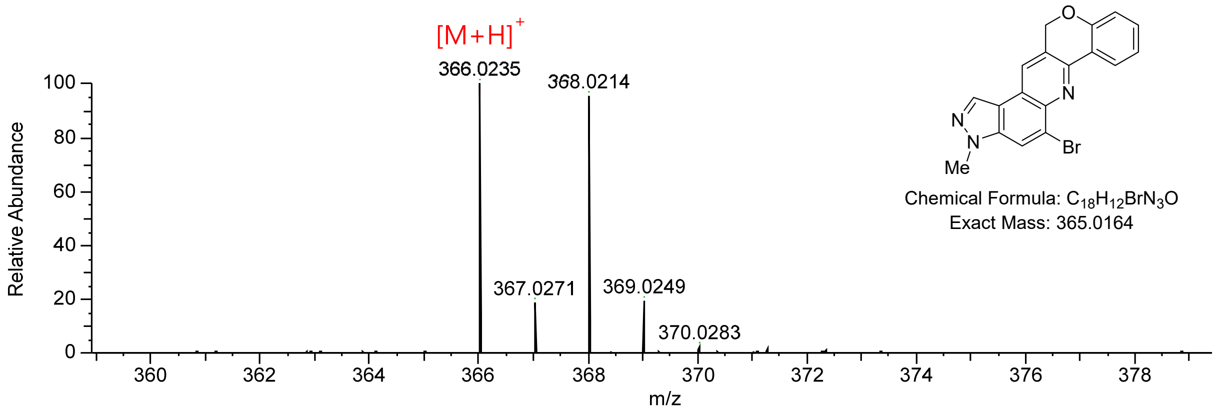

[M+H]+
366.0235
368.0214
367.0271
369.0249
370.0283
Relative Abundance
m/z
Me
Br
Chemical Formula: C18H12BrN3O
Exact Mass: 365.0164

5-Bromo-9-methoxy-3-methyl-3,12-dihydrochromeno[4,3-b]pyrazolo[4,3-f]quinoline (2B)

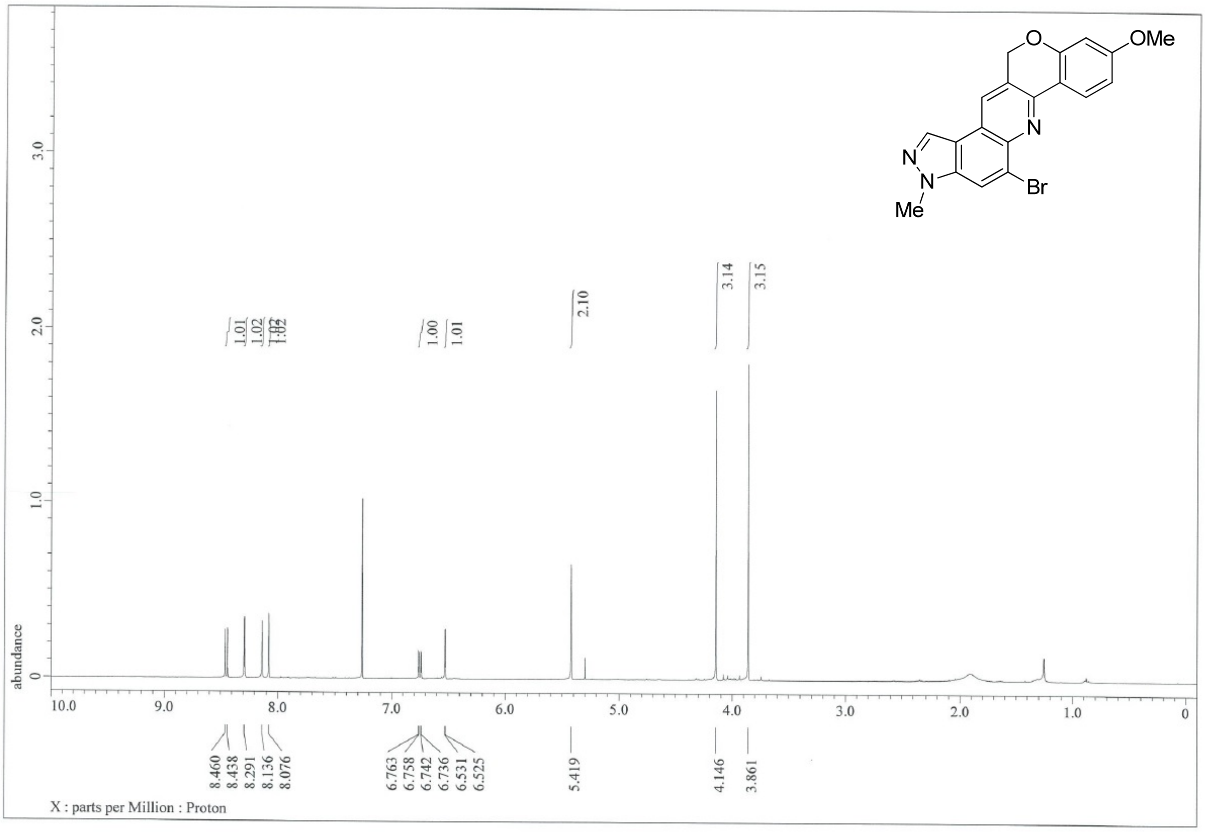

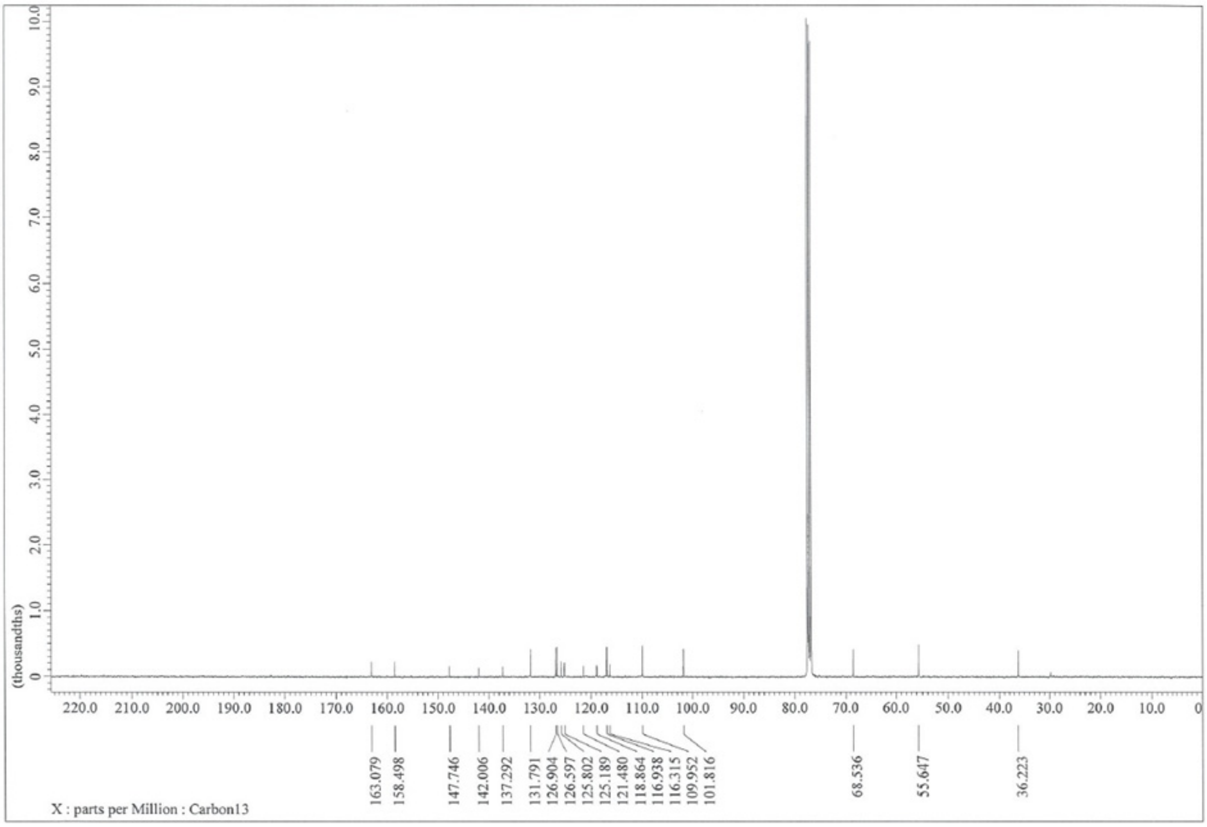
(thousandths)
X : parts per Million : Carbon13
163.079
158.498
147.746
142.006
137.292
131.791
126.904
126.597
125.802
125.189
121.480
118.864
116.938
116.315
109.952
101.816
68.536
55.647
36.223

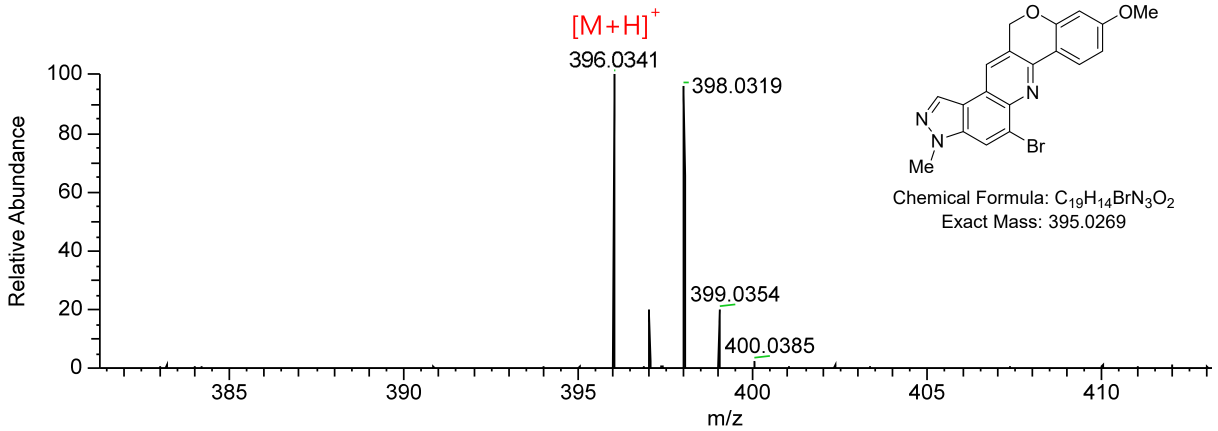
[M+H]+
396.0341
398.0319
399.0354
400.0385
100
80
60
40
20
0
Relative Abundance
385
390
395
400
405
410
m/z
OMe
O
N
N
N
Me
Br
Chemical Formula: C19H14BrN3O2
Exact Mass: 395.0269

5-Bromo-8-methoxy-3-methyl-3,12-dihydrochromeno[4,3-b]pyrazolo[4,3-f]quinoline (2C)

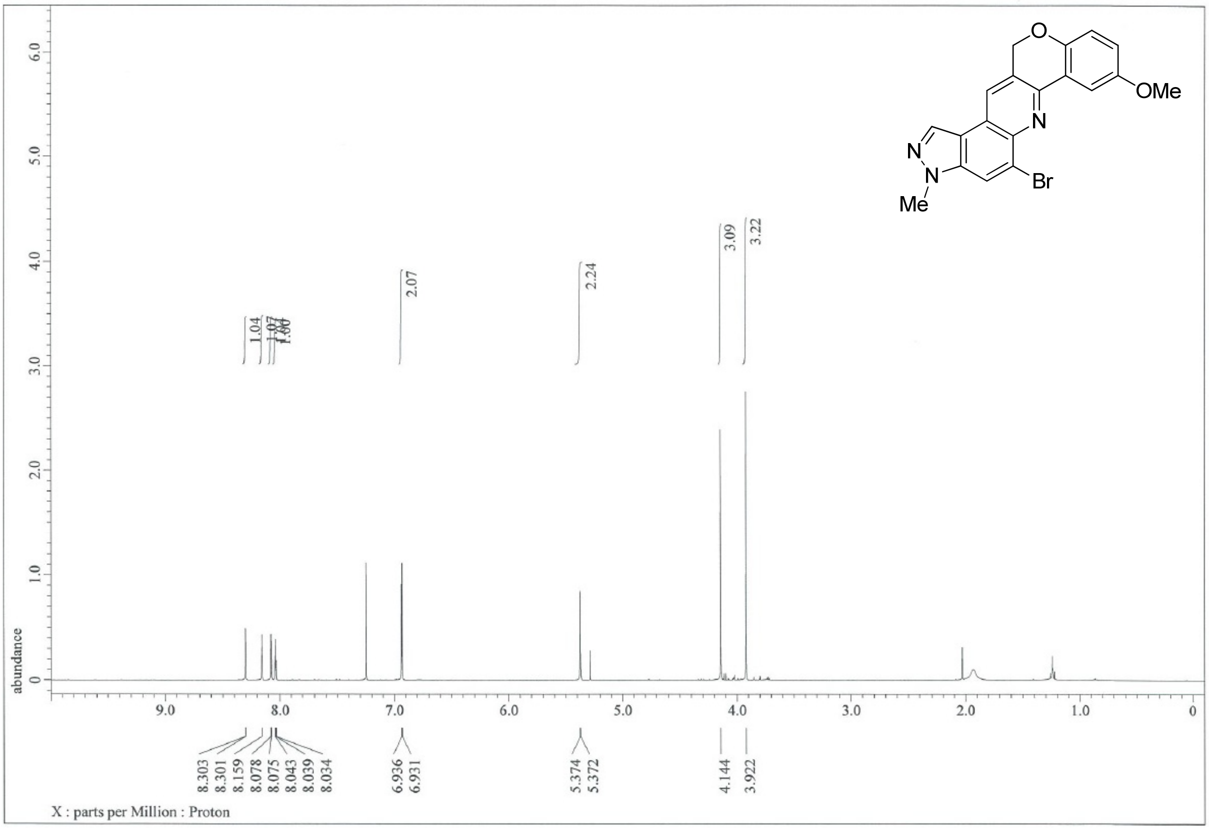

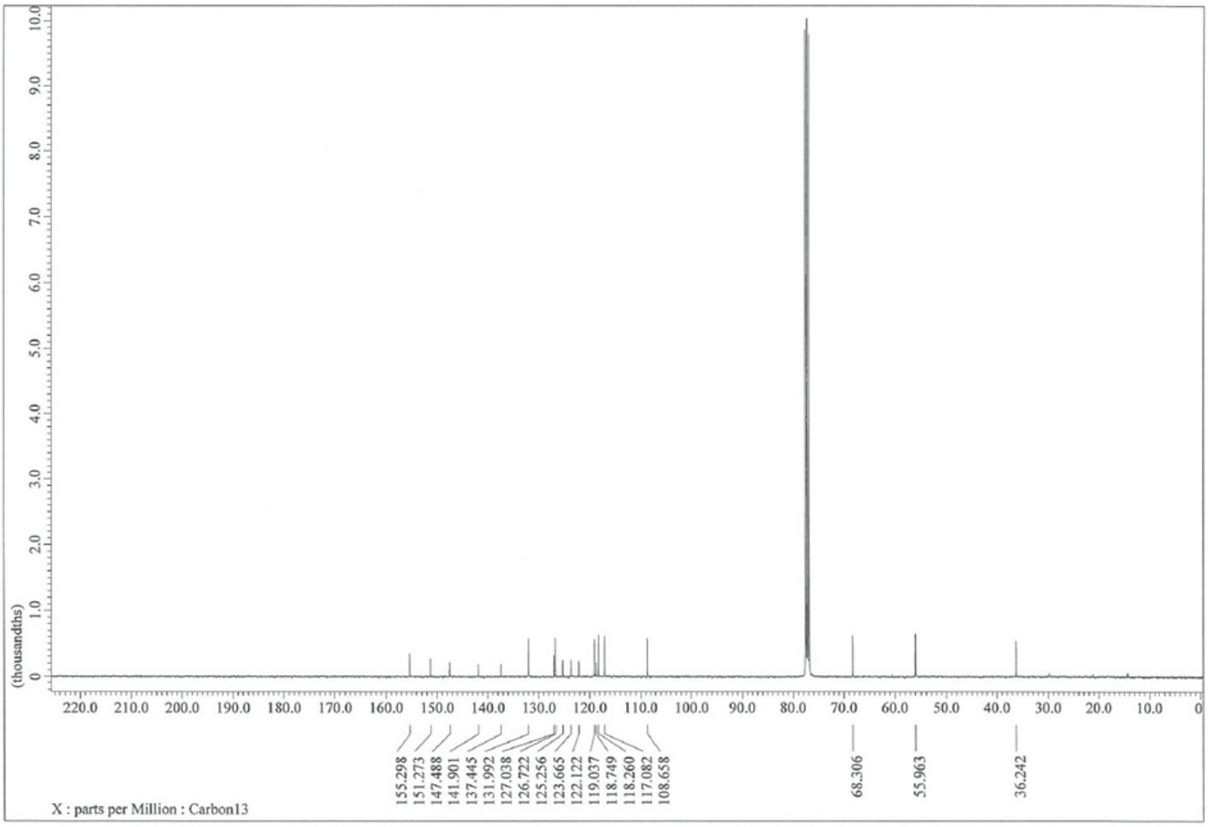
(thousandths)
10.0
9.0
8.0
7.0
6.0
5.0
4.0
3.0
2.0
1.0
0
220.0 210.0 200.0 190.0 180.0 170.0 160.0 150.0 140.0 130.0 120.0 110.0 100.0 90.0 80.0 70.0 60.0 50.0 40.0 30.0 20.0 10.0 0
155.298
151.273
147.488
141.901
137.445
131.992
127.038
126.722
125.256
123.665
122.122
119.037
118.749
118.260
117.082
108.658
68.306
55.963
36.242
X : parts per Million : Carbon13

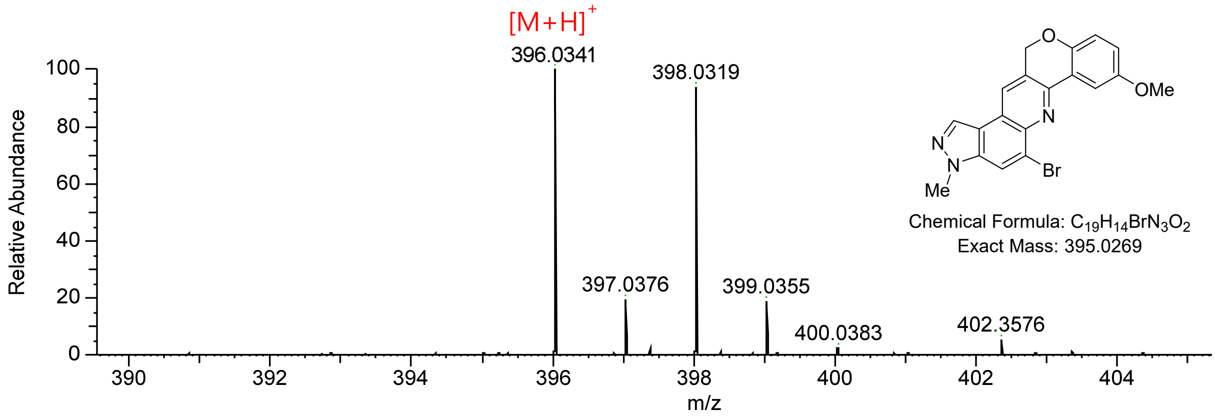
[M+H]+
396.0341
398.0319
397.0376
399.0355
400.0383
402.3576
Relative Abundance
m/z
OMe
N
N
N
Me
Br
O
Chemical Formula: C19H14BrN3O2
Exact Mass: 395.0269

5-Bromo-3,10-dimethyl-3,12-dihydrochromeno[4,3-b]pyrazolo[4,3-f]quinoline (2D)

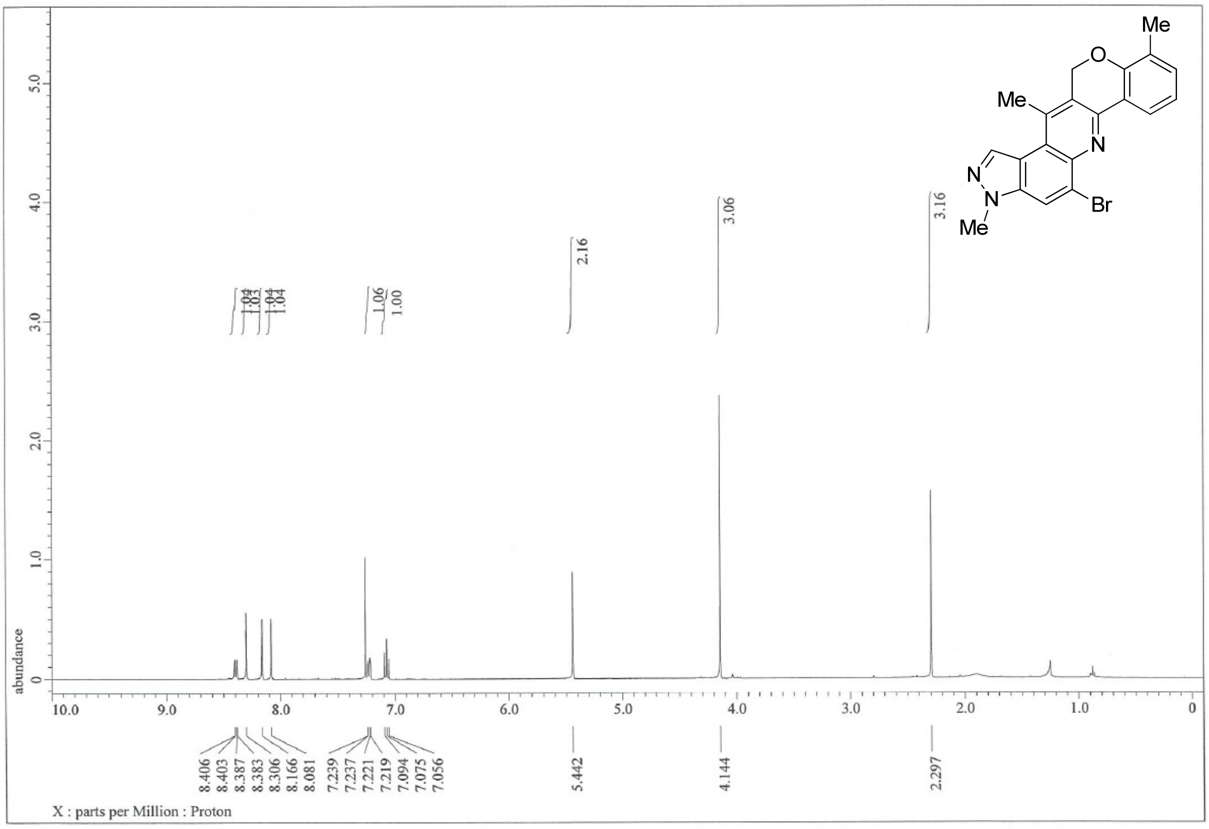

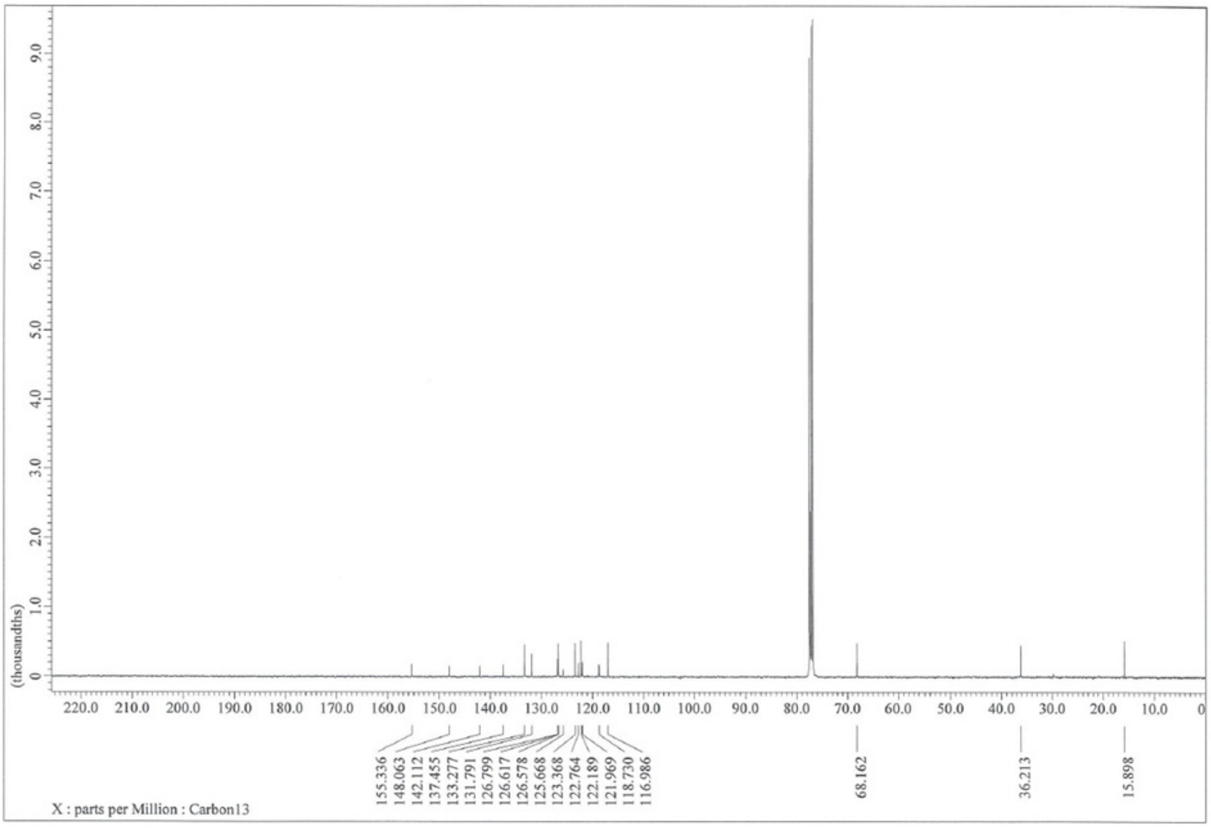
(thousandths)
9.0
8.0
7.0
6.0
5.0
4.0
3.0
2.0
1.0
0
220.0 210.0 200.0 190.0 180.0 170.0 160.0 150.0 140.0 130.0 120.0 110.0 100.0 90.0 80.0 70.0 60.0 50.0 40.0 30.0 20.0 10.0 0
155.336
148.063
142.112
137.455
133.277
131.791
126.799
126.617
126.578
125.668
123.368
122.764
122.189
121.969
118.730
116.986
68.162
36.213
15.898
X : parts per Million : Carbon13

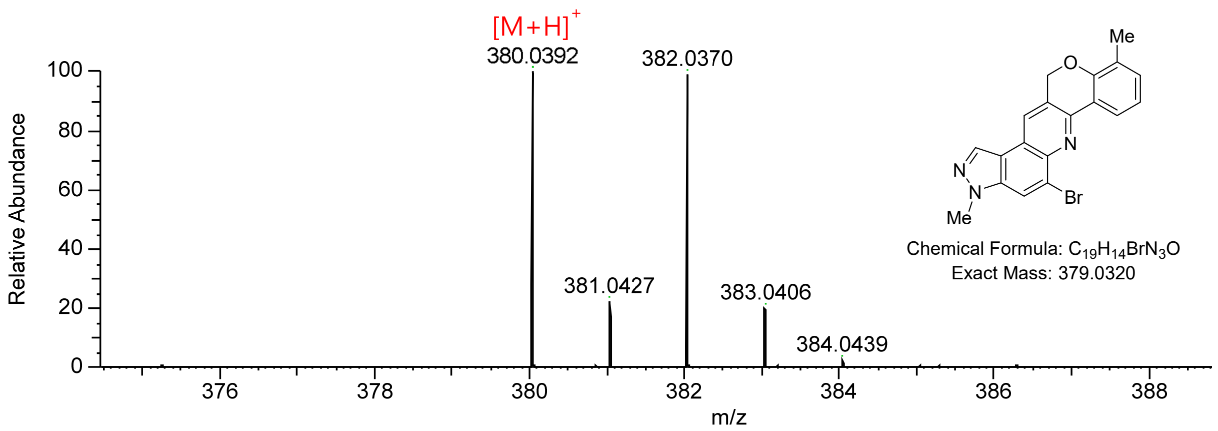

[M+H]+
380.0392
382.0370
381.0427
383.0406
384.0439
Relative Abundance
m/z
100
80
60
40
20
0
376
378
380
382
384
386
388
Me
O
N
N
N
Me
Br
Chemical Formula: C19H14BrN3O
Exact Mass: 379.0320

5-Bromo-7-fluoro-3-methyl-3,12-dihydrochromeno[4,3-b]pyrazolo[4,3-f]quinoline (2E)

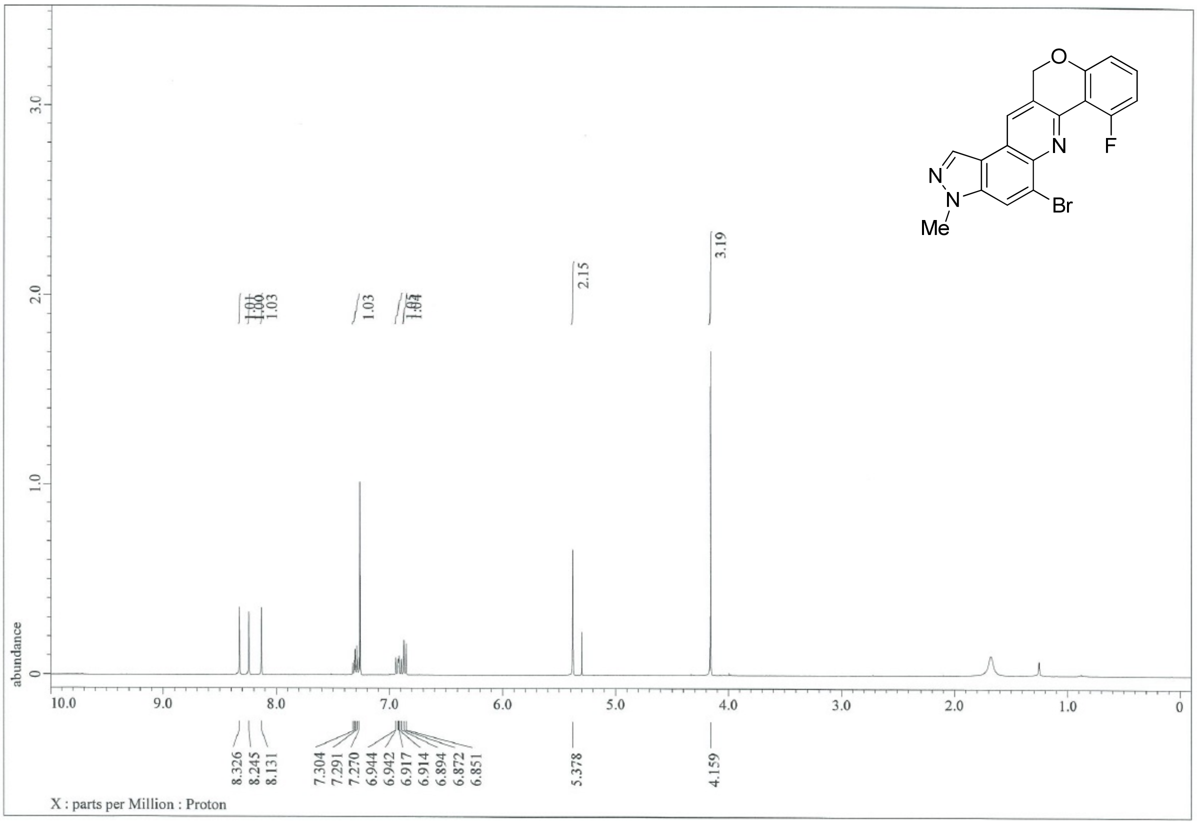

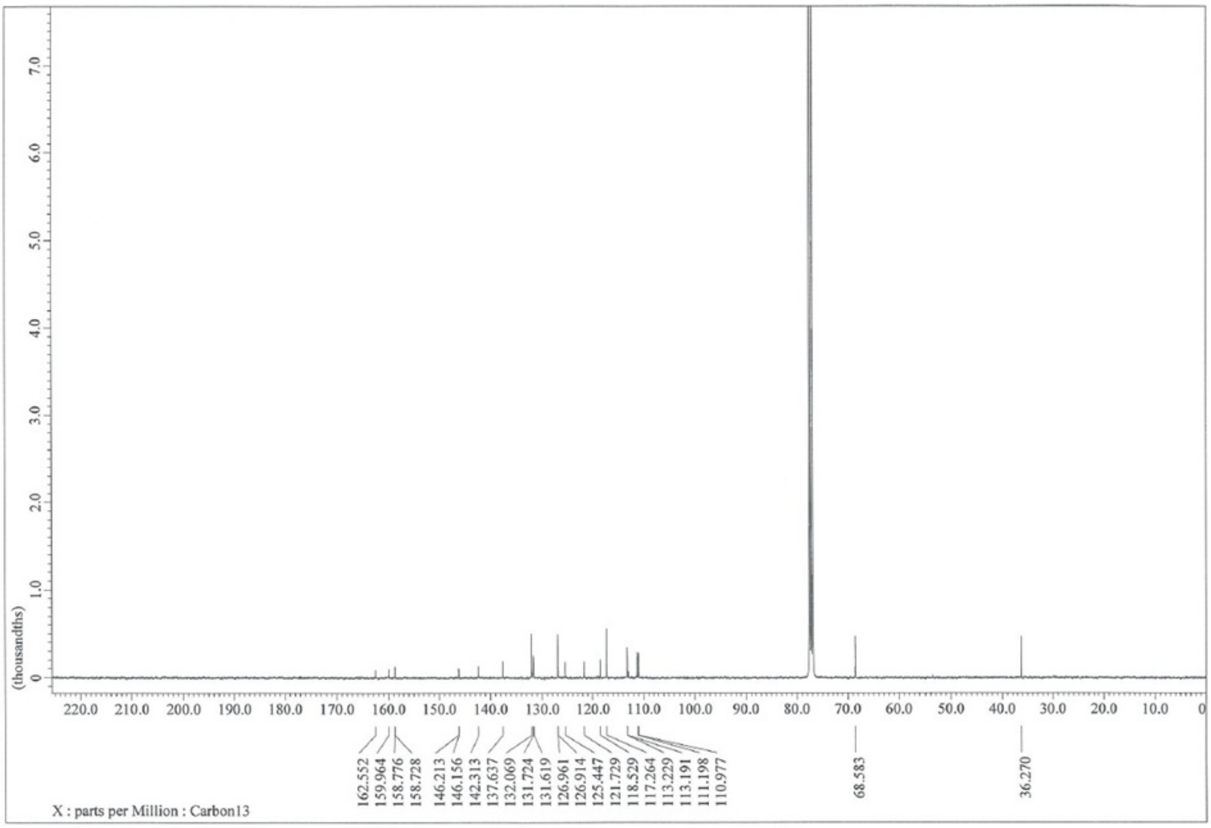

(thousandths)
0
1.0
2.0
3.0
4.0
5.0
6.0
7.0
220.0 210.0 200.0 190.0 180.0 170.0 160.0 150.0 140.0 130.0 120.0 110.0 100.0 90.0 80.0 70.0 60.0 50.0 40.0 30.0 20.0 10.0 0
162.552
159.964
158.776
158.728
146.213
146.156
142.313
137.637
132.069
131.724
131.619
126.961
126.914
125.447
121.729
118.529
117.264
113.229
113.191
111.198
110.977
68.583
36.270
X : parts per Million : Carbon13

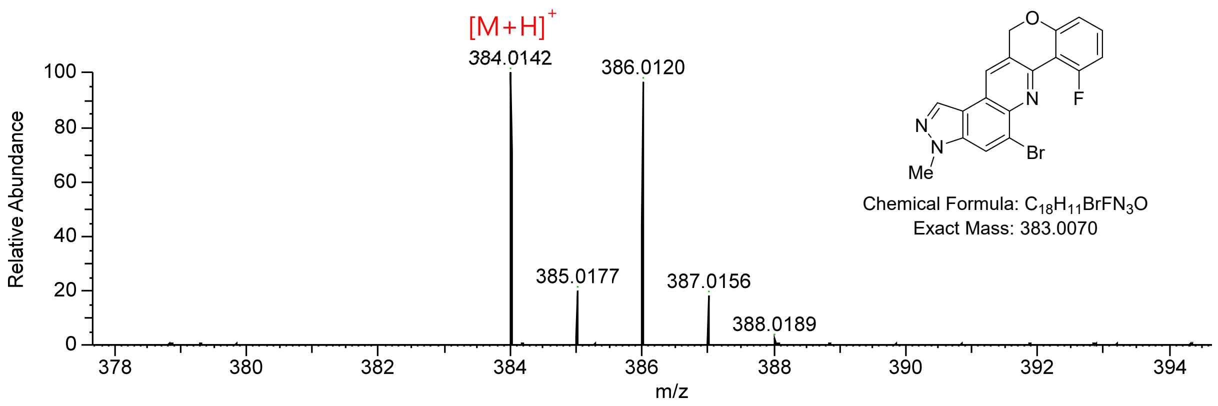

[M+H]+
384.0142
386.0120
385.0177
387.0156
388.0189
Relative Abundance
m/z
Chemical Formula: C18H11BrFN3O
Exact Mass: 383.0070

5-Bromo-8-fluoro-3-methyl-3,12-dihydrochromeno[4,3-b]pyrazolo[4,3-f]quinoline (2F)

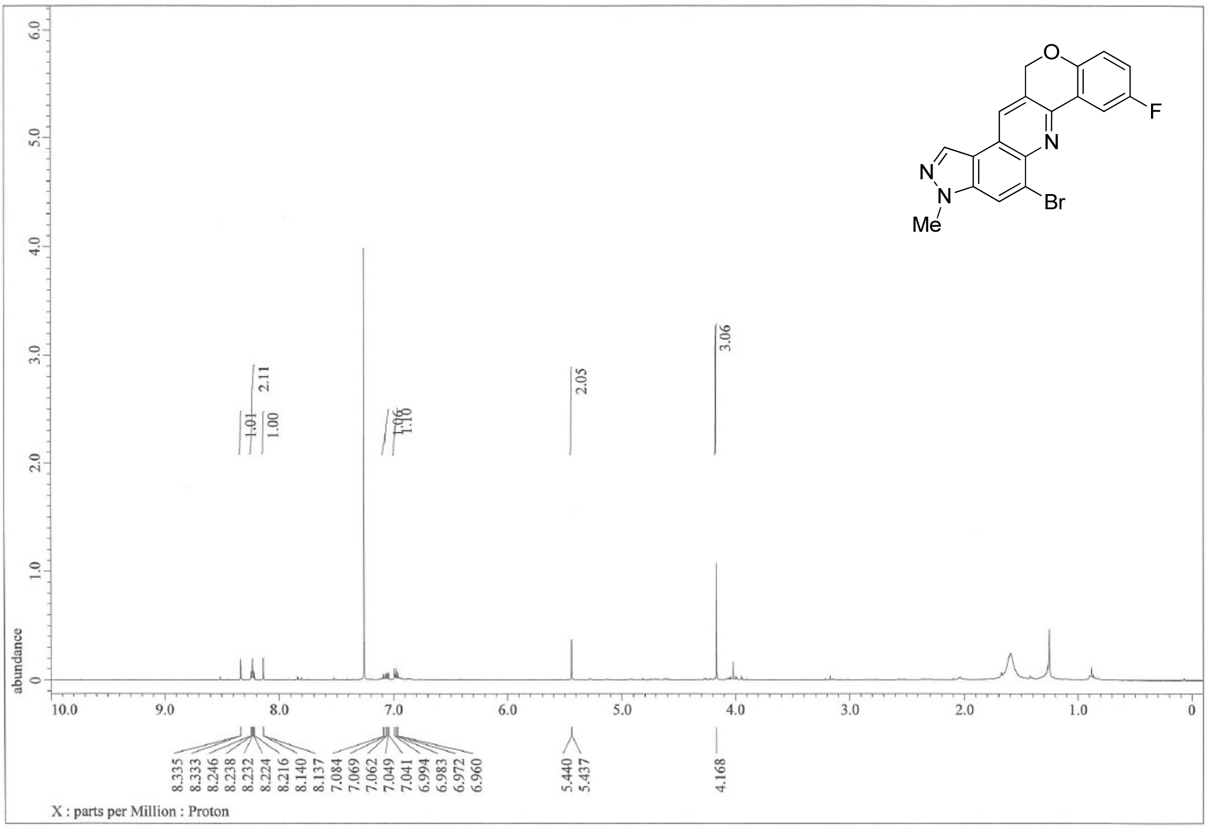

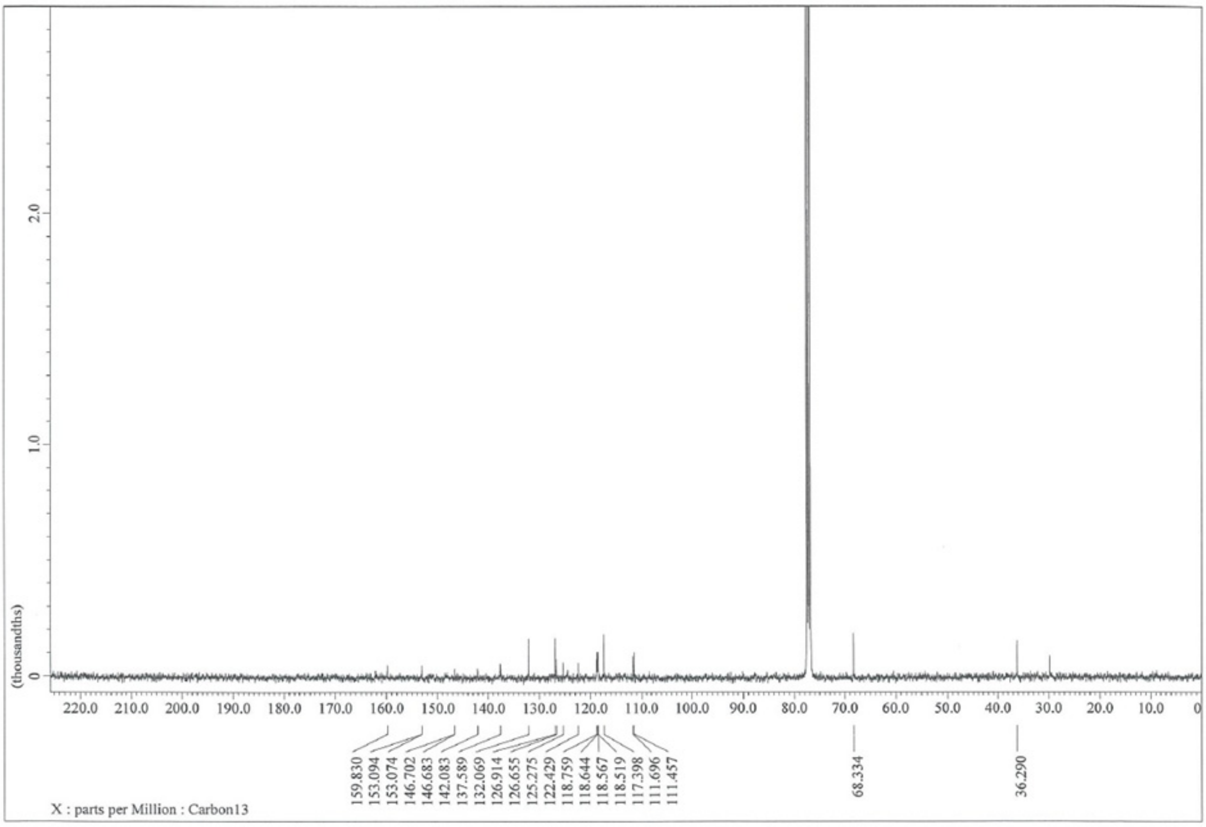

(thousandths)
X : parts per Million : Carbon13
159.830
153.094
153.074
146.702
146.683
142.083
137.589
132.069
126.914
126.655
125.275
122.429
118.759
118.644
118.567
118.519
117.398
111.696
111.457
68.334
36.290

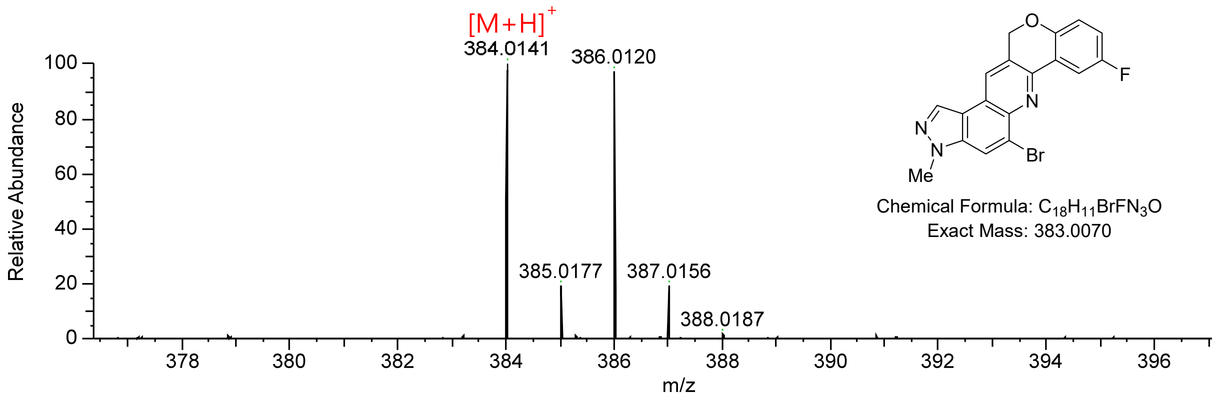
[M+H]+
384.0141
386.0120
385.0177
387.0156
388.0187
Relative Abundance
m/z
100
80
60
40
20
0
378
380
382
384
386
388
390
392
394
396
O
F
N
N
N
Me
Br
Chemical Formula: C18H11BrFN3O
Exact Mass: 383.0070

5-Bromo-9-fluoro-3-methyl-3,12-dihydrochromeno[4,3-b]pyrazolo[4,3-f]quinoline (2G)

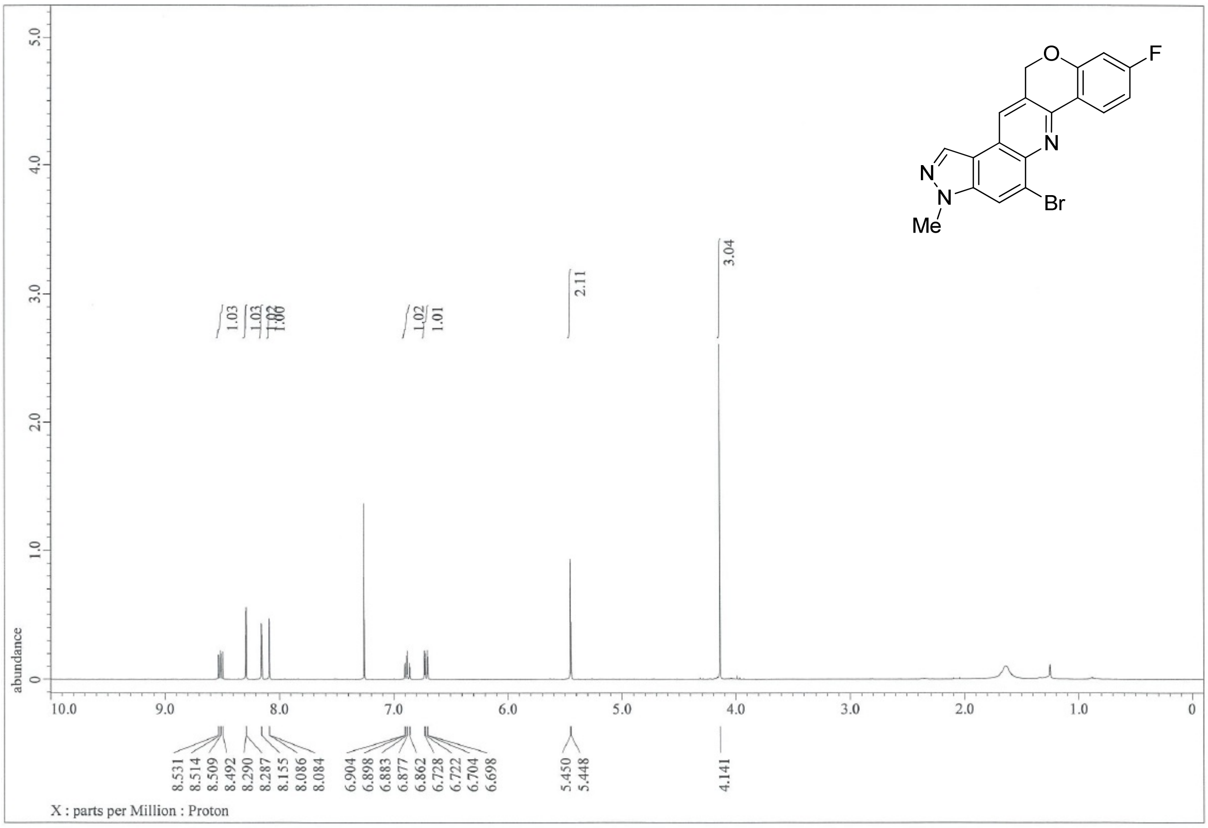

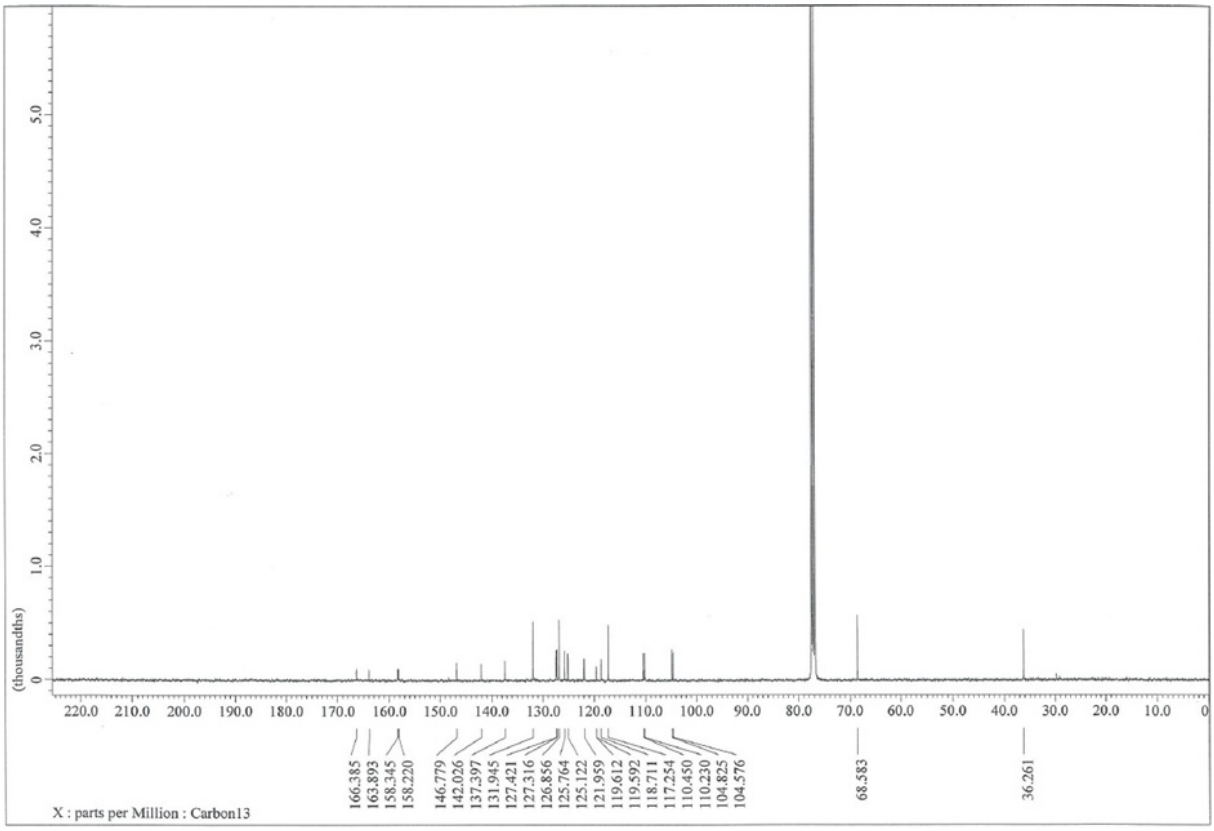
(thousandths)
0
1.0
2.0
3.0
4.0
5.0
220.0 210.0 200.0 190.0 180.0 170.0 160.0 150.0 140.0 130.0 120.0 110.0 100.0 90.0 80.0 70.0 60.0 50.0 40.0 30.0 20.0 10.0 0
166.385
163.893
158.345
158.220
146.779
142.026
137.397
131.945
127.421
127.316
126.856
125.764
125.122
121.959
119.612
119.592
118.711
117.254
110.450
110.230
104.825
104.576
68.583
36.261
X : parts per Million : Carbon13

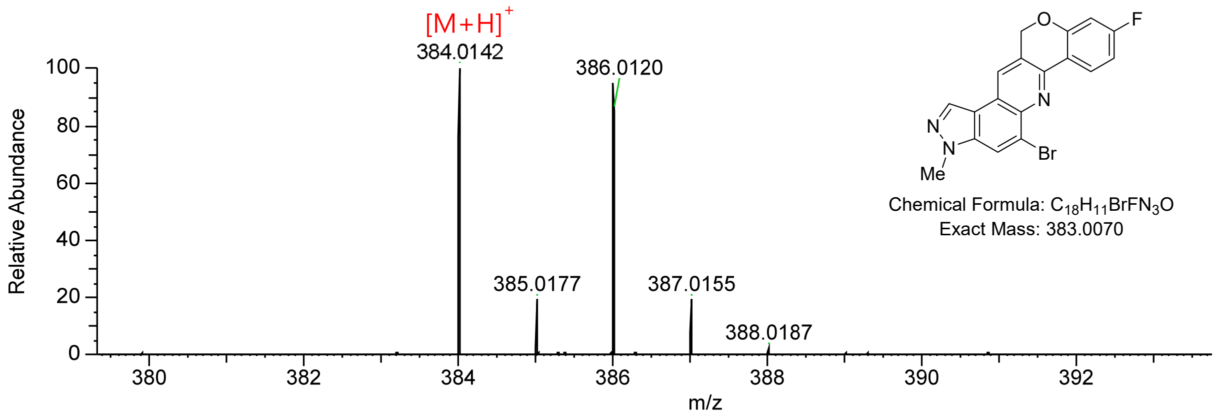
[M+H]+
384.0142
386.0120
385.0177
387.0155
388.0187
100
80
60
40
20
0
Relative Abundance
380
382
384
386
388
390
392
m/z
N
N
Me
N
O
F
Br
Chemical Formula: $C_{18}H_{11}BrFN_3O$
Exact Mass: 383.0070

5-Bromo-10-fluoro-3-methyl-3,12-dihydrochromeno[4,3-b]pyrazolo[4,3-f]quinoline (2H)

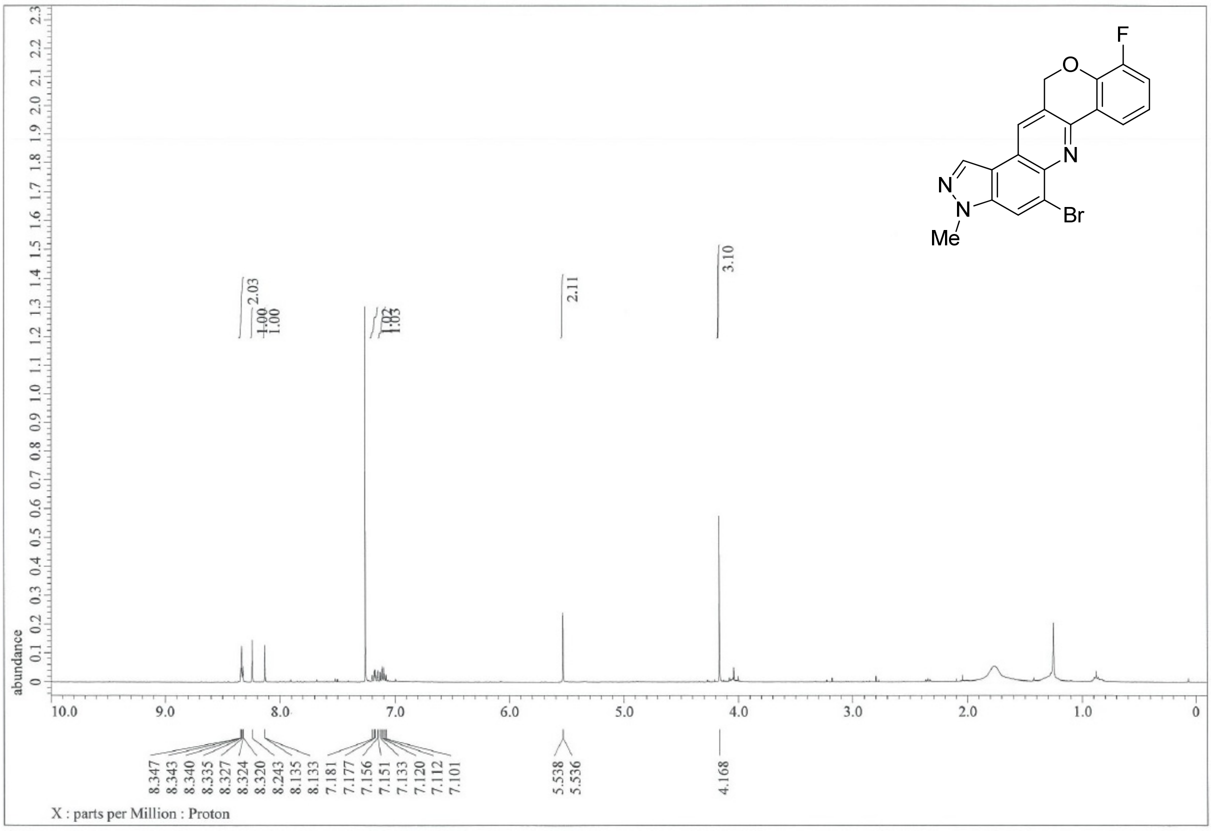

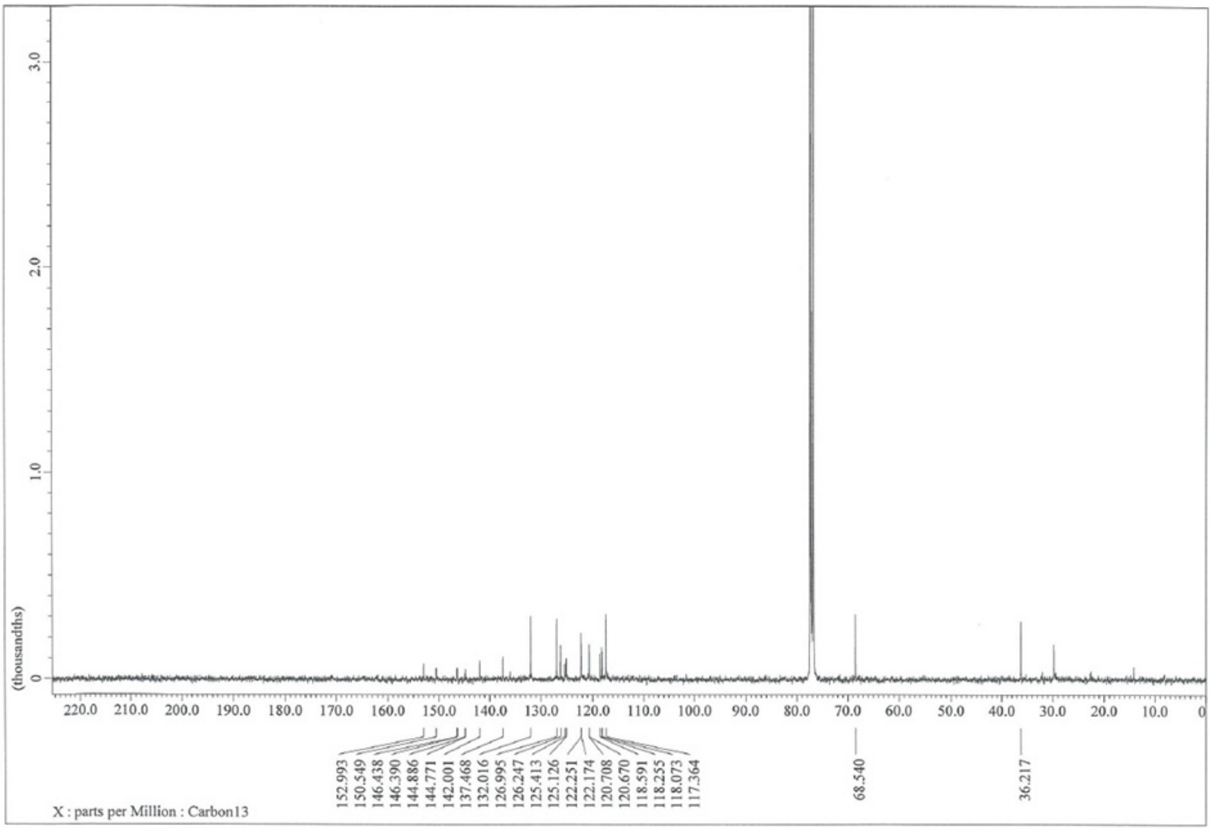

(thousandths)
3.0
2.0
1.0
0
220.0 210.0 200.0 190.0 180.0 170.0 160.0 150.0 140.0 130.0 120.0 110.0 100.0 90.0 80.0 70.0 60.0 50.0 40.0 30.0 20.0 10.0 0
152.993
150.549
146.438
146.390
144.886
144.771
142.001
137.468
132.016
126.995
126.247
125.413
125.126
122.251
122.174
120.708
120.670
118.591
118.255
118.073
117.364
68.540
36.217
X : parts per Million : Carbon13

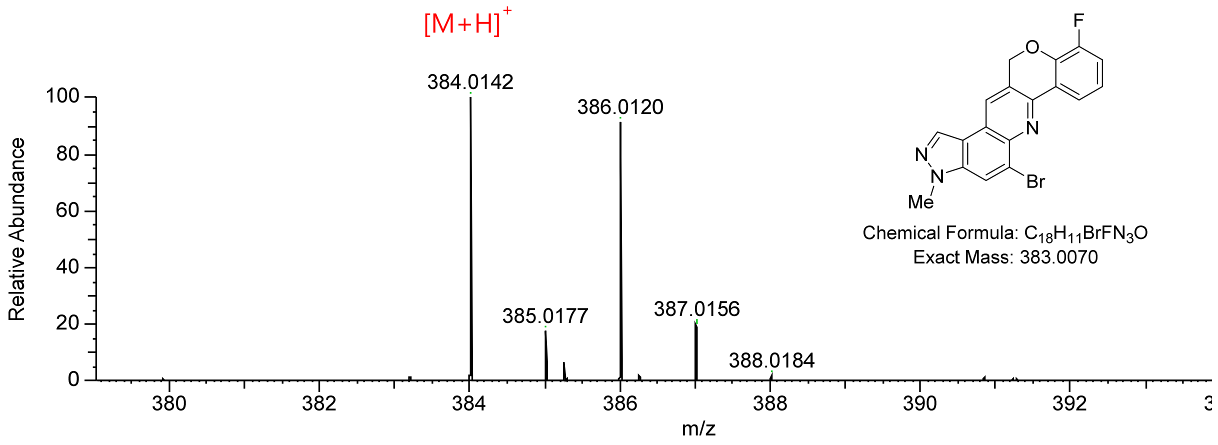
[M+H]+
384.0142
386.0120
385.0177
387.0156
388.0184
Relative Abundance
m/z
F
O
N
N
N
Me
Br
Chemical Formula: $C_{18}H_{11}BrFN_3O$
Exact Mass: 383.0070

5-Bromo-7-chloro-3-methyl-3,12-dihydrochromeno[4,3-b]pyrazolo[4,3-f]quinoline (2l)

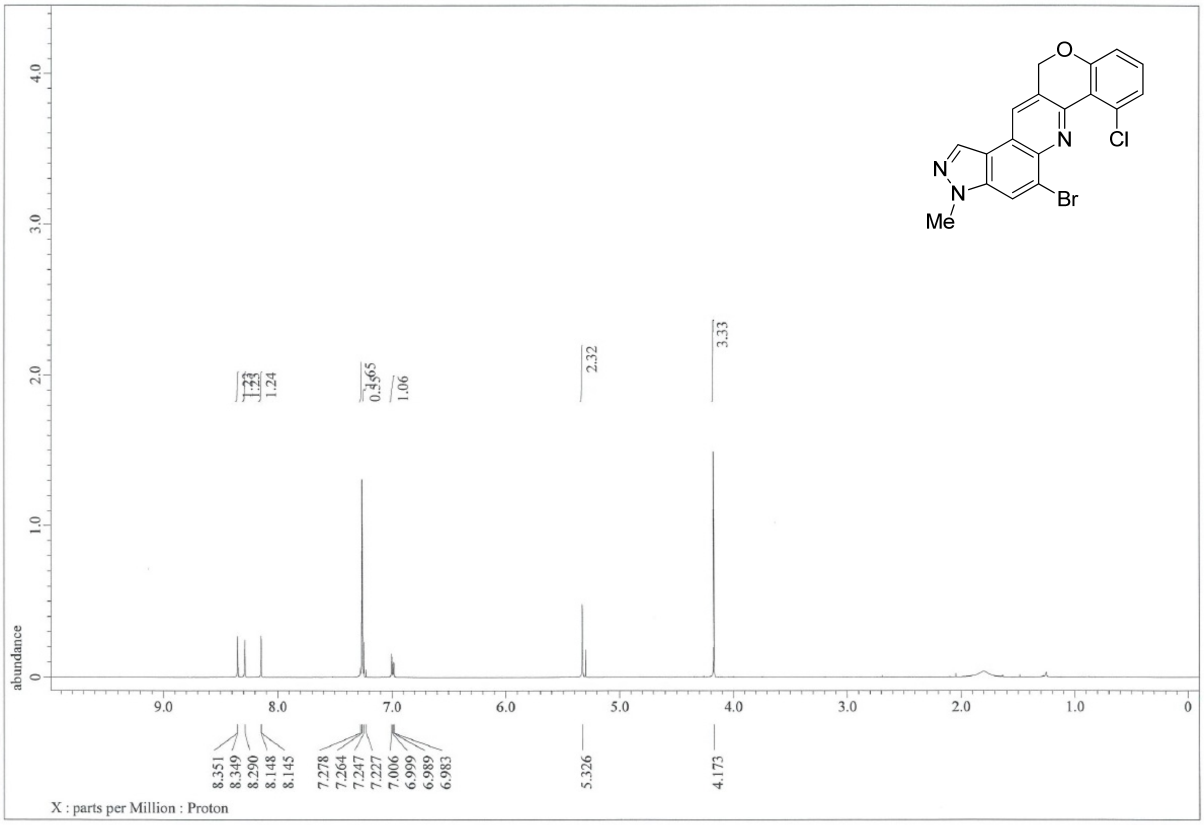

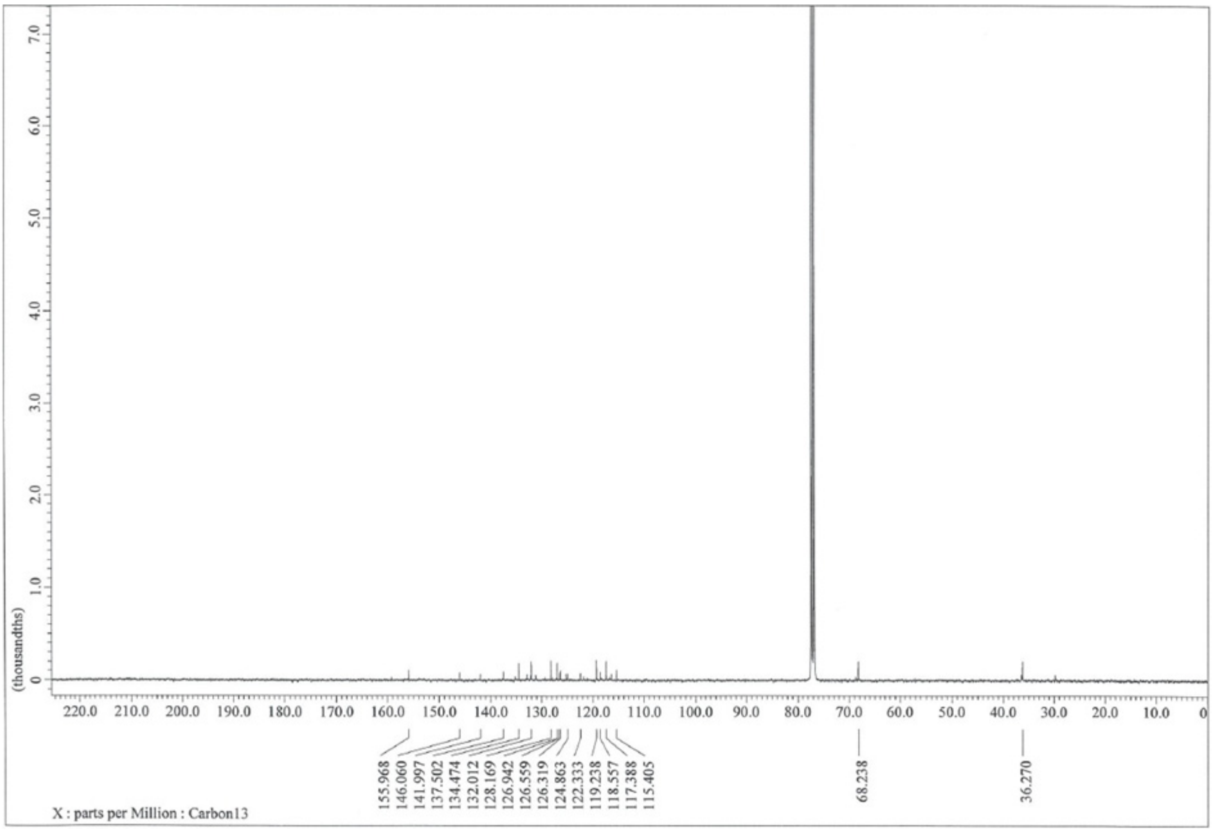
(thousandths)
0
1.0
2.0
3.0
4.0
5.0
6.0
7.0
220.0 210.0 200.0 190.0 180.0 170.0 160.0 150.0 140.0 130.0 120.0 110.0 100.0 90.0 80.0 70.0 60.0 50.0 40.0 30.0 20.0 10.0 0
155.968
146.060
141.997
137.502
134.474
132.012
128.169
126.942
126.559
126.319
124.863
122.333
119.238
118.557
117.388
115.405
68.238
36.270
X : parts per Million : Carbon13

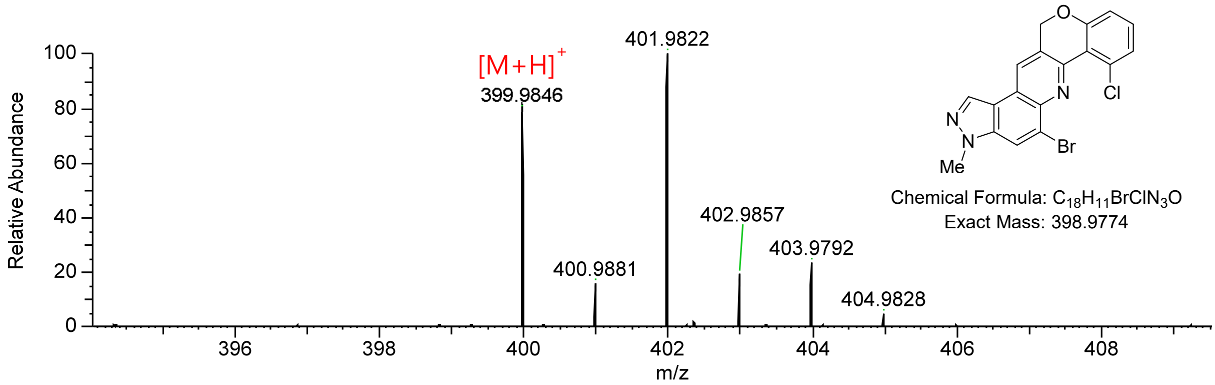

[M+H]+
399.9846
401.9822
400.9881
402.9857
403.9792
404.9828
Relative Abundance
m/z
N
Cl
Br
Me
Chemical Formula: C18H11BrClN3O
Exact Mass: 398.9774

5-Bromo-8-chloro-3-methyl-3,12-dihydrochromeno[4,3-b]pyrazolo[4,3-f]quinoline (2J)

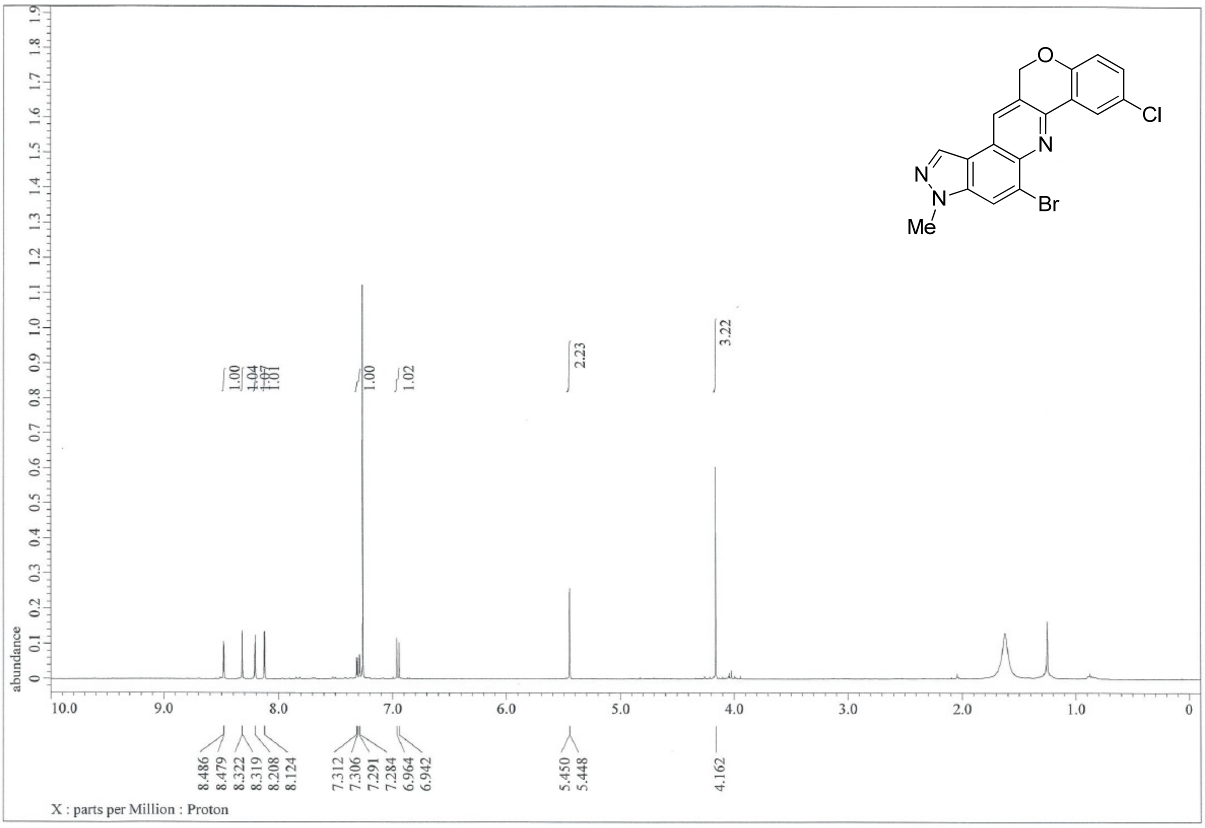

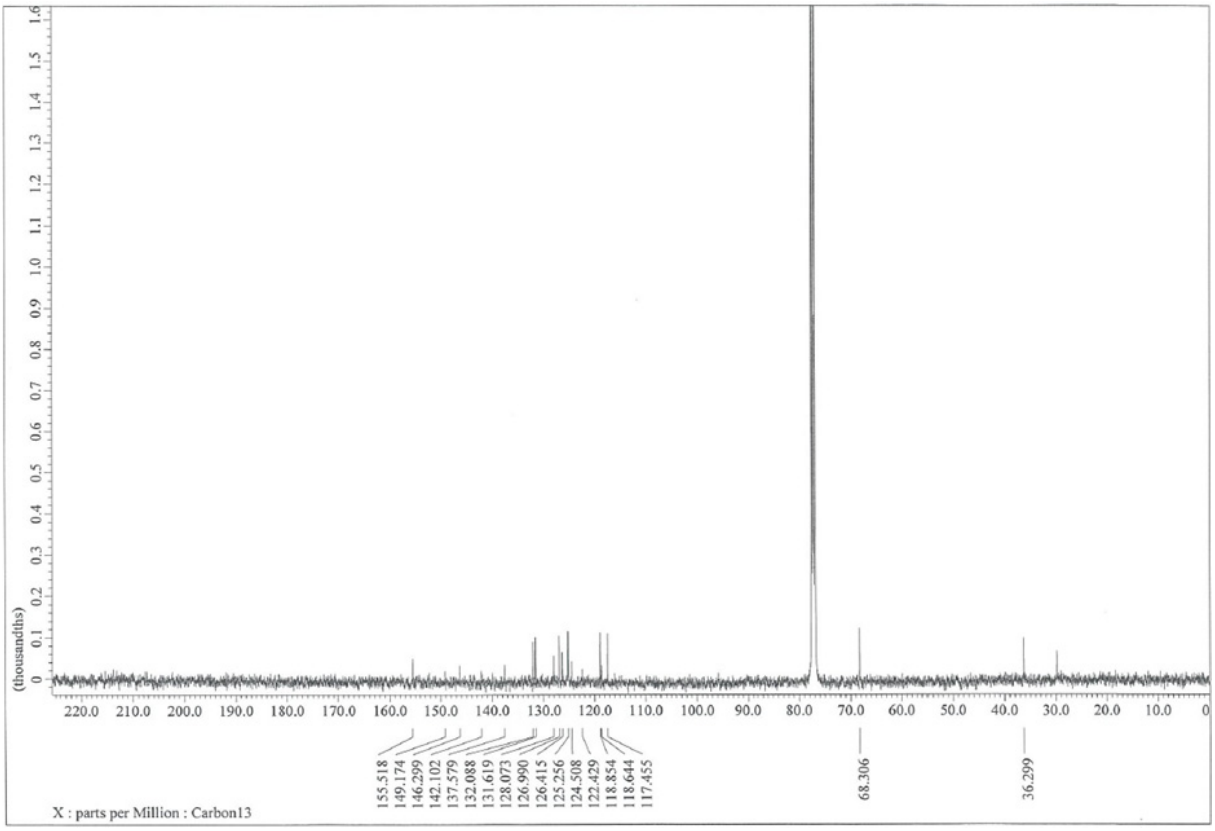

(thousandths)
X : parts per Million : Carbon13
155.518
149.174
146.299
142.102
137.579
132.088
131.619
128.073
126.990
126.415
125.256
124.508
122.429
118.854
118.644
117.455
68.306
36.299

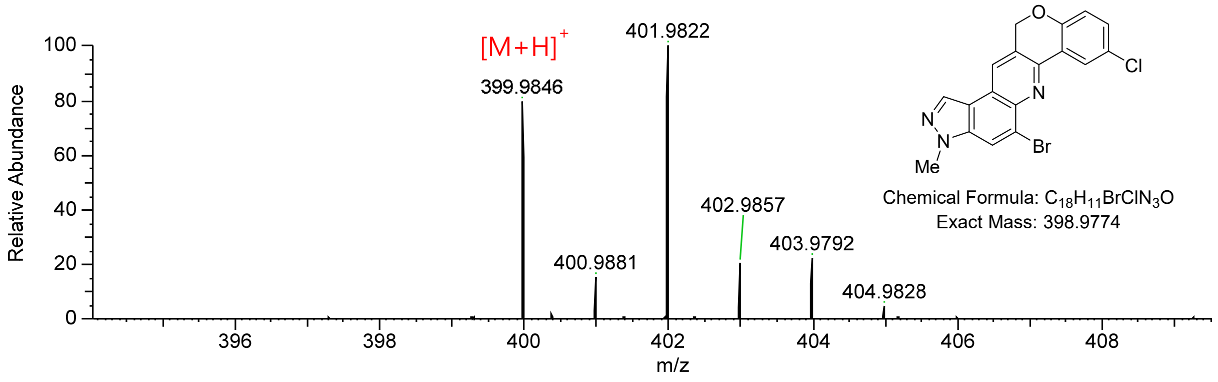

[M+H]+
399.9846
400.9881
401.9822
402.9857
403.9792
404.9828
Relative Abundance
m/z
0
20
40
60
80
100
396
398
400
402
404
406
408
N
N
Me
N
O
Cl
Br
Chemical Formula: C18H11BrClN3O
Exact Mass: 398.9774

5-Bromo-9-chloro-3-methyl-3,12-dihydrochromeno[4,3-b]pyrazolo[4,3-f] quinoline (2K)

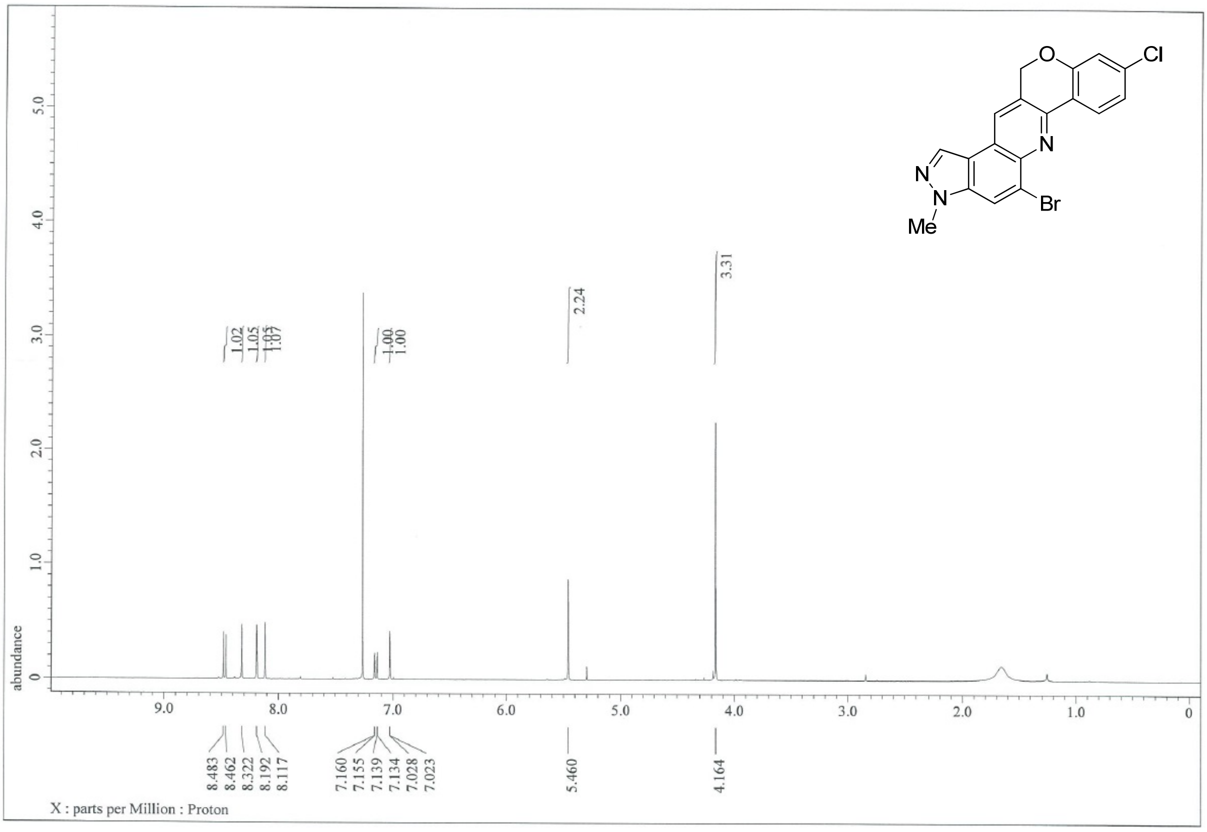

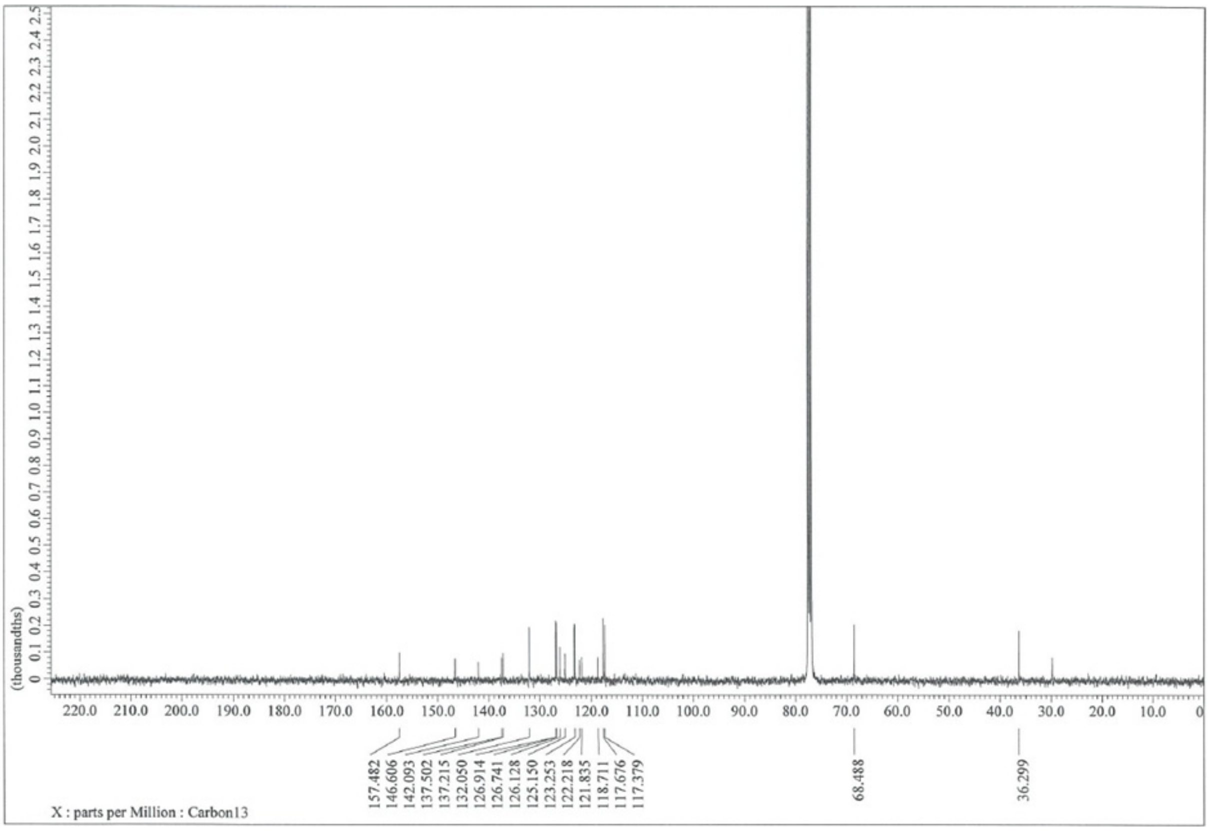
(thousandths)
X : parts per Million : Carbon13
157.482
146.606
142.093
137.502
137.215
132.050
126.914
126.741
126.128
125.150
123.253
122.218
121.835
118.711
117.676
117.379
68.488
36.299

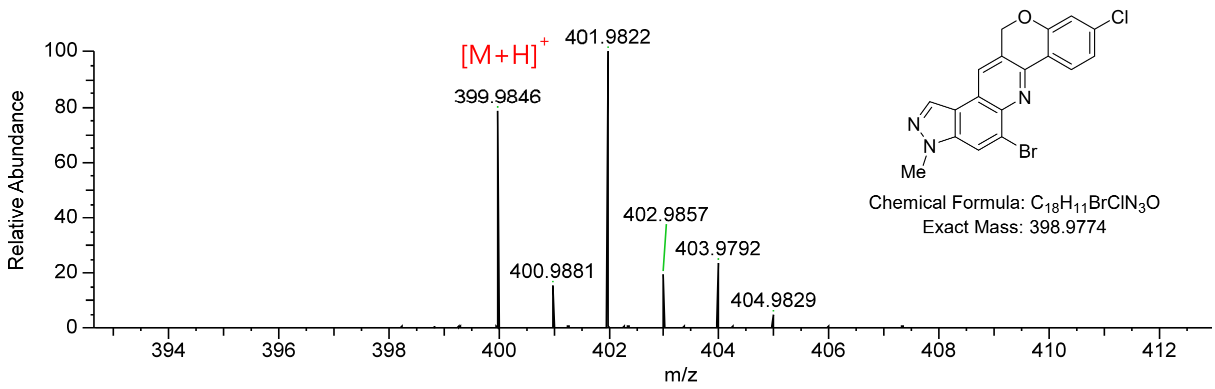
[M+H]+
401.9822
399.9846
400.9881
402.9857
403.9792
404.9829
Relative Abundance
m/z
Chemical Formula: C18H11BrClN3O
Exact Mass: 398.9774
N
N
Me
Br
O
Cl

5-Bromo-10-chloro-3-methyl-3,12-dihydrochromeno[4,3-b]pyrazolo[4,3-f]quinoline (2L)

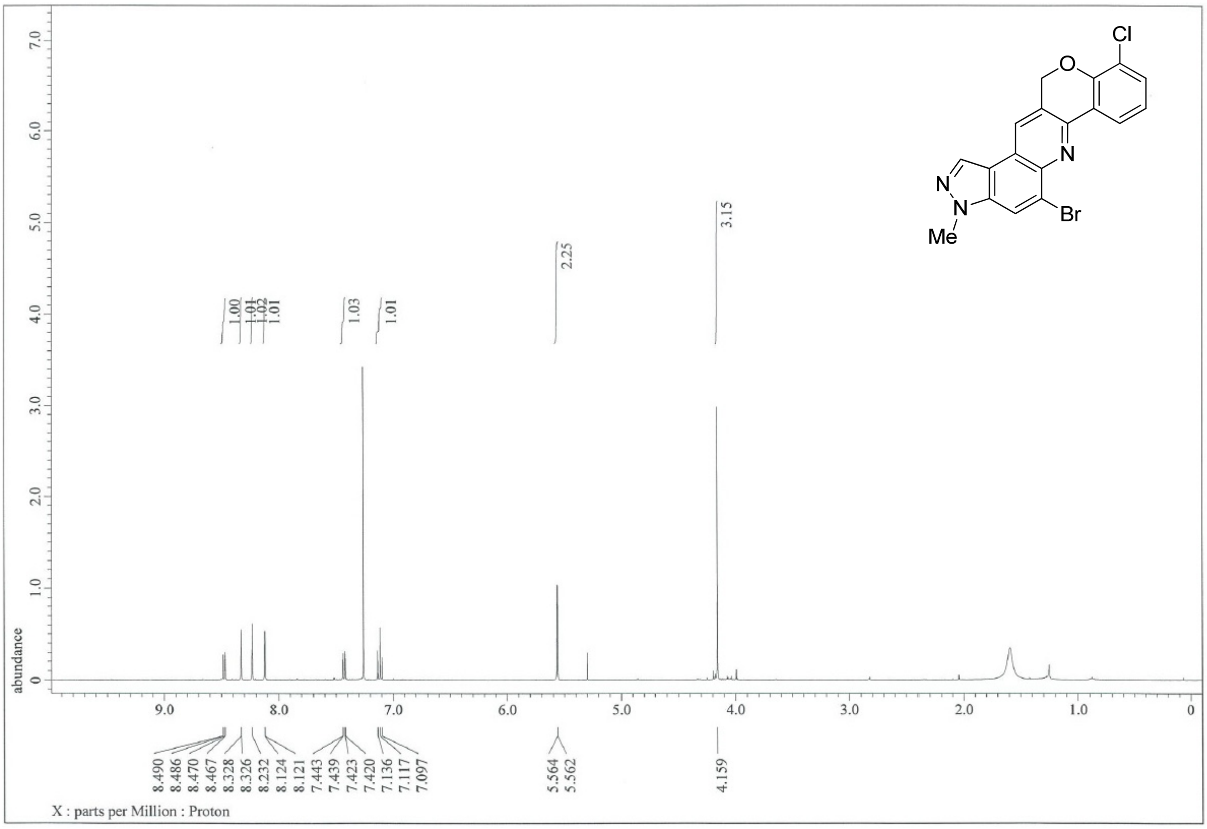

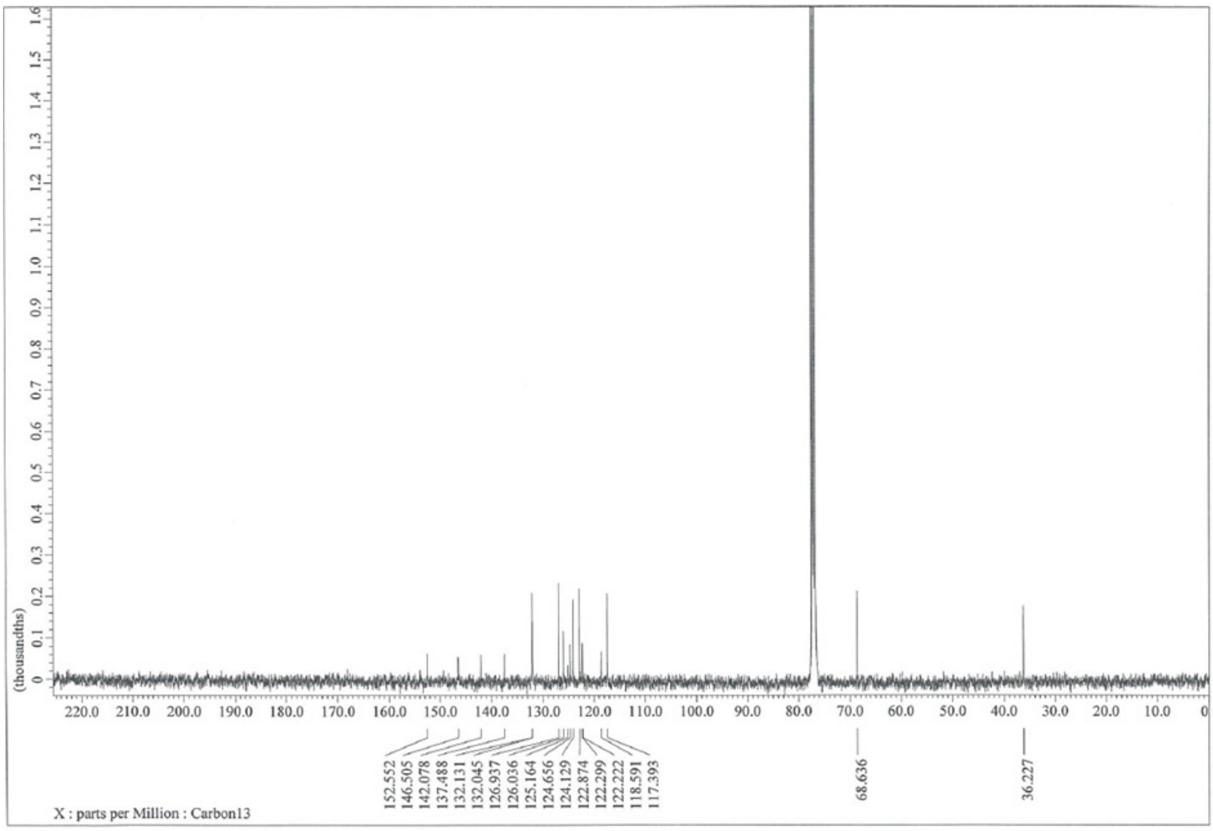
(thousandths)
152.552
146.505
142.078
137.488
132.131
132.045
126.937
126.036
125.164
124.656
124.129
122.874
122.299
122.222
118.591
117.393
68.636
36.227
X : parts per Million : Carbon13

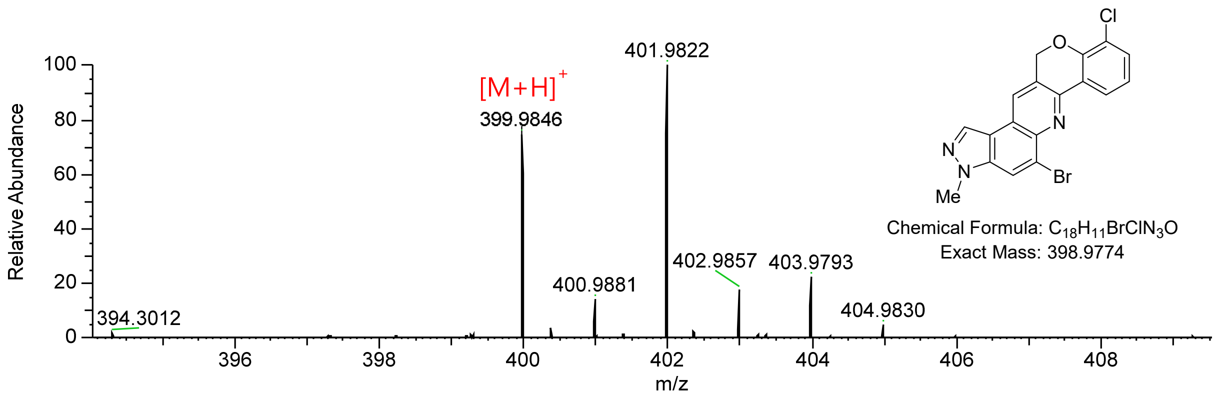

[M+H]+
399.9846
401.9822
400.9881
402.9857
403.9793
404.9830
394.3012
Relative Abundance
m/z
Cl
O
N
N
N
Me
Br
Chemical Formula: C18H11BrClN3O
Exact Mass: 398.9774

5-Bromo-8,10-dichloro-3-methyl-3,12-dihydrochromeno[4,3-b]pyrazolo[4,3-f]quinoline (2M)

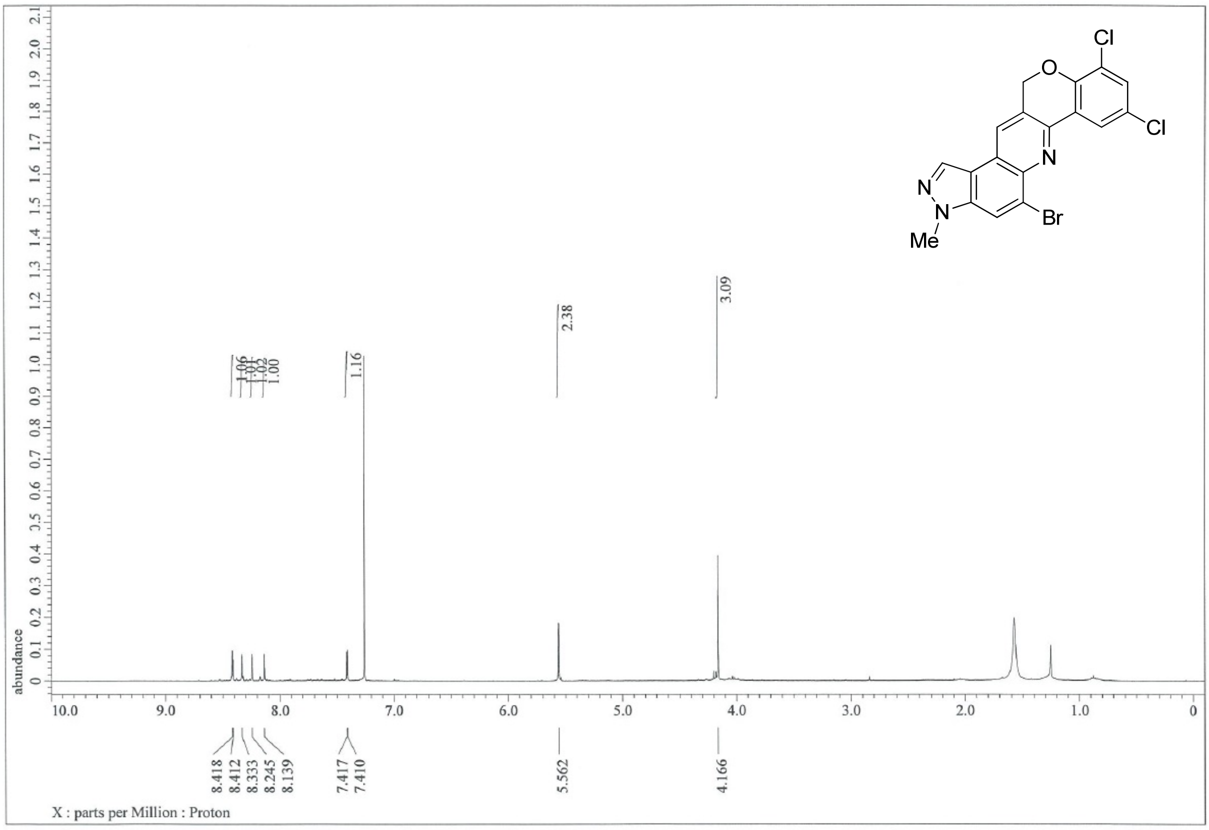

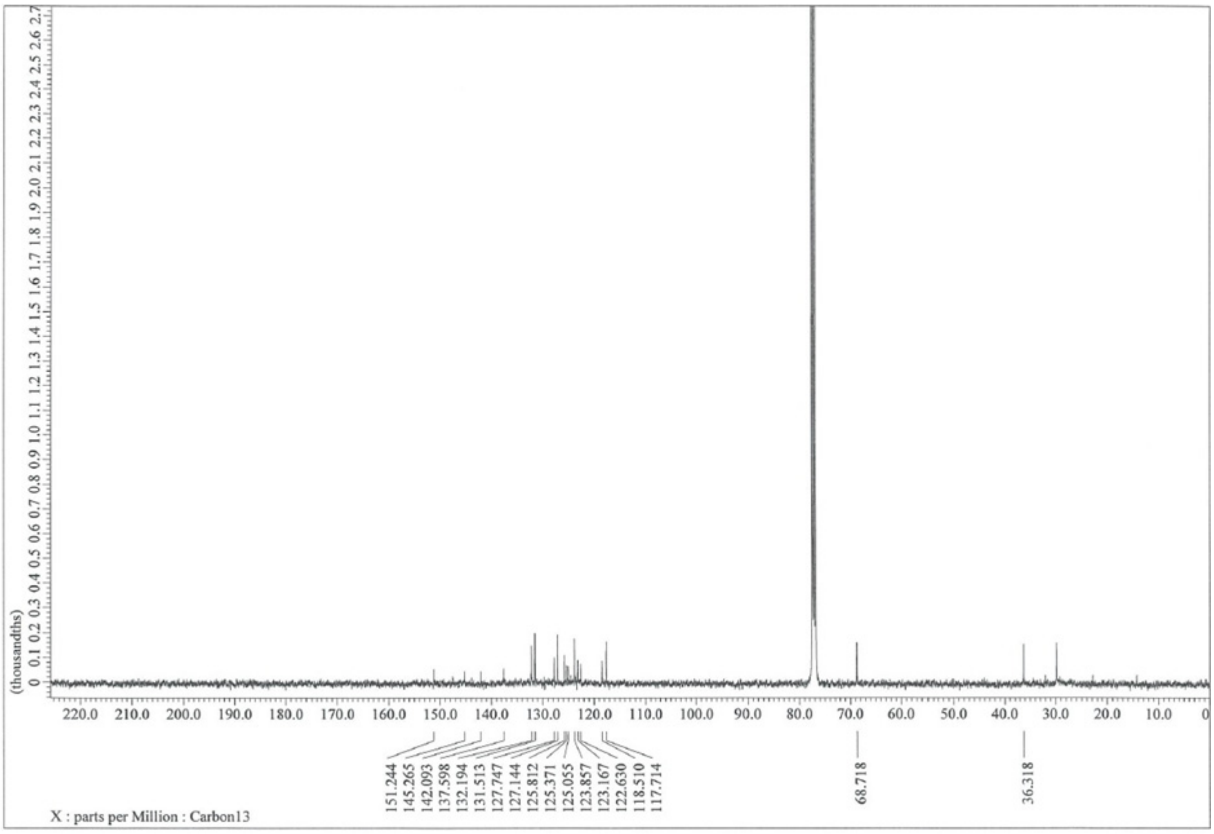
(thousandths)
0 0.1 0.2 0.3 0.4 0.5 0.6 0.7 0.8 0.9 1.0 1.1 1.2 1.3 1.4 1.5 1.6 1.7 1.8 1.9 2.0 2.1 2.2 2.3 2.4 2.5 2.6 2.7
220.0 210.0 200.0 190.0 180.0 170.0 160.0 150.0 140.0 130.0 120.0 110.0 100.0 90.0 80.0 70.0 60.0 50.0 40.0 30.0 20.0 10.0 0
151.244
145.265
142.093
137.598
132.194
131.513
127.747
127.144
125.812
125.371
125.055
123.857
123.167
122.630
118.510
117.714
68.718
36.318
X : parts per Million : Carbon13

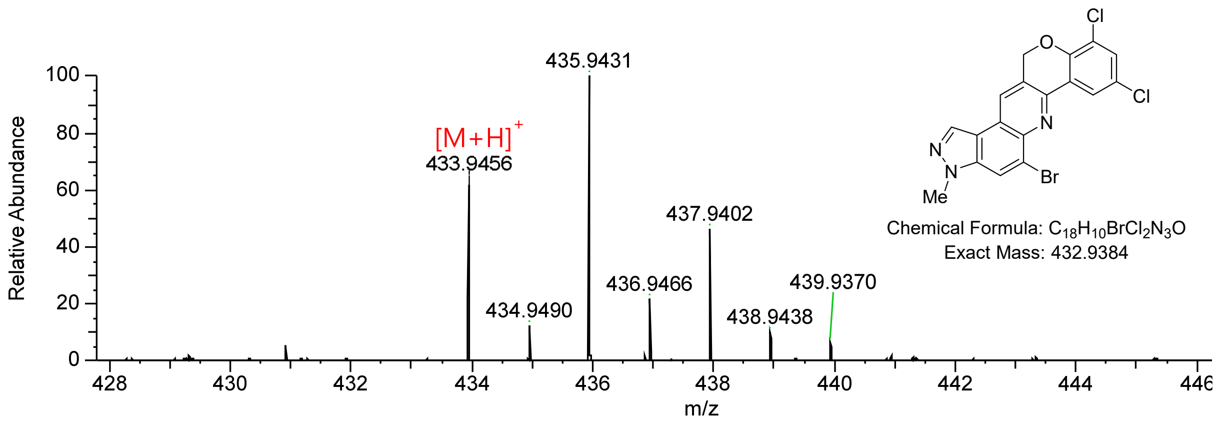

Relative Abundance
m/z
[M+H]+
433.9456
434.9490
435.9431
436.9466
437.9402
438.9438
439.9370
Chemical Formula: C18H10BrCl2N3O
Exact Mass: 432.9384
Cl
O
Cl
N
N
N
Me
Br

5-Bromo-8-bromo-3-methyl-3,12-dihydrochromeno[4,3-b]pyrazolo[4,3-f]quinoline (2N)

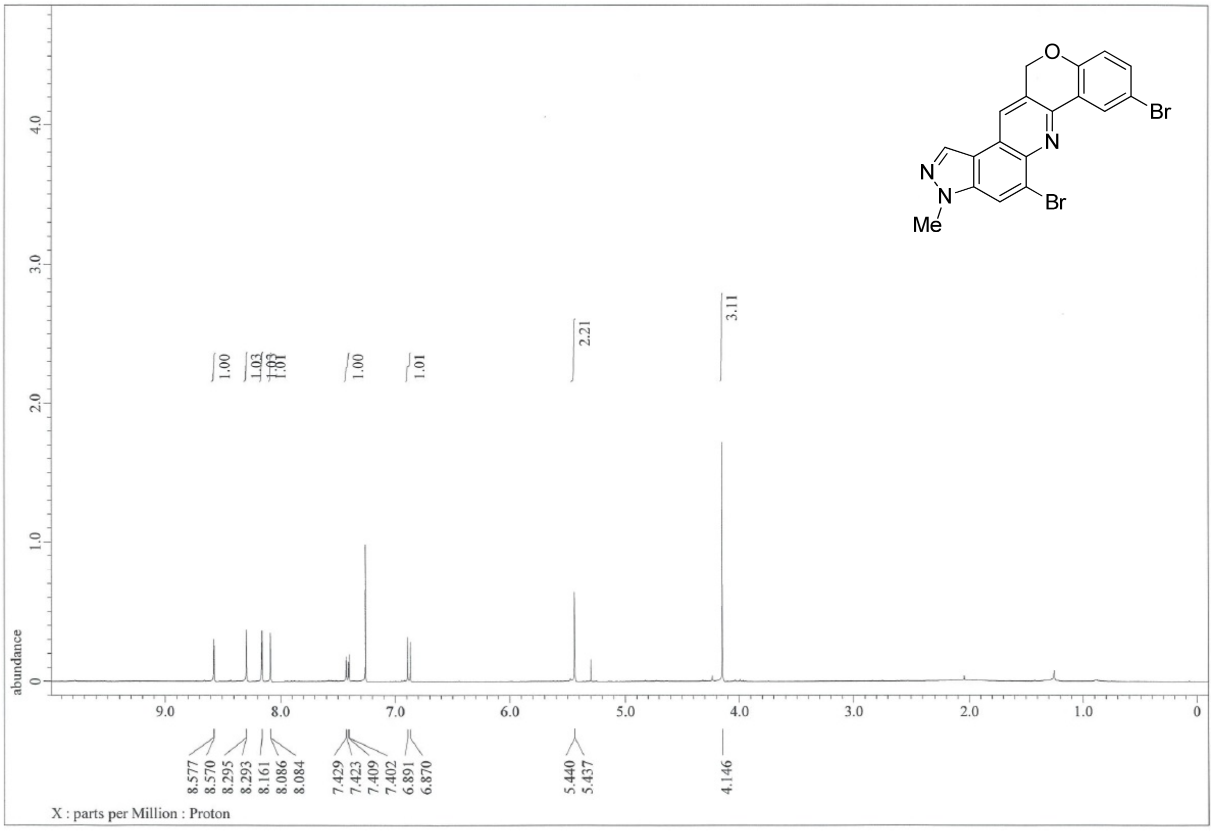

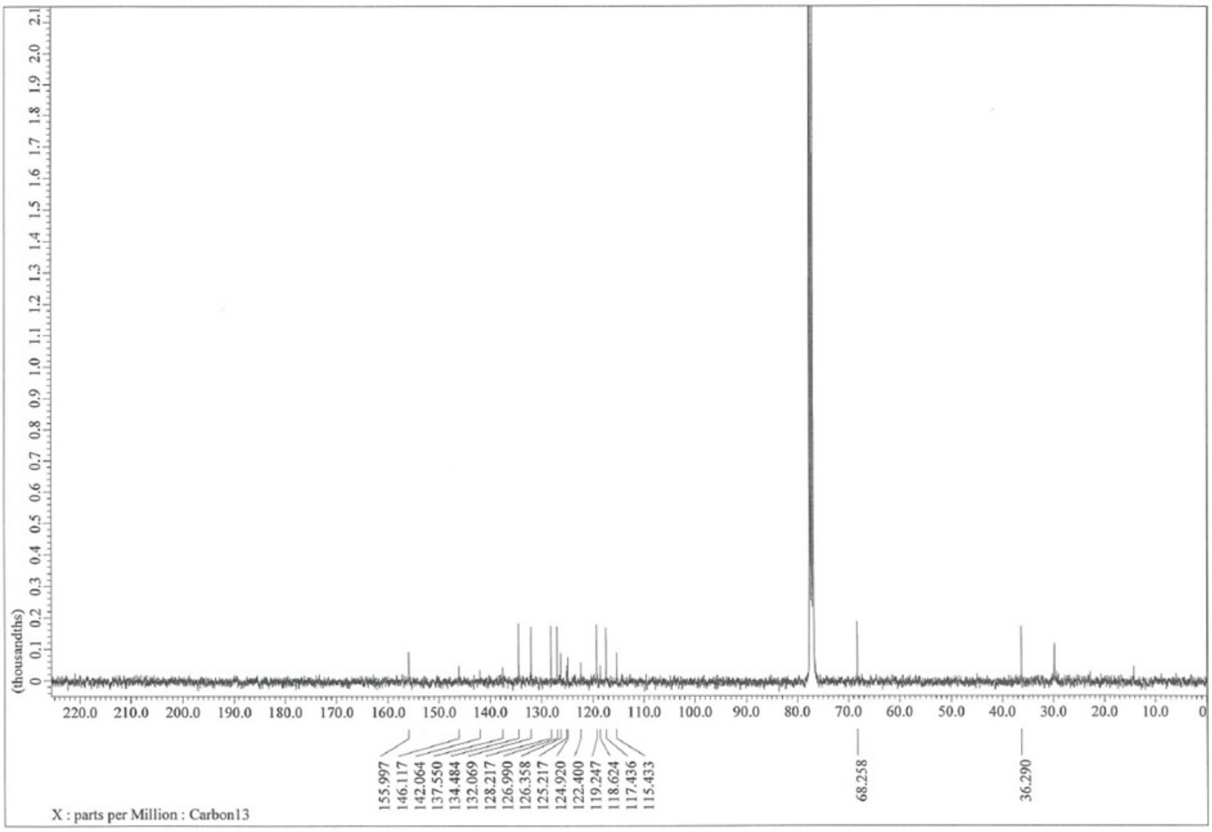
(thousandths)
X : parts per Million : Carbon13
155.997
146.117
142.064
137.550
134.484
132.069
128.217
126.990
126.358
125.217
124.920
122.400
119.247
118.624
117.436
115.433
68.258
36.290

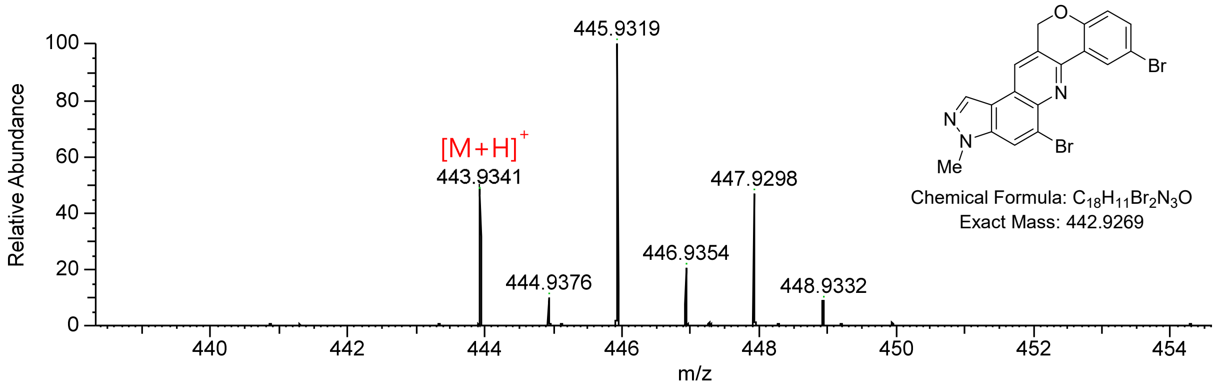

Relative Abundance
100
80
60
40
20
0
[M+H]+
443.9341
444.9376
445.9319
446.9354
447.9298
448.9332
440
442
444
446
448
450
452
454
m/z
N
N
Me
N
O
Br
Br
Chemical Formula: C18H11Br2N3O
Exact Mass: 442.9269

5-Bromo-9-bromo-3-methyl-3,12-dihydrochromeno[4,3-b]pyrazolo[4,3-f] quinoline (2O)

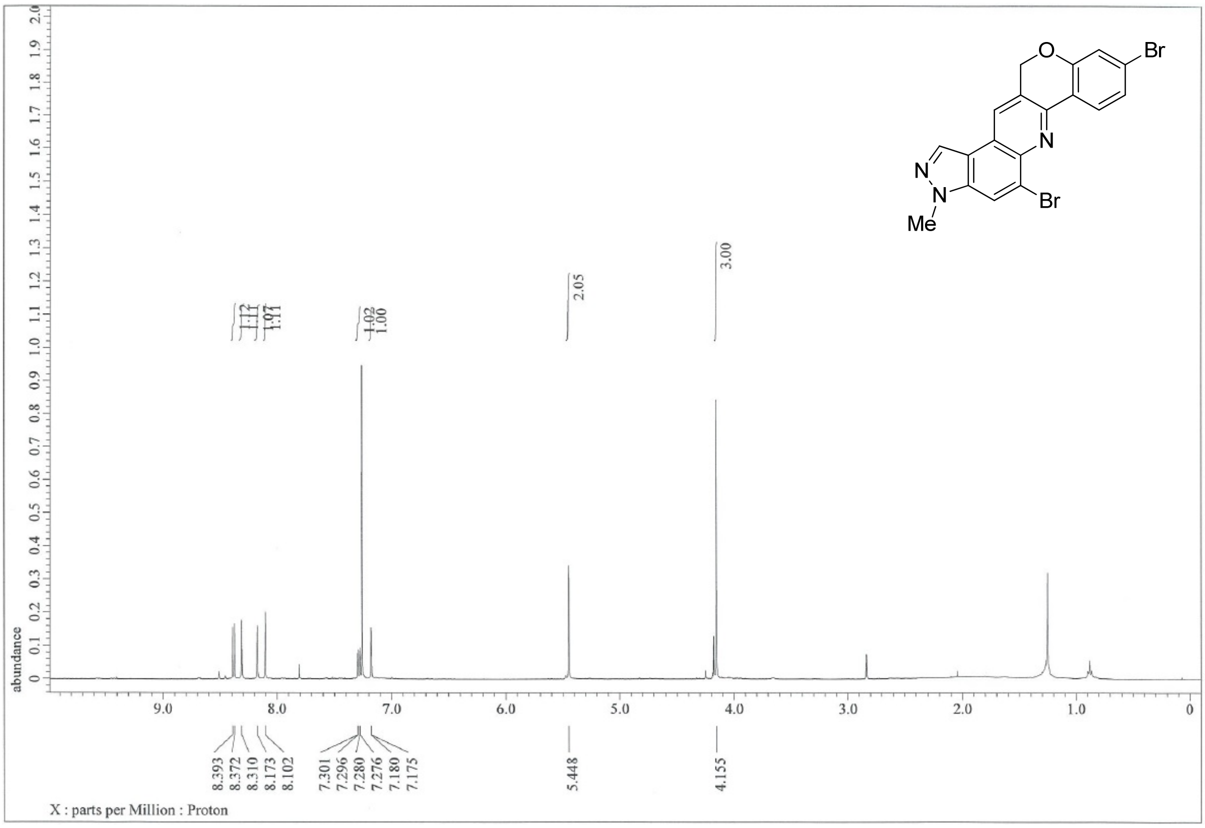

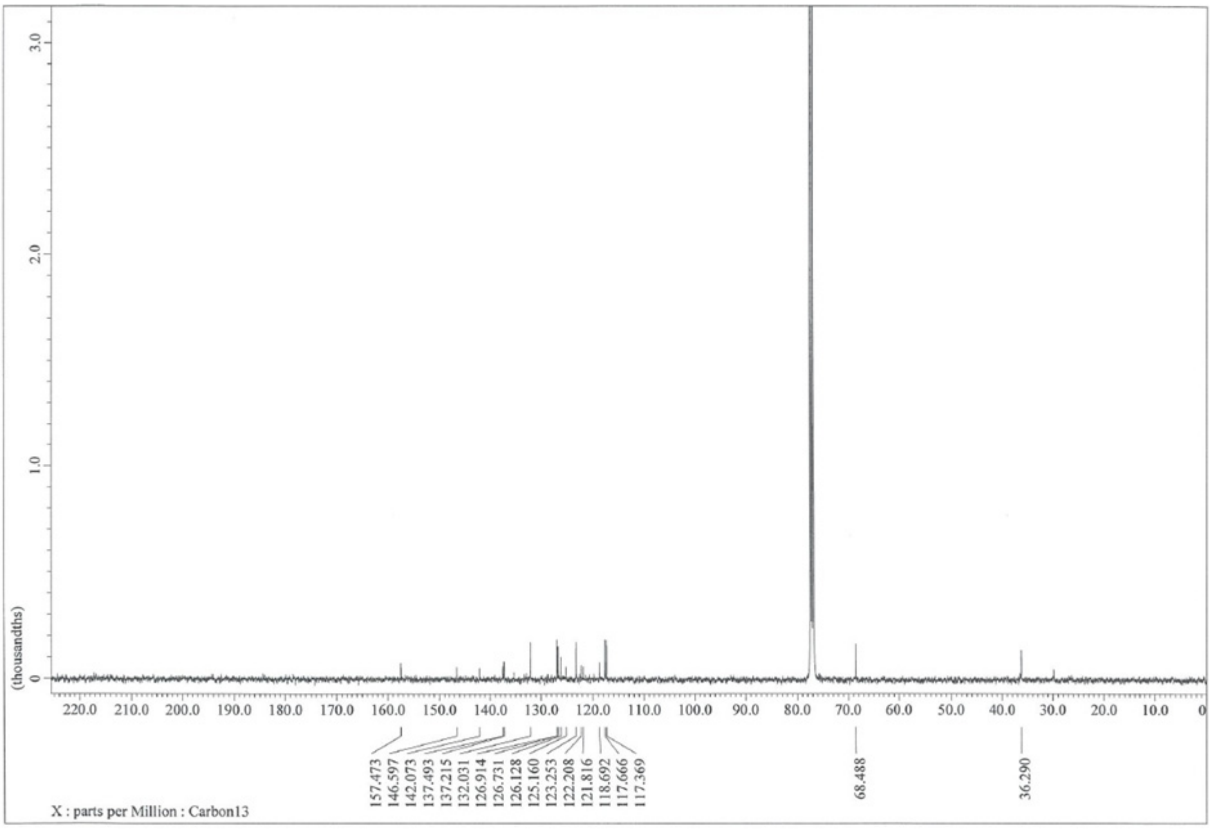
(thousandths)
3.0
2.0
1.0
0
220.0 210.0 200.0 190.0 180.0 170.0 160.0 150.0 140.0 130.0 120.0 110.0 100.0 90.0 80.0 70.0 60.0 50.0 40.0 30.0 20.0 10.0 0
157.473
146.597
142.073
137.493
137.215
132.031
126.914
126.731
126.128
125.160
123.253
122.208
121.816
118.692
117.666
117.369
68.488
36.290
X : parts per Million : Carbon13

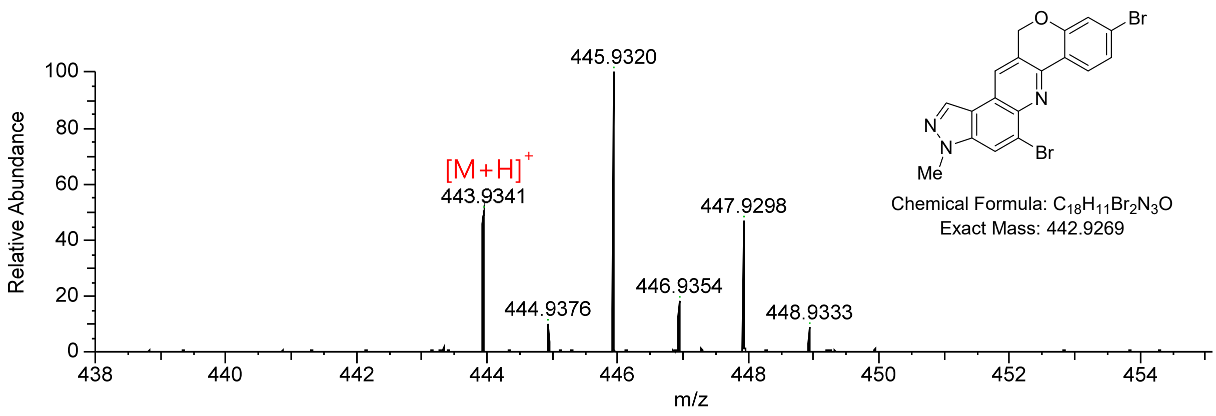

Relative Abundance
m/z
[M+H]+
443.9341
444.9376
445.9320
446.9354
447.9298
448.9333
Br
N
N
N
Me
Br
O
Chemical Formula: C18H11Br2N3O
Exact Mass: 442.9269

5-Bromo-10-bromo-3-methyl-3,12-dihydrochromeno[4,3-b]pyrazolo[4,3-f]quinoline (2P)

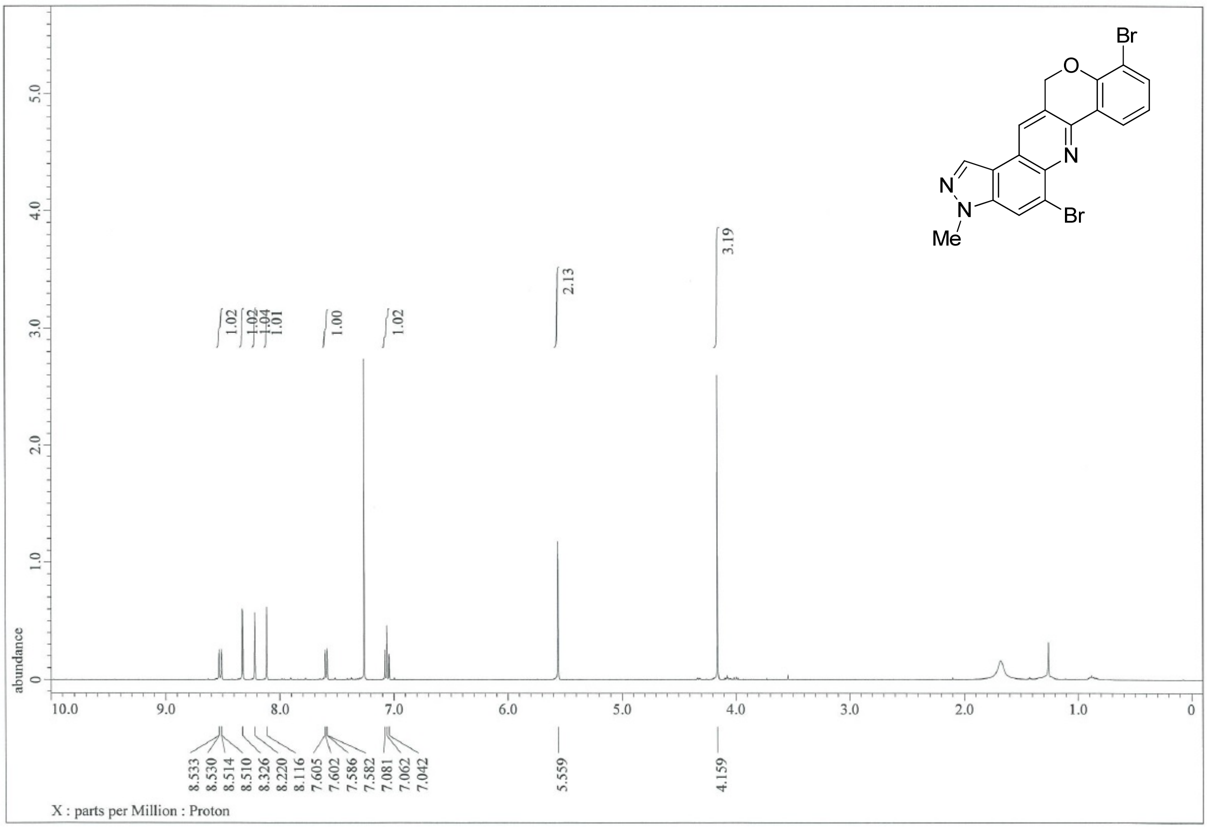

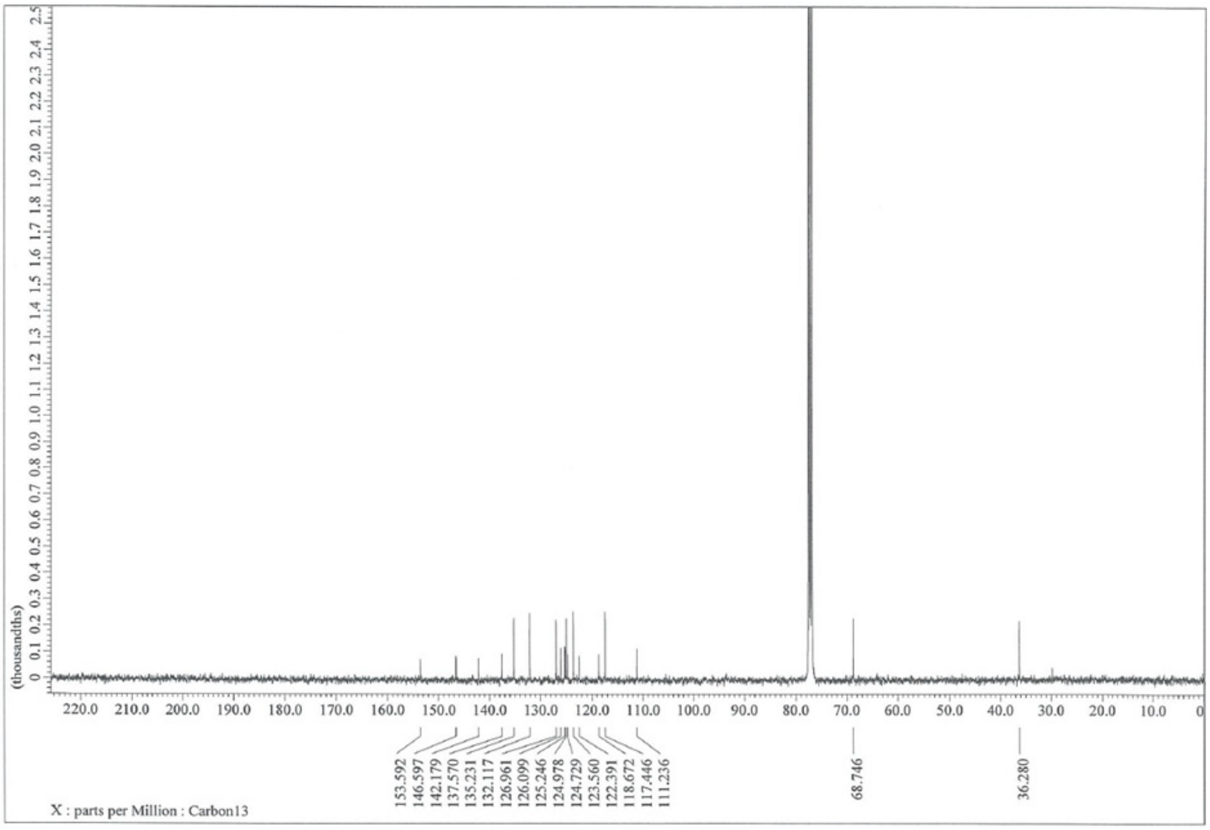
(thousandths)
X : parts per Million : Carbon13
153.592
146.597
142.179
137.570
135.231
132.117
126.961
126.099
125.246
124.978
124.729
123.560
122.391
118.672
117.446
111.236
68.746
36.280

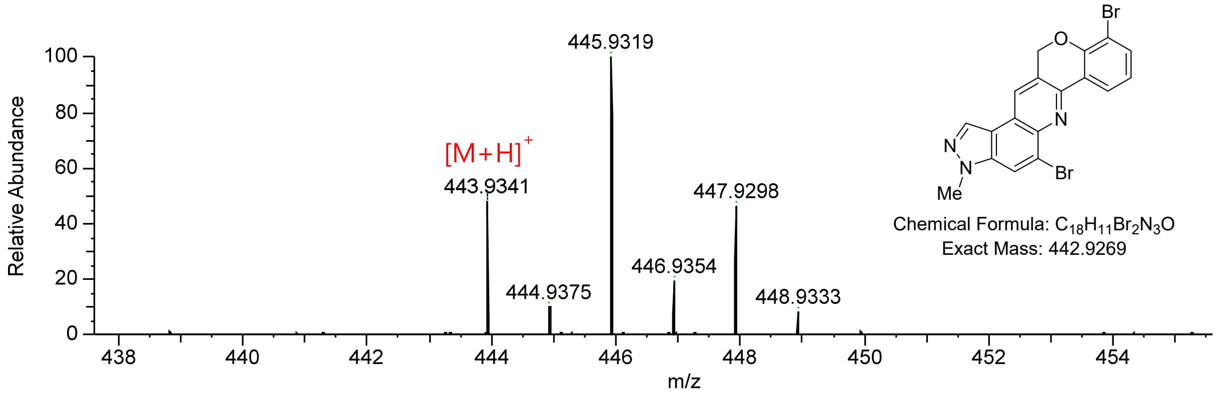

445.9319
[M+H]+
443.9341
447.9298
444.9375
446.9354
448.9333
Relative Abundance
m/z
Br
O
N
N
N
Me
Br
Chemical Formula: C18H11Br2N3O
Exact Mass: 442.9269

12-Bromo-10-methyl-6,10-dihydrobenzo[7,8]chromeno[4,3-b]pyrazolo[4,3-f]quinoline (2Q)

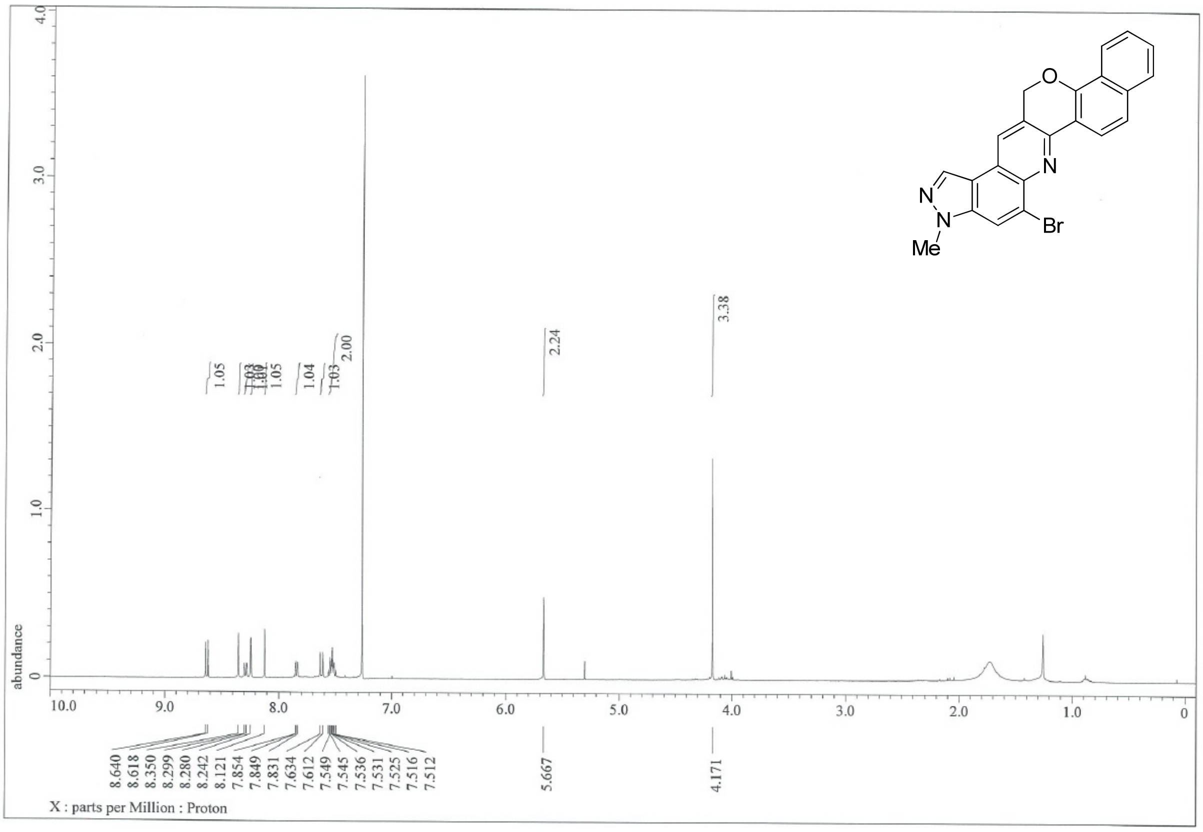

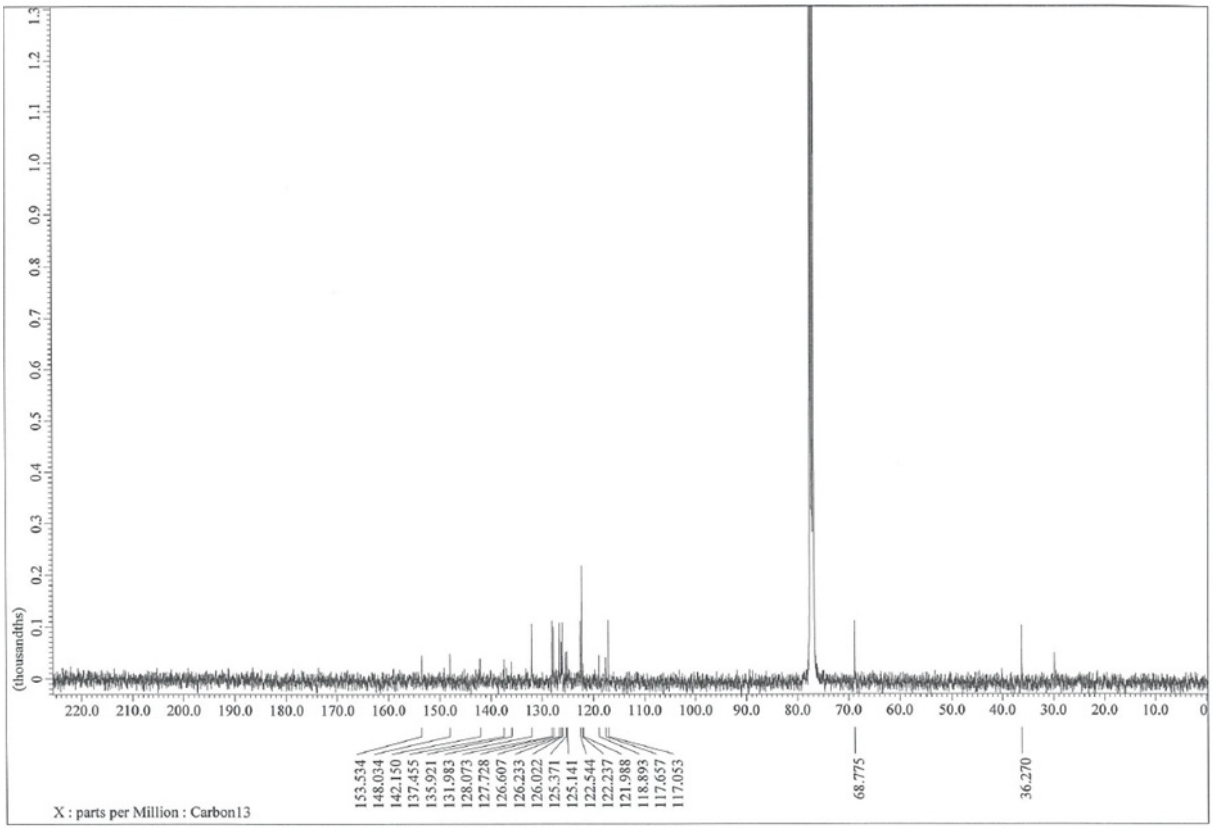

(thousandths)
X : parts per Million : Carbon13
153.534
148.034
142.150
137.455
135.921
131.983
128.073
127.728
126.607
126.233
126.022
125.371
125.141
122.544
122.237
121.988
118.893
117.657
117.053
68.775
36.270

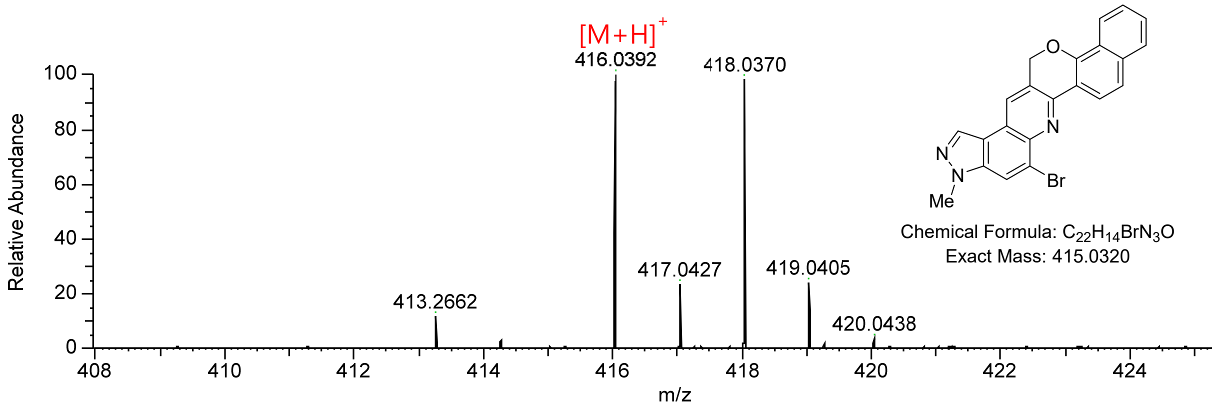
[M+H]+
416.0392
418.0370
417.0427
419.0405
420.0438
413.2662
Relative Abundance
m/z
N
N
Me
N
O
Br
Chemical Formula: C22H14BrN3O
Exact Mass: 415.0320

14-Bromo-12-methyl-8,12-dihydrobenzo[5,6]chromeno[4,3-b]pyrazolo[4,3-f]quinoline (2R)

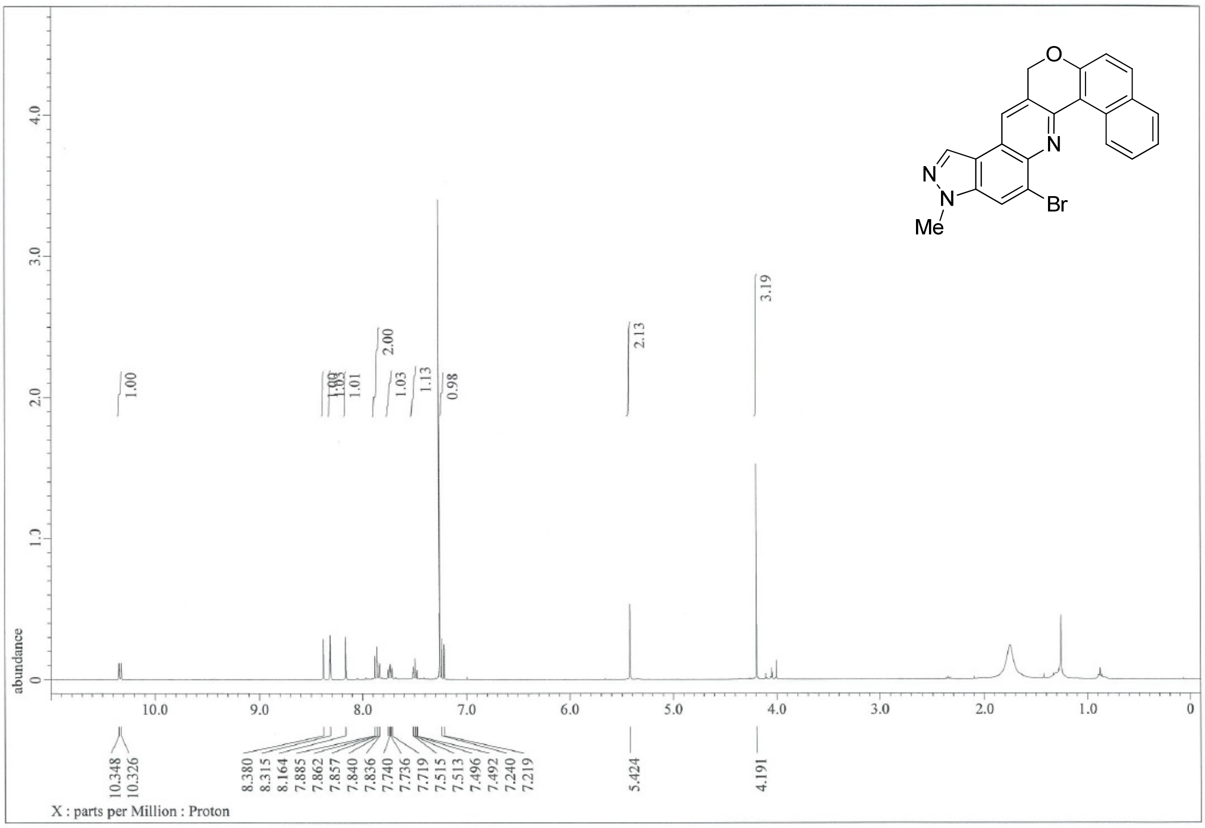

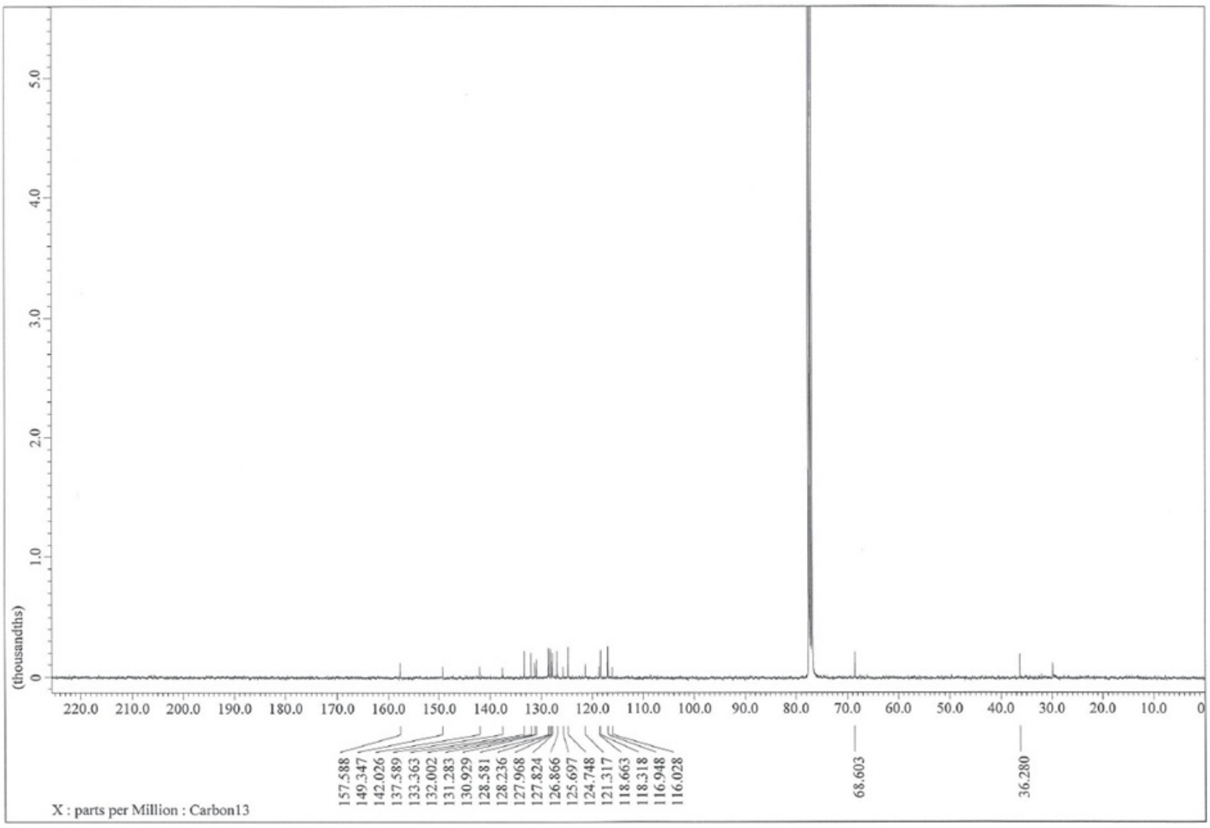

(thousandths)
0
1.0
2.0
3.0
4.0
5.0
220.0 210.0 200.0 190.0 180.0 170.0 160.0 150.0 140.0 130.0 120.0 110.0 100.0 90.0 80.0 70.0 60.0 50.0 40.0 30.0 20.0 10.0 0
157.588
149.347
142.026
137.589
133.363
132.002
131.283
130.929
128.581
128.236
127.968
127.824
126.866
125.697
124.748
121.317
118.663
118.318
116.948
116.028
68.603
36.280
X : parts per Million : Carbon13

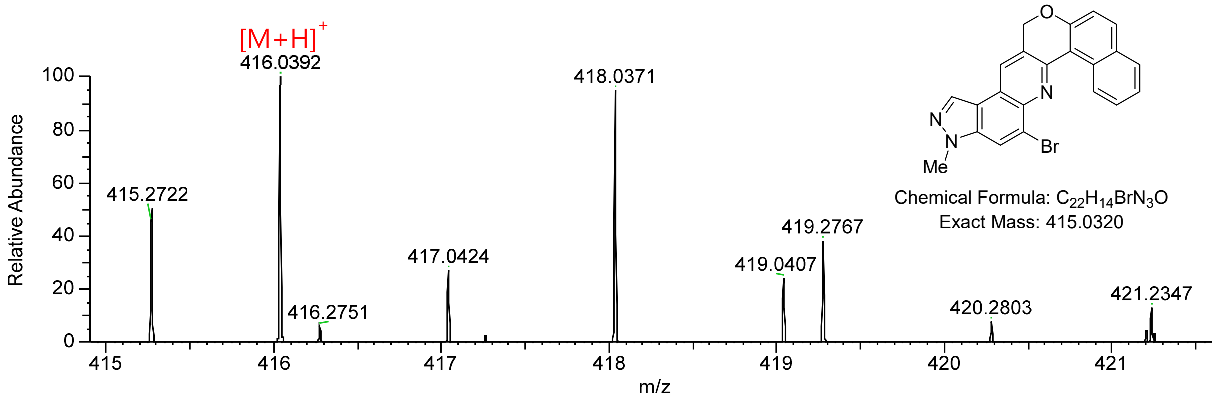
[M+H]+
416.0392
415.2722
416.2751
417.0424
418.0371
419.0407
419.2767
420.2803
421.2347
Relative Abundance
m/z
Chemical Formula: C22H14BrN3O
Exact Mass: 415.0320
Br
Me
N
O

8-Allyl-5-bromo-10-methoxy-3-methyl-3,12-dihydrochromeno[4,3-b]pyrazolo[4,3-f]quinoline (2S)

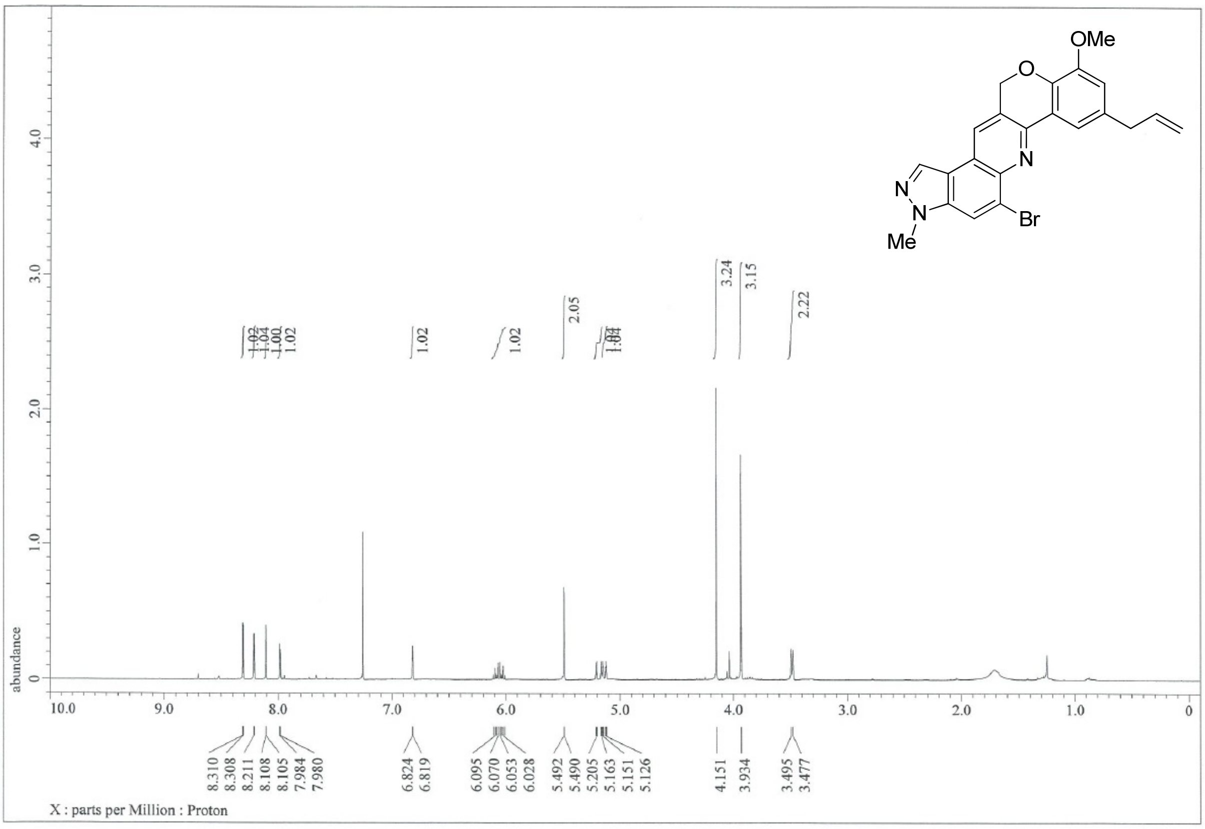

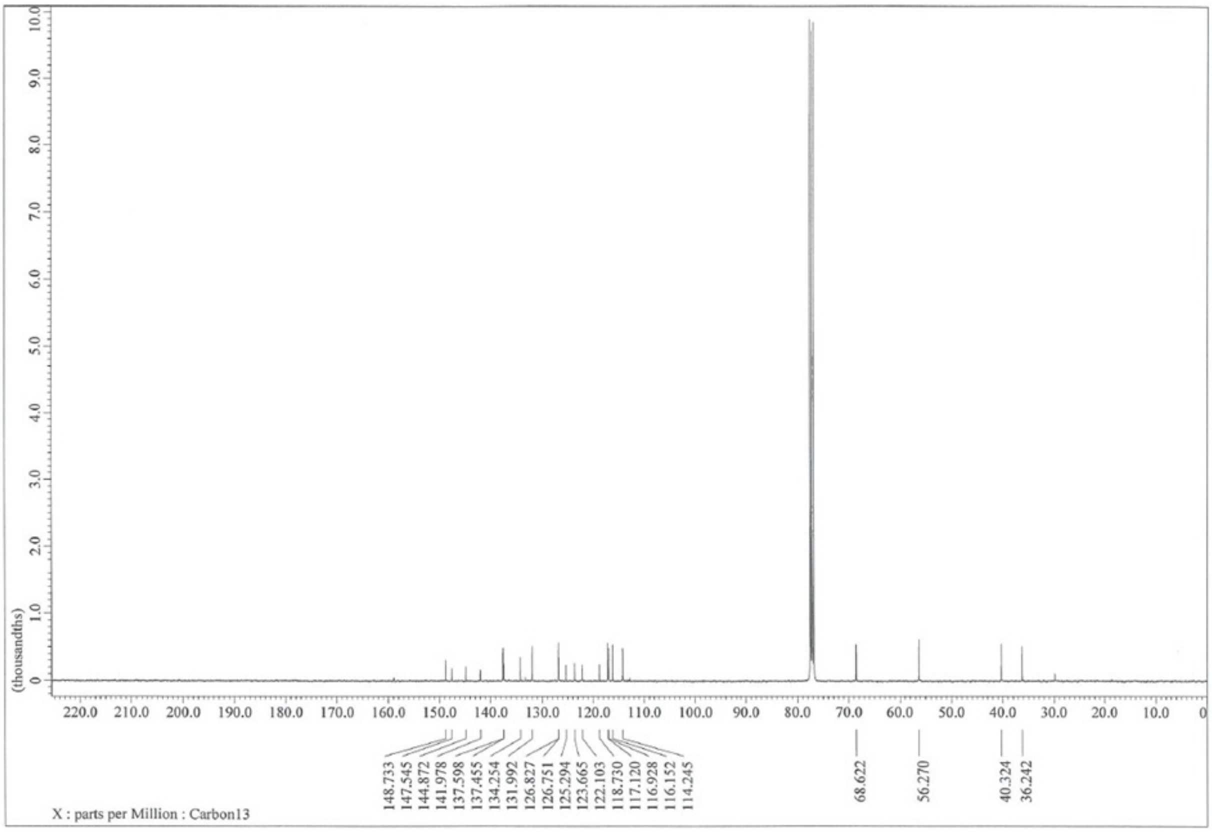
(thousandths)
0
1.0
2.0
3.0
4.0
5.0
6.0
7.0
8.0
9.0
10.0
220.0 210.0 200.0 190.0 180.0 170.0 160.0 150.0 140.0 130.0 120.0 110.0 100.0 90.0 80.0 70.0 60.0 50.0 40.0 30.0 20.0 10.0 0
148.733
147.545
144.872
141.978
137.598
137.455
134.254
131.992
126.827
126.751
125.294
123.665
122.103
118.730
117.120
116.928
116.152
114.245
68.622
56.270
40.324
36.242
X : parts per Million : Carbon13

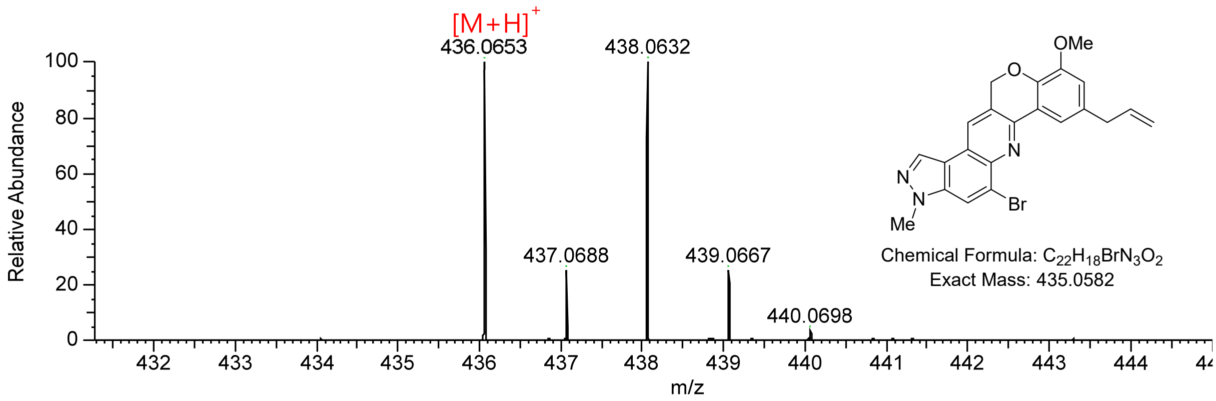

[M+H]+
436.0653
438.0632
437.0688
439.0667
440.0698
100
80
60
40
20
0
Relative Abundance
432
433
434
435
436
437
438
439
440
441
442
443
444
m/z
OMe
O
N
N
N
Me
Br
Chemical Formula: C22H18BrN3O2
Exact Mass: 435.0582

14-Bromo-9,12-dimethyl-8,12-dihydrobenzo[5,6]chromeno[4,3-b]pyrazolo[4,3-f]quinoline (2T)

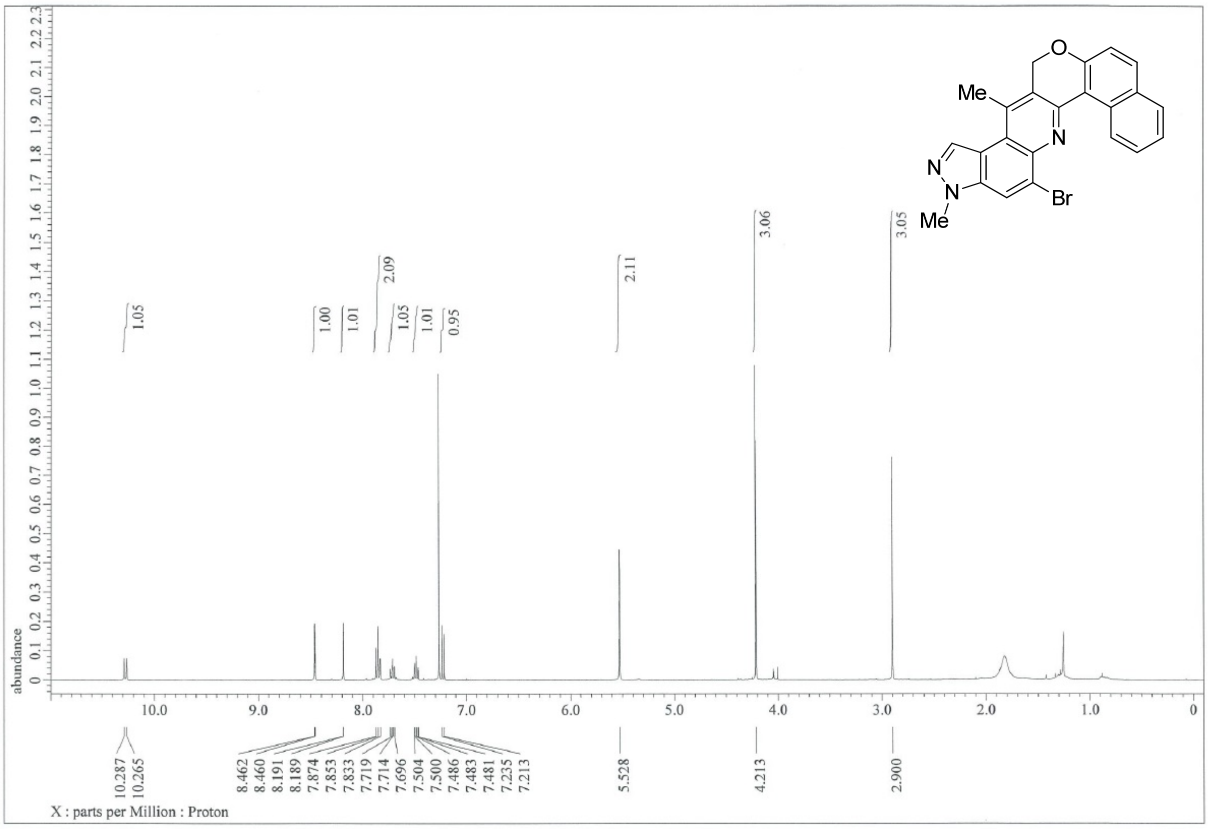

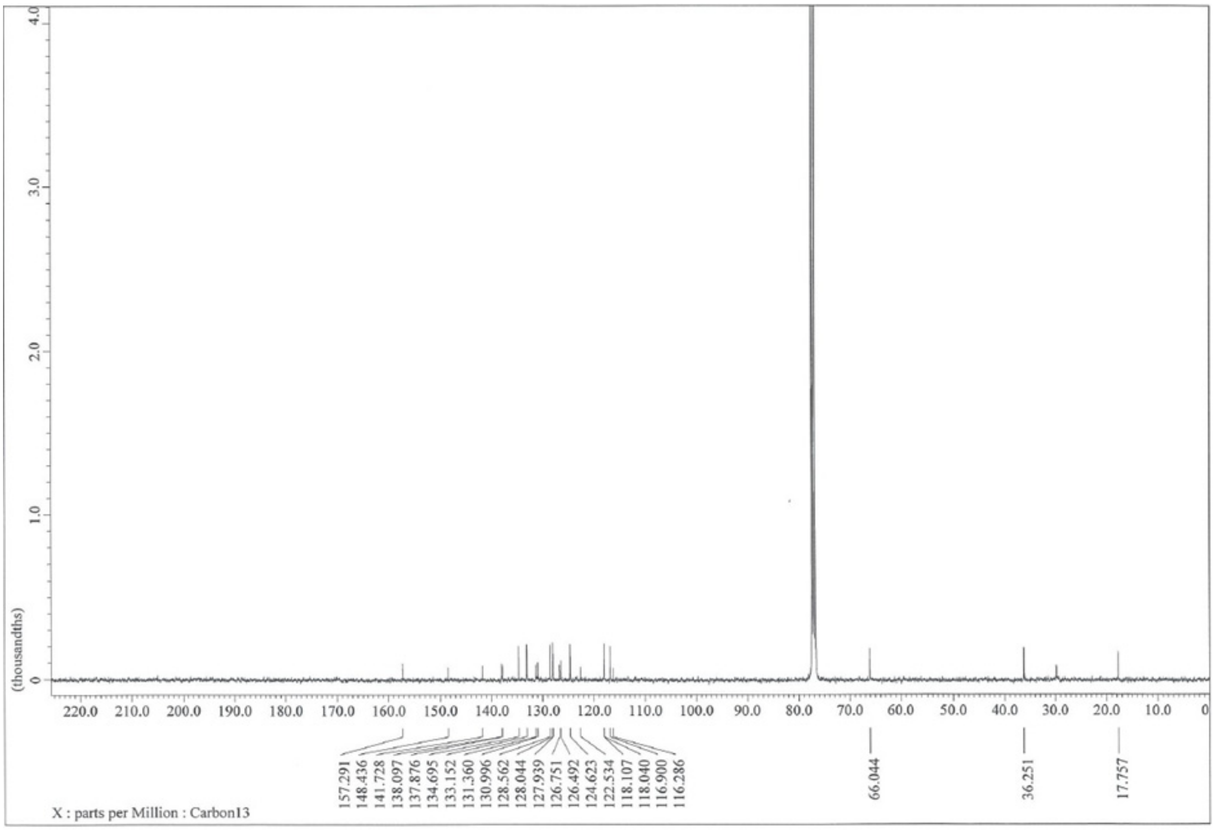

(thousandths)
X : parts per Million : Carbon13
157.291
148.436
141.728
138.097
137.876
134.695
133.152
131.360
130.996
128.562
128.044
127.939
126.751
126.492
124.623
122.534
118.107
118.040
116.900
116.286
66.044
36.251
17.757

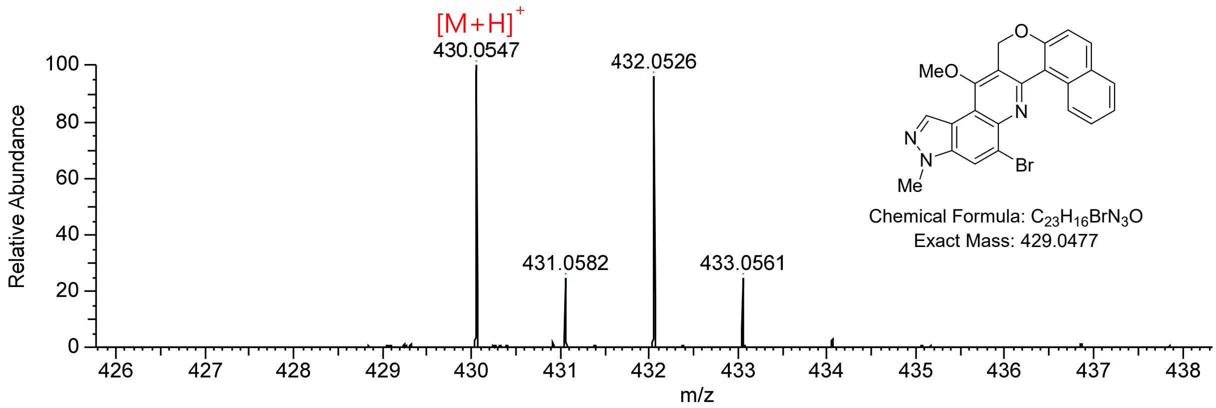

[M+H]+
430.0547
432.0526
431.0582
433.0561
Relative Abundance
m/z
MeO
N
N
N
Me
O
Br
Chemical Formula: C23H16BrN3O
Exact Mass: 429.0477

2-(Prop-2-yn-1-yloxy)benzaldehyde (6A)

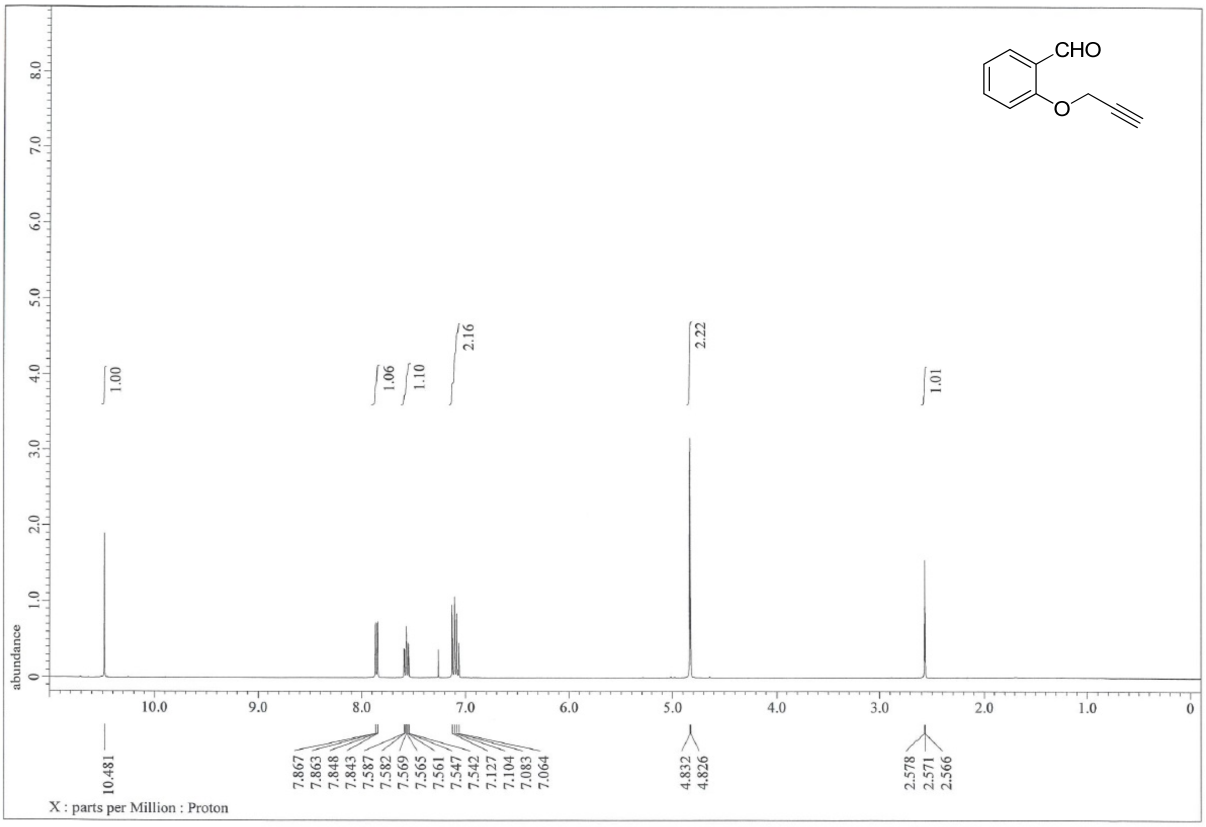

4-Methoxy-2-(prop-2-yn-1-yloxy)benzaldehyde (6B)

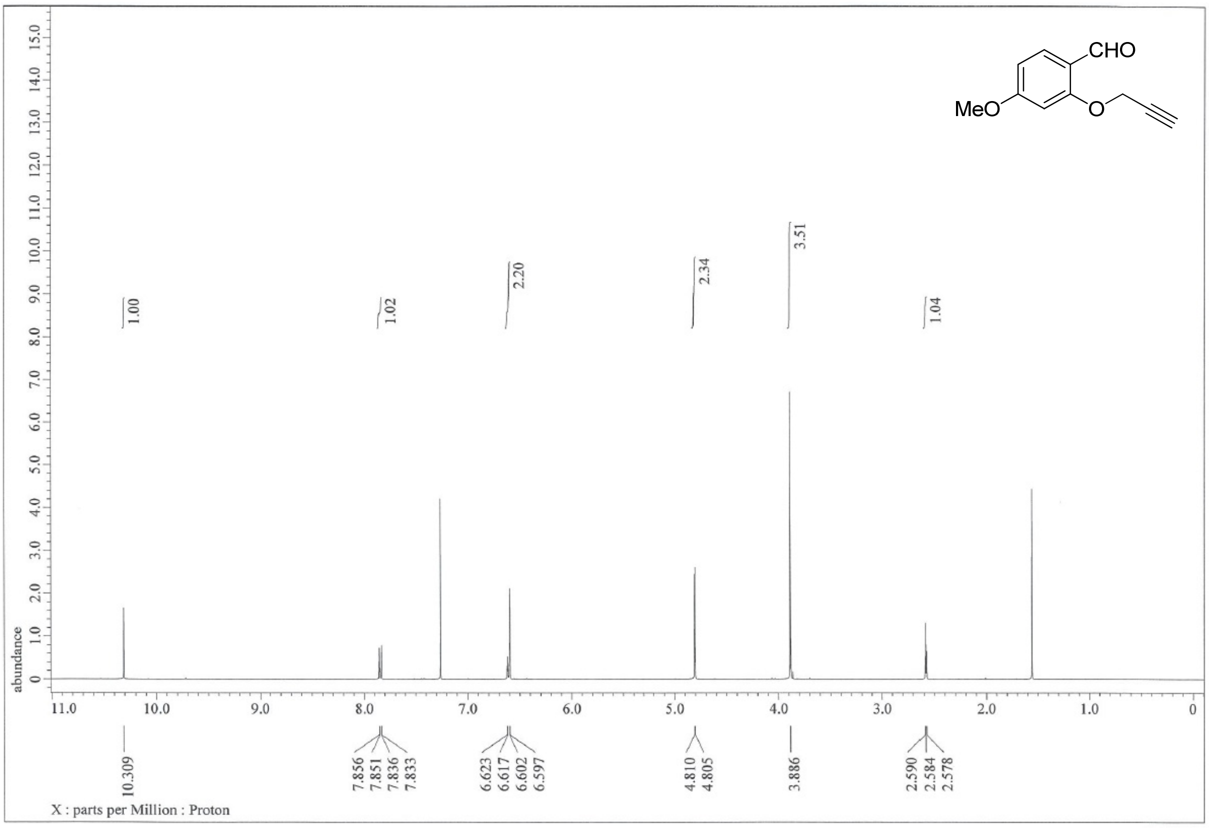

5-Methoxy-2-(prop-2-yn-1-yloxy)benzaldehyde (6C)

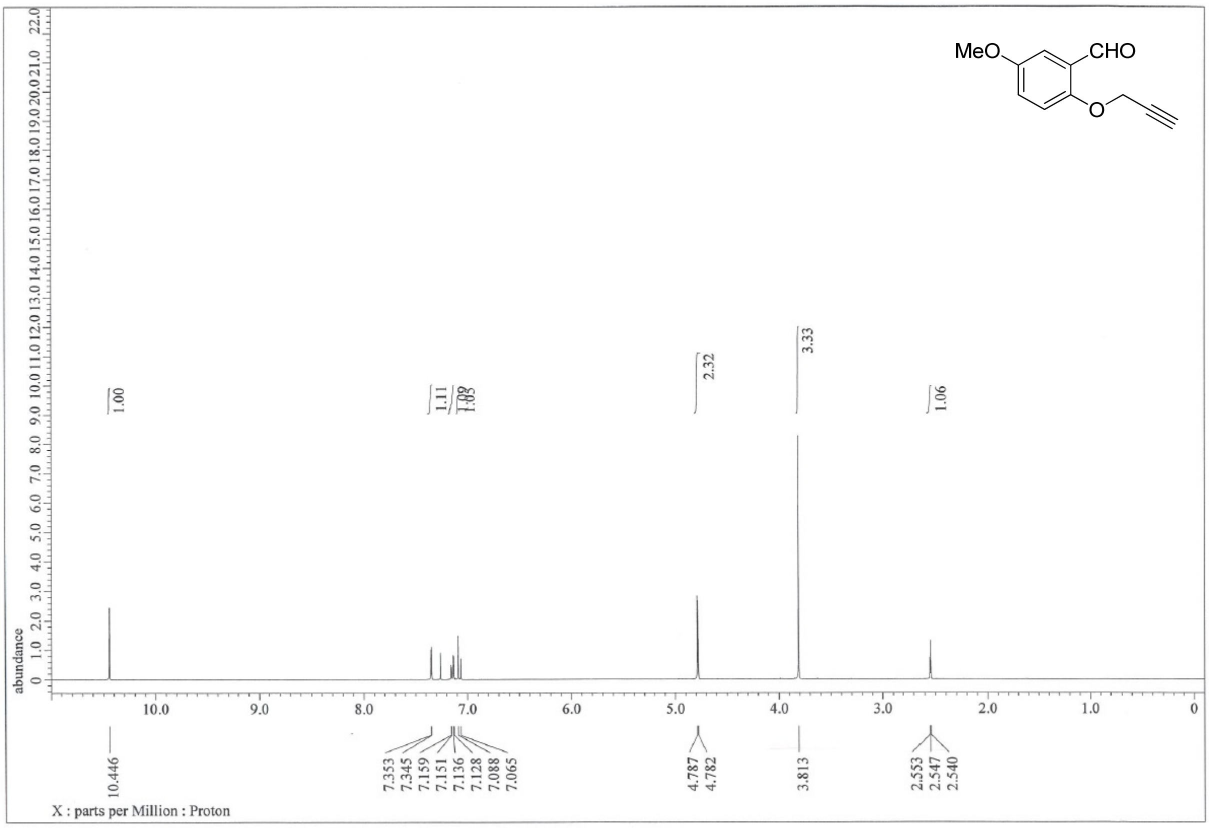

3-Methyl-2-(prop-2-yn-1-yloxy)benzaldehyde (6D)

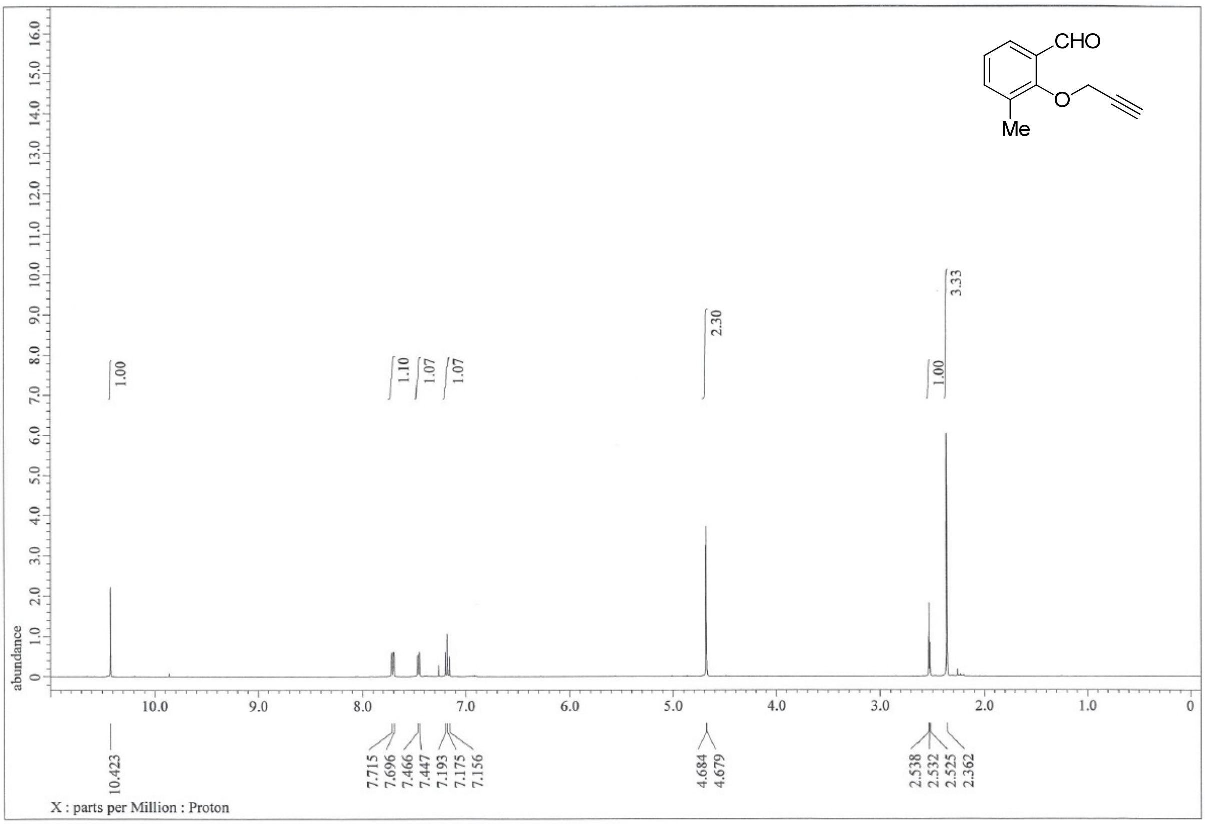

6-Fluoro-2-(prop-2-yn-1-yloxy)benzaldehyde (6E)

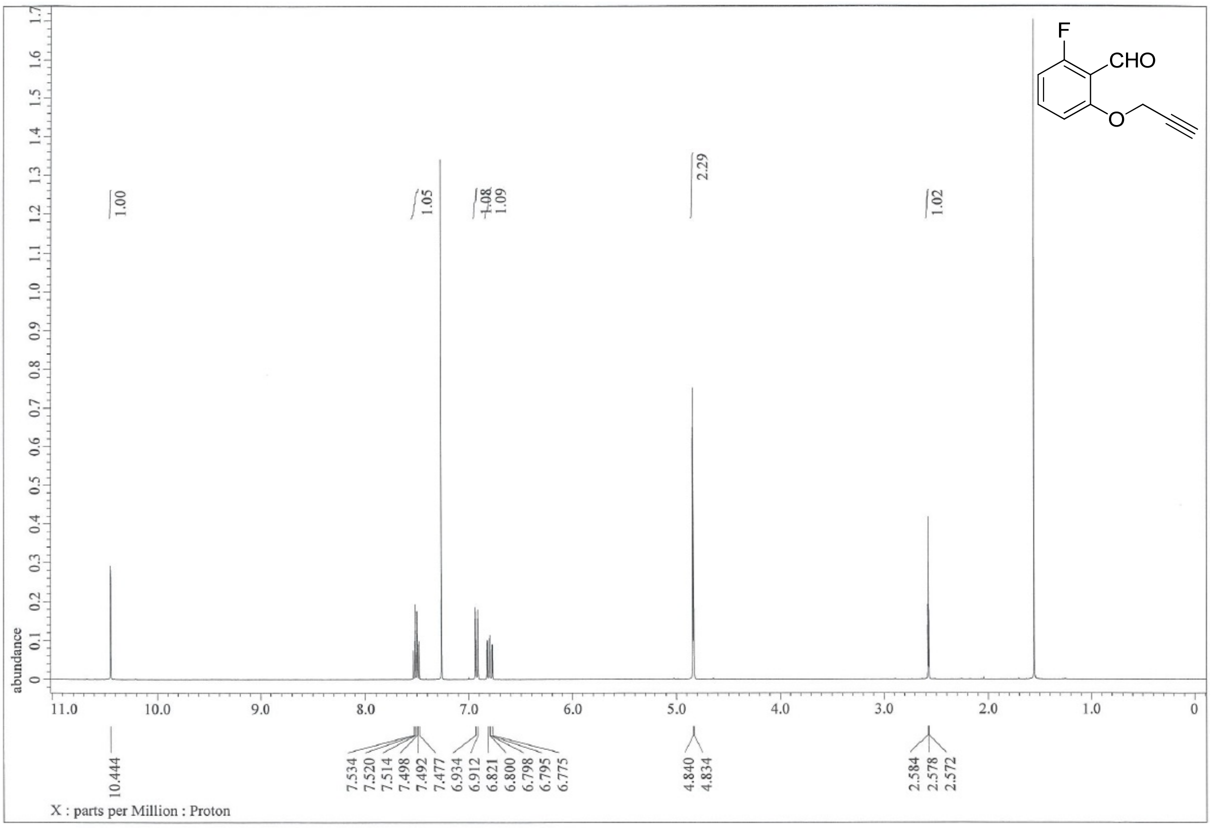

5-Fluoro-2-(prop-2-yn-1-yloxy)benzaldehyde (6F)

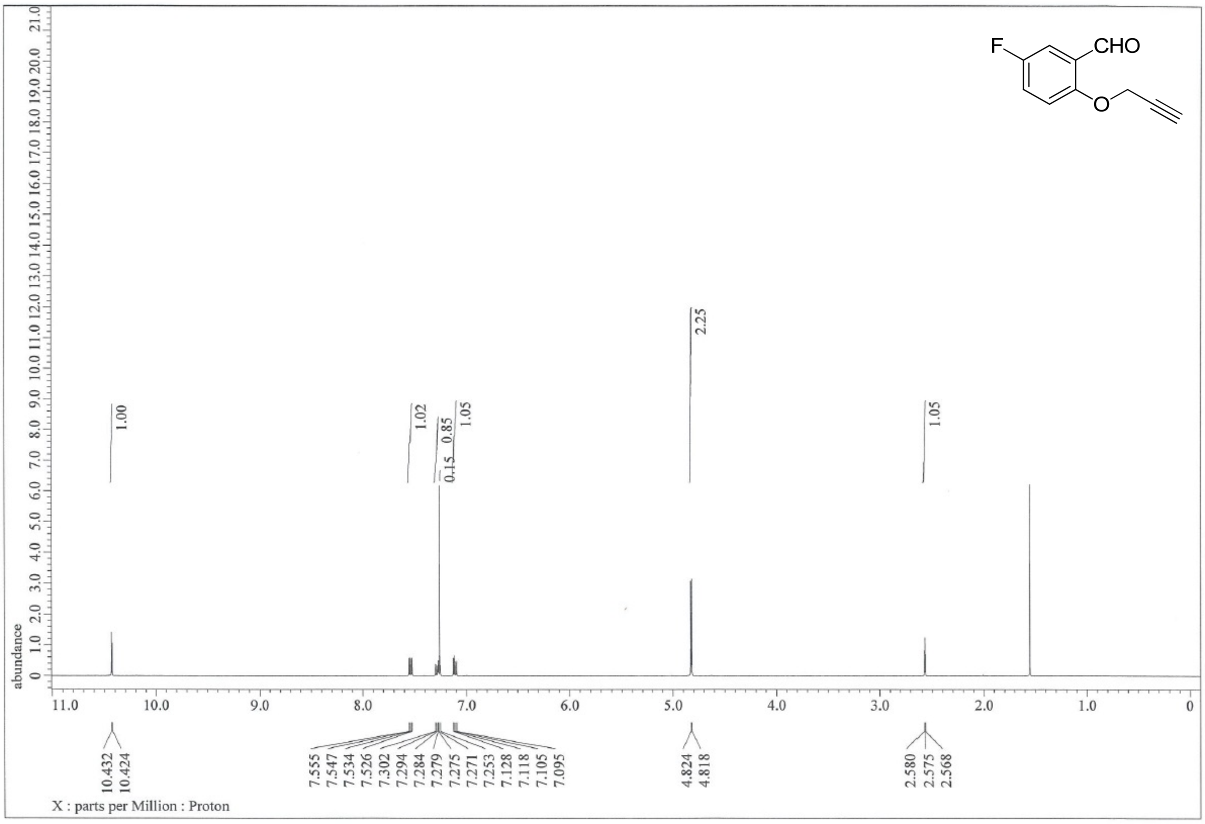

4-Fluoro-2-(prop-2-yn-1-yloxy)benzaldehyde (6G)

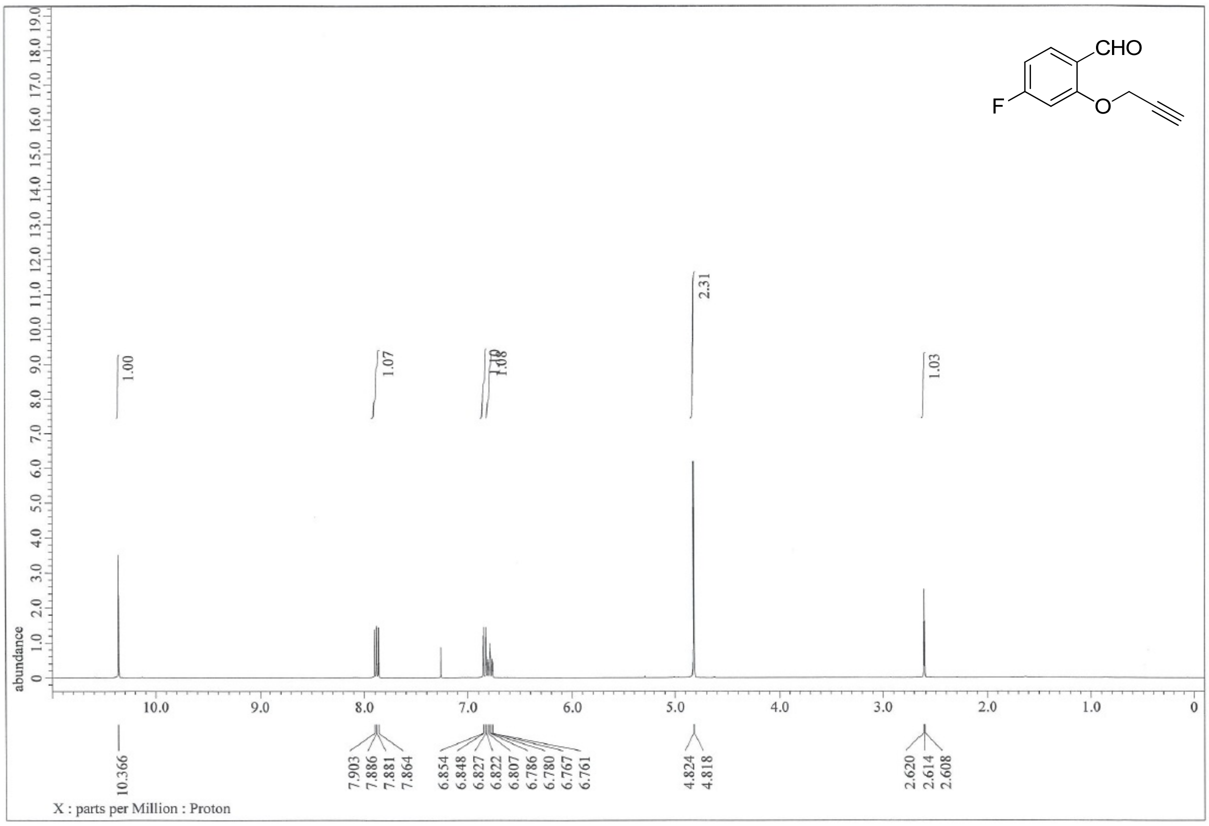

3-Fluoro-2-(prop-2-yn-1-yloxy)benzaldehyde (6H)

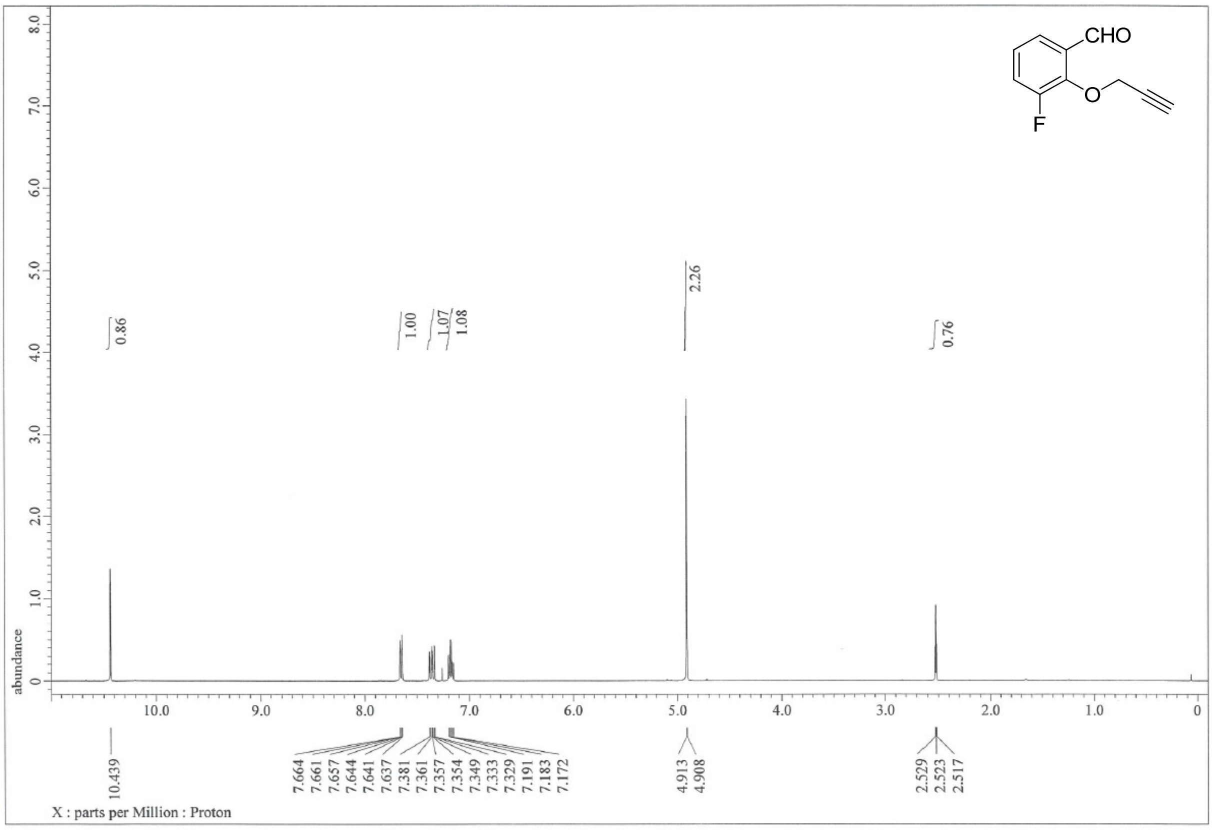

6-Chloro-2-(prop-2-yn-1-yloxy)benzaldehyde (6I)

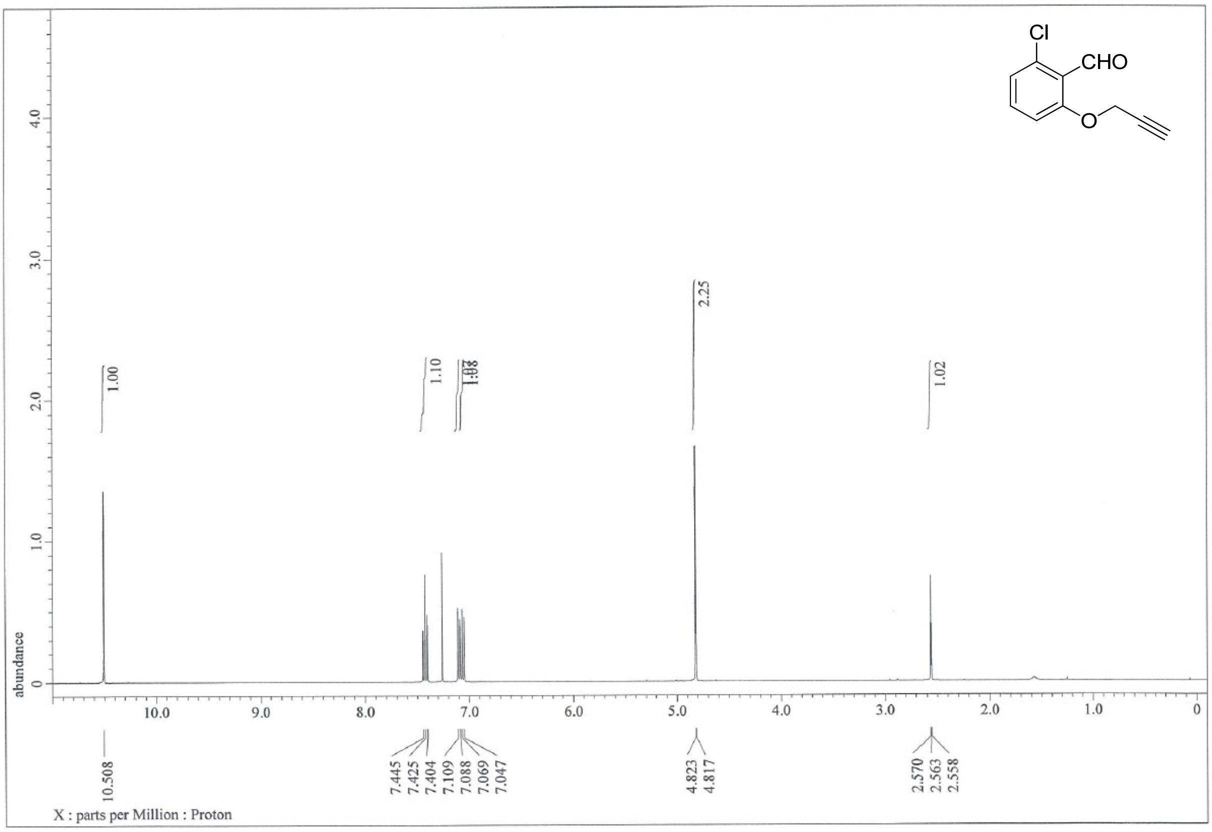

5-Chloro-2-(prop-2-yn-1-yloxy)benzaldehyde (6J)

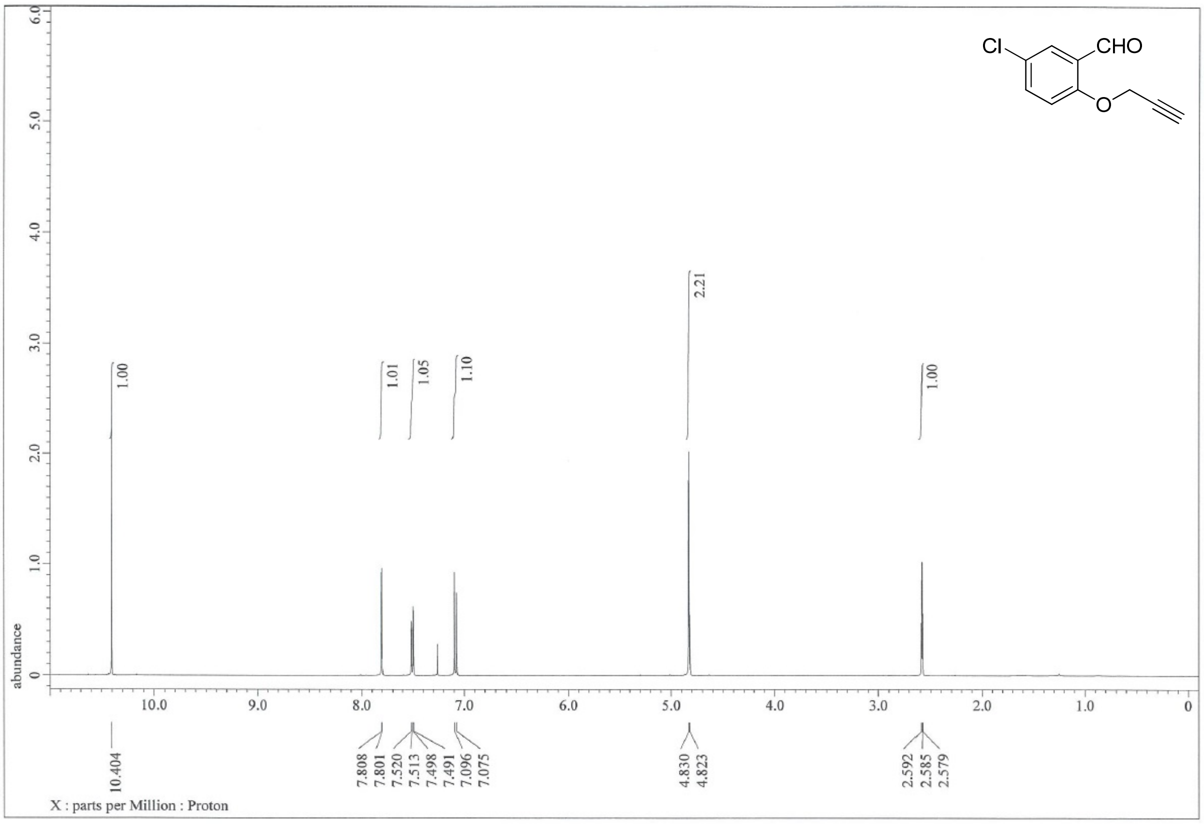

4-Chloro-2-(prop-2-yn-1-yloxy)benzaldehyde (6K)

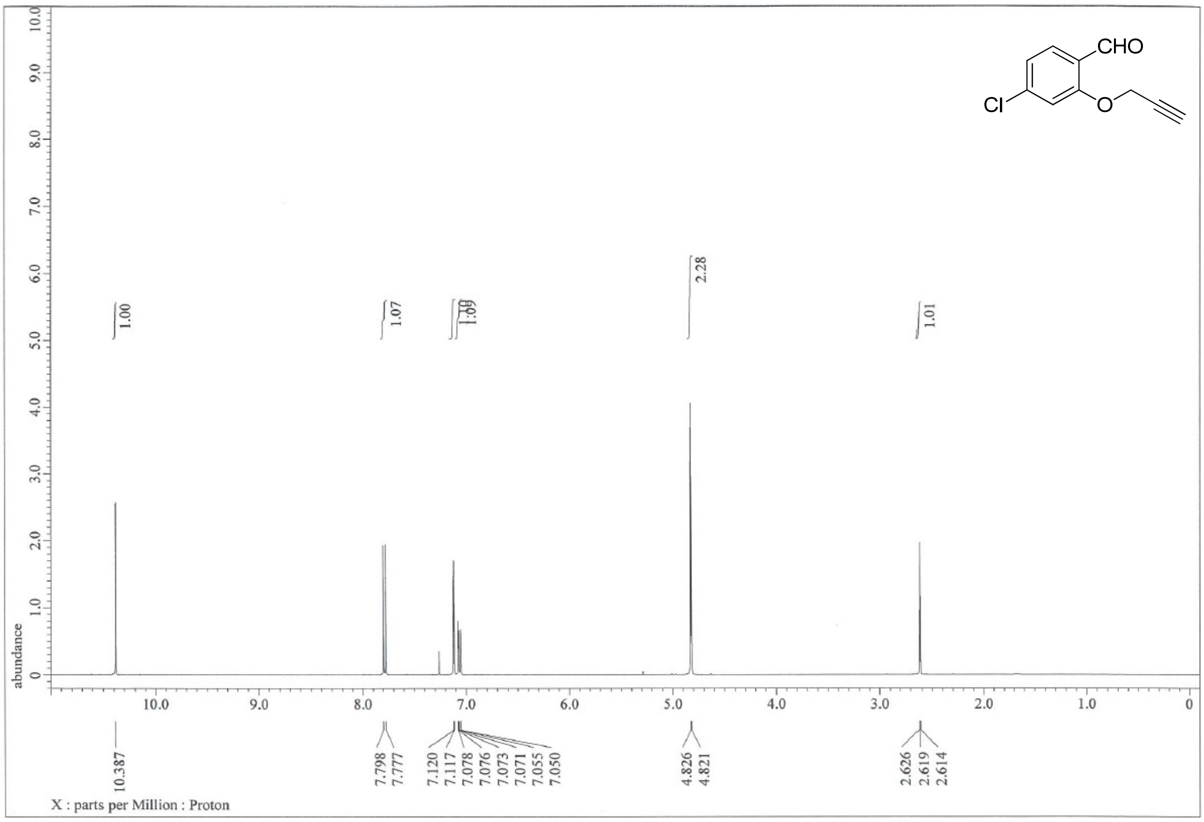

3-Chloro-2-(prop-2-yn-1-yloxy)benzaldehyde (6L)

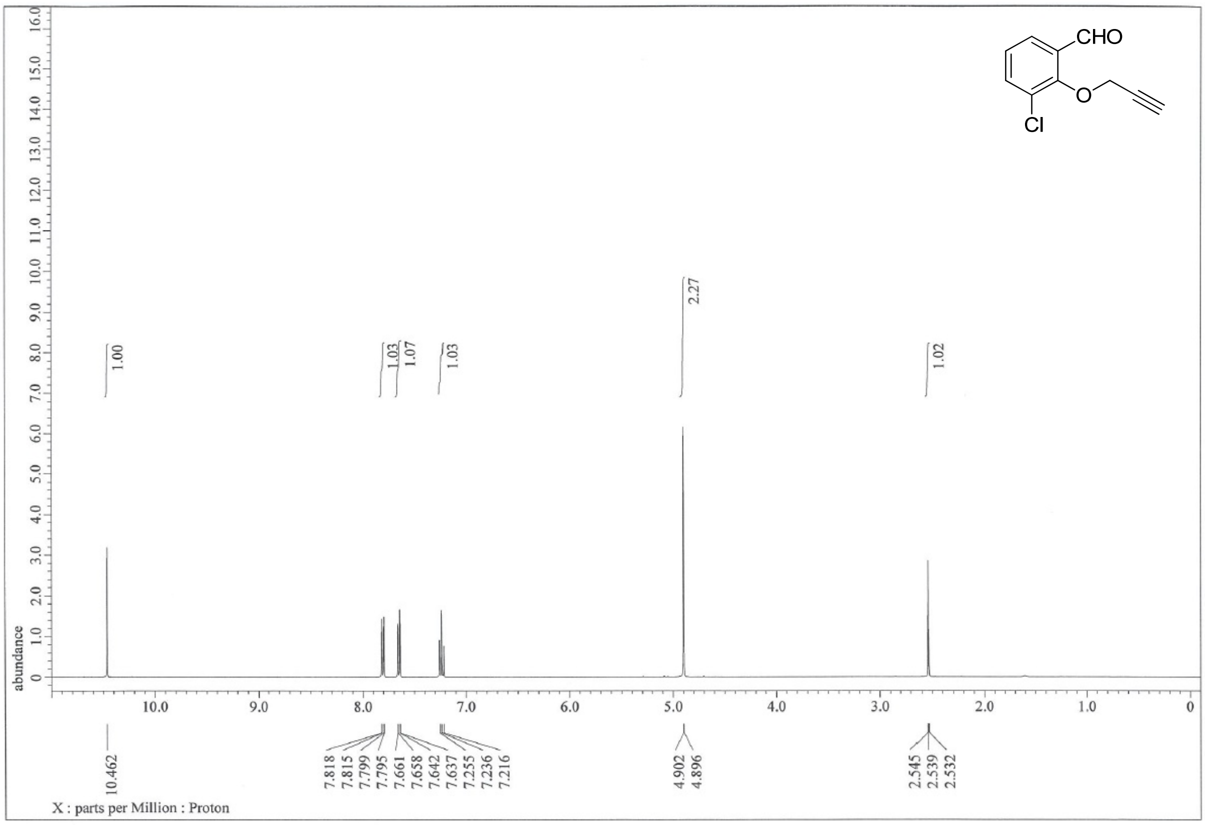

3,5-Dichloro-2-(prop-2-yn-1-yloxy)benzaldehyde (6M)

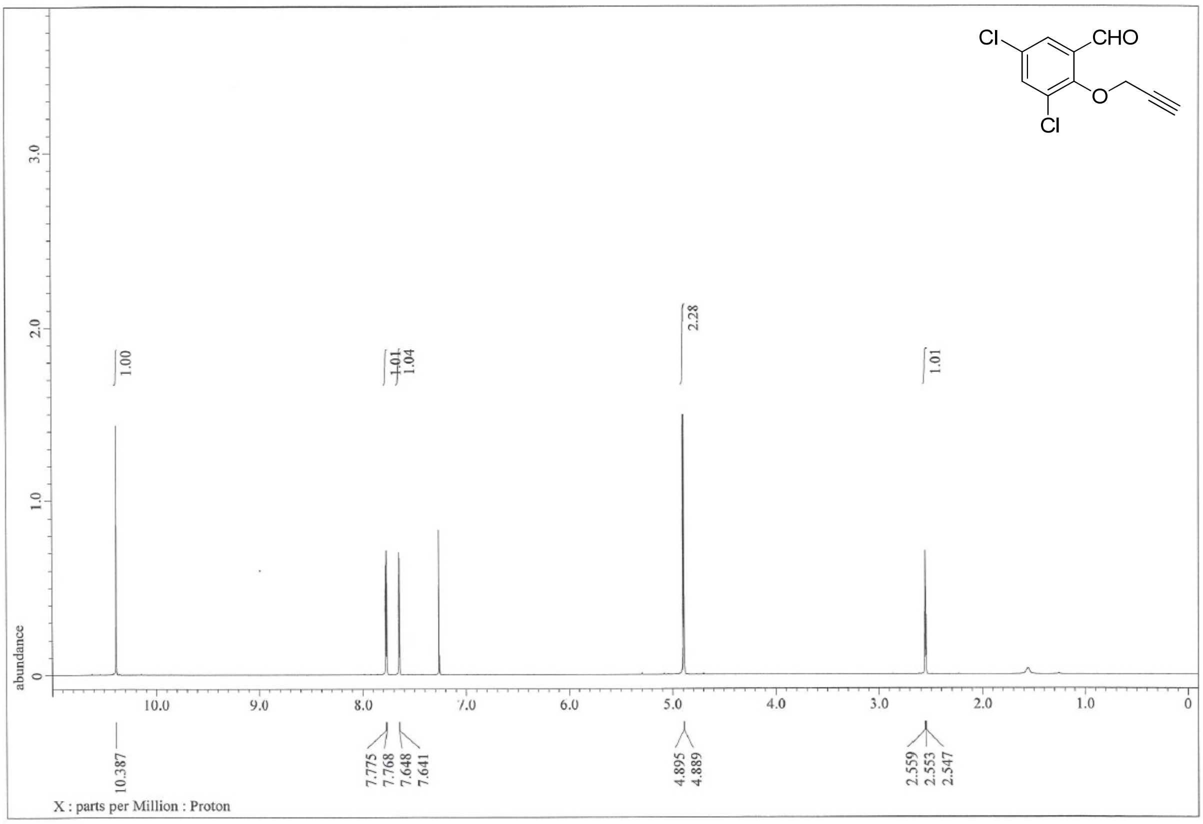

5-Bromo-2-(prop-2-yn-1-yloxy)benzaldehyde (6N)

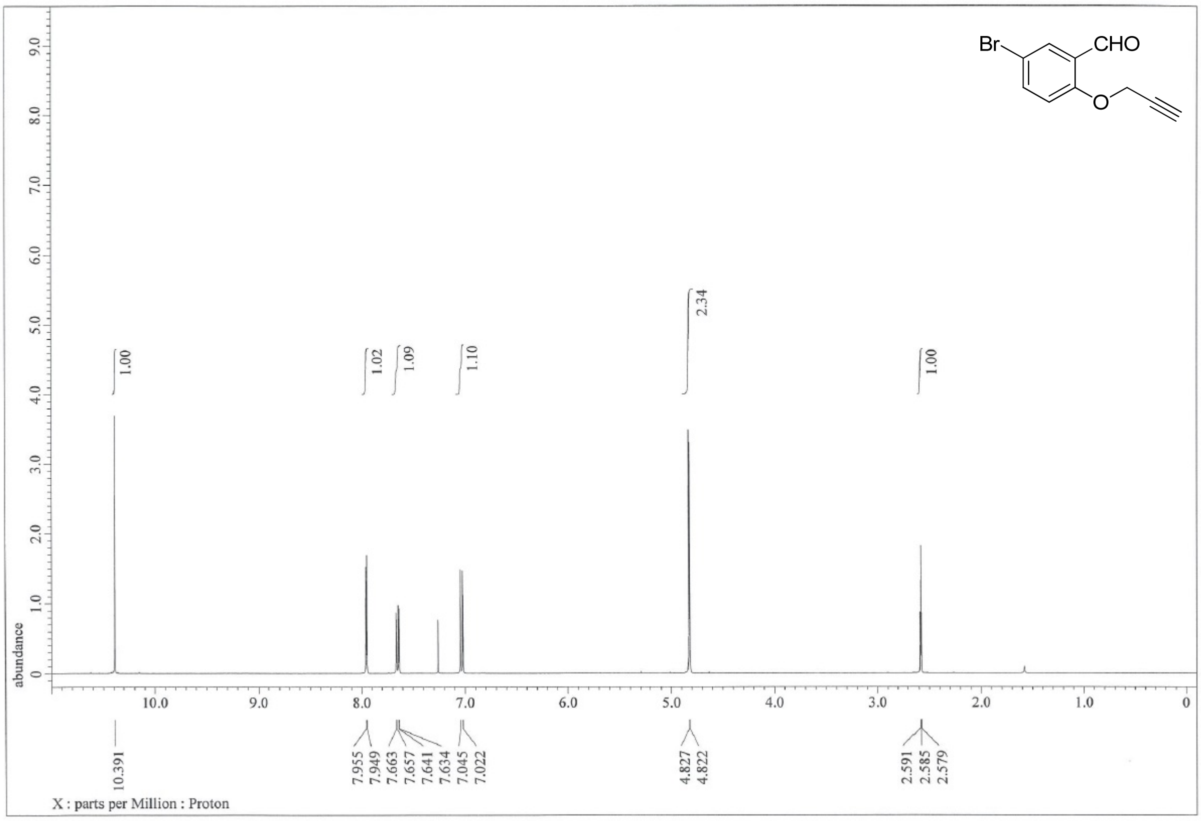

4-Bromo-2-(prop-2-yn-1-yloxy)benzaldehyde (6O)

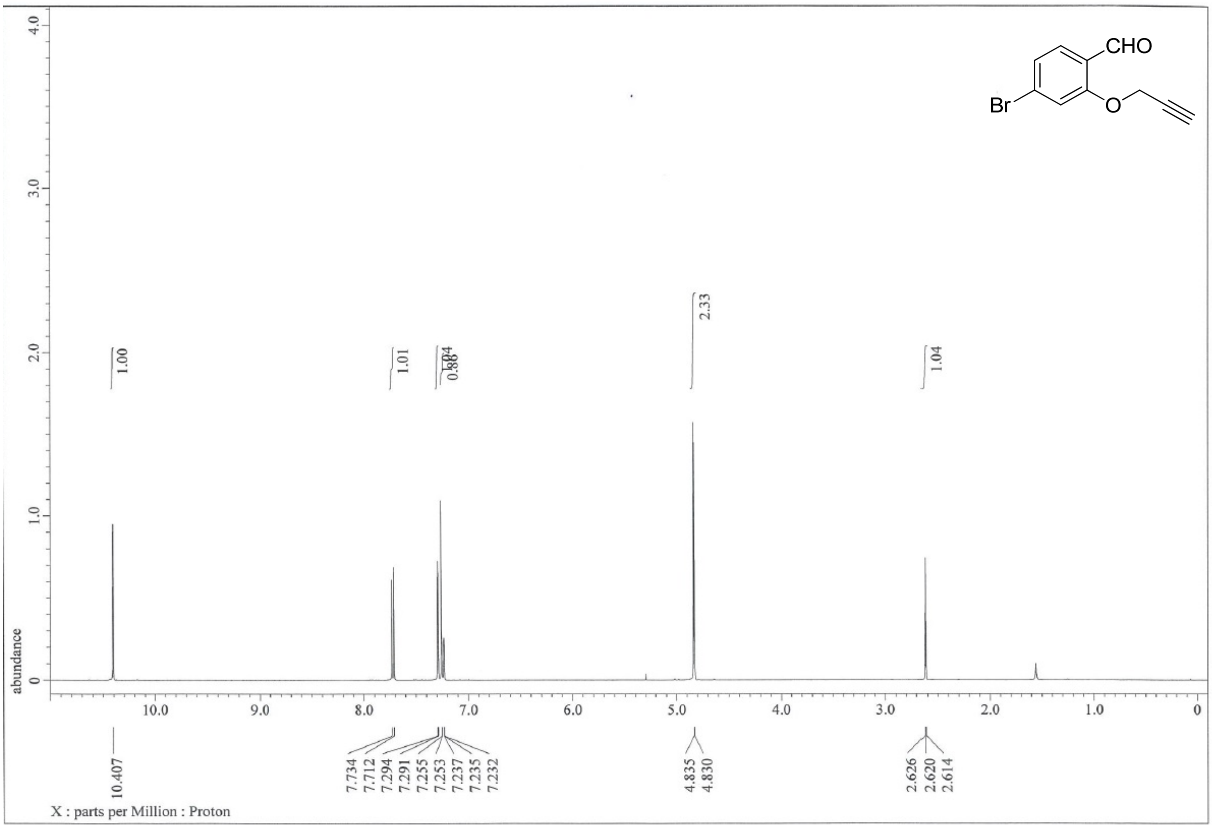

3-Bromo-2-(prop-2-yn-1-yloxy)benzaldehyde (6P)

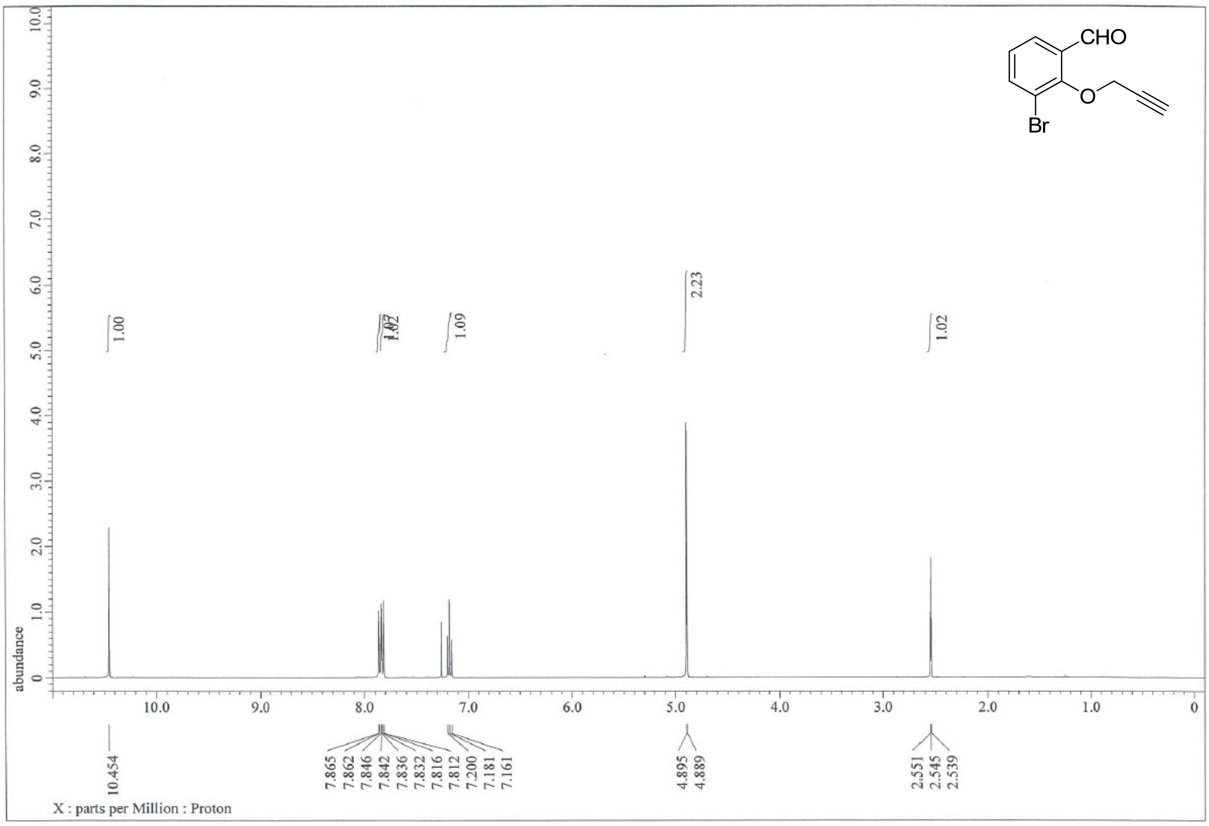

1-(Prop-2-yn-1-yloxy)-2-naphthaldehyde (6Q)

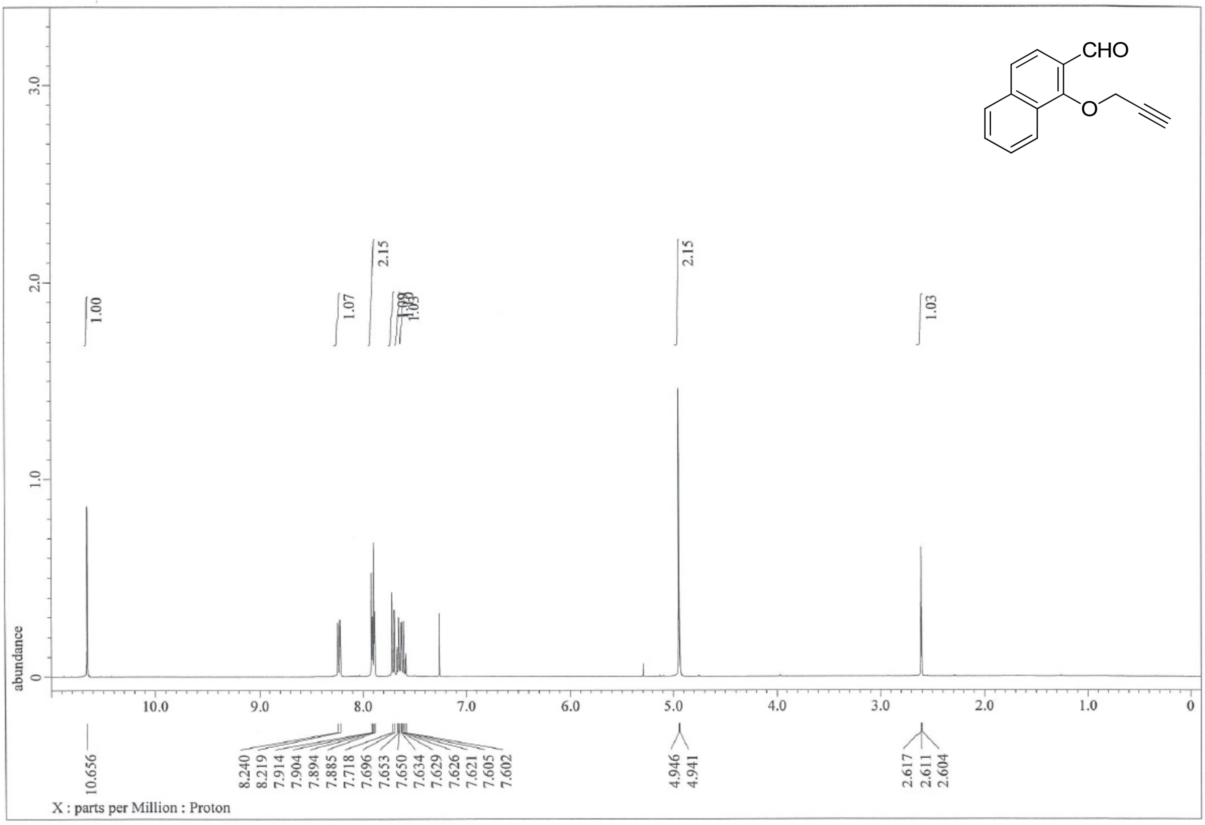

2-(Prop-2-yn-1-yloxy)-1-naphthaldehyde (6R)

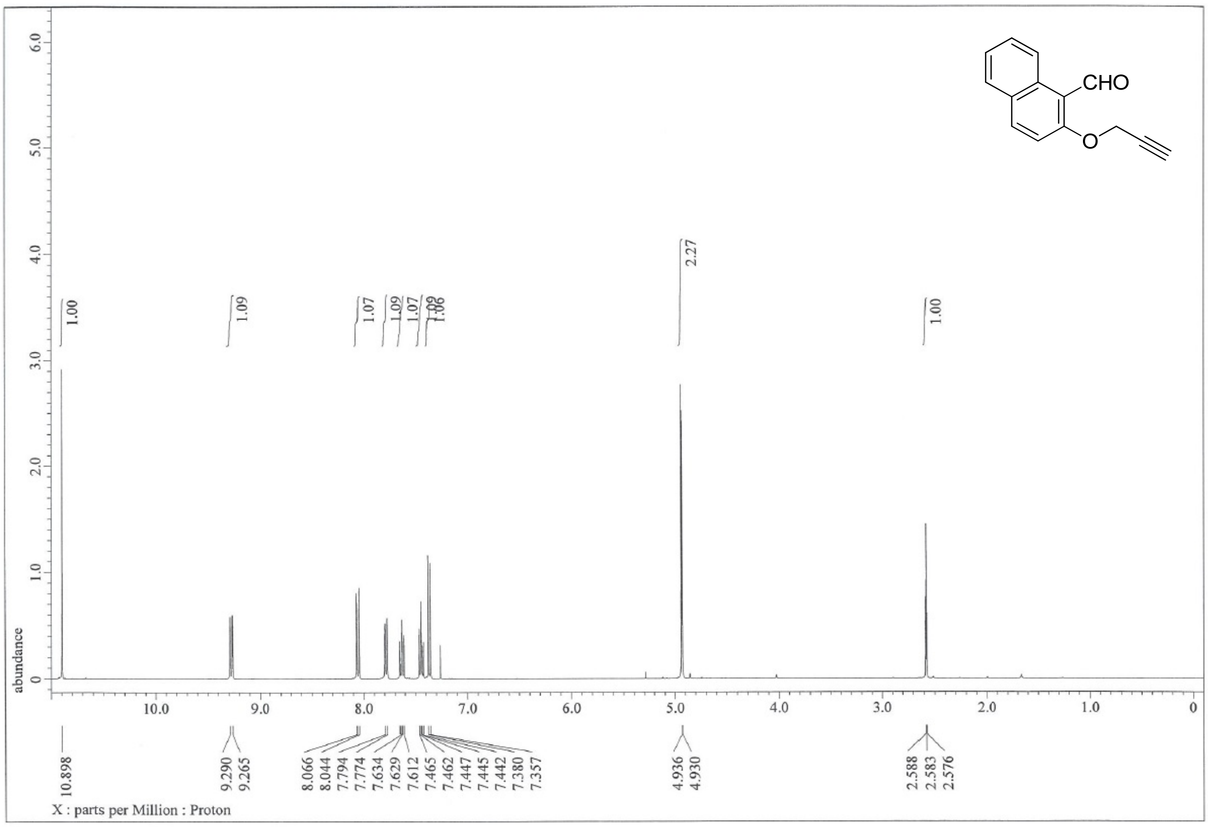

5-Allyl-3-methoxy-2-(prop-2-yn-1-yloxy)benzaldehyde (6S)

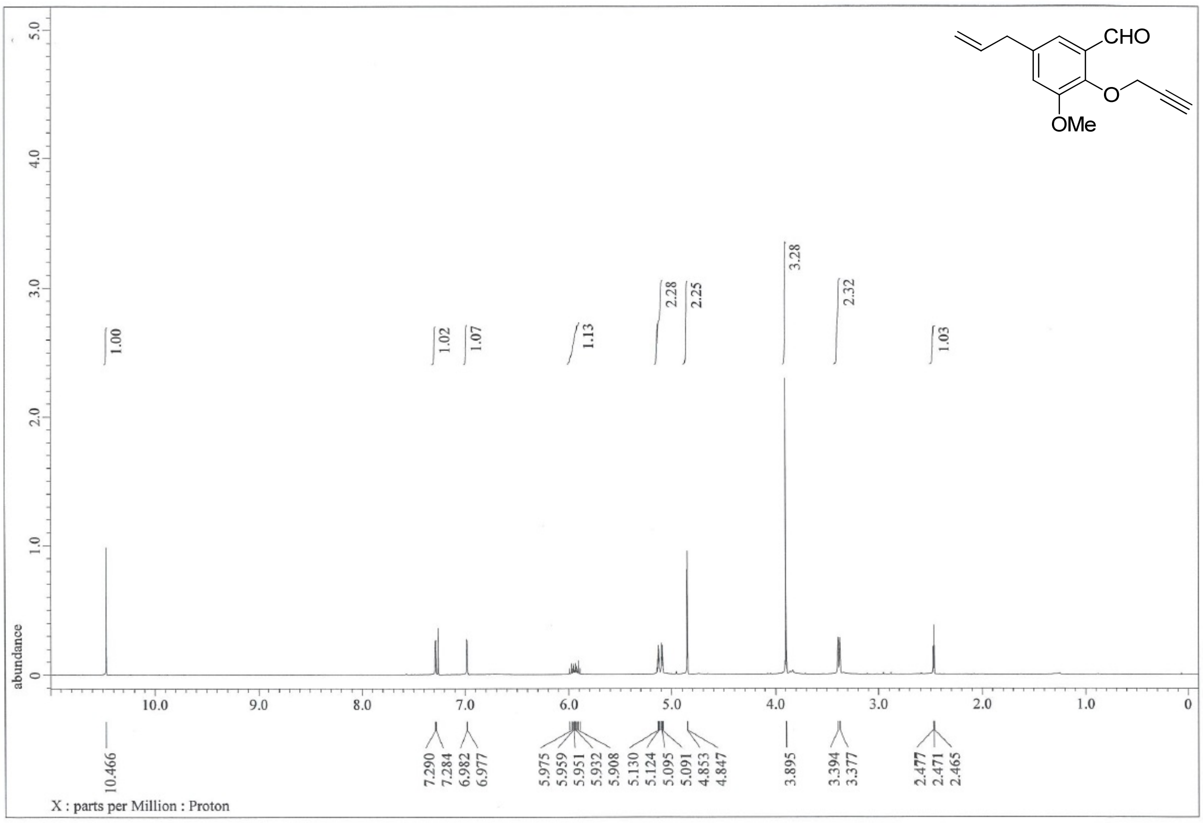

2-(But-2-yn-1-yloxy)-1-naphthaldehyde (6T)

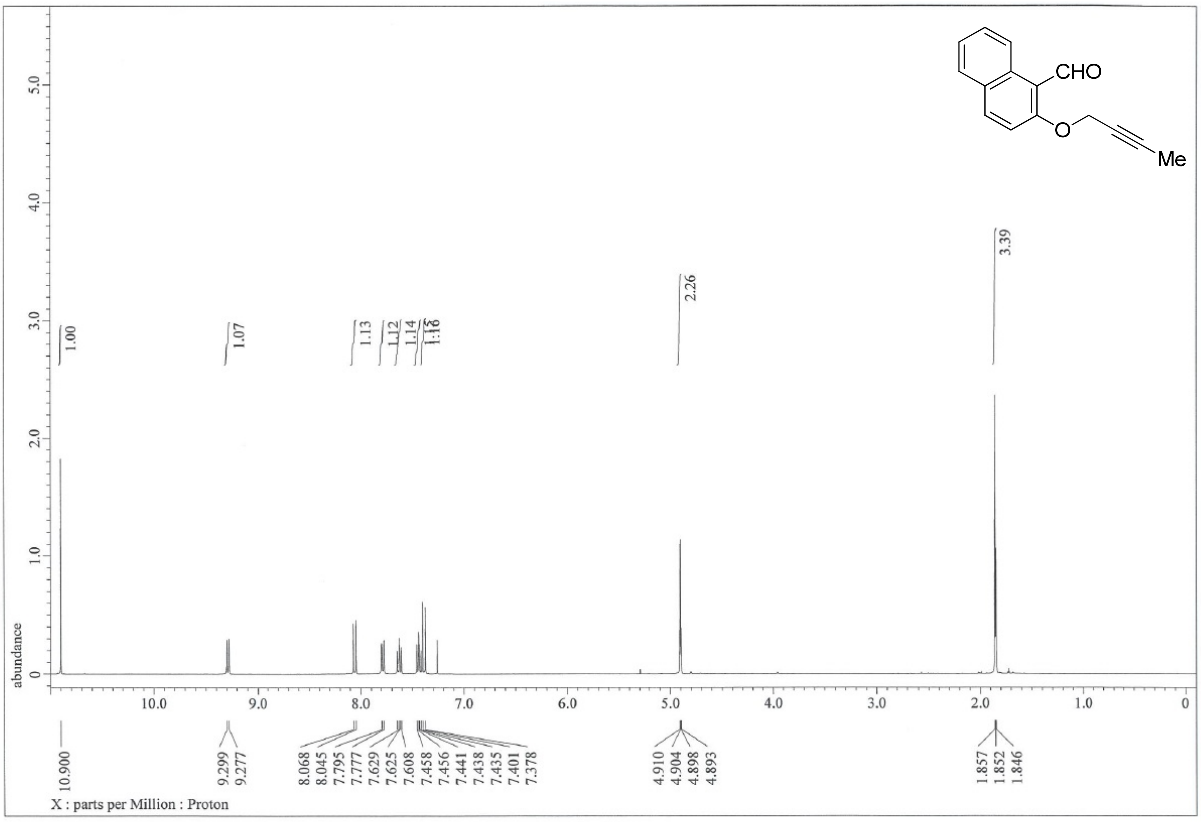

THE END
